# Childhood cancer burden and health inequality: A systematic analysis from the global burden of diseases study 2021

**DOI:** 10.1371/journal.pone.0341303

**Published:** 2026-01-27

**Authors:** Guotian Pei, Yingshun Yang, Shuai Wang, Shushi Meng, Jun Liu, Yuqing Huang

**Affiliations:** 1 Department of Thoracic Surgery, Beijing Haidian Hospital (Haidian Section of Peking University Third Hospital), Beijing, China; 2 Department of Thoracic Surgery, Peking University People’s Hospital, Beijing, China; National Center for Chronic and Noncommunicable Disease Control and Prevention, Chinese Center for Disease Control and Prevention, CHINA

## Abstract

**Objective:**

To estimate the burden, trends, and inequalities of childhood cancers (aged 0–14 years) at global, regional, and national levels from 1990 to 2021.

**Methods:**

We analyzed Global Burden of Diseases Study (GBD) 2021 data, using age-standardized disability-adjusted life years (DALYs) rates (ASDR) to assess childhood cancer burden across 204 countries and territories, grouped into 21 GBD regions by the socio-demographic index (SDI). Temporal trends were assessed using Joinpoint regression. The slope index of inequality and concentration index were calculated to quantify absolute and relative inequalities in the disease burden.

**Results:**

In 2021, childhood cancer caused 70.45 million (95% uncertainty interval [UI]: 57.7–82.79) DALYs globally, with 98.6% attributed to years of life lost. Global ASDR declined from 664.31 (95% UI: 552.99–785.90) in 1990 to 354.06 (95% UI: 289.08–417.49) per 100,000 in 2021. Low-SDI countries had the highest ASDR (467.41, 95% UI: 342.00–589.20), reflecting challenges in diagnosis, treatment, and healthcare access. Acute lymphoblastic leukemia dominated the burden in children under 5, while brain cancers were more common in the 10−14 age group. Boys exhibited higher ASDR (395.55, 95% UI: 307.57–477.68) versus girls (309.80, 95% UI: 253.75–364.91). Socioeconomic inequalities widened, with DALYs concentration index shifting from −0.03 (95% CI: −0.06–0.01) in 1990 to −0.13 (95% CI: −0.16 - −0.11) in 2021, reflecting disproportionate burdens in low-SDI countries.

**Conclusions:**

Persistent disparities in childhood cancer outcomes highlight systemic inequities in healthcare access. High-SDI countries achieved significant mortality reductions, while low-SDI countries face escalating burdens due to delayed diagnoses and fragmented care. Prioritizing cost-effective innovations, strengthening healthcare infrastructure, and implementing gender-sensitive policies are critical to achieving Sustainable Development Goals 3.4 targets. Global collaboration to expand cancer registries and equitable resource allocation is urgently needed to mitigate disparities.

## Introduction

With the global shifts in population demographics and lifestyle patterns, non-communicable diseases (NCDs) have emerged as a major threat to global health. Among children, cancers and other NCDs pose significant public health challenges due to their rising incidence and mortality rates [1]. According to the World Health Organization (WHO), approximately 400,000 children are newly diagnosed with cancer each year, with a disproportionate burden observed in low- and middle-income countries (LMICs) [2]. Childhood cancers not only severely impact the health and well-being of affected children and their families but also represent a critical obstacle to achieving the Sustainable Development Goals (SDGs). Specifically, SDG 3.4 sets an ambitious target of reducing premature mortality from NCDs by one-third by 2030, emphasizing the urgent need for research into the epidemiology, prevention, and treatment of childhood cancers [3].

The WHO’s Global Initiative for Childhood Cancer prioritizes improving survival rates to at least 60% by 2030, particularly focusing on six highly curable pediatric cancers, including acute lymphoblastic leukemia and certain brain tumors [4–7]. While significant progress has been made in high-income countries due to advances in early diagnosis and treatment, stark global disparities persist. Children in low-income regions face substantial challenges, such as delayed diagnosis, limited access to effective therapies, and poor healthcare infrastructure, resulting in higher mortality rates and worse outcomes [8–10]. Understanding these inequalities and their driving factors is essential for designing effective interventions to bridge the gap.

This study utilizes the Global Burden of Disease (GBD) 2021 database to systematically analyze the trends in incidence, mortality, disease burden, and health inequalities of childhood cancers (ages 0–14) from 1990 to 2021. By focusing on regional disparities, socio-economic factors, and the six highly curable cancer types identified by the WHO, the study aims to uncover critical drivers of the observed disparities in childhood cancer outcomes. Employing advanced statistical models and robust methodologies, this research provides actionable evidence to inform targeted prevention and treatment strategies. These findings will offer novel perspectives for policymakers and global health experts, contributing to the reduction of childhood cancer burden and the achievement of global health equity.

## Methods

### Data sources and study design

This study utilized data from the GBD 2021 database to analyze the global burden of childhood cancers in the 0–14 age group. The GBD database provides standardized health metrics, including age-standardized incidence rate (ASIR), age-standardized mortality rate (ASMR), and age-standardized disability-adjusted life years (DALYs) rate (ASDR), across 204 countries and territories. Data sources included vital registration systems, cancer registries, surveys, and verbal autopsies. The burden of childhood cancers was assessed by country, region, and Socio-demographic Index (SDI) quintiles, stratified by sex and age groups (<5 years, 5–9 years, and 10–14 years). Ethical approval was not required since the study utilized publicly available data and did not involve personal information.This study adhered to the Guidelines for Accurate and Transparent Health Estimates Reporting (GATHER) [11].

### Disease classification and study population

The childhood age group in this analysis was defined as 0–14 years. Data specific to this age range were obtained from the GBD Compare Tool and GBD Results Tool [12], with additional processing steps applied to ensure completeness and accuracy. Cancers were classified according to the International Classification of Diseases, 10th Revision (ICD-10) [13]. The analysis primarily targeted major cancer types, including acute lymphoid leukemia (ICD-10: C91.0), acute myeloid leukemia (C92.0), non-Hodgkin lymphoma (C82-C85), Hodgkin lymphoma (C81), and brain and central nervous system cancers (C70-C72), along with other solid tumors. Only malignant neoplasms were included, with non-melanoma skin cancers excluded. This classification ensured consistency and comparability across age, sex, location, and time.

### Estimation of cancer burden

Childhood cancer mortality rates were estimated using data from cancer registries, vital registration systems, and verbal autopsy data. Mortality-to-incidence ratios (MIRs) were applied to derive mortality estimates in regions with limited direct data. MIRs were modeled using spatiotemporal Gaussian process regression to account for variations by age, sex, region, and time, enhancing accuracy and consistency. Garbage codes were redistributed to appropriate causes to ensure reliable cause-specific mortality estimates.

DALYs were calculated as the sum of years of life lost (YLLs) and years lived with disability (YLDs). YLLs were calculated based on the number of deaths and the reference life expectancy at the age of death, following GBD standard life tables. YLDs were derived by multiplying cancer prevalence by disability weights, which reflected the severity of health states during different cancer stages (diagnosis, treatment, remission, and terminal phases). Disability weights ranged from 0 (indicating full health) to 1 (indicating death). Detailed methods and technical approaches are provided in the Supporting information File and previous GBD publications [14–16].

### Trends and inequality analysis

Joinpoint regression was used to estimate the Annual Average Percentage Change (AAPC) in ASIR, ASMR, and ASDR from 1990 to 2021, with statistical significance assessed using 95% confidence intervals (CIs) [17,18]. To evaluate health inequalities, concentration indices were calculated across SDI quintiles. Positive values of the concentration index indicated a higher disease burden concentrated in high-SDI countries, while negative values reflected a higher burden in low-SDI countries. These methods provided a robust framework for assessing temporal trends and disparities in childhood cancer outcomes.

### Data analysis and uncertainty estimation

All metrics were estimated using the standard GBD modeling framework, which employs Bayesian meta-regression models (e.g., DisMod-MR 2.1) to synthesize data from multiple sources. Uncertainty intervals (UIs) were derived from 1,000 Monte Carlo simulations conducted at each step of the estimation process and were reported as the 2.5th and 97.5th percentiles of the distribution. These intervals accounted for both stochastic and systematic uncertainty, ensuring robust and reliable estimates. Data analysis and visualization were conducted using R software (version 4.2.2). Statistical significance for all analyses was defined as *p* < 0.05.

## Results

### Global burden of childhood cancer in 2021

**Fig 1:**
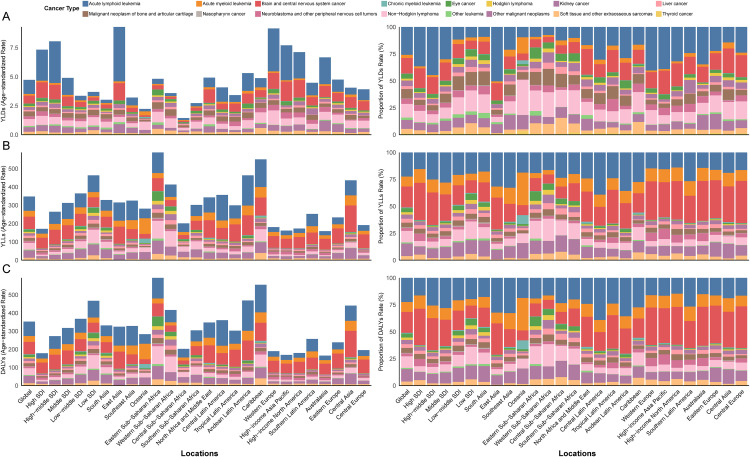
Age-standardized Rates of YLDs, YLLs, and DALYs due to Childhood Cancers by Global, SDI Levels, and GBD Regions, 2021. Panels A, B, and C present the age-standardized rates and proportional burden of childhood cancers (ages 0-14) in terms of YLDs, YLLs, and DALYs, respectively, across different GBD regions and SDI quintiles in 2021. YLDs = Years Lived with Disability, YLLs = Years of Life Lost, DALYs = Disability-Adjusted Life Years, GBD = Global Burden of Disease, Injuries, and Risk Factors Study.

In 2021, childhood cancer in the 0–14 age group posed significant global health challenges, characterized by distinct epidemiological patterns. The global ASIR was 166.28 per 100·000 population (95% UI: 114.64 to 232.71), corresponding to approximately 3.37 million new cases (S2 Table). In contrast, the global ASMR was substantially lower at 4.18 per 100·000 (95% UI: 3.42 to 4.91), accounting for an estimated 83.340 deaths (95% UI: 68.600 to 97.700) (S2 Table). The total burden of childhood cancer, measured by DALYs, reached 70.45 million (95% UI: 57.73 to 82.79 million), with over 98.6% attributable to YLLs and less than 1.4% to YLDs (Figs 1; S1 and S1 Table). Sex-based differences were notable. Although girls had a higher ASIR (212.63; 95% UI: 145.13 to 298.52) compared to boys (122.78; 95% UI: 86.06 to 171.19), boys exhibited greater disease severity and mortality risk. The ASMR for boys was 4.67 per 100000 (95% UI: 3.65 to 5.62), significantly exceeding that of girls (3.65; 95% UI: 3.00 to 4.30). Similarly, boys had a higher ASDR of 395.55 (95% UI: 307.57 to 477.68) compared to girls at 309.80 (95% UI: 253.75 to 364.91) (S1 Fig). These findings highlight marked gender disparities in the incidence, severity, and outcomes of childhood cancers, underscoring the global burden of these diseases.

**Fig 2:**
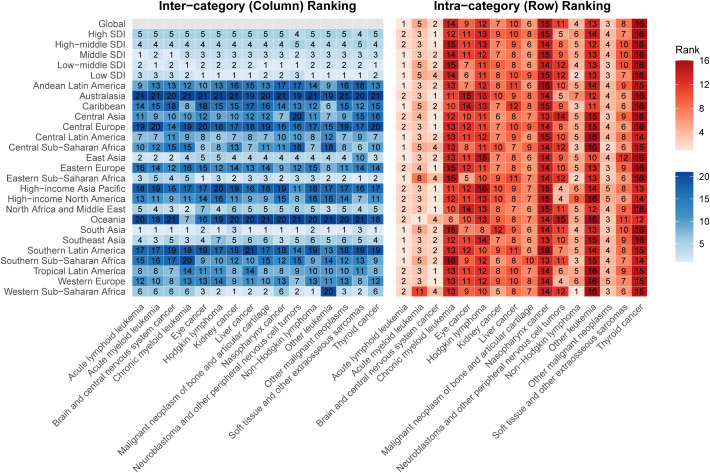
Childhood Cancers Ranked by Number of DALYs for Both Sexes Combined, 2021. Inter-category ranking refers to vertical ranking across categories, including SDI quintiles (ordered from high to low) and the 21 GBD regions (ordered alphabetically). Intra-category ranking refers to horizontal ranking within categories, specifically within each SDI quintile and GBD region. Colour intensity is proportional to the absolute number of DALYs within each ranking category (columns or rows). Numerical rankings represent the total absolute DALYs, where rank 1 indicates the highest burden. SDI = Sociodemographic Index. DALY = disability-adjusted life-year. GBD = Global Burden of Disease, Injuries, and Risk Factors Study.

By SDI category, high-SDI countries had the highest ASIR (280.9 per 100000) but benefited from advanced diagnostic and treatment capabilities, resulting in the lowest ASMR (2.09 per 100000) and ASDR (181.35 per 100000) (S1 and S2 Tables, S2 Fig). Brain and central nervous system cancers were the primary contributors to the disease burden in these countries (Fig 2). In contrast, low-SDI countries had the lowest ASIR (90.36 per 100000) but the highest ASMR (5.50 per 100000) and ASDR (467.41 per 100000), reflecting a high prevalence of late-stage diagnoses and severe resource limitations (S1 and S2 Tables, S2 Fig). Acute myeloid leukemia (AML) was the major contributor to the disease burden in low-SDI countries (Fig 2). At the regional level, Central Europe had the highest ASIR (S2 Table, S3 Fig), with brain and central nervous system cancers being the primary contributors to the disease burden, while Eastern Sub-Saharan Africa had the highest ASMR and ASDR, primarily driven by acute lymphoid leukemia (Fig 2). At the national level, in 2021, countries with the highest ASIR were concentrated in Central and Eastern Europe (e.g., Poland and Romania, S3 Table, S4 Fig), while those with the highest ASMR and ASDR were predominantly located in Sub-Saharan Africa and small island nations (e.g., Tokelau and Niue, S3 Table, S5 and S6 Figs), reflecting significant disparities in disease diagnosis and treatment capabilities across regions.

An analysis of age distribution reveals that girls have a higher age-specific incidence rate, while boys experience greater age-specific mortality and DALY rates. The number of new cases rises with age, reaching its peak in the 10–14 age group, whereas mortality and DALYs are highest in the < 5 age group and steadily decline with increasing age. Acute lymphoid leukemia dominates the disease burden in children under 5, while brain and central nervous system cancers become the leading contributors in the 10–14 age group (S2 Table, S7 Fig).

### Temporal trends in childhood cancer burden from 1990 to 2021

**Fig 3:**
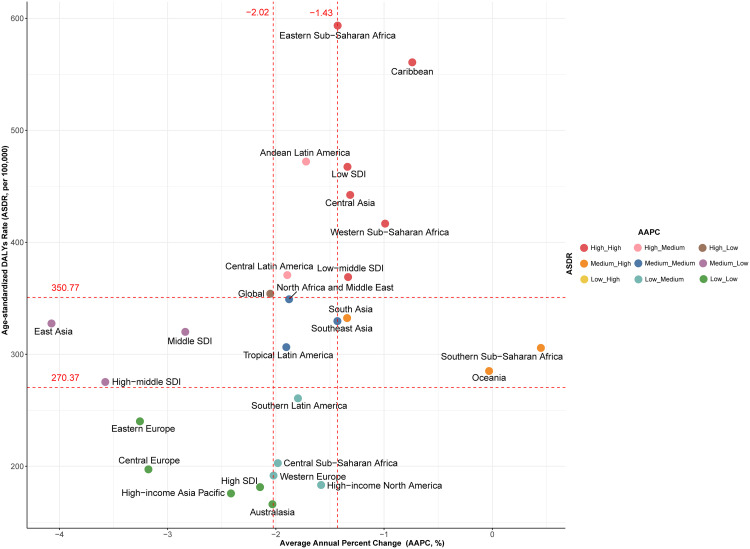
Relationship Between Age-standardized DALYs Rates and Average Annual Percent Change for Childhood Cancers Across SDI Categories and GBD Regions, Both Sexes Combined, 2021. This scatter plot illustrates the correlation between ASDR and AAPC for childhood cancers in 2021, across 21 GBD regions and five SDI levels. The vertical dashed red lines represent the 1/3 (−2.02%) and 2/3 (−1.43%) quantiles of AAPC, while the horizontal dashed red lines indicate the 1/3 (270.37 per 100,000) and 2/3 (350.77 per 100,000) quantiles of ASDR. Each point represents a region or SDI level, with color-coded categories reflecting nine combinations of ASDR and AAPC classifications: Low_Low, Low_Medium, Low_High, Medium_Low, Medium_Medium, Medium_High, High_Low, High_Medium, and High_High. Regions in the top-right quadrant (e.g., High_High) exhibit high age-standardized DALYs rates (ASDR) and high average annual percent change (AAPC), indicating both a high burden of childhood cancers and an increasing trend in the DALYs over time. Conversely, regions in the bottom-left quadrant (e.g., Low_Low) show low ASDR and low AAPC, reflecting a lower burden of childhood cancers and slower changes in DALYs over time. DALYs = disability-adjusted life years; AAPC = average annual percent change; ASDR = age-standardized DALYs rate; SDI = Sociodemographic Index; GBD = Global Burden of Disease, Injuries, and Risk Factors Study.

Globally, from 1990 to 2021, the ASIR of childhood cancers remained stable, while the ASMR (from 7.81 to 4.18) and ASDR (from 664.31 to 354.06) declined significantly, with an AAPC of −2.04% (95% CI: −2.27% to −1.81%) and −2.05% (95% CI: −2.28% to −1.82%), respectively. High SDI countries demonstrated markedly greater reductions in ASMR (from 4.09 to 2.09) and ASDR (from 348.35 to 181.35) compared to low SDI countries, highlighting regional disparities in diagnostic and treatment capabilities (S1 and S2 Tables, S2 Fig). At the regional level, East Asia showed the most significant improvements, with ASMR and ASDR reductions exceeding the global average (AAPC: −4.07, 95% CI: −4.39 to −3.75; −4.07, 95% CI: −4.40 to −3.75, respectively), providing valuable lessons for other regions. In contrast, low-income regions such as sub-Saharan Africa and the Caribbean exhibited limited declines in ASMR and ASDR, underscoring the need for enhanced public health interventions and resource allocation (Figs 3, S8, S1 and S2 Tables). For ASIR, Tropical Latin America displayed the largest decline (AAPC: −1.18, 95% CI: −1.27 to −1.10). In comparison, ASIR trends in high-income regions were relatively stable, while low-income regions such as sub-Saharan Africa showed minimal change, reflecting slow progress in reducing disease burden (S2 Table, S9 Fig). At the national level, high-income countries, such as Luxembourg and Hungary, have made significant progress in reducing childhood cancer mortality rates and disease burden. However, low-income and resource-limited countries, such as Botswana and Dominica, continue to face challenges of increasing disease burden, highlighting global health disparities and inequities in healthcare resource allocation (S3 Table).

**Fig 4:**
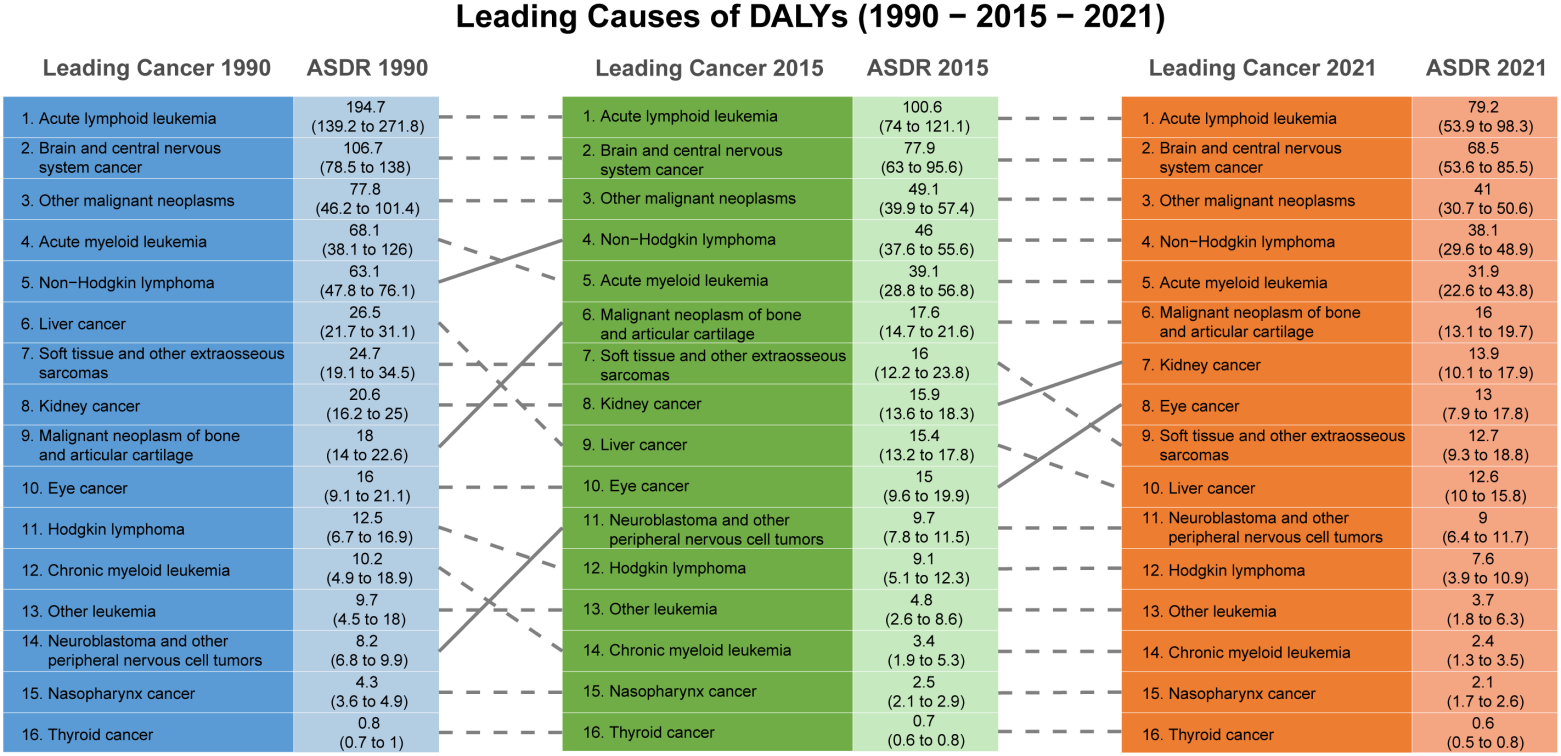
Leading Causes of DALYs for Childhood Cancers (1990-2015-2021). This figure shows the leading causes of DALYs for childhood cancers from 1990 to 2021. The leftmost column presents the leading cancer types and their corresponding ASDR in 1990, while the middle column shows the data for 2015, and the rightmost column presents the 2021 data. The numbers for each cancer type are accompanied by their ASDR values in parentheses. The dashed lines highlight the transitions between the top-ranked cancers across the years, illustrating trends in the leading causes of DALYs in childhood cancers. The chart emphasizes the shifts in childhood cancer burdens over the three decades. DALYs = disability-adjusted life years; ASDR = age-standardized DALYs rate.

From the perspective of cancer types, chronic myeloid leukemia and acute myeloid leukemia exhibited the most significant global reductions in ASMR (S4 Table) and ASDR (Fig 4; S4 Table). Hodgkin lymphoma showed remarkable declines in burden in high-middle SDI countries, while improvements in low-SDI countries were limited. In Central Asia, brain and central nervous system cancers displayed an increasing burden, whereas acute lymphoid leukemia in Central Europe achieved the most pronounced reductions in burden (S4 Table).

### Global health inequalities in childhood cancer outcomes

**Fig 5:**
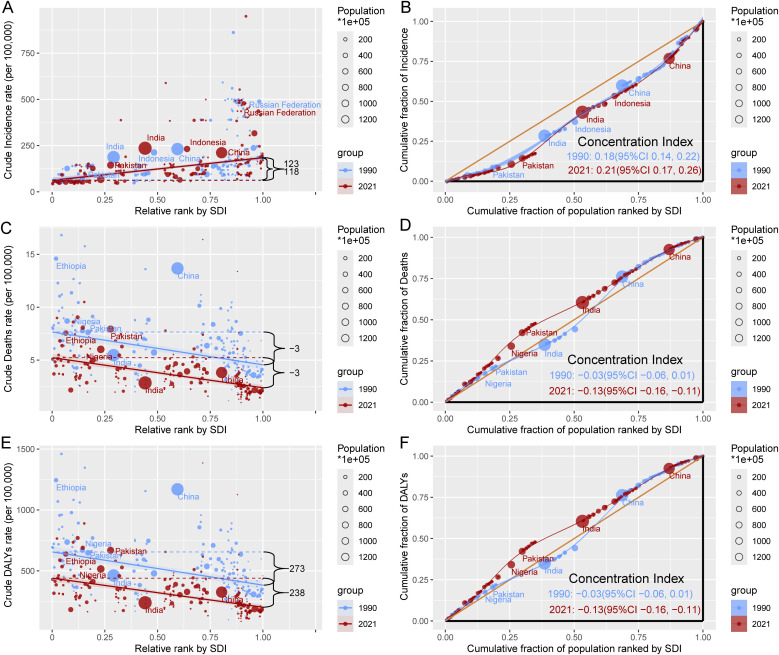
Health Inequality Regression Curves and Concentration Curves for the Incidence, Mortality, and DALYs of Childhood Cancer. Panels A and B show the health inequality regression curves and concentration curves for the incidence of childhood cancer, respectively. Panels C and D display the health inequality regression curves and concentration curves for childhood cancer mortality, respectively. Panels E and F illustrate the health inequality regression curves and concentration curves for DALYs due to childhood cancer, respectively. DALYs = disability-adjusted life years; SDI = Sociodemographic Index.

From 1990 to 2021, the global crude incidence rate of childhood cancers increased from 117.90 (95% CI: 91.54 to 144.27) to 123·25 (95% CI: 94.30 to 152.19) per 100000, with the concentration index rising from 0.18 (95% CI: 0.14 to 0.22) to 0.21 (95% CI: 0.17 to 0.26). This indicates that cases are increasingly concentrated in high-SDI countries due to their superior diagnostic capabilities. In contrast, crude mortality rates declined significantly from −3.10 (95% CI: −4.03 to −2.17) to −2.85 (95% CI: −3.38 to −2.32), while the concentration index decreased from −0.03 (95% CI: −0.06 to 0.01) to −0.13 (95% CI: −0.16 to −0.11), reflecting a worsening disparity in mortality rates in low-SDI countries. Similarly, DALYs showed a decline in the global burden, with the concentration index dropping from −0·03 (95% CI: −0.06 to 0.01) to −0.13 (95% CI: −0.16 to −0.11). While the incidence rate increased, mortality rates and DALYs improved significantly, particularly in high-SDI countries. However, inequality in mortality and DALYs remains severe in low-SDI countries (Fig 5, S5 Table).

Among specific childhood cancer types, acute lymphoid leukemia showed a significant increase in incidence, rising from 1.30 (95% CI: 0.75 to 1.86) to 1.70 (95% CI: 1.18 to 2.23) per 100000, with the concentration index increasing from 0.17 to 0.32, reflecting improved diagnostic capacity in high-SDI countries. In contrast, mortality rates and DALYs in low-SDI countries demonstrated a sharp disparity, with the concentration index dropping to −0.07 and −0.07, respectively (S5 Table, S10 Fig). Brain and central nervous system cancers exhibited a modest rise in incidence, from 1.82 in 1990 to 1.91 in 2021, with the concentration index increasing to 0.26. Despite this, low-SDI countries, such as Nigeria, continued to face disproportionately high mortality and disease burdens (S5 Table, S11 Fig). Furthermore, Non-Hodgkin lymphoma showed worsening inequality in mortality and DALYs, with concentration indices decreasing to −0.37 and −0.37, respectively (S5 Table, S12 Fig). These findings highlight the critical need to improve diagnostic and treatment resources in low-SDI regions to address the persistent disparities in childhood cancer outcomes.

## Discussion

This study is the first to comprehensively analyze trends in the global burden of childhood cancers (ages 0–14) from 1990 to 2021 using DALYs as a key measure to highlight inequities across countries with differing SDI. Leveraging robust, globally standardized data from the GBD database, covering 204 countries and territories, the analysis reveals that while the global ASIR has remained stable, the ASMR and ASDR have significantly declined, primarily in high-SDI countries. In contrast, low-SDI countries continue to experience disproportionately higher mortality and disease burden, reflecting systemic barriers such as delayed diagnoses, inadequate healthcare infrastructure, and limited access to effective treatments. Gender disparities were observed, with boys showing higher ASMR and ASDR, potentially linked to immune response differences, tumor aggressiveness, or delayed access to care. These findings emphasize the urgent need for targeted interventions, including cancer registries, early diagnostic programs, and equitable treatment access. This study directly supports the achievement of SDG 3.4, providing critical evidence to reduce childhood cancer mortality and advance global health equity.

Our study comprehensively analyzed the global burden of childhood cancers from 1990 to 2021, revealing significant geographic, socioeconomic, and gender disparities. These findings align with existing literature while offering novel insights into underlying mechanisms and potential intervention strategies. Disparities in childhood cancer outcomes between high-SDI and low-SDI countries have been consistently reported in prior studies, attributed to late-stage diagnoses, limited access to treatments, and inadequate healthcare infrastructure [19–21]. Our results corroborate these disparities but further highlight the slower reductions in mortality rates in low-SDI countries compared to high-SDI countries, emphasizing the urgent need for equitable resource allocation and tailored interventions [7,21]. These findings underscore the intertwined nature of systemic barriers and health outcomes. Addressing these challenges requires a comprehensive approach that integrates evidence-based interventions with scalable innovations.

Socioeconomic inequalities remain a pervasive challenge. Research indicates that higher human development index correlates with improved survival rates, with disparities most pronounced in resource-limited settings [22–24]. Our analysis supports these findings, demonstrating the critical role of interventions like improved diagnostic infrastructure, subsidized treatment, and capacity-building initiatives in bridging these gaps [25–27]. However, existing strategies often encounter scalability challenges in underserved regions. Expanding initiatives such as “twinning programs,” which foster partnerships between high- and low-SDI countries, and integrating these with digital health tools like telemedicine, could significantly enhance access and outcomes in these areas. For instance, a pilot program in sub-Saharan Africa demonstrated the feasibility of using mobile health units to deliver early diagnostic services for childhood cancers, significantly improving survival rates [28]. Similarly, AI algorithms for prioritizing high-risk cases and optimizing treatment allocation in low-resource environments represent a promising avenue, as evidenced by recent trials in South Asia where machine learning tools improved early diagnosis rates by 25% [29].

Gender disparities in childhood cancer outcomes, with boys exhibiting higher mortality and disease burden, are consistent with earlier findings [16,30-31–]. Biological mechanisms, such as immune response differences and tumor aggressiveness, along with sociocultural factors, contribute to these discrepancies. For instance, in South Asia, cultural norms sometimes favor delayed care for boys due to perceived resilience, exacerbating their risk [31,32,33]. Region-specific gender-sensitive healthcare policies, such as culturally adapted awareness campaigns, have shown promise in addressing these biases in India, reducing diagnostic delays by 15% [25]. Future research should explore the role of sex hormones and immune modulation in tumor progression to support personalized treatment strategies for boys and girls. Additionally, these findings highlight the need for integrating sociocultural education into community health programs to mitigate systemic biases.

Geographic and socioeconomic disparities are also linked to systemic barriers, such as inadequate diagnostic infrastructure and limited treatment access in low-SDI countries. Delayed diagnoses and insufficient treatment exacerbate the burden, particularly for acute lymphoid leukemia and brain cancers [19,34]. High-SDI countries benefit from advanced treatment protocols, including targeted therapies, but these remain inaccessible in resource-limited settings due to cost and logistical challenges [26,35]. Simplified treatment regimens and portable diagnostic tools could be deployed, coupled with community-based screening programs. For example, a low-cost diagnostic device tested in rural Kenya successfully reduced the diagnostic time for childhood leukemia from six weeks to two weeks, showcasing its potential scalability [20]. Global partnerships could further facilitate the transfer of such technologies while addressing sustainability concerns through local capacity-building and training.

Our findings underscore the necessity of integrated approaches that address systemic barriers, leverage global partnerships, and foster technological innovation. Policymakers should prioritize the development of sustainable frameworks for implementing community-based initiatives and enhancing healthcare infrastructure. Establishing a global childhood cancer database with real-time analytics and standardized data sharing could facilitate evidence-based decision-making and international collaboration. For instance, blockchain technology could ensure secure and transparent global data sharing, while AI-driven predictive models could optimize resource allocation and patient management. Pilot projects integrating these technologies in low-SDI countries could serve as a blueprint for broader implementation. By contextualizing these disparities within the broader literature and proposing innovative solutions, this study provides critical evidence for targeted interventions to reduce the global burden of childhood cancers and advance health equity.

This study provides valuable insights into the global burden of childhood cancers, but several limitations should be acknowledged. First, while the GBD database integrates data from diverse sources, cancer registry coverage in low-SDI countries remains inadequate, with regions like sub-Saharan Africa covering less than 20% of the population, potentially leading to substantial underestimations of cancer incidence and mortality. Second, the reliance on model-based estimates, such as those derived from DisMod-MR, introduces uncertainties, particularly in areas with limited primary data. Third, DALYs, while comprehensive, may not fully capture the long-term psychosocial and economic impacts of childhood cancers on families and societies. Finally, this study did not address the potential impacts of global crises, such as COVID-19, which may have disrupted cancer diagnosis and treatment, especially in resource-constrained settings.

Future research should prioritize addressing these gaps. Strengthening cancer registries, particularly in low-SDI countries, is critical to improving data accuracy and guiding resource allocation. Emerging technologies, such as AI-driven predictive models, could further optimize early detection and treatment strategies in underserved regions. Longitudinal studies are also needed to explore the biological and socio-economic drivers of gender and regional disparities in childhood cancer outcomes. Additionally, assessing the resilience of healthcare systems during global crises, such as COVID-19, will provide valuable insights to mitigate future disruptions. These efforts are vital to reducing childhood cancer mortality, addressing global health inequities, and supporting the achievement of SDG 3.4.

## Conclusion

This study provides groundbreaking insights into the global burden of childhood cancers, emphasizing the critical need for equitable interventions and global collaboration. By addressing systemic disparities with innovative solutions, this research lays the foundation for transformative progress in pediatric oncology, driving the global agenda toward health equity and SDG 3.4.

## Supporting information

S1 Fig Trends in Childhood Cancer Incidence, Mortality, and Disability Burden (1990–2021). This figure presents the trends in the global burden of childhood cancers (ages 0–14) from 1990 to 2021, stratified by sex. The top panel shows the number of incidences and age-standardized incidence rates (ASIR) (per 100,000 population). The middle panel displays the number of deaths and age-standardized mortality rates (ASMR), while the bottom panel presents the number of disability-adjusted life years (DALYs) and age-standardized DALY rates.
(TIF)

S2 Fig Trends in ASIR, ASMR, and ASDR for Childhood Cancers (Ages 0–14) Globally and Across Five SDI Categories (1990–2021), Including Average Annual Percentage Change. ASIR = age-standardised incidence rates. ASMR = age-standardised mortality rates. ASDR = age-standardised disability-adjusted life years rates. DALYs = disability-adjusted life years. SDI = Sociodemographic Index.
(TIF)

S3 Fig Trends in Childhood Cancer (Ages 0–14) Incidence, Mortality, and DALYs Across 21 GBD Regions from 1990 to 2021, Including Average Annual Percentage Change. Panel A presents the total number of incident cases, deaths, and DALYs due to childhood cancers (ages 0–14) across 21 GBD regions from 1990 to 2021. Panel B shows the trends in ASIR, ASMR, and ASDR over the same period. Panel C displays the average annual percentage change for incidence, mortality, and DALYs, stratified by sex and GBD regions. ASIR = age-standardized incidence rates; ASMR = age-standardized mortality rates; ASDR = age-standardized disability-adjusted life years rates; DALYs = disability-adjusted life years; GBD = Global Burden of Disease, Injuries, and Risk Factors Study. Mil. = million; K = thousand.
(TIF)

S4 Fig Global Distribution of Age-standardized Incidence Rates for Childhood Cancers Across 204 Countries and Territories, Both Sexes Combined, 2021. This map illustrates the global distribution of age-standardized incidence rates (ASIR) for childhood cancers in 2021, categorized into four quartiles based on ASIR values (per 100,000 population): < 60.22, 60.22 to <95.18, 95.18 to <130.62, and 130.62 to <151.25. The map highlights notable subregions, including the Caribbean and Central America, Persian Gulf, Balkan Peninsula, Southeast Asia, Northern Europe, Western Europe, and the Eastern Mediterranean. The visualization emphasizes the substantial geographic disparities in the burden of childhood cancers worldwide.
(TIF)

S5 Fig Global Distribution of Age-standardized Mortality Rates for Childhood Cancers Across 204 Countries and Territories, Both Sexes Combined, 2021. This map illustrates the global distribution of age-standardized mortality rates (ASMR) for childhood cancers in 2021, categorized into five groups based on ASMR values (per 100,000 population): < 1.99, 1.99 to <2.58, 2.58 to <3.36, 3.36 to <4.08, and 4.08 to <5.12. The map highlights regions with the highest and lowest mortality rates, revealing significant geographic disparities in the burden of childhood cancers. Notable subregions include the Caribbean and Central America, Persian Gulf, Balkan Peninsula, Southeast Asia, Northern Europe, Western Europe, and Eastern Mediterranean.
(TIF)

S6 Fig Global Distribution of Age-standardized DALYs Rates for Childhood Cancers Across 204 Countries and Territories, Both Sexes Combined, 2021. This map illustrates the global distribution of age-standardized disability-adjusted life years (DALYs) rates for childhood cancers in 2021, categorized into five groups based on DALYs values (per 100,000 population): < 170.96, 170.96 to <220.85, 220.85 to <285.11, 285.11 to <340.45, and 340.45 to <434. The map highlights regions with the highest and lowest DALYs rates, revealing significant geographic disparities in the burden of childhood cancers. Notable subregions include the Caribbean and Central America, Persian Gulf, Balkan Peninsula, Southeast Asia, Northern Europe, Western Europe, and the Eastern Mediterranean.
(TIF)

S7 Fig Age-specific Burden of Childhood Cancers (Ages 0–14) and Distribution of Cancer Types Globally in 2021. Panels A, B, and C display the age-specific incidence rate, mortality rate, and disability-adjusted life years (DALY) rate for childhood cancers across three age groups (<5 years, 5–9 years, and 10–14 years) globally in 2021, stratified by sex. Panels D, E, and F present the absolute numbers of new cases, deaths, and DALYs for the same age groups, disaggregated by cancer type. Panels G, H, and I illustrate the proportional contributions of each cancer type to the total incidence rate, mortality rate, and DALY rate, respectively, using stacked bar charts. Data are reported for both sexes combined. DALYs = disability-adjusted life years; Mil. = million.
(TIF)

S8 Fig Relationship Between Age-standardized Incidence Rates and Average Annual Percent Change of Childhood Cancers Across SDI Categories and GBD Regions, Both Sexes Combined, 2021. This scatter plot shows the relationship between ASIR and AAPC of childhood cancers in 2021. Each point represents a global region or SDI quintile, color-coded according to ASIR and AAPC categories. Red dashed lines divide the data into nine categories based on terciles of ASIR (ASIR 1/3 = 113.51, ASIR 2/3 = 187.21) and AAPC (AAPC 1/3 = −0.13, AAPC 2/3 = −0.05). Regions in the top-right quadrant (e.g., High_High) exhibit high ASIR and high AAPC, indicating a high incidence of childhood cancers and an increasing trend. Regions in the bottom-left quadrant (e.g., Low_Low) have low ASIR and low AAPC, reflecting low incidence and slower changes over time. AAPC = average annual percent change; ASIR = age-standardized incidence rate; SDI = Sociodemographic Index; GBD = Global Burden of Disease, Injuries, and Risk Factors Study.
(TIF)

S9 Fig Relationship Between Age-standardized Mortality Rates and Average Annual Percent Change for Childhood Cancers Across SDI Categories and GBD Regions, Both Sexes Combined, 2021. This scatter plot illustrates the relationship between ASMR and AAPC for childhood cancers across 21 GBD regions and five SDI levels in 2021. Each point represents a region or SDI level, categorized into nine combinations based on ASMR and AAPC terciles: Low_Low, Low_Medium, Low_High, Medium_Low, Medium_Medium, Medium_High, High_Low, High_Medium, and High_High. The red dashed lines divide the plot according to the 1/3 and 2/3 quantiles of ASMR and AAPC. Regions in the “High_High” category, such as Eastern Sub-Saharan Africa and the Caribbean, exhibit the highest mortality rates coupled with slow or minimal improvements in trends. In contrast, regions in the “Low_Low” category, including High-income North America and High-income Asia Pacific, show the lowest mortality rates and the fastest declines in AAPC. AAPC = average annual percent change; ASMR = age-standardized mortality rate; SDI = Sociodemographic Index; GBD = Global Burden of Disease, Injuries, and Risk Factors Study.
(TIF)

S10 Fig Health Inequality Regression Curves and Concentration Curves for Acute Lymphoid Leukemia in Childhood Cancer. Panels A and B show the health inequality regression curves and concentration curves for the incidence of acute lymphoid leukemia (ALL) in childhood cancer, respectively. Panels C and D display the health inequality regression curves and concentration curves for mortality due to ALL in childhood cancer, respectively. Panels E and F illustrate the health inequality regression curves and concentration curves for DALYs due to ALL, respectively. DALYs = disability-adjusted life years.
(TIF)

S11 Fig Health Inequality Regression Curves and Concentration Curves for Brain and Central Nervous System Cancers in Childhood Cancer. Panels A and B show the health inequality regression curves and concentration curves for the incidence of brain and central nervous system cancers, respectively. Panels C and D display the health inequality regression curves and concentration curves for childhood cancer mortality due to brain and central nervous system cancers, respectively. Panels E and F illustrate the health inequality regression curves and concentration curves for DALYs due to brain and central nervous system cancers, respectively. DALYs = disability-adjusted life years.
(TIF)

S12 Fig Health Inequality Regression Curves and Concentration Curves for Non-Hodgkin Lymphoma in Childhood Cancer. Panels A and B show the health inequality regression curves and concentration curves for the incidence of Non-Hodgkin lymphoma in childhood cancer, respectively. Panels C and D display the health inequality regression curves and concentration curves for mortality due to Non-Hodgkin lymphoma in childhood cancer, respectively. Panels E and F illustrate the health inequality regression curves and concentration curves for DALYs due to Non-Hodgkin lymphoma in childhood cancer, respectively. DALYs = disability-adjusted life years.
(TIF)

S1 Table Childhood cancer burden in 1990 and 2021 and their temporal trends from 1990 to 2021. (DOCX)

S2 Table Incidence and Mortality of Childhood Cancer in 1990 and 2021. (DOCX)

S3 Table Childhood Cancer Burden by Different Countries and Territories from 1990 to 2021. (DOCX)

S4 Table Burden of Different Childhood Cancers from 1990 to 2021. (DOCX)

S5 Table Slope index of inequality and concentration index in global incidence, deaths, and DALYs. of childhood cancer and its subtypes in 2021. (DOCX)
