# Supplementary figures and images for: Childhood cancer burden and health inequality: A systematic analysis from the global burden of diseases study 2021
Source: PLoS One. 2026 Jan 27;21(1):e0341303. doi: 10.1371/journal.pone.0341303 (PMC12843563; doi:10.1371/journal.pone.0341303)

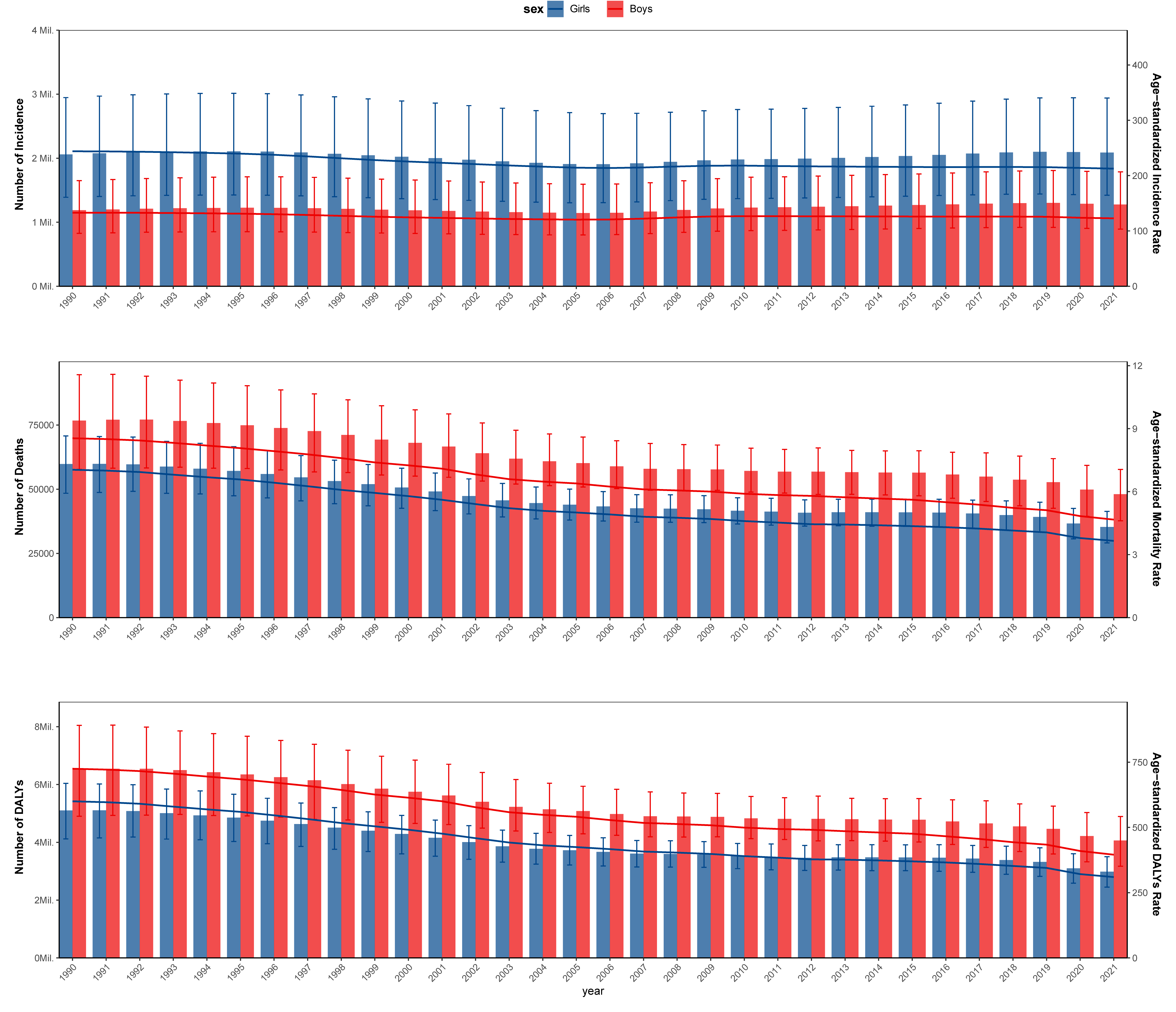

Supplement: S1 Fig — This figure presents the trends in the global burden of childhood cancers (ages 0–14) from 1990 to 2021, stratified by sex. The top panel shows the number of incidences and age-standardized incidence rates (ASIR) (per 100,000 population). The middle panel displays the number of deaths and age-standardized mortality rates (ASMR), while the bottom panel presents the number of disability-adjusted life years (DALYs) and age-standardized DALY rates. (TIF) [file pone.0341303.s001.tif]

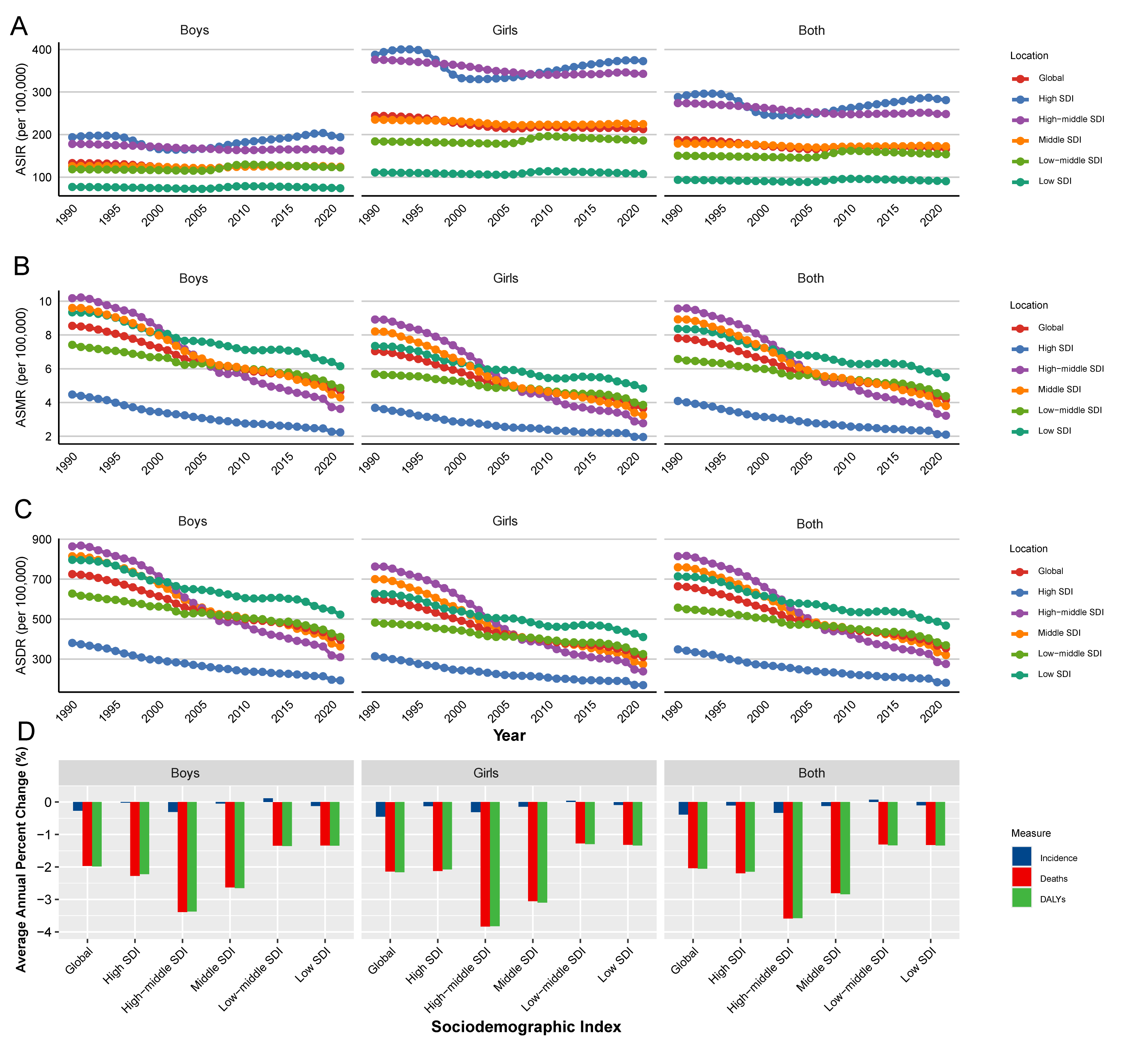

Supplement: S2 Fig — ASIR = age-standardised incidence rates. ASMR = age-standardised mortality rates. ASDR = age-standardised disability-adjusted life years rates. DALYs = disability-adjusted life years. SDI = Sociodemographic Index. (TIF) [file pone.0341303.s002.tif]

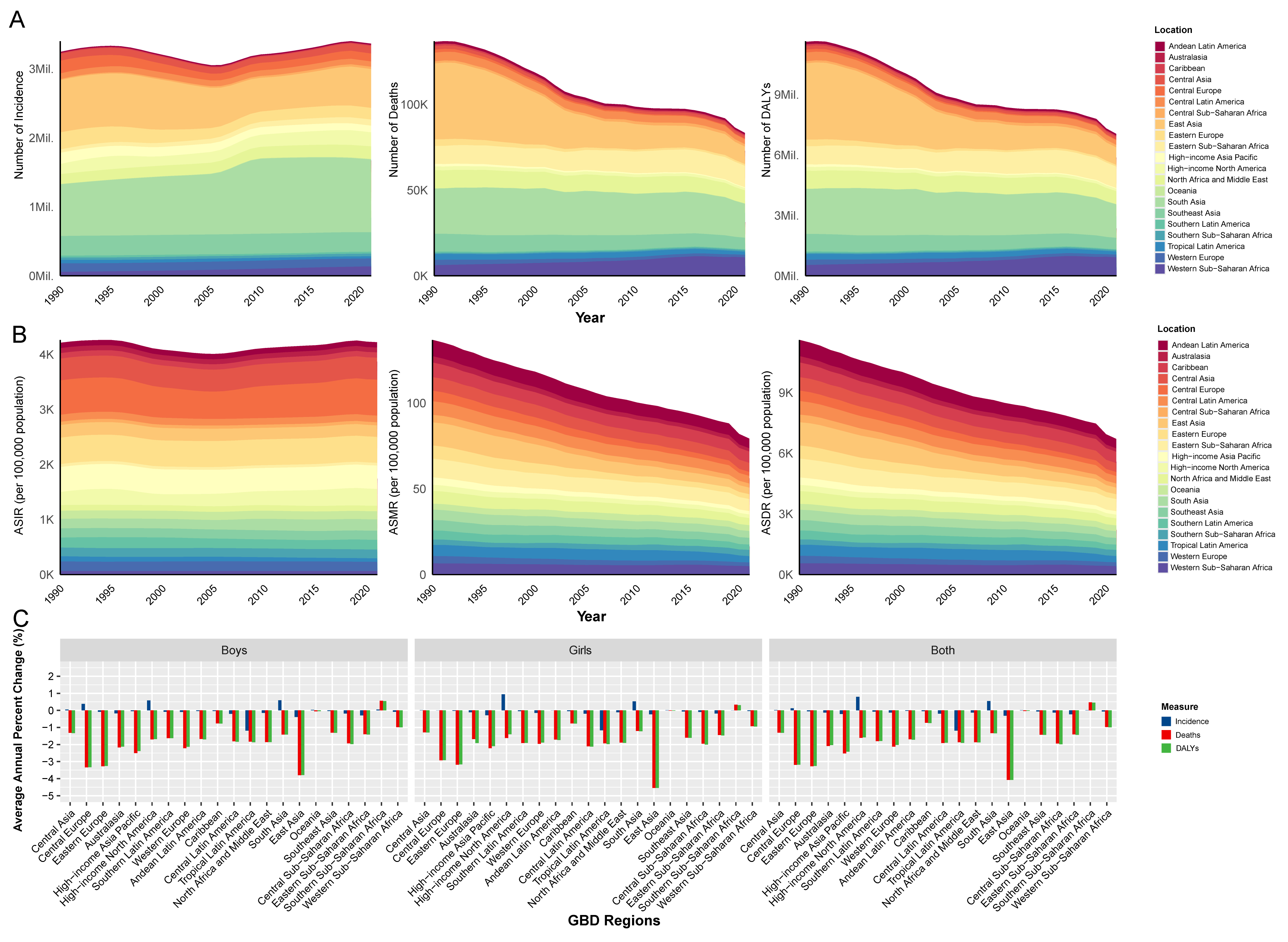

Supplement: S3 Fig — Panel A presents the total number of incident cases, deaths, and DALYs due to childhood cancers (ages 0–14) across 21 GBD regions from 1990 to 2021. Panel B shows the trends in ASIR, ASMR, and ASDR over the same period. Panel C displays the average annual percentage change for incidence, mortality, and DALYs, stratified by sex and GBD regions. ASIR = age-standardized incidence rates; ASMR = age-standardized mortality rates; ASDR = age-standardized disability-adjusted life years rates; DALYs = disability-adjusted life years; GBD = Global Burden of Disease, Injuries, and Risk Factors Study. Mil. = million; K = thousand. (TIF) [file pone.0341303.s003.tif]

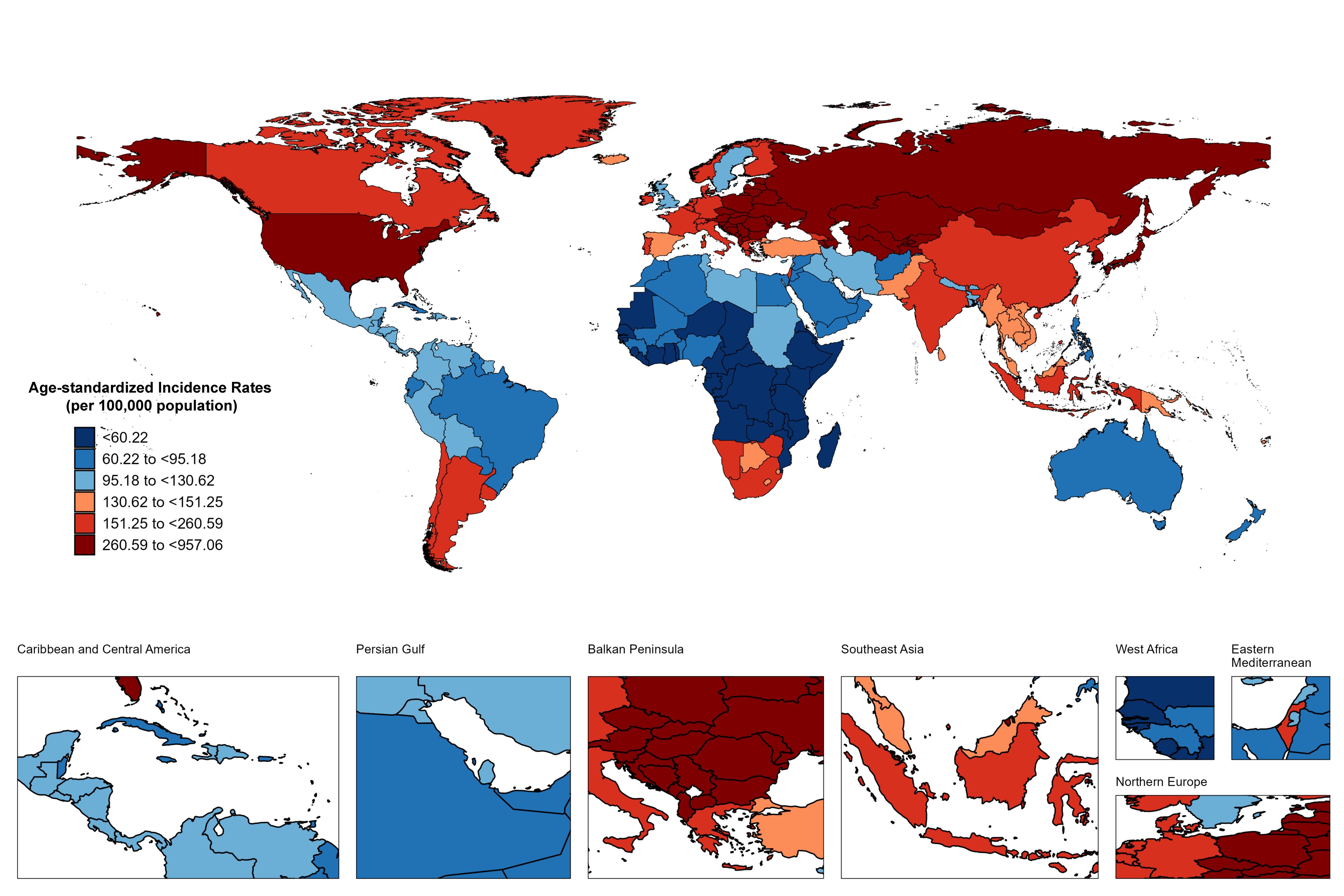

Supplement: S4 Fig — This map illustrates the global distribution of age-standardized incidence rates (ASIR) for childhood cancers in 2021, categorized into four quartiles based on ASIR values (per 100,000 population): < 60.22, 60.22 to <95.18, 95.18 to <130.62, and 130.62 to <151.25. The map highlights notable subregions, including the Caribbean and Central America, Persian Gulf, Balkan Peninsula, Southeast Asia, Northern Europe, Western Europe, and the Eastern Mediterranean. The visualization emphasizes the substantial geographic disparities in the burden of childhood cancers worldwide. (TIF) [file pone.0341303.s004.tif]

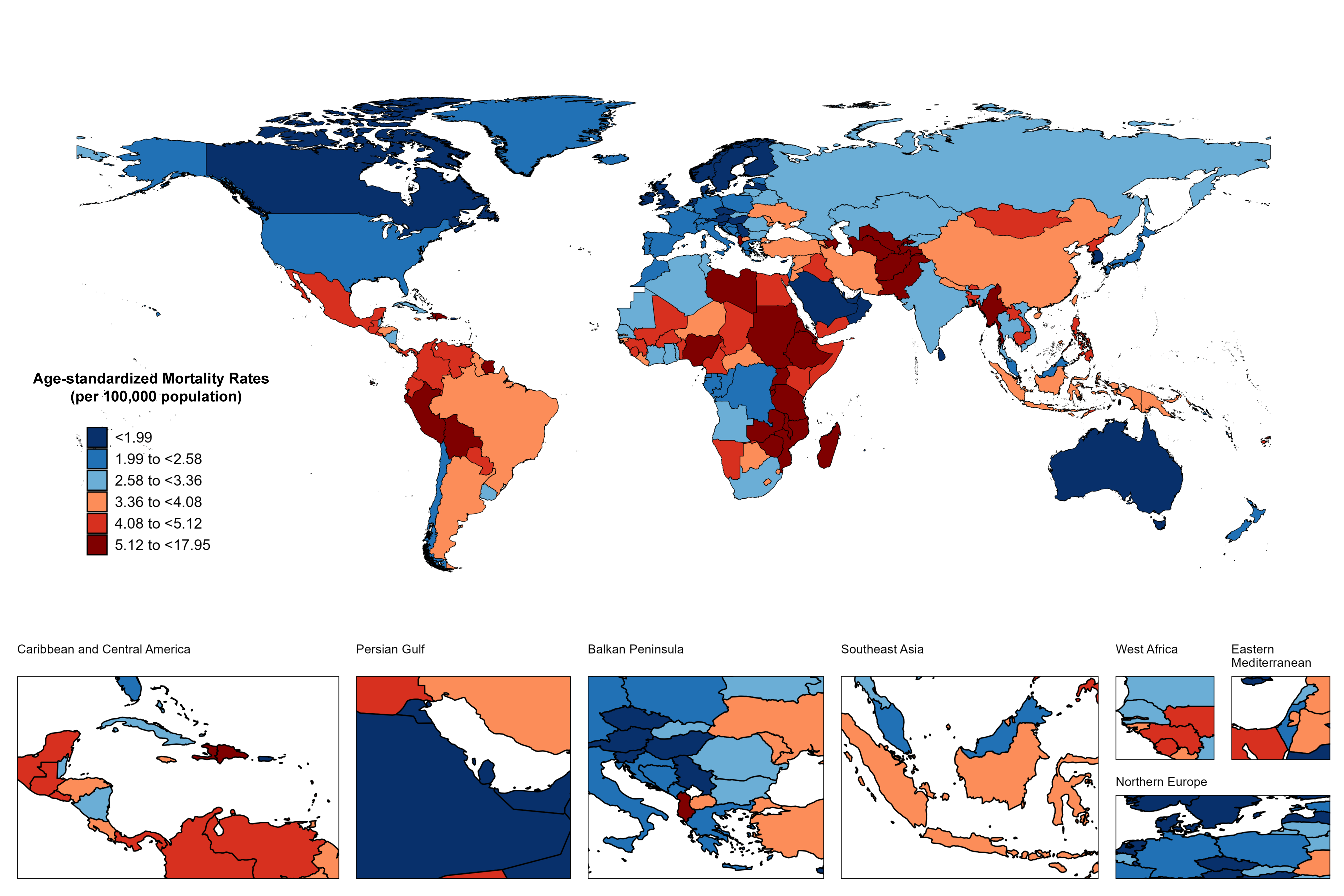

Supplement: S5 Fig — This map illustrates the global distribution of age-standardized mortality rates (ASMR) for childhood cancers in 2021, categorized into five groups based on ASMR values (per 100,000 population): < 1.99, 1.99 to <2.58, 2.58 to <3.36, 3.36 to <4.08, and 4.08 to <5.12. The map highlights regions with the highest and lowest mortality rates, revealing significant geographic disparities in the burden of childhood cancers. Notable subregions include the Caribbean and Central America, Persian Gulf, Balkan Peninsula, Southeast Asia, Northern Europe, Western Europe, and Eastern Mediterranean. (TIF) [file pone.0341303.s005.tif]

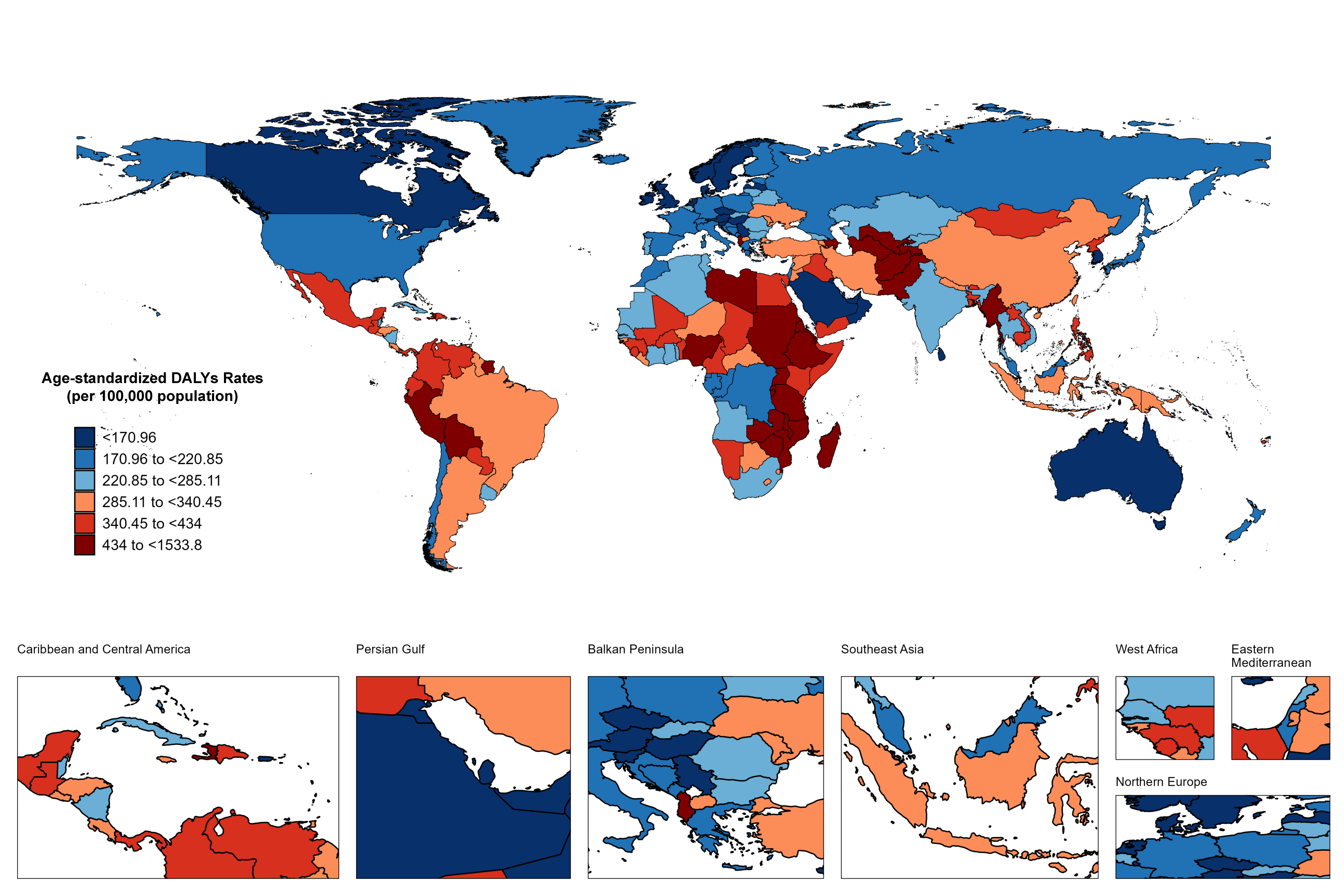

Supplement: S6 Fig — This map illustrates the global distribution of age-standardized disability-adjusted life years (DALYs) rates for childhood cancers in 2021, categorized into five groups based on DALYs values (per 100,000 population): < 170.96, 170.96 to <220.85, 220.85 to <285.11, 285.11 to <340.45, and 340.45 to <434. The map highlights regions with the highest and lowest DALYs rates, revealing significant geographic disparities in the burden of childhood cancers. Notable subregions include the Caribbean and Central America, Persian Gulf, Balkan Peninsula, Southeast Asia, Northern Europe, Western Europe, and the Eastern Mediterranean. (TIF) [file pone.0341303.s006.tif]

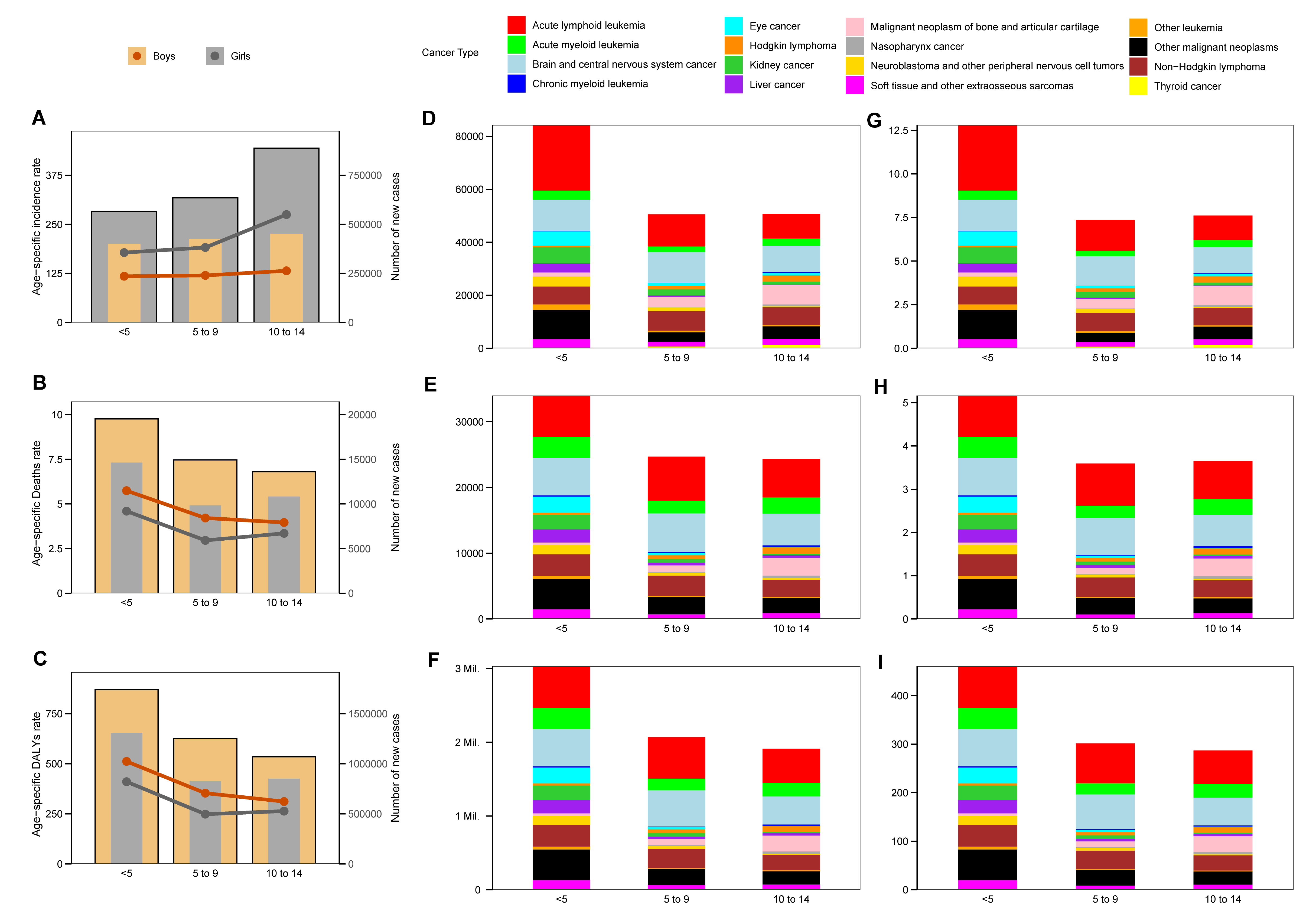

Supplement: S7 Fig — Panels A, B, and C display the age-specific incidence rate, mortality rate, and disability-adjusted life years (DALY) rate for childhood cancers across three age groups (<5 years, 5–9 years, and 10–14 years) globally in 2021, stratified by sex. Panels D, E, and F present the absolute numbers of new cases, deaths, and DALYs for the same age groups, disaggregated by cancer type. Panels G, H, and I illustrate the proportional contributions of each cancer type to the total incidence rate, mortality rate, and DALY rate, respectively, using stacked bar charts. Data are reported for both sexes combined. DALYs = disability-adjusted life years; Mil. = million. (TIF) [file pone.0341303.s007.tif]

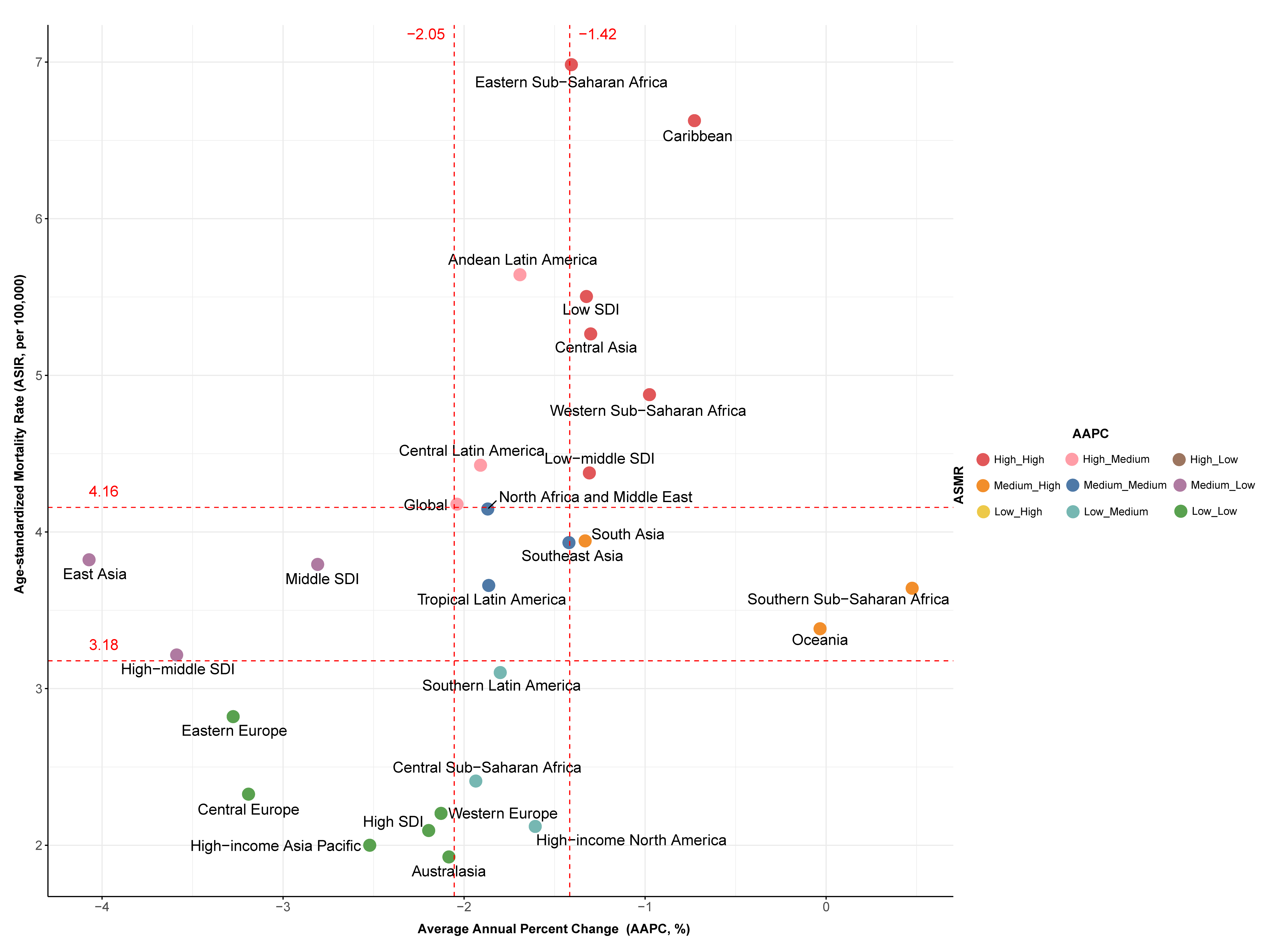

Supplement: S8 Fig — This scatter plot shows the relationship between ASIR and AAPC of childhood cancers in 2021. Each point represents a global region or SDI quintile, color-coded according to ASIR and AAPC categories. Red dashed lines divide the data into nine categories based on terciles of ASIR (ASIR 1/3 = 113.51, ASIR 2/3 = 187.21) and AAPC (AAPC 1/3 = −0.13, AAPC 2/3 = −0.05). Regions in the top-right quadrant (e.g., High_High) exhibit high ASIR and high AAPC, indicating a high incidence of childhood cancers and an increasing trend. Regions in the bottom-left quadrant (e.g., Low_Low) have low ASIR and low AAPC, reflecting low incidence and slower changes over time. AAPC = average annual percent change; ASIR = age-standardized incidence rate; SDI = Sociodemographic Index; GBD = Global Burden of Disease, Injuries, and Risk Factors Study. (TIF) [file pone.0341303.s008.tif]

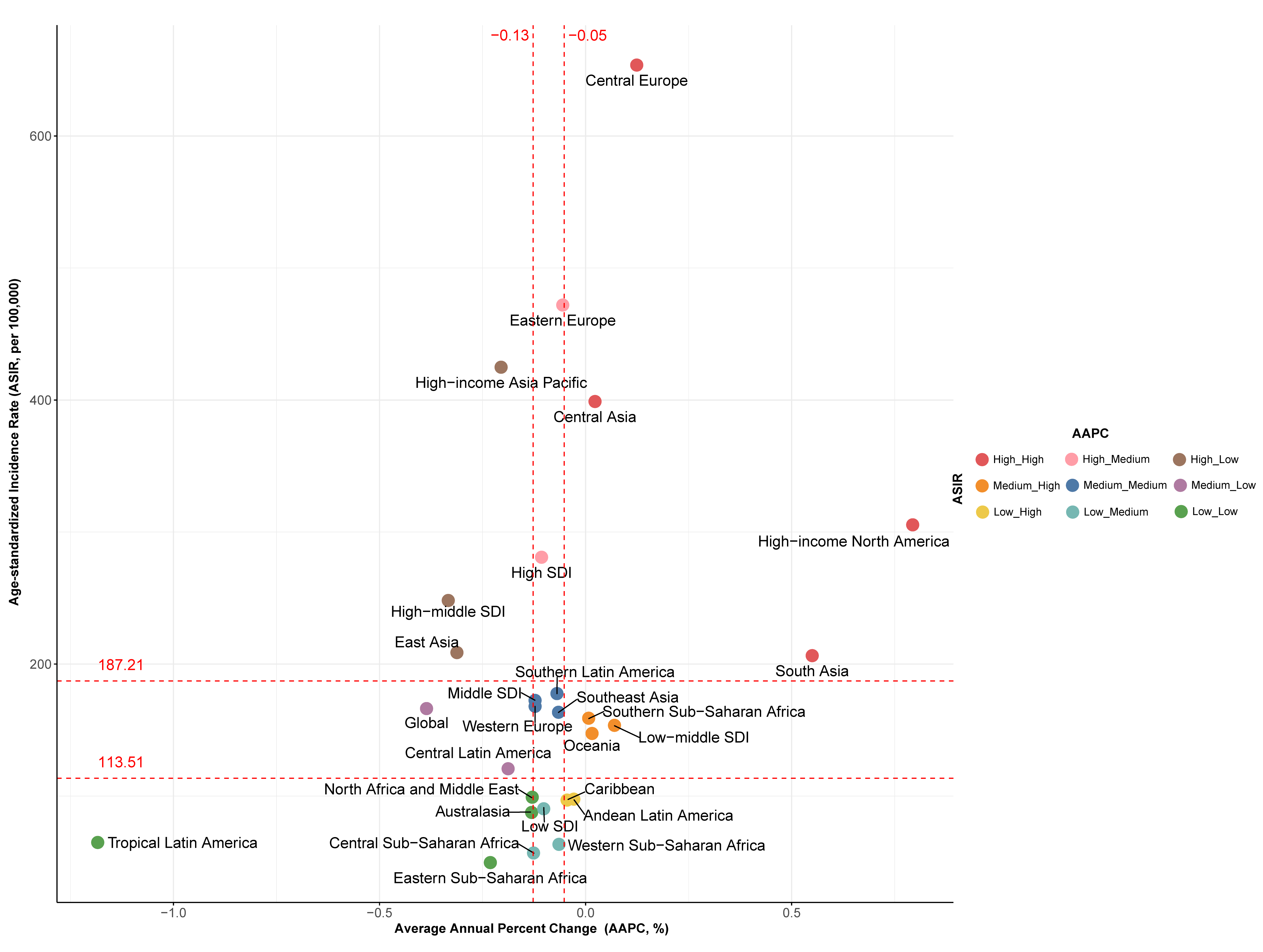

Supplement: S9 Fig — This scatter plot illustrates the relationship between ASMR and AAPC for childhood cancers across 21 GBD regions and five SDI levels in 2021. Each point represents a region or SDI level, categorized into nine combinations based on ASMR and AAPC terciles: Low_Low, Low_Medium, Low_High, Medium_Low, Medium_Medium, Medium_High, High_Low, High_Medium, and High_High. The red dashed lines divide the plot according to the 1/3 and 2/3 quantiles of ASMR and AAPC. Regions in the “High_High” category, such as Eastern Sub-Saharan Africa and the Caribbean, exhibit the highest mortality rates coupled with slow or minimal improvements in trends. In contrast, regions in the “Low_Low” category, including High-income North America and High-income Asia Pacific, show the lowest mortality rates and the fastest declines in AAPC. AAPC = average annual percent change; ASMR = age-standardized mortality rate; SDI = Sociodemographic Index; GBD = Global Burden of Disease, Injuries, and Risk Factors Study. (TIF) [file pone.0341303.s009.tif]

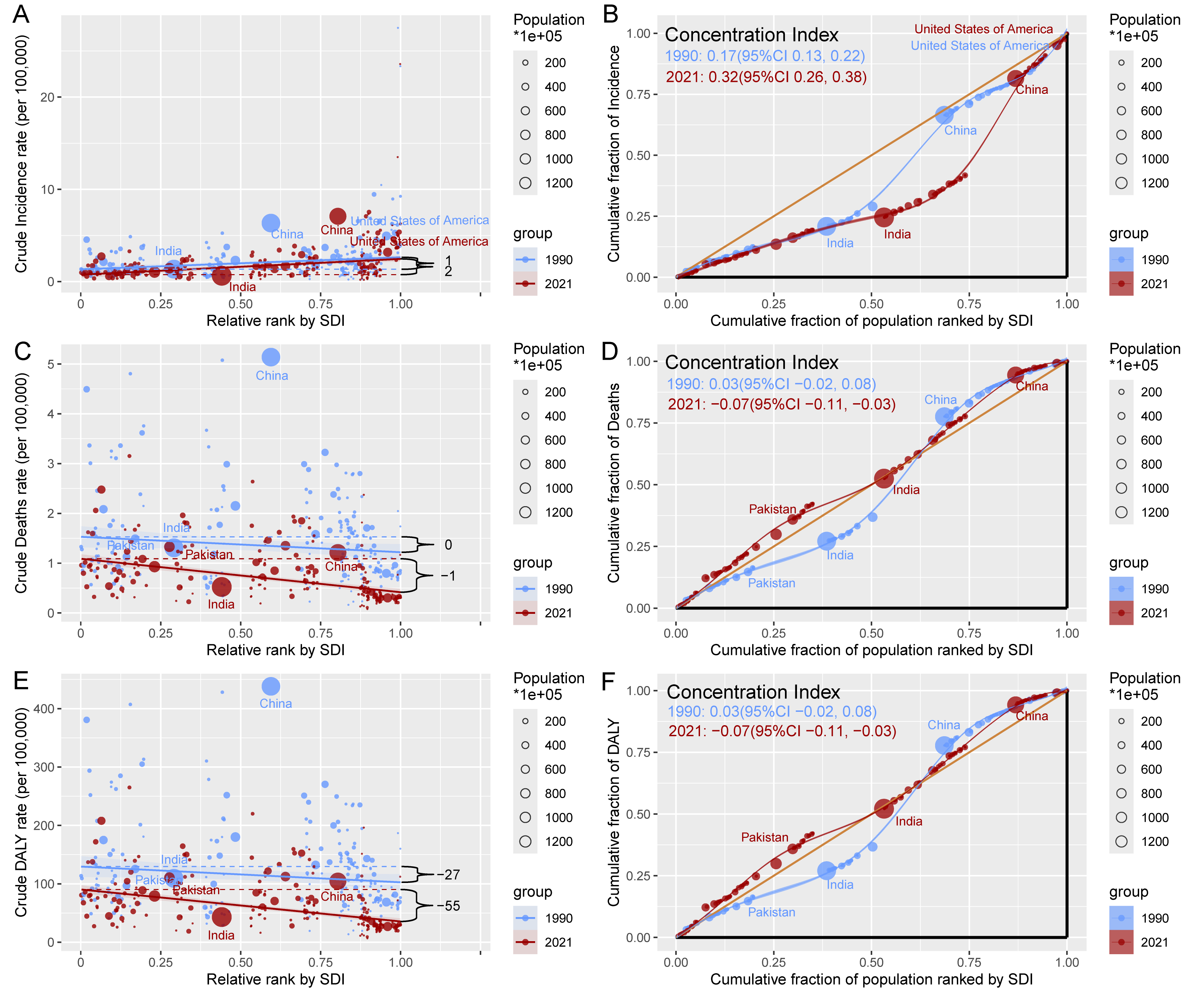

Supplement: S10 Fig — Panels A and B show the health inequality regression curves and concentration curves for the incidence of acute lymphoid leukemia (ALL) in childhood cancer, respectively. Panels C and D display the health inequality regression curves and concentration curves for mortality due to ALL in childhood cancer, respectively. Panels E and F illustrate the health inequality regression curves and concentration curves for DALYs due to ALL, respectively. DALYs = disability-adjusted life years. (TIF) [file pone.0341303.s010.tif]

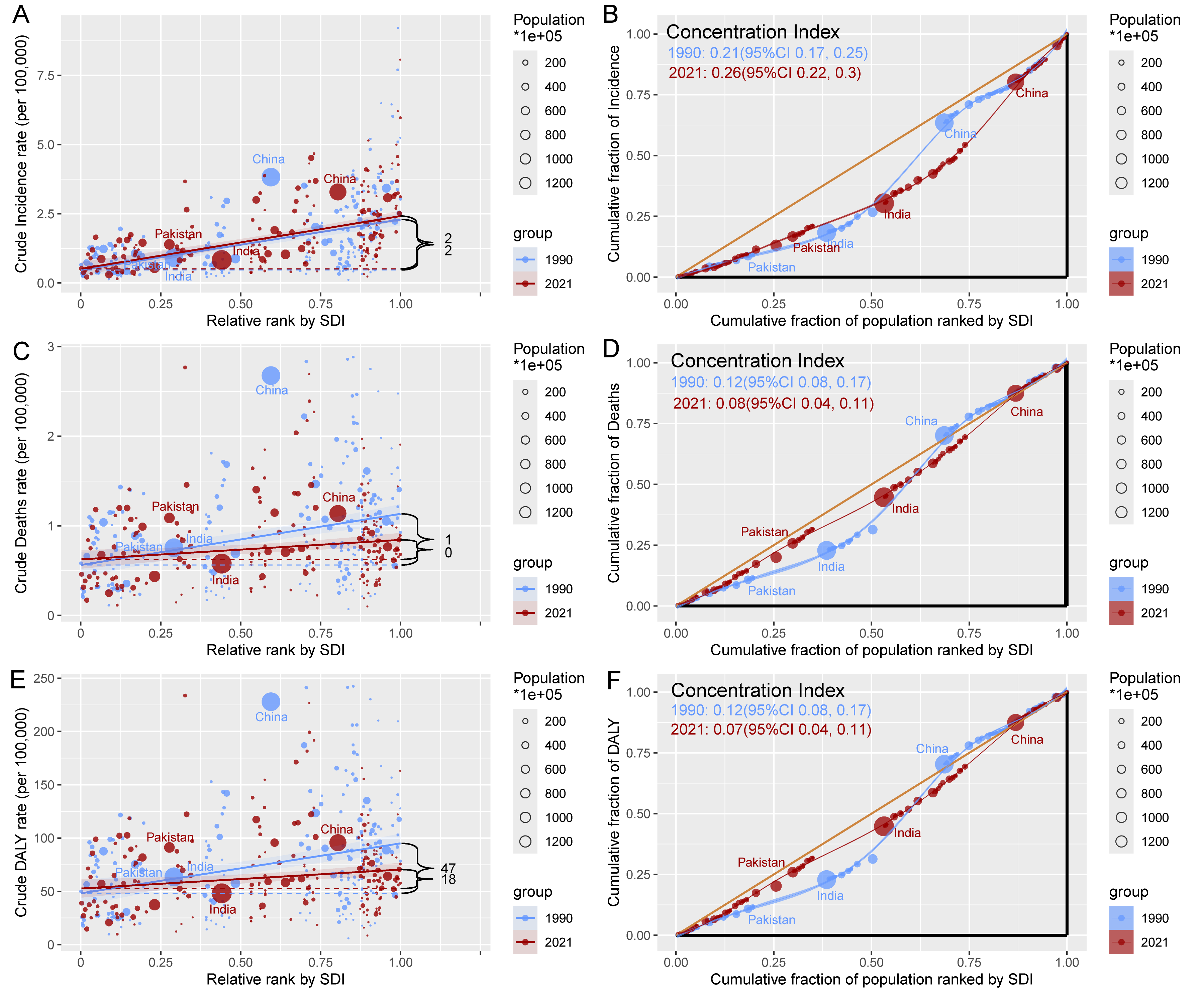

Supplement: S11 Fig — Panels A and B show the health inequality regression curves and concentration curves for the incidence of brain and central nervous system cancers, respectively. Panels C and D display the health inequality regression curves and concentration curves for childhood cancer mortality due to brain and central nervous system cancers, respectively. Panels E and F illustrate the health inequality regression curves and concentration curves for DALYs due to brain and central nervous system cancers, respectively. DALYs = disability-adjusted life years. (TIF) [file pone.0341303.s011.tif]

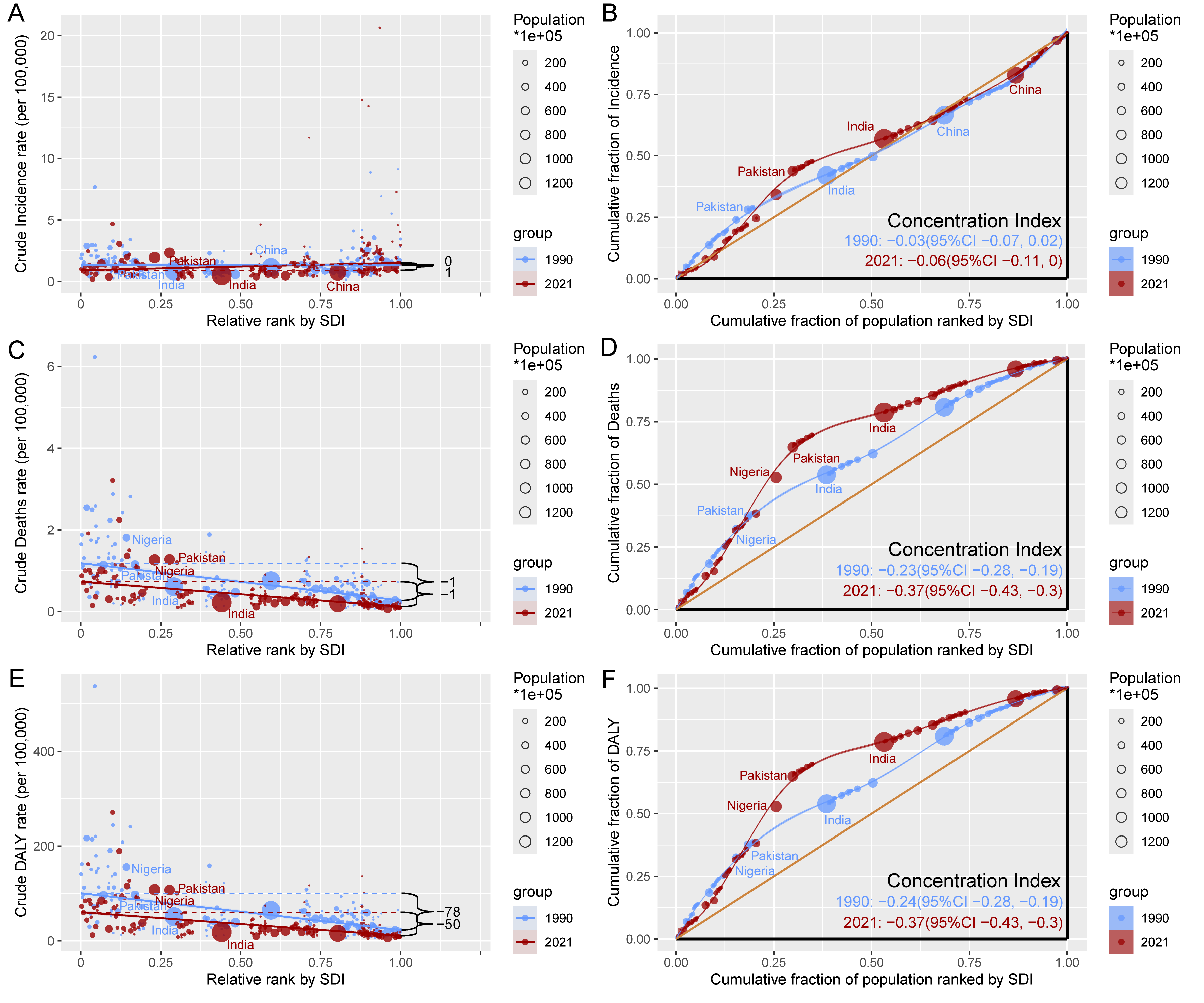

Supplement: S12 Fig — Panels A and B show the health inequality regression curves and concentration curves for the incidence of Non-Hodgkin lymphoma in childhood cancer, respectively. Panels C and D display the health inequality regression curves and concentration curves for mortality due to Non-Hodgkin lymphoma in childhood cancer, respectively. Panels E and F illustrate the health inequality regression curves and concentration curves for DALYs due to Non-Hodgkin lymphoma in childhood cancer, respectively. DALYs = disability-adjusted life years. (TIF) [file pone.0341303.s012.tif]
