# Supplementary material for: Childhood cancer burden and health inequality: A systematic analysis from the global burden of diseases study 2021
Source: PLoS One. 2026 Jan 27;21(1):e0341303. doi: 10.1371/journal.pone.0341303 (PMC12843563; doi:10.1371/journal.pone.0341303)
Supplement: S1 Table — (DOCX) [file pone.0341303.s013.docx]

**S1 Table. Childhood cancer burden in 1990 and 2021 and their temporal trends from 1990 to 2021.**

| **Characteristics** | **Absolute YLLs, 1990**  **(95% UI)** | **Absolute YLLs, 2021**  **(95% UI)** | **Percentage change, %**  **(1990-2021)** | **Absolute YLDs, 1990**  **(95% UI)** | **Absolute YLDs, 2021**  **(95% UI)** | **Percentage change, %**  **(1990-2021)** | **Absolute DALYs, 1990**  **(95% UI)** | **Absolute DALYs, 2021**  **(95% UI)** | **Percentage change, %**  **(1990-2021)** | **Age-standardised DALYs rate, 1990**  **(95% UI)** | **Age-standardised DALYs rate, 2021**  **(95% UI)** | **AAPC, %**  **(95% CI)** | ***P*** |
| --- | --- | --- | --- | --- | --- | --- | --- | --- | --- | --- | --- | --- | --- |
| Global | 11517191 (9580097 to 13631531) | 6943792 (5693156 to 8162284) | -39.71 | 100216 (69444 to 138795) | 100756 (69192 to 144128) | 0.54 | 11617407 (9665848 to 13751200) | 7044548 (5773433 to 8279675) | -39.36 | 664.31 (552.99 to 785.90) | 354.06 (289.08 to 417.49) | -2.05 (-2.28 to -1.82) | 0 |
| Boys | 6460271 (4848556 to 7984461) | 4004297 (3128956 to 4819120) | -38.02 | 55824 (38212 to 77862) | 57577 (38612 to 82642) | 3.14 | 6516095 (4901706 to 8046401) | 4061875 (3171115 to 4892859) | 37.66 | 725.01 (545.90 to 894.62) | 395.55 (307.57 to 477.68) | -1.98 (-2.14 to -1.83) | 0 |
| Girls | 5056920 (4083511 to 5987994) | 2939495 (2416010 to 3456255) | -41.87 | 44392 (30385 to 61505) | 43178 (29697 to 62717) | -2.73 | 5101313 (4118630 to 6040017) | 2982673 (2450184 to 3502234) | -41.53 | 600.20 (484.87 to 710.38) | 309.80 (253.75 to 364.91) | -2.16 (-2.34 to -1.98) | 0 |
| **SDI level** | | | | | | | | | | | | | |
| High SDI | 629139 (605344 to 653346) | 298874 (279133 to 318810) | -52.49 | 15752 (11044 to 21659) | 13613 (9649 to 18976) | -13.58 | 644891 (619801 to 670250) | 312487 (291019 to 334375) | -51.54 | 348.35 (334.70 to 362.23) | 181.35 (168.53 to 194.46) | -2.15 (-2.34 to -1.95) | 0 |
| High-middle SDI | 2185275 (1846826 to 2515787) | 612255 (508717 to 707622) | -71.98 | 21159 (14919 to 28970) | 18600 (11959 to 28131) | -12.1 | 2206434 (1866145 to 2540290) | 630854 (523018 to 732730) | -71.41 | 814.73 (688.34 to 939.12) | 275.24 (226.83 to 321.58) | -3.58 (-3.91 to -3.24) | 0 |
| Middle SDI | 4337494 (3551042 to 5106443) | 1774814 (1461408 to 2095840) | -59.08 | 32448 (22762 to 45083) | 29378 (19846 to 42699) | -9.46 | 4369941 (3579913 to 5141777) | 1804191 (1486179 to 2129425) | -58.71 | 759.08 (621.70 to 893.42) | 320.02 (262.38 to 379.67) | -2.84 (-3.04 to -2.64) | 0 |
| Low-middle SDI | 2646992 (1960954 to 3391253) | 2100130 (1743754 to 2481583) | -20.66 | 18973 (12409 to 27171) | 21190 (14534 to 29715) | 11.68 | 2665966 (1975392 to 3416405) | 2121320 (1761478 to 2503094) | -20.43 | 557.07 (414.16 to 712.45) | 368.94 (305.58 to 436.13) | -1.33 (-1.57 to -1.09) | 0 |
| Low SDI | 1710002 (1203779 to 2262183) | 2152017 (1571368 to 2714484) | 25.85 | 11807 (7495 to 17171) | 17906 (11451 to 26385) | 51.65 | 1721809 (1213058 to 2279335) | 2169923 (1584086 to 2739239) | 26.03 | 713.83 (505.17 to 943.11) | 467.41 (342.00 to 589.20) | -1.34 (-1.54 to -1.14) | 0 |
| **GBD regions** | | | | | | | | | | | | | |
| Central Asia | 167613 (149467 to 189071) | 121753 (102046 to 145653) | -27.36 | 1197 (830 to 1619) | 1152 (794 to 1637) | -3.75 | 168810 (150597 to 190365) | 122905 (103035 to 147063) | -27.19 | 664.21 (594.27 to 746.58) | 442.29 (371.33 to 528.35) | -1.31 (-1.56 to -1.06) | 0 |
| Central Europe | 148698 (139153 to 158601) | 34006 (30485 to 38099) | -77.13 | 1630 (1148 to 2234) | 779 (541 to 1108) | -52.18 | 150328 (140592 to 160256) | 34785 (31191 to 38922) | -76.86 | 520.41 (485.99 to 555.87) | 197.22 (176.02 to 221.74) | -3.18 (-3.67 to -2.69) | 0 |
| Eastern Europe | 328193 (311723 to 345429) | 81776 (74504 to 89322) | -75.08 | 4084 (2874 to 5566) | 1753 (1229 to 2454) | -57.08 | 332277 (315832 to 349660) | 83529 (76073 to 91315) | -74.86 | 648.32 (615.89 to 682.58) | 240.11 (217.37 to 264.19) | -3.26 (-4.06 to -2.45) | 0 |
| Australasia | 13952 (13087 to 14901) | 9133 (8034 to 10330) | -34.54 | 321 (222 to 449) | 381 (253 to 560) | 18.53 | 14274 (13393 to 15262) | 9513 (8364 to 10764) | -33.35 | 312.44 (293.09 to 334.10) | 166.08 (145.52 to 188.41) | -2.03 (-2.58 to -1.48) | 0 |
| High-income Asia Pacific | 125704 (113055 to 138488) | 37068 (33413 to 40150) | -70.51 | 3415 (2348 to 4770) | 2257 (1551 to 3191) | -33.9 | 129119 (116298 to 141916) | 39326 (35429 to 42790) | -69.54 | 367.05 (330.28 to 404.12) | 175.72 (157.53 to 192.08) | -2.41 (-2.76 to -2.07) | 0 |
| High-income North America | 182463 (178842 to 186213) | 115089 (106628 to 124151) | -36.92 | 6016 (4174 to 8244) | 5035 (3521 to 6999) | -16.32 | 188479 (184259 to 192899) | 120124 (111371 to 129583) | -36.27 | 305.65 (298.81 to 312.81) | 183.25 (169.47 to 198.12) | -1.58 (-1.89 to -1.28) | 0 |
| Southern Latin America | 65384 (60799 to 69959) | 37368 (32677 to 42818) | -42.85 | 688 (487 to 927) | 691 (477 to 975) | 0.39 | 66072 (61478 to 70647) | 38059 (33295 to 43547) | -42.4 | 443.04 (412.22 to 473.77) | 260.65 (226.81 to 299.99) | -1.80 (-2.33 to -1.26) | 0 |
| Western Europe | 252482 (246119 to 258968) | 124063 (115466 to 132978) | -50.86 | 6880 (4777 to 9394) | 6216 (4379 to 8664) | -9.65 | 259363 (252583 to 266357) | 130280 (121192 to 140160) | -49.77 | 367.36 (357.59 to 377.42) | 191.68 (177.70 to 206.82) | -2.02 (-2.13 to -1.91) | 0 |
| Andean Latin America | 118958 (98840 to 146576) | 84385 (64599 to 107303) | -29.06 | 858 (578 to 1196) | 1038 (666 to 1564) | 20.99 | 119815 (99568 to 147614) | 85423 (65401 to 108630) | -28.7 | 803.98 (668.53 to 989.83) | 472.11 (360.90 to 601.08) | -1.72 (-2.11 to -1.33) | 0 |
| Caribbean | 82887 (59576 to 107607) | 63309 (43490 to 85930) | -23.62 | 698 (449 to 973) | 636 (411 to 914) | -8.83 | 83584 (60127 to 108363) | 63946 (44010 to 86715) | -23.5 | 726.62 (524.69 to 940.56) | 560.72 (384.08 to 762.28) | -0.74 (-0.89 to -0.59) | 0 |
| Central Latin America | 428180 (400990 to 461456) | 232812 (196024 to 281022) | -45.63 | 3653 (2571 to 4996) | 3361 (2313 to 4830) | -7.98 | 431833 (404407 to 465492) | 236173 (198828 to 284824) | -45.31 | 669.08 (626.84 to 720.90) | 370.70 (310.11 to 450.28) | -1.89 (-2.17 to -1.62) | 0 |
| Tropical Latin America | 284996 (251107 to 318545) | 151929 (122073 to 179142) | -46.69 | 2189 (1545 to 2944) | 1828 (1282 to 2620) | -16.48 | 287185 (253263 to 320828) | 153757 (123598 to 181637) | -46.46 | 546.57 (481.28 to 612.00) | 306.37 (245.86 to 362.36) | -1.90 (-2.16 to -1.65) | 0 |
| North Africa and Middle East | 889291 (675614 to 1123923) | 629678 (500657 to 752500) | -29.19 | 7517 (4940 to 10718) | 9369 (6529 to 13246) | 24.64 | 896808 (681881 to 1134235) | 639047 (508805 to 762205) | -28.74 | 633.45 (482.91 to 799.47) | 349.12 (277.60 to 416.90) | -1.88 (-1.97 to -1.79) | 0 |
| South Asia | 2222027 (1565815 to 2956676) | 1643431 (1357713 to 1980451) | -26.04 | 16560 (10696 to 24373) | 17603 (11938 to 25027) | 6.3 | 2238587 (1577807 to 2978510) | 1661035 (1373472 to 2003420) | -25.8 | 511.64 (361.69 to 679.56) | 332.32 (273.66 to 402.93) | -1.34 (-1.58 to -1.10) | 0 |
| East Asia | 3756946 (2919742 to 4579930) | 843971 (654159 to 1058349) | -77.54 | 27268 (18773 to 38264) | 24286 (14501 to 37751) | -10.93 | 3784214 (2942971 to 4611767) | 868257 (670850 to 1094734) | -77.06 | 1148.01 (892.72 to 1399.06) | 327.51 (251.32 to 416.07) | -4.07 (-4.40 to -3.75) | 0 |
| Oceania | 7771 (4689 to 11403) | 14518 (9261 to 21730) | 86.83 | 60 (35 to 92) | 132 (79 to 209) | 118.34 | 7831 (4734 to 11489) | 14650 (9359 to 21914) | 87.07 | 289.80 (174.85 to 424.17) | 285.02 (181.92 to 424.93) | -0.03 (-0.46 to 0.40) | 0.893 |
| Southeast Asia | 866282 (590747 to 1181512) | 560461 (447337 to 679222) | -35.3 | 5922 (3586 to 8724) | 5692 (3873 to 8009) | -3.89 | 872205 (595040 to 1190575) | 566153 (451496 to 685714) | -35.09 | 512.99 (348.83 to 701.85) | 329.61 (262.04 to 400.65) | -1.43 (-1.58 to -1.28) | 0 |
| Central Sub-Saharan Africa | 101509 (59675 to 143472) | 118629 (87249 to 159036) | 16.87 | 687 (362 to 1052) | 899 (575 to 1297) | 30.77 | 102196 (60187 to 144400) | 119527 (87945 to 160153) | 16.96 | 376.13 (226.38 to 528.11) | 202.77 (149.34 to 271.38) | -1.98 (-2.08 to -1.88) | 0 |
| Eastern Sub-Saharan Africa | 892096 (640346 to 1165029) | 1057511 (762195 to 1393540) | 18.54 | 6121 (3880 to 8841) | 8767 (5522 to 13316) | 43.24 | 898217 (644630 to 1172351) | 1066278 (768815 to 1405325) | 18.71 | 933.32 (672.22 to 1216.03) | 593.51 (428.67 to 781.28) | -1.43 (-1.57 to -1.29) | 0 |
| Southern Sub-Saharan Africa | 54648 (42656 to 67067) | 72547 (57455 to 90005) | 32.75 | 513 (340 to 736) | 756 (509 to 1094) | 47.46 | 55161 (43072 to 67682) | 73304 (58045 to 90891) | 32.89 | 265.10 (207.32 to 324.89) | 305.69 (241.44 to 379.78) | 0.45 (-0.09 to 0.99) | 0.105 |
| Western Sub-Saharan Africa | 527111 (407188 to 649411) | 910353 (562152 to 1256053) | 72.71 | 3939 (2598 to 5623) | 8124 (4638 to 12940) | 106.23 | 531051 (410357 to 654298) | 918477 (567076 to 1265252) | 72.95 | 558.24 (429.86 to 689.20) | 416.66 (258.44 to 572.74) | -0.99 (-1.13 to -0.85) | 0 |

Estimates are for individuals aged 0-14 years. Absolute YLLs, YLDs, and DALYs represent the total childhood cancer (0-14 years, both sexes combined) values. Rates are reported per 100000 population. UI=uncertainty interval. CI=confidence interval. AAPC=average annual percent change. SDI=Socio-demographic Index. GBD=Global Burden of Disease.
