# Supplementary material for: Childhood cancer burden and health inequality: A systematic analysis from the global burden of diseases study 2021
Source: PLoS One. 2026 Jan 27;21(1):e0341303. doi: 10.1371/journal.pone.0341303 (PMC12843563; doi:10.1371/journal.pone.0341303)
Supplement: S2 Table — (DOCX) [file pone.0341303.s014.docx]

**S2 Table. Incidence and Mortality of Childhood Cancer in 1990 and 2021**

| **Characteristics** | **Absolute incidence, 1990** | **Absolute incidence, 2021** | **Percentage change, %**  **(1990-2021)** | **Age-standardised incidence rate, 1990 (95% UI)** | **Age-standardised incidence rate, 2021 (95% UI)** | **AAPC, %**  **(95% CI)** | ***P*** | **Absolute mortality, 1990** | **Absolute mortality, 2021** | **Percentage change, %**  **(1990-2021)** | **Age-standardised mortality rate, 1990 (95% UI)** | **Age-standardised mortality rate, 2021 (95% UI)** | **AAPC, %**  **(95% CI)** | ***P*** |
| --- | --- | --- | --- | --- | --- | --- | --- | --- | --- | --- | --- | --- | --- | --- |
| Global | 3246586 (2204141 to 4587296) | 3365505 (2312684 to 4724095) | 3.66 | 187.02 (126.83 to 264.50) | 166.28 (114.64 to 232.71) | -0.39 (-0.44 to -0.33) | 0 | 136529 (113730 to 161314) | 83340 (68566 to 97691) | -38.96 | 7.81 (6.51 to 9.23) | 4.18 (3.42 to 4.91) | -2.04 (-2.27 to -1.81) | 0 |
| Boys | 1187391  (825785 to 1649036) | 1277319 (891802 to 1786642) | 7.57 | 132.99 (92.37 to 184.94) | 122.78 (86.06 to 171.19) | -0.27 (-0.32 to -0.22) | 0 | 76710 (57754 to 94599) | 48056 (37684 to 57708) | -37.35 | 8.54 (6.44 to 10.53) | 4.67 (3.65 to 5.62) | -1.97 (-2.13 to -1.81) | 0 |
| Girls | 2059195 (1389618 to 2946991) | 2088187 (1421144 to 2939318) | 1.41 | 243.98 (164.53 to 349.43) | 212.63 (145.13 to 298.52) | -0.45 (-0.53 to -0.37) | 0 | 59819 (48414 to 70728) | 35284 (29074 to 41373) | -41.02 | 7.04 (5.70 to 8.32) | 3.65 (3.00 to 4.30) | -2.14 (-2.33 to -1.96) | 0 |
| **SDI level** | | | | | | | | | | | | | | |
| High SDI | 538105 (364966 to 762981) | 488261 (343027 to 673030) | -9.26 | 288.20 (196.02 to 407.60) | 280.90 (198.33 to 385.60) | -0.11 (-0.21 to 0.00) | 0.052 | 7586 (7301 to 7874) | 3628 (3393 to 3865) | -52.18 | 4.09 (3.93 to 4.25) | 2.09 (1.95 to 2.24) | -2.20 (-2.40 to -1.99) | 0 |
| High-middle SDI | 752184 (506255 to 1063685) | 577532 (401454 to 801268) | -23.22 | 273.92 (184.68 to 386.78) | 248.15 (173.62 to 342.32) | -0.33 (-0.38 to -0.29) | 0 | 25939 (21969 to 29798) | 7411 (6175 to 8542) | -71.43 | 9.56 (8.09 to 11.00) | 3.21 (2.66 to 3.73) | -3.59 (-3.92 to -3.25) | 0 |
| Middle SDI | 1033950 (704605 to 1460886) | 986376 (676520 to 1383467) | -4.60 | 178.92 (121.97 to 252.71) | 172.46 (118.91 to 240.79) | -0.12 (-0.14 to -0.10) | 0 | 51403 (42167 to 60376) | 21479 (17732 to 25292) | -58.22 | 8.93 (7.32 to 10.49) | 3.79 (3.12 to 4.49) | -2.81 (-3.01 to -2.61) | 0 |
| Low-middle SDI | 706033 (480478 to 1005444) | 895366 (598788 to 1284641) | 26.82 | 150.38 (102.05 to 214.73) | 153.57 (103.04 to 219.72) | 0.07 (0.05 to 0.09) | 0 | 31417 (23351 to 40174) | 25231 (21001 to 29758) | -19.69 | 6.58 (4.91 to 8.40) | 4.38 (3.63 to 5.17) | -1.31 (-1.53 to -1.09) | 0 |
| Low SDI | 212107 (147386 to 295679) | 414837 (284457 to 588666) | 95.58 | 93.63 (64.57 to 131.49) | 90.36 (61.85 to 128.40) | -0.10 (-0.15 to -0.05) | 0 | 20085 (14163 to 26550) | 25523 (18727 to 32102) | 27.07 | 8.37 (5.93 to 11.04) | 5.50 (4.05 to 6.91) | -1.32 (-1.52 to -1.12) | 0 |
| **GBD regions** | | | | | | | | | | | | | | |
| Central Asia | 98908 (67092 to 139436) | 110285 (74631 to 155966) | 11.5 | 396.01 (267.18 to 560.81) | 398.89 (269.39 to 565.02) | 0.02 (0.02 to 0.03) | 0 | 1993 (1780 to 2244) | 1461 (1227 to 1743) | -26.71 | 7.87 (7.05 to 8.83) | 5.26 (4.43 to 6.27) | -1.30 (-1.55 to -1.05) | 0 |
| Central Europe | 183913 (123559 to 260812) | 114964 (83535 to 153502) | -37.49 | 625.61 (423.56 to 881.29) | 653.75 (477.57 to 869.29) | 0.12 (0.03 to 0.22) | 0.008 | 1788 (1674 to 1905) | 412 (371 to 461) | -76.93 | 6.16 (5.76 to 6.57) | 2.33 (2.08 to 2.61) | -3.19 (-3.68 to -2.70) | 0 |
| Eastern Europe | 247311 (164294 to 350132) | 168532 (110013 to 241165) | -31.85 | 479.90 (319.61 to 678.10) | 471.97 (311.34 to 669.85) | -0.06 (-0.07 to -0.04) | 0 | 3935 (3741 to 4138) | 989 (902 to 1078) | -74.87 | 7.67 (7.28 to 8.07) | 2.82 (2.56 to 3.10) | -3.28 (-4.04 to -2.50) | 0 |
| Australasia | 4202 (2947 to 5807) | 5036 (3533 to 6949) | 19.85 | 91.38 (64.23 to 126.08) | 87.58 (61.74 to 120.37) | -0.13 (-0.23 to -0.03) | 0.012 | 168 (158 to 179) | 111 (98 to 125) | -34.09 | 3.67 (3.45 to 3.92) | 1.93 (1.69 to 2.18) | -2.08 (-2.65 to -1.51) | 0 |
| High-income Asia Pacific | 162260 (108127 to 236509) | 96261 (65995 to 136754) | -40.67 | 452.95 (304.53 to 654.35) | 424.86 (294.36 to 598.25) | -0.20 (-0.24 to -0.17) | 0 | 1531 (1377 to 1685) | 451 (408 to 488) | -70.51 | 4.32 (3.88 to 4.76) | 2.00 (1.80 to 2.17) | -2.52 (-2.86 to -2.18) | 0 |
| High-income North America | 146956 (100045 to 207476) | 203392 (141186 to 282871) | 38.4 | 238.09 (162.03 to 336.24) | 305.46 (212.84 to 423.65) | 0.79 (0.59 to 1.00) | 0 | 2197 (2154 to 2241) | 1398 (1297 to 1506) | -36.37 | 3.56 (3.49 to 3.63) | 2.12 (1.96 to 2.29) | -1.61 (-1.91 to -1.30) | 0 |
| Southern Latin America | 26937 (18276 to 38417) | 26064 (18921 to 35349) | -3.24 | 180.21 (122.33 to 256.83) | 177.64 (129.87 to 239.44) | -0.07 (-0.23 to 0.09) | 0.386 | 788 (733 to 842) | 456 (399 to 521) | -42.16 | 5.28 (4.91 to 5.65) | 3.10 (2.71 to 3.57) | -1.80 (-2.34 to -1.26) | 0 |
| Western Europe | 124089 (87088 to 171200) | 114886 (82713 to 154993) | -7.42 | 174.05 (122.68 to 239.25) | 167.97 (121.52 to 225.58) | -0.12 (-0.16 to -0.09) | 0 | 3051 (2975 to 3128) | 1506 (140 to 1611) | -50.63 | 4.30 (4.19 to 4.41) | 2.20 (2.05 to 2.36) | -2.13 (-2.29 to -1.96) | 0 |
| Andean Latin America | 14634 (10695 to 19744) | 17691 (13083 to 23663) | 20.89 | 98.48 (71.91 to 132.98) | 97.65 (72.31 to 130.47) | -0.03 (-0.07 to 0.01) | 0.146 | 1417 (1180 to 1741) | 1022 (785 to 1297) | -27.89 | 9.52 (7.93 to 11.68) | 5.64 (4.33 to 7.17) | -1.69 (-2.09 to -1.30) | 0 |
| Caribbean | 11269 (7884 to 15563) | 11158 (7728 to 15514) | -0.98 | 98.65 (68.89 to 136.49) | 96.95 (67.29 to 134.47) | -0.04 (-0.07 to -0.02) | 0.001 | 983 (711 to 1272) | 757 (523 to 1023) | -22.99 | 8.55 (6.21 to 11.05) | 6.63 (4.56 to 8.98) | -0.73 (-0.88 to -0.58) | 0 |
| Central Latin America | 82274 (57100 to 115576) | 76622 (53131 to 107877) | -6.87 | 127.75 (88.57 to 179.66) | 120.70 (84.12 to 169.11) | -0.19 (-0.24 to -0.14) | 0 | 5111 (4792 to 5501) | 2832 (2394 to 3404) | -44.6 | 7.92 (7.43 to 8.52) | 4.43 (3.72 to 5.36) | -1.91 (-2.15 to -1.67) | 0 |
| Tropical Latin America | 50518 (36819 to 66985) | 32647 (23616 to 44127) | -35.38 | 94.07 (68.83 to 124.32) | 64.84 (46.94 to 87.60) | -1.18 (-1.27 to -1.10) | 0 | 3403 (3002 to 3796) | 1838 (1482 to 2161) | -45.97 | 6.45 (5.69 to 7.22) | 3.66 (2.94 to 4.31) | -1.86 (-2.11 to -1.61) | 0 |
| North Africa and Middle East | 143825 (100238 to 201976) | 182789 (125889 to 259870) | 27.09 | 103.18 (71.79 to 145.21) | 99.12 (68.42 to 140.64) | -0.13 (-0.15 to -0.11) | 0 | 10598 (8080 to 13360) | 7603 (6057 to 9072) | -28.27 | 7.50 (5.73 to 9.43) | 4.15 (3.30 to 4.95) | -1.87 (-1.96 to -1.78) | 0 |
| South Asia | 750636 (506092 to 1077448) | 1055506 (693825 to 1526656) | 40.61 | 174.19 (117.23 to 250.60) | 206.37 (136.53 to 296.84) | 0.55 (0.51 to 0.59) | 0 | 26438 (18689 to 35119) | 19808 (16400 to 23808) | -25.08 | 6.05 (4.29 to 8.03) | 3.94 (3.25 to 4.76) | -1.33 (-1.58 to -1.09) | 0 |
| East Asia | 757830 (516361 to 1082761) | 563810 (396307 to 787611) | -25.6 | 229.06 (156.00 to 327.28) | 208.69 (147.72 to 289.68) | -0.31 (-0.38 to -0.25) | 0 | 44216 (34426 to 53777) | 10193 (7925 to 12742) | -76.95 | 13.41 (10.44 to 16.31) | 3.82 (2.95 to 4.81) | -4.07 (-4.39 to -3.75) | 0 |
| Oceania | 3906 (2616 to 5657) | 7427 (4995 to 10726) | 90.13 | 146.81 (98.01 to 213.39) | 147.42 (98.77 to 213.90) | 0.02 (-0.00 to 0.03) | 0.065 | 93 (56 to 136) | 173 (110 to 259) | 86.66 | 3.44 (2.07 to 5.03) | 3.38 (2.15 to 5.03) | -0.03 (-0.47 to 0.41) | 0.882 |
| Southeast Asia | 285139 (189632 to 412287) | 282740 (192644 to 402668) | -0.84 | 166.77 (111.05 to 240.79) | 163.48 (111.82 to 231.96) | -0.07 (-0.07 to -0.06) | 0 | 10376 (7129 to 14079) | 6774 (5419 to 8186) | -34.72 | 6.10 (4.18 to 8.29) | 3.93 (3.14 to 4.77) | -1.42 (-1.57 to -1.27) | 0 |
| Central Sub-Saharan Africa | 14722 (9932 to 20928) | 33225 (21880 to 47976) | 125.68 | 59.12 (39.51 to 84.74) | 56.87 (37.41 to 82.19) | -0.13 (-0.13 to -0.12) | 0 | 1189 (703 to 1678) | 1419 (1046 to 1899) | 19.28 | 4.40 (2.66 to 6.17) | 2.41 (1.78 to 3.22) | -1.94 (-2.04 to -1.83) | 0 |
| Eastern Sub-Saharan Africa | 48438 (35158 to 65088) | 88479 (63506 to 122456) | 82.67 | 53.31 (38.42 to 72.18) | 49.61 (35.57 to 68.73) | -0.23 (-0.27 to -0.19) | 0 | 10448 (7511 to 13635) | 12537 (9082 to 16460) | 19.99 | 10.91 (7.87 to 14.20) | 6.98 (5.07 to 9.16) | -1.41 (-1.54 to -1.27) | 0 |
| Southern Sub-Saharan Africa | 32631 (21755 to 47083) | 38561 (25822 to 55812) | 18.17 | 158.51 (105.53 to 229.02) | 158.98 (106.66 to 229.67) | 0.01 (-0.01 to 0.02) | 0.359 | 651 (509 to 798) | 875 (695 to 1083) | 34.28 | 3.13 (2.45 to 3.84) | 3.64 (2.88 to 4.52) | 0.48 (-0.06 to 1.02) | 0.084 |
| Western Sub-Saharan Africa | 56189 (39463 to 77686) | 135430 (92917 to 190023) | 141.03 | 64.60 (44.86 to 90.16) | 63.42 (43.36 to 89.25) | -0.06 (-0.08 to -0.04) | 0 | 6155 (4746 to 7591) | 10727 (6645 to 14776) | 74.27 | 6.50 (4.99 to 8.03) | 4.88 (3.04 to 6.70) | -0.98 (-1.11 to -0.84) | 0 |

Estimates are for individuals aged 0-14 years. Absolute incidence, mortality represent the total childhood cancer (0-14 years, both sexes combined) values. Rates are reported per 100000 population. UI=uncertainty interval. CI=confidence interval. AAPC=average annual percent change. SDI=Socio-demographic Index. GBD=Global Burden of Disease.
