# Supplementary material for: Childhood cancer burden and health inequality: A systematic analysis from the global burden of diseases study 2021
Source: PLoS One. 2026 Jan 27;21(1):e0341303. doi: 10.1371/journal.pone.0341303 (PMC12843563; doi:10.1371/journal.pone.0341303)
Supplement: S3 Table — (DOCX) [file pone.0341303.s015.docx]

**S3 Table. Childhood Cancer Burden by Different Countries and Territories from 1990 to 2021**

| **Categories** | **Age-standardized incidence rate** | | | | **Age-standardized mortality rate** | | | | **Age-standardized DALYs rate** | | | |
| --- | --- | --- | --- | --- | --- | --- | --- | --- | --- | --- | --- | --- |
|  | **Rates in 1990**  **(95% UI)** | **Rates in 2021 (95% UI)** | **AAPC, %**  **(95% CI)** | ***P*** | **Rates in 1990**  **(95% UI)** | **Rates in 2021 (95% UI)** | **AAPC, %**  **(95% CI)** | ***P*** | **Rates in 1990**  **(95% UI)** | **Rates in 2021 (95% UI)** | **AAPC, %**  **(95% CI)** | ***P*** |
| Afghanistan | 95.50 (64.41 to 136.99) | 93.70 (64.10 to 133.76) | -0.07 (-0.15 to 0.02) | 0.117 | 8.38 (3.81 to 13.57) | 6.36 (4.39 to 9.02) | -0.84 (-1.70 to 0.02) | 0.056 | 703.93 (315.38 to 1143.11) | 532.41 (366.52 to 757.16) | -0.85 (-1.73 to 0.03) | 0.058 |
| Albania | 501.43 (341.37 to 698.47) | 500.93 (339.62 to 696.63) | -0.01 (-0.04 to 0.03) | 0.68 | 9.62 (6.70 to 11.97) | 5.25 (3.92 to 7.01) | -2.13 (-2.79 to -1.47) | 0 | 816.34 (566.62 to 1016.48) | 451.06 (337.04 to 601.16) | -2.10 (-2.75 to -1.46) | 0 |
| Algeria | 96.02 (66.61 to 136.08) | 93.93 (63.91 to 133.82) | -0.07 (-0.08 to -0.05) | 0 | 6.23 (4.47 to 8.34) | 2.88 (2.11 to 3.82) | -2.42 (-2.69 to -2.16) | 0 | 522.68 (374.80 to 701.78) | 242.60 (177.02 to 322.08) | -2.41 (-2.69 to -2.14) | 0 |
| American Samoa | 145.79 (97.46 to 212.26) | 149.01 (100.21 to 216.54) | 0.07 (0.06 to 0.09) | 0 | 2.24 (1.70 to 2.92) | 2.40 (1.68 to 3.29) | 0.36 (0.14 to 0.57) | 0.001 | 187.65 (142.90 to 244.73) | 202.64 (141.31 to 277.66) | 0.38 (0.08 to 0.67) | 0.013 |
| Andorra | 175.95 (122.42 to 242.70) | 161.73 (112.35 to 224.71) | -0.23 (-0.43 to -0.04) | 0.019 | 6.17 (3.95 to 8.85) | 2.32 (1.74 to 3.00) | -3.08 (-3.78 to -2.37) | 0 | 538.56 (342.55 to 772.96) | 203.39 (151.56 to 264.31) | -3.06 (-3.78 to -2.33) | 0 |
| Angola | 61.05 (40.75 to 87.13) | 57.75 (38.32 to 83.28) | -0.18 (-0.20 to -0.16) | 0 | 5.96 (3.18 to 9.08) | 2.83 (1.90 to 4.01) | -2.35 (-2.58 to -2.11) | 0 | 510.45 (271.01 to 780.09) | 239.06 (160.21 to 338.39) | -2.39 (-2.61 to -2.16) | 0 |
| Antigua and Barbuda | 91.82 (62.52 to 129.85) | 93.78 (64.40 to 131.27) | 0.06 (0.03 to 0.10) | 0 | 3.41 (2.78 to 4.10) | 3.37 (2.86 to 3.95) | -0.01 (-0.42 to 0.41) | 0.964 | 287.93 (234.08 to 346.74) | 285.44 (241.73 to 336.00) | -0.08 (-0.82 to 0.68) | 0.845 |
| Argentina | 170.99 (115.65 to 241.93) | 169.44 (114.15 to 240.35) | -0.04 (-0.09 to 0.01) | 0.128 | 5.66 (5.18 to 6.14) | 3.43 (2.94 to 4.00) | -1.72 (-2.27 to -1.17) | 0 | 475.83 (435.60 to 516.45) | 288.24 (246.29 to 336.45) | -1.58 (-1.96 to -1.20) | 0 |
| Armenia | 337.48 (227.19 to 474.80) | 385.23 (263.89 to 529.97) | 0.45 (0.38 to 0.52) | 0 | 7.02 (5.84 to 8.27) | 3.81 (3.00 to 4.86) | -1.91 (-2.94 to -0.88) | 0 | 591.51 (491.24 to 698.26) | 319.92 (251.26 to 410.90) | -1.92 (-2.94 to -0.89) | 0 |
| Australia | 90.11 (63.40 to 124.44) | 88.69 (61.97 to 122.72) | -0.05 (-0.17 to 0.07) | 0.434 | 3.59 (3.33 to 3.86) | 1.90 (1.65 to 2.17) | -2.04 (-2.71 to -1.35) | 0 | 305.45 (283.03 to 329.12) | 163.69 (141.26 to 188.47) | -1.98 (-2.66 to -1.30) | 0 |
| Austria | 331.98 (266.69 to 395.62) | 304.72 (234.19 to 388.61) | -0.26 (-0.35 to -0.17) | 0 | 3.64 (3.32 to 3.99) | 1.74 (1.51 to 2.00) | -2.27 (-3.33 to -1.21) | 0 | 313.36 (284.86 to 343.72) | 152.47 (131.68 to 176.05) | -2.22 (-3.23 to -1.20) | 0 |
| Azerbaijan | 390.95 (263.01 to 554.27) | 388.87 (262.16 to 550.72) | -0.02 (-0.02 to -0.01) | 0 | 9.90 (7.52 to 12.97) | 7.37 (5.30 to 10.20) | -0.89 (-1.13 to -0.65) | 0 | 835.03 (632.74 to 1094.80) | 616.68 (443.06 to 856.66) | -0.98 (-1.44 to -0.53) | 0 |
| Bahamas | 93.33 (64.01 to 131.40) | 92.87 (63.49 to 130.91) | -0.00 (-0.11 to 0.11) | 0.979 | 3.98 (3.27 to 4.81) | 2.86 (2.21 to 3.78) | -0.85 (-2.05 to 0.36) | 0.169 | 336.72 (275.23 to 407.27) | 241.79 (185.47 to 320.19) | -0.85 (-2.03 to 0.34) | 0.159 |
| Bahrain | 91.84 (62.50 to 132.14) | 91.90 (62.44 to 132.18) | 0.00 (-0.08 to 0.08) | 0.933 | 3.58 (2.90 to 4.39) | 1.74 (1.38 to 2.23) | -2.08 (-3.00 to -1.15) | 0 | 300.20 (243.18 to 368.07) | 146.23 (115.42 to 188.18) | -2.18 (-3.09 to -1.25) | 0 |
| Bangladesh | 127.42 (86.92 to 179.48) | 125.52 (85.59 to 177.27) | -0.05 (-0.07 to -0.02) | 0 | 8.53 (4.73 to 12.98) | 4.91 (3.60 to 6.51) | -1.78 (-2.07 to -1.49) | 0 | 720.02 (396.47 to 1097.46) | 408.15 (299.33 to 541.40) | -1.83 (-2.11 to -1.55) | 0 |
| Barbados | 97.03 (68.03 to 135.18) | 95.67 (66.64 to 133.62) | -0.04 (-0.21 to 0.12) | 0.597 | 6.11 (5.12 to 7.18) | 3.91 (2.78 to 5.34) | -1.25 (-2.60 to 0.13) | 0.076 | 511.66 (427.76 to 601.52) | 330.03 (234.26 to 451.78) | -1.23 (-2.59 to 0.14) | 0.077 |
| Belarus | 435.80 (292.41 to 623.12) | 434.91 (291.95 to 622.52) | -0.01 (-0.04 to 0.02) | 0.556 | 5.00 (4.29 to 5.79) | 2.77 (2.13 to 3.51) | -1.55 (-4.77 to 1.77) | 0.356 | 421.99 (361.74 to 489.17) | 235.88 (180.32 to 300.95) | -1.50 (-4.82 to 1.93) | 0.387 |
| Belgium | 171.05 (119.72 to 237.74) | 170.07 (119.02 to 235.51) | -0.04 (-0.42 to 0.33) | 0.819 | 4.38 (3.95 to 4.84) | 2.64 (2.25 to 3.05) | -1.93 (-3.44 to -0.40) | 0.014 | 374.61 (338.09 to 416.12) | 228.02 (193.71 to 264.50) | -1.90 (-3.44 to -0.34) | 0.017 |
| Belize | 95.15 (65.65 to 133.18) | 91.31 (61.93 to 129.17) | -0.14 (-0.22 to -0.06) | 0.001 | 5.92 (5.10 to 6.84) | 2.63 (2.16 to 3.20) | -2.65 (-3.98 to -1.30) | 0 | 504.41 (433.89 to 583.04) | 223.52 (183.27 to 272.21) | -2.66 (-3.82 to -1.48) | 0 |
| Benin | 60.87 (41.72 to 86.47) | 60.28 (40.46 to 86.25) | -0.03 (-0.09 to 0.03) | 0.353 | 5.83 (4.20 to 7.53) | 4.64 (2.85 to 6.62) | -0.84 (-1.13 to -0.54) | 0 | 502.53 (362.20 to 649.01) | 397.40 (242.66 to 567.17) | -0.86 (-1.15 to -0.56) | 0 |
| Bermuda | 94.42 (64.77 to 131.93) | 99.81 (70.39 to 137.28) | 0.17 (0.11 to 0.23) | 0 | 3.96 (3.14 to 4.87) | 2.98 (2.35 to 3.66) | -0.92 (-1.51 to -0.32) | 0.003 | 336.97 (266.23 to 414.61) | 257.91 (202.71 to 317.84) | -0.87 (-1.46 to -0.26) | 0.005 |
| Bhutan | 124.02 (82.77 to 175.29) | 125.04 (84.84 to 177.68) | 0.04 (-0.01 to 0.08) | 0.111 | 6.17 (3.02 to 9.27) | 4.61 (3.06 to 6.69) | -0.75 (-1.56 to 0.07) | 0.072 | 527.49 (260.85 to 791.88) | 389.56 (259.61 to 565.92) | -0.79 (-1.59 to 0.02) | 0.056 |
| Bolivia (Plurinational State of) | 104.66 (73.92 to 144.09) | 100.26 (70.53 to 140.94) | -0.13 (-0.16 to -0.11) | 0 | 13.35 (9.09 to 18.17) | 7.81 (5.71 to 10.58) | -1.70 (-1.84 to -1.56) | 0 | 1131.43 (765.93 to 1543.47) | 657.99 (480.66 to 891.42) | -1.72 (-1.86 to -1.58) | 0 |
| Bosnia and Herzegovina | 496.72 (335.72 to 695.93) | 495.15 (334.08 to 693.70) | -0.01 (-0.02 to 0.00) | 0.125 | 4.32 (3.33 to 5.39) | 2.46 (1.96 to 3.04) | -1.92 (-2.86 to -0.97) | 0 | 360.86 (277.45 to 451.40) | 204.59 (162.23 to 253.34) | -1.92 (-2.86 to -0.96) | 0 |
| Botswana | 147.79 (97.60 to 213.80) | 148.91 (99.41 to 214.78) | 0.03 (-0.20 to 0.27) | 0.782 | 2.71 (1.87 to 3.73) | 3.57 (2.46 to 5.03) | 0.82 (0.22 to 1.43) | 0.007 | 227.39 (157.23 to 312.88) | 301.36 (207.22 to 426.58) | 0.85 (0.24 to 1.46) | 0.006 |
| Brazil | 94.59 (69.29 to 124.98) | 64.28 (46.56 to 86.80) | -1.23 (-1.32 to -1.14) | 0 | 6.48 (5.68 to 7.27) | 3.63 (2.92 to 4.27) | -1.93 (-2.24 to -1.62) | 0 | 548.86 (480.75 to 616.50) | 303.71 (244.18 to 358.79) | -1.97 (-2.29 to -1.66) | 0 |
| Brunei Darussalam | 403.87 (270.92 to 585.66) | 401.01 (267.92 to 580.90) | -0.02 (-0.04 to -0.01) | 0.006 | 5.64 (4.44 to 7.07) | 3.46 (2.73 to 4.41) | -1.61 (-2.21 to -1.01) | 0 | 470.99 (371.57 to 588.76) | 295.39 (233.30 to 376.77) | -1.54 (-2.12 to -0.96) | 0 |
| Bulgaria | 500.79 (339.79 to 699.53) | 496.16 (334.61 to 694.77) | -0.03 (-0.07 to 0.01) | 0.099 | 6.44 (5.65 to 7.17) | 3.00 (2.49 to 3.59) | -2.34 (-4.36 to -0.27) | 0.027 | 541.18 (475.01 to 602.99) | 251.64 (208.27 to 302.01) | -2.73 (-4.87 to -0.55) | 0.014 |
| Burkina Faso | 60.94 (41.26 to 86.68) | 60.43 (40.74 to 86.13) | -0.03 (-0.06 to 0.01) | 0.173 | 5.65 (4.04 to 7.43) | 4.83 (3.15 to 6.67) | -0.48 (-0.81 to -0.15) | 0.005 | 488.46 (349.34 to 641.40) | 414.98 (270.04 to 574.07) | -0.50 (-0.83 to -0.17) | 0.003 |
| Burundi | 51.53 (37.58 to 69.97) | 45.68 (31.56 to 64.83) | -0.38 (-0.47 to -0.30) | 0 | 10.54 (7.95 to 13.89) | 5.44 (3.07 to 8.83) | -2.07 (-2.39 to -1.74) | 0 | 898.73 (678.75 to 1183.64) | 458.20 (256.34 to 748.28) | -2.10 (-2.44 to -1.76) | 0 |
| Cabo Verde | 58.80 (39.48 to 84.90) | 62.06 (42.15 to 87.94) | 0.17 (0.12 to 0.22) | 0 | 3.74 (2.36 to 5.30) | 4.03 (2.88 to 5.81) | 0.26 (-0.11 to 0.62) | 0.167 | 321.68 (203.52 to 455.14) | 341.68 (243.01 to 494.61) | 0.20 (-0.17 to 0.58) | 0.289 |
| Cambodia | 140.97 (93.84 to 202.13) | 137.63 (91.55 to 198.05) | -0.07 (-0.08 to -0.06) | 0 | 7.67 (4.26 to 11.63) | 4.56 (3.36 to 6.25) | -1.62 (-1.73 to -1.51) | 0 | 646.02 (355.66 to 982.84) | 381.35 (281.38 to 521.85) | -1.64 (-1.75 to -1.53) | 0 |
| Cameroon | 60.20 (40.99 to 85.72) | 59.88 (40.37 to 85.37) | -0.01 (-0.10 to 0.07) | 0.748 | 5.03 (3.58 to 6.60) | 4.24 (2.86 to 5.73) | -0.56 (-1.13 to 0.01) | 0.055 | 431.28 (306.39 to 564.60) | 360.55 (243.51 to 487.59) | -0.59 (-1.16 to -0.01) | 0.046 |
| Canada | 243.72 (165.80 to 346.88) | 240.02 (161.83 to 342.89) | -0.03 (-0.11 to 0.04) | 0.405 | 3.37 (3.15 to 3.63) | 1.90 (1.66 to 2.19) | -1.77 (-2.54 to -0.98) | 0 | 290.29 (270.58 to 313.49) | 165.60 (143.93 to 191.34) | -1.70 (-2.47 to -0.93) | 0 |
| Central African Republic | 59.54 (39.91 to 85.09) | 58.11 (38.57 to 83.35) | -0.08 (-0.12 to -0.04) | 0 | 4.98 (2.84 to 7.36) | 3.66 (2.35 to 5.31) | -1.02 (-1.48 to -0.56) | 0 | 424.10 (240.87 to 628.24) | 309.56 (198.23 to 449.55) | -1.05 (-1.50 to -0.59) | 0 |
| Chad | 59.50 (40.21 to 84.99) | 59.69 (40.31 to 85.22) | 0.01 (-0.03 to 0.06) | 0.634 | 4.52 (3.04 to 6.07) | 4.59 (3.05 to 6.27) | 0.06 (-0.40 to 0.52) | 0.792 | 388.03 (261.63 to 521.31) | 390.74 (260.36 to 533.26) | 0.03 (-0.42 to 0.49) | 0.892 |
| Chile | 202.77 (135.04 to 292.87) | 199.33 (152.55 to 256.13) | -0.06 (-0.27 to 0.16) | 0.597 | 4.36 (3.99 to 4.77) | 2.23 (1.94 to 2.54) | -2.22 (-2.99 to -1.44) | 0 | 364.53 (333.14 to 398.53) | 187.64 (163.08 to 214.17) | -2.19 (-2.95 to -1.43) | 0 |
| China | 230.51 (156.92 to 329.33) | 209.36 (148.26 to 290.14) | -0.32 (-0.39 to -0.26) | 0 | 13.67 (10.63 to 16.66) | 3.83 (2.96 to 4.82) | -4.13 (-4.45 to -3.80) | 0 | 1170.21 (909.60 to 1429.09) | 328.10 (251.32 to 416.74) | -4.13 (-4.46 to -3.79) | 0 |
| Colombia | 118.14 (81.85 to 165.20) | 119.14 (82.97 to 166.64) | 0.02 (-0.04 to 0.08) | 0.511 | 7.52 (6.59 to 8.48) | 4.53 (3.64 to 5.55) | -1.72 (-2.41 to -1.03) | 0 | 631.20 (552.00 to 712.31) | 378.38 (303.36 to 466.27) | -1.74 (-2.43 to -1.04) | 0 |
| Comoros | 49.83 (35.71 to 68.49) | 48.88 (34.88 to 67.99) | -0.10 (-0.41 to 0.22) | 0.542 | 8.94 (5.81 to 12.18) | 7.25 (4.93 to 10.07) | -0.86 (-2.26 to 0.57) | 0.237 | 762.81 (497.64 to 1039.01) | 614.77 (416.70 to 857.65) | -0.87 (-2.24 to 0.52) | 0.219 |
| Congo | 58.78 (39.16 to 84.23) | 57.08 (37.52 to 82.42) | -0.09 (-0.13 to -0.05) | 0 | 3.99 (2.59 to 5.54) | 2.37 (1.76 to 3.14) | -1.67 (-2.22 to -1.11) | 0 | 340.76 (222.03 to 472.62) | 199.47 (147.69 to 264.57) | -1.72 (-2.26 to -1.17) | 0 |
| Cook Islands | 145.16 (96.62 to 212.34) | 148.94 (98.99 to 217.06) | 0.09 (0.06 to 0.12) | 0 | 1.16 (0.82 to 1.61) | 1.02 (0.60 to 1.66) | -0.02 (-0.95 to 0.92) | 0.966 | 98.08 (69.19 to 135.61) | 89.33 (52.29 to 146.02) | 0.11 (-0.84 to 1.08) | 0.818 |
| Costa Rica | 117.69 (81.85 to 164.49) | 118.14 (81.95 to 165.02) | 0.02 (-0.02 to 0.07) | 0.323 | 5.42 (5.00 to 5.93) | 3.92 (3.38 to 4.53) | -0.76 (-1.02 to -0.49) | 0 | 456.99 (421.07 to 499.82) | 327.46 (280.91 to 378.96) | -0.77 (-1.03 to -0.51) | 0 |
| Coted'Ivoire | 58.99 (39.82 to 84.75) | 58.48 (39.18 to 84.26) | -0.03 (-0.07 to 0.02) | 0.194 | 4.02 (2.82 to 5.58) | 3.25 (2.01 to 5.26) | -0.70 (-1.12 to -0.28) | 0.001 | 343.29 (241.06 to 477.79) | 275.51 (169.85 to 446.61) | -0.73 (-1.15 to -0.31) | 0.001 |
| Croatia | 504.84 (344.55 to 712.59) | 501.68 (375.36 to 648.44) | -0.03 (-0.15 to 0.08) | 0.556 | 3.98 (3.45 to 4.59) | 2.27 (1.86 to 2.79) | -2.06 (-3.72 to -0.37) | 0.017 | 336.22 (290.76 to 388.52) | 195.30 (160.12 to 240.96) | -2.00 (-3.65 to -0.33) | 0.019 |
| Cuba | 96.61 (67.36 to 134.75) | 95.09 (65.65 to 132.76) | -0.07 (-0.17 to 0.04) | 0.203 | 5.31 (4.80 to 5.91) | 3.11 (2.56 to 3.72) | -1.75 (-2.51 to -0.98) | 0 | 445.99 (403.66 to 497.24) | 262.42 (215.91 to 315.00) | -1.73 (-2.49 to -0.95) | 0 |
| Cyprus | 101.56 (70.17 to 141.86) | 104.09 (72.77 to 145.37) | 0.06 (-0.29 to 0.42) | 0.724 | 3.02 (2.36 to 3.88) | 1.63 (1.23 to 2.08) | -1.99 (-3.13 to -0.84) | 0.001 | 256.75 (200.67 to 330.65) | 141.82 (106.10 to 182.18) | -1.92 (-3.06 to -0.76) | 0.001 |
| Czechia | 453.26 (305.18 to 638.53) | 451.31 (302.59 to 636.88) | -0.03 (-0.06 to -0.00) | 0.039 | 4.45 (3.96 to 5.00) | 1.78 (1.44 to 2.20) | -3.01 (-3.49 to -2.54) | 0 | 374.15 (332.69 to 420.93) | 151.01 (121.74 to 187.69) | -2.97 (-3.44 to -2.50) | 0 |
| Democratic People's Republic of Korea | 190.48 (130.31 to 272.70) | 185.85 (125.49 to 266.89) | -0.08 (-0.09 to -0.07) | 0 | 8.08 (5.41 to 11.28) | 4.46 (2.89 to 6.96) | -1.89 (-1.97 to -1.81) | 0 | 688.93 (460.61 to 962.85) | 377.76 (244.10 to 593.10) | -1.91 (-1.99 to -1.82) | 0 |
| Democratic Republic of the Congo | 58.62 (39.18 to 84.03) | 56.42 (37.01 to 81.68) | -0.13 (-0.14 to -0.12) | 0 | 4.00 (2.36 to 5.63) | 2.16 (1.54 to 3.01) | -2.01 (-2.12 to -1.90) | 0 | 341.58 (200.53 to 480.87) | 181.64 (128.70 to 253.08) | -2.06 (-2.17 to -1.96) | 0 |
| Denmark | 162.11 (111.84 to 225.41) | 161.56 (112.20 to 225.41) | 0.00 (-0.18 to 0.18) | 0.978 | 3.98 (3.58 to 4.38) | 1.88 (1.58 to 2.26) | -2.32 (-2.68 to -1.97) | 0 | 338.99 (304.46 to 373.00) | 164.60 (137.70 to 198.30) | -2.21 (-2.55 to -1.87) | 0 |
| Djibouti | 49.17 (35.22 to 68.46) | 46.82 (32.76 to 65.98) | -0.12 (-0.30 to 0.05) | 0.165 | 8.61 (6.17 to 11.83) | 6.07 (3.68 to 9.25) | -0.89 (-1.48 to -0.30) | 0.003 | 738.22 (529.00 to 1015.99) | 513.72 (309.79 to 785.97) | -0.92 (-1.52 to -0.32) | 0.003 |
| Dominica | 92.85 (63.68 to 130.64) | 101.71 (72.34 to 139.99) | 0.30 (0.28 to 0.32) | 0 | 4.65 (3.64 to 5.82) | 7.81 (5.87 to 10.02) | 1.66 (1.47 to 1.86) | 0 | 390.07 (304.98 to 489.04) | 660.30 (495.38 to 849.23) | 1.68 (1.48 to 1.88) | 0 |
| Dominican Republic | 97.95 (68.36 to 136.11) | 95.27 (65.90 to 132.78) | -0.08 (-0.14 to -0.02) | 0.006 | 8.32 (6.11 to 10.49) | 5.13 (3.66 to 7.11) | -1.47 (-2.23 to -0.71) | 0 | 710.54 (519.28 to 896.51) | 432.43 (307.10 to 600.01) | -1.51 (-2.26 to -0.76) | 0 |
| Ecuador | 87.03 (66.27 to 110.57) | 91.72 (71.61 to 114.32) | 0.17 (0.04 to 0.30) | 0.012 | 6.01 (5.24 to 6.85) | 4.79 (3.84 to 5.94) | -0.85 (-1.79 to 0.09) | 0.077 | 502.83 (438.11 to 572.65) | 399.48 (318.90 to 496.95) | -0.86 (-1.79 to 0.08) | 0.074 |
| Egypt | 95.06 (65.61 to 135.03) | 94.07 (64.63 to 134.85) | -0.03 (-0.08 to 0.03) | 0.302 | 6.95 (5.15 to 10.60) | 4.28 (3.34 to 5.55) | -1.50 (-1.82 to -1.18) | 0 | 586.08 (433.20 to 895.70) | 359.17 (279.34 to 466.18) | -1.52 (-1.84 to -1.19) | 0 |
| El Salvador | 116.83 (80.94 to 164.11) | 114.84 (79.49 to 161.84) | -0.06 (-0.12 to 0.00) | 0.06 | 7.54 (6.01 to 9.42) | 4.11 (3.04 to 5.47) | -2.12 (-2.63 to -1.61) | 0 | 633.98 (504.06 to 793.80) | 339.59 (251.04 to 454.14) | -2.17 (-2.66 to -1.67) | 0 |
| Equatorial Guinea | 58.42 (38.96 to 83.94) | 56.08 (37.54 to 80.30) | -0.14 (-0.16 to -0.11) | 0 | 4.12 (2.34 to 6.02) | 2.27 (1.18 to 4.41) | -1.96 (-2.40 to -1.50) | 0 | 349.91 (197.29 to 511.18) | 190.38 (99.53 to 366.88) | -1.98 (-2.43 to -1.54) | 0 |
| Eritrea | 48.58 (34.02 to 67.76) | 47.93 (33.99 to 66.68) | -0.03 (-0.09 to 0.03) | 0.295 | 8.57 (5.84 to 11.83) | 7.35 (4.90 to 10.59) | -0.44 (-0.70 to -0.17) | 0.001 | 730.47 (496.66 to 1008.33) | 624.31 (414.69 to 900.24) | -0.45 (-0.72 to -0.17) | 0.001 |
| Estonia | 445.62 (302.02 to 633.28) | 452.55 (312.48 to 638.36) | 0.06 (-0.02 to 0.14) | 0.13 | 5.84 (5.16 to 6.57) | 2.30 (1.87 to 2.78) | -2.77 (-4.31 to -1.21) | 0.001 | 496.97 (439.70 to 560.13) | 206.79 (167.21 to 252.61) | -2.59 (-4.17 to -1.00) | 0.002 |
| Eswatini | 148.75 (98.89 to 214.76) | 148.11 (99.22 to 213.07) | -0.01 (-0.03 to 0.01) | 0.244 | 3.52 (2.40 to 5.02) | 3.90 (2.73 to 5.36) | 0.37 (-0.24 to 0.97) | 0.236 | 295.63 (201.42 to 422.78) | 326.42 (228.97 to 449.67) | 0.35 (-0.26 to 0.96) | 0.261 |
| Ethiopia | 58.29 (39.24 to 80.54) | 52.34 (37.25 to 72.89) | -0.35 (-0.42 to -0.27) | 0 | 14.04 (6.34 to 21.77) | 7.51 (5.17 to 11.07) | -2.02 (-2.39 to -1.65) | 0 | 1191.85 (533.65 to 1851.06) | 634.59 (435.04 to 939.38) | -2.04 (-2.41 to -1.66) | 0 |
| Fiji | 148.68 (99.61 to 216.09) | 151.12 (102.10 to 219.01) | 0.06 (0.04 to 0.08) | 0 | 3.93 (2.63 to 5.44) | 4.27 (2.82 to 6.10) | 0.34 (-0.12 to 0.79) | 0.146 | 326.45 (217.83 to 452.19) | 358.08 (235.99 to 511.78) | 0.37 (-0.08 to 0.82) | 0.107 |
| Finland | 172.57 (117.07 to 244.01) | 177.21 (121.49 to 248.20) | 0.11 (-0.14 to 0.35) | 0.382 | 2.63 (2.37 to 2.91) | 1.97 (1.68 to 2.33) | -1.47 (-1.74 to -1.21) | 0 | 223.17 (200.96 to 246.95) | 171.12 (145.86 to 202.05) | -1.43 (-1.88 to -0.97) | 0 |
| France | 157.94 (108.77 to 220.25) | 160.58 (111.73 to 223.91) | 0.08 (0.07 to 0.09) | 0 | 3.85 (3.59 to 4.11) | 2.32 (2.02 to 2.63) | -1.59 (-2.33 to -0.85) | 0 | 328.43 (306.67 to 351.26) | 202.97 (176.55 to 231.48) | -1.50 (-2.26 to -0.74) | 0 |
| Gabon | 57.97 (38.66 to 83.41) | 57.95 (38.58 to 83.25) | -0.00 (-0.03 to 0.02) | 0.763 | 3.17 (2.22 to 4.22) | 2.44 (1.60 to 3.57) | -0.91 (-1.36 to -0.46) | 0 | 269.10 (187.44 to 358.30) | 205.48 (134.32 to 300.90) | -0.94 (-1.37 to -0.51) | 0 |
| Gambia | 61.33 (41.93 to 87.03) | 59.53 (39.93 to 85.12) | -0.10 (-0.23 to 0.03) | 0.119 | 5.45 (3.84 to 7.15) | 3.70 (2.44 to 5.64) | -1.28 (-2.47 to -0.08) | 0.036 | 470.41 (332.60 to 617.16) | 316.11 (207.98 to 482.24) | -1.32 (-2.50 to -0.11) | 0.032 |
| Georgia | 303.18 (205.73 to 424.47) | 223.20 (171.20 to 285.84) | -0.97 (-1.01 to -0.93) | 0 | 6.79 (5.56 to 7.95) | 2.86 (2.18 to 3.60) | -2.37 (-3.28 to -1.46) | 0 | 568.40 (465.48 to 666.78) | 239.29 (181.94 to 302.01) | -2.36 (-3.18 to -1.54) | 0 |
| Germany | 193.80 (133.36 to 273.30) | 194.23 (133.85 to 273.54) | 0.01 (-0.13 to 0.14) | 0.937 | 4.12 (3.85 to 4.38) | 2.20 (1.96 to 2.46) | -2.16 (-2.59 to -1.73) | 0 | 349.47 (327.06 to 372.06) | 190.20 (168.61 to 213.32) | -2.09 (-2.52 to -1.66) | 0 |
| Ghana | 61.87 (42.53 to 87.26) | 58.45 (39.34 to 84.10) | -0.18 (-0.22 to -0.14) | 0 | 6.67 (4.11 to 9.49) | 3.23 (2.06 to 4.78) | -2.27 (-2.56 to -1.98) | 0 | 566.77 (349.41 to 805.71) | 273.45 (173.81 to 405.60) | -2.29 (-2.58 to -2.00) | 0 |
| Greece | 176.00 (123.13 to 244.52) | 174.55 (130.03 to 227.46) | -0.02 (-0.15 to 0.11) | 0.779 | 4.00 (3.69 to 4.30) | 2.55 (2.18 to 2.92) | -1.34 (-2.34 to -0.34) | 0.009 | 342.37 (315.58 to 368.54) | 220.35 (188.68 to 253.40) | -1.31 (-2.26 to -0.36) | 0.007 |
| Greenland | 237.09 (159.10 to 339.68) | 230.56 (152.91 to 331.90) | -0.09 (-0.10 to -0.08) | 0 | 6.80 (4.76 to 9.41) | 2.51 (1.89 to 3.36) | -3.17 (-3.40 to -2.93) | 0 | 571.80 (399.95 to 789.79) | 213.03 (160.56 to 285.03) | -3.14 (-3.38 to -2.90) | 0 |
| Grenada | 95.39 (65.70 to 133.46) | 95.61 (66.93 to 133.10) | 0.01 (-0.09 to 0.11) | 0.808 | 6.04 (4.98 to 7.32) | 4.47 (3.67 to 5.41) | -0.94 (-1.87 to 0.00) | 0.051 | 507.29 (417.19 to 615.60) | 373.77 (306.72 to 454.09) | -0.95 (-1.82 to -0.06) | 0.036 |
| Guam | 149.82 (100.99 to 217.51) | 149.73 (100.89 to 217.54) | -0.01 (-0.13 to 0.10) | 0.795 | 2.46 (1.93 to 3.07) | 1.71 (1.17 to 2.36) | -1.69 (-2.89 to -0.48) | 0.006 | 206.27 (161.96 to 256.89) | 148.83 (102.37 to 205.12) | -1.52 (-2.64 to -0.38) | 0.009 |
| Guatemala | 116.76 (80.74 to 163.89) | 113.95 (77.95 to 160.93) | -0.08 (-0.11 to -0.05) | 0 | 7.63 (6.77 to 8.57) | 4.19 (3.43 to 5.11) | -1.95 (-2.48 to -1.42) | 0 | 647.62 (574.37 to 727.59) | 351.05 (286.44 to 429.09) | -2.00 (-2.51 to -1.48) | 0 |
| Guinea | 64.90 (45.46 to 90.47) | 60.52 (41.07 to 86.64) | -0.22 (-0.30 to -0.14) | 0 | 8.54 (6.13 to 11.35) | 4.81 (2.80 to 7.82) | -1.80 (-2.41 to -1.20) | 0 | 740.46 (532.27 to 983.45) | 413.15 (239.94 to 672.81) | -1.83 (-2.44 to -1.23) | 0 |
| Guinea-Bissau | 61.86 (42.15 to 87.59) | 58.34 (39.08 to 84.00) | -0.17 (-0.21 to -0.14) | 0 | 6.52 (4.47 to 8.84) | 3.57 (2.15 to 5.82) | -1.74 (-2.13 to -1.34) | 0 | 559.05 (383.62 to 758.29) | 301.50 (181.17 to 489.30) | -1.78 (-2.18 to -1.38) | 0 |
| Guyana | 92.46 (62.84 to 130.64) | 92.44 (63.04 to 130.40) | 0.01 (-0.07 to 0.10) | 0.752 | 5.01 (4.00 to 6.04) | 3.92 (2.99 to 5.10) | -0.66 (-1.83 to 0.52) | 0.272 | 421.75 (336.49 to 508.74) | 328.57 (250.70 to 428.77) | -0.67 (-1.81 to 0.49) | 0.256 |
| Haiti | 104.90 (71.62 to 144.64) | 100.24 (69.07 to 138.86) | -0.14 (-0.17 to -0.11) | 0 | 15.17 (6.78 to 23.59) | 10.43 (5.50 to 16.20) | -1.17 (-1.37 to -0.96) | 0 | 1291.64 (571.28 to 2010.46) | 882.73 (461.95 to 1374.28) | -1.19 (-1.39 to -0.99) | 0 |
| Honduras | 117.41 (81.61 to 164.33) | 112.12 (76.55 to 158.91) | -0.15 (-0.16 to -0.13) | 0 | 8.44 (6.73 to 10.60) | 3.42 (2.11 to 5.34) | -2.86 (-3.14 to -2.58) | 0 | 706.70 (563.78 to 888.79) | 286.57 (176.44 to 447.31) | -2.86 (-3.14 to -2.58) | 0 |
| Hungary | 499.10 (337.89 to 698.75) | 496.21 (334.55 to 695.05) | -0.02 (-0.03 to -0.00) | 0.009 | 4.65 (4.26 to 5.09) | 1.75 (1.45 to 2.13) | -3.19 (-4.44 to -1.93) | 0 | 392.20 (358.47 to 429.27) | 150.14 (124.22 to 183.07) | -3.14 (-4.38 to -1.87) | 0 |
| Iceland | 149.28 (106.16 to 205.07) | 148.10 (104.88 to 205.77) | 0.05 (-0.48 to 0.58) | 0.847 | 4.34 (3.82 to 4.87) | 2.34 (1.99 to 2.72) | -1.72 (-3.83 to 0.43) | 0.116 | 371.65 (327.54 to 418.37) | 203.62 (171.95 to 238.32) | -1.66 (-3.72 to 0.45) | 0.123 |
| India | 188.07 (126.39 to 270.67) | 233.49 (152.80 to 336.34) | 0.71 (0.68 to 0.74) | 0 | 5.44 (3.93 to 7.20) | 2.87 (2.22 to 3.65) | -2.05 (-2.45 to -1.64) | 0 | 459.31 (331.61 to 608.96) | 242.51 (187.16 to 308.84) | -2.05 (-2.44 to -1.66) | 0 |
| Indonesia | 212.14 (140.25 to 306.86) | 231.67 (160.88 to 325.74) | 0.29 (0.27 to 0.30) | 0 | 5.76 (3.48 to 8.56) | 3.94 (2.79 to 5.23) | -1.24 (-1.43 to -1.04) | 0 | 483.63 (288.93 to 724.33) | 329.00 (231.84 to 438.86) | -1.26 (-1.47 to -1.04) | 0 |
| Iran (Islamic Republic of) | 101.97 (70.83 to 142.56) | 100.30 (69.04 to 141.77) | -0.04 (-0.14 to 0.06) | 0.441 | 8.79 (6.49 to 11.39) | 4.06 (2.70 to 5.28) | -2.44 (-2.63 to -2.24) | 0 | 743.29 (547.88 to 966.37) | 339.83 (225.13 to 444.45) | -2.47 (-2.66 to -2.27) | 0 |
| Iraq | 96.89 (67.04 to 137.28) | 97.60 (67.56 to 137.71) | 0.01 (-0.03 to 0.05) | 0.576 | 7.55 (5.27 to 10.48) | 4.41 (3.25 to 5.84) | -1.85 (-2.09 to -1.62) | 0 | 631.55 (438.88 to 878.50) | 374.14 (275.40 to 497.15) | -1.81 (-2.04 to -1.57) | 0 |
| Ireland | 157.31 (107.97 to 219.55) | 154.50 (104.68 to 217.07) | -0.05 (-0.13 to 0.03) | 0.227 | 3.67 (3.30 to 4.08) | 1.45 (1.24 to 1.68) | -2.65 (-3.71 to -1.58) | 0 | 312.32 (281.31 to 347.19) | 126.81 (108.27 to 146.54) | -2.57 (-3.62 to -1.50) | 0 |
| Israel | 158.50 (108.56 to 220.83) | 155.01 (105.23 to 218.28) | -0.06 (-0.18 to 0.05) | 0.299 | 4.90 (4.46 to 5.35) | 2.01 (1.73 to 2.36) | -2.86 (-3.85 to -1.87) | 0 | 418.15 (380.90 to 457.26) | 172.92 (147.77 to 202.03) | -2.82 (-3.78 to -1.85) | 0 |
| Italy | 224.82 (156.19 to 311.89) | 251.76 (186.88 to 328.60) | 0.35 (0.27 to 0.42) | 0 | 5.27 (5.07 to 5.50) | 2.50 (2.22 to 2.79) | -2.30 (-2.77 to -1.83) | 0 | 452.75 (434.13 to 473.05) | 219.03 (193.15 to 245.25) | -2.23 (-2.69 to -1.77) | 0 |
| Jamaica | 97.42 (67.92 to 135.11) | 94.17 (64.92 to 132.00) | -0.09 (-0.19 to 0.00) | 0.059 | 6.26 (5.31 to 7.27) | 3.67 (2.88 to 4.66) | -1.54 (-2.48 to -0.60) | 0.001 | 529.67 (448.70 to 616.13) | 308.68 (242.31 to 392.66) | -1.56 (-2.49 to -0.61) | 0.001 |
| Japan | 478.60 (321.95 to 689.42) | 431.76 (301.24 to 604.52) | -0.32 (-0.39 to -0.25) | 0 | 3.49 (3.37 to 3.61) | 2.02 (1.88 to 2.15) | -1.69 (-2.08 to -1.30) | 0 | 300.86 (290.08 to 311.61) | 177.86 (165.17 to 191.27) | -1.63 (-1.99 to -1.26) | 0 |
| Jordan | 66.43 (46.77 to 90.88) | 67.97 (50.89 to 88.20) | 0.07 (0.01 to 0.13) | 0.014 | 5.92 (4.85 to 7.28) | 3.62 (2.75 to 4.72) | -1.59 (-1.75 to -1.43) | 0 | 495.97 (406.37 to 610.44) | 306.53 (232.96 to 400.14) | -1.55 (-1.71 to -1.39) | 0 |
| Kazakhstan | 389.61 (261.76 to 553.15) | 383.71 (256.32 to 546.62) | -0.05 (-0.08 to -0.03) | 0 | 7.68 (6.82 to 8.58) | 2.81 (2.33 to 3.40) | -3.37 (-4.38 to -2.35) | 0 | 646.13 (573.25 to 722.88) | 236.82 (195.90 to 287.04) | -3.37 (-4.40 to -2.33) | 0 |
| Kenya | 48.94 (34.11 to 68.53) | 47.94 (33.26 to 67.68) | -0.07 (-0.16 to 0.02) | 0.135 | 5.55 (4.08 to 7.22) | 4.24 (3.17 to 5.63) | -0.80 (-1.13 to -0.47) | 0 | 475.71 (349.31 to 619.70) | 360.25 (268.30 to 479.38) | -0.83 (-1.16 to -0.49) | 0 |
| Kiribati | 146.95 (97.79 to 214.03) | 146.83 (97.62 to 214.21) | -0.00 (-0.01 to 0.01) | 0.781 | 3.49 (2.21 to 5.00) | 2.87 (1.44 to 5.19) | -0.66 (-0.98 to -0.33) | 0 | 296.17 (187.02 to 423.57) | 241.22 (120.86 to 438.22) | -0.68 (-1.01 to -0.35) | 0 |
| Kuwait | 101.43 (71.52 to 142.08) | 97.67 (68.00 to 138.36) | -0.09 (-0.30 to 0.11) | 0.366 | 4.95 (4.32 to 5.74) | 1.90 (1.55 to 2.33) | -2.86 (-3.81 to -1.90) | 0 | 418.79 (364.81 to 485.93) | 164.86 (133.89 to 201.02) | -2.78 (-3.73 to -1.81) | 0 |
| Kyrgyzstan | 590.68 (392.10 to 843.40) | 589.06 (390.42 to 842.29) | -0.01 (-0.03 to 0.01) | 0.285 | 5.90 (4.80 to 7.20) | 3.21 (2.65 to 3.87) | -1.99 (-2.51 to -1.48) | 0 | 498.17 (405.07 to 609.11) | 268.65 (221.58 to 324.39) | -2.03 (-2.54 to -1.52) | 0 |
| Lao People's Democratic Republic | 140.86 (93.83 to 201.19) | 137.97 (91.45 to 198.61) | -0.07 (-0.09 to -0.05) | 0 | 8.11 (3.59 to 13.71) | 4.87 (3.27 to 6.97) | -1.70 (-1.83 to -1.56) | 0 | 682.33 (298.19 to 1158.12) | 406.85 (272.16 to 583.05) | -1.72 (-1.86 to -1.58) | 0 |
| Latvia | 406.27 (271.42 to 578.69) | 399.51 (265.40 to 571.42) | -0.06 (-0.14 to 0.02) | 0.151 | 5.50 (4.93 to 6.14) | 1.89 (1.53 to 2.28) | -3.14 (-5.06 to -1.17) | 0.002 | 462.32 (414.12 to 516.21) | 160.80 (129.95 to 195.38) | -3.11 (-5.02 to -1.15) | 0.002 |
| Lebanon | 93.82 (64.43 to 134.40) | 96.75 (67.18 to 137.54) | 0.11 (-0.00 to 0.22) | 0.05 | 4.72 (3.49 to 6.27) | 2.80 (1.83 to 4.13) | -1.63 (-2.25 to -1.01) | 0 | 398.02 (293.57 to 529.28) | 240.57 (155.83 to 358.09) | -1.57 (-2.22 to -0.92) | 0 |
| Lesotho | 147.02 (97.03 to 212.88) | 148.54 (98.92 to 214.25) | 0.03 (0.02 to 0.05) | 0 | 2.51 (1.81 to 3.41) | 3.73 (2.57 to 5.28) | 1.22 (0.73 to 1.70) | 0 | 210.47 (151.82 to 286.50) | 310.96 (213.48 to 440.46) | 1.20 (0.56 to 1.85) | 0 |
| Liberia | 62.38 (42.13 to 88.93) | 59.05 (39.76 to 84.52) | -0.20 (-0.36 to -0.04) | 0.013 | 6.71 (4.19 to 9.15) | 3.67 (2.30 to 5.19) | -2.10 (-2.84 to -1.35) | 0 | 578.48 (360.32 to 789.38) | 310.66 (193.83 to 440.41) | -2.15 (-2.89 to -1.41) | 0 |
| Libya | 98.06 (68.12 to 139.43) | 103.01 (71.72 to 143.91) | 0.16 (0.03 to 0.29) | 0.02 | 6.59 (5.04 to 8.44) | 6.22 (4.41 to 8.49) | -0.23 (-0.79 to 0.34) | 0.435 | 557.43 (425.78 to 714.89) | 528.31 (373.81 to 724.13) | -0.21 (-0.79 to 0.38) | 0.485 |
| Lithuania | 465.79 (311.96 to 661.56) | 462.23 (308.77 to 657.31) | -0.03 (-0.19 to 0.13) | 0.701 | 4.85 (4.38 to 5.38) | 2.71 (2.29 to 3.21) | -1.90 (-3.12 to -0.66) | 0.003 | 409.61 (369.16 to 454.37) | 228.53 (192.25 to 271.27) | -1.90 (-3.14 to -0.65) | 0.003 |
| Luxembourg | 171.21 (118.09 to 240.69) | 166.30 (113.70 to 235.07) | -0.06 (-0.60 to 0.47) | 0.816 | 4.92 (4.37 to 5.52) | 1.79 (1.45 to 2.22) | -3.69 (-4.39 to -2.97) | 0 | 417.87 (370.95 to 469.57) | 156.67 (127.27 to 193.91) | -3.56 (-4.25 to -2.87) | 0 |
| Madagascar | 48.80 (34.81 to 66.94) | 45.91 (31.89 to 64.56) | -0.18 (-0.23 to -0.13) | 0 | 8.22 (6.13 to 10.52) | 5.41 (3.69 to 7.68) | -1.25 (-1.51 to -1.00) | 0 | 704.07 (526.97 to 901.35) | 458.17 (311.24 to 652.25) | -1.29 (-1.54 to -1.03) | 0 |
| Malawi | 58.61 (44.07 to 78.48) | 51.88 (35.35 to 73.90) | -0.37 (-0.54 to -0.20) | 0 | 15.17 (11.40 to 19.43) | 8.75 (4.51 to 15.18) | -1.69 (-2.14 to -1.23) | 0 | 1310.90 (987.86 to 1677.41) | 749.80 (383.67 to 1303.24) | -1.71 (-2.17 to -1.25) | 0 |
| Malaysia | 137.13 (90.93 to 197.89) | 136.14 (90.20 to 197.14) | -0.03 (-0.07 to 0.01) | 0.167 | 4.49 (2.99 to 6.21) | 2.54 (1.75 to 3.35) | -1.95 (-2.87 to -1.03) | 0 | 371.82 (246.70 to 514.82) | 210.70 (145.37 to 279.03) | -1.93 (-2.85 to -1.01) | 0 |
| Maldives | 138.18 (91.81 to 198.37) | 138.27 (92.11 to 199.14) | 0.00 (-0.01 to 0.02) | 0.776 | 5.25 (2.80 to 8.31) | 2.60 (1.85 to 3.52) | -2.28 (-2.53 to -2.03) | 0 | 440.10 (233.30 to 700.91) | 221.10 (156.94 to 300.84) | -2.23 (-2.48 to -1.98) | 0 |
| Mali | 64.43 (44.78 to 89.84) | 61.50 (41.57 to 87.51) | -0.15 (-0.24 to -0.06) | 0.001 | 7.37 (5.06 to 9.98) | 4.20 (2.50 to 6.81) | -1.76 (-2.45 to -1.07) | 0 | 638.42 (439.02 to 863.29) | 361.30 (214.48 to 586.54) | -1.78 (-2.47 to -1.09) | 0 |
| Malta | 128.40 (89.85 to 177.40) | 146.96 (106.35 to 196.55) | 0.55 (0.28 to 0.83) | 0 | 3.54 (2.68 to 4.11) | 4.11 (3.28 to 5.00) | 0.81 (-0.13 to 1.76) | 0.093 | 303.72 (228.97 to 351.97) | 355.22 (284.57 to 432.43) | 0.87 (-0.02 to 1.76) | 0.055 |
| Marshall Islands | 145.68 (96.81 to 213.09) | 147.78 (98.89 to 215.66) | 0.05 (0.04 to 0.05) | 0 | 2.04 (1.36 to 2.93) | 2.56 (1.61 to 3.76) | 0.72 (0.50 to 0.94) | 0 | 169.76 (113.19 to 244.67) | 212.86 (133.85 to 313.93) | 0.72 (0.50 to 0.94) | 0 |
| Mauritania | 59.21 (39.51 to 84.62) | 58.92 (39.40 to 84.58) | -0.02 (-0.04 to 0.01) | 0.192 | 4.27 (2.93 to 6.24) | 3.00 (1.94 to 4.30) | -1.13 (-1.40 to -0.85) | 0 | 365.44 (250.21 to 537.73) | 255.49 (164.64 to 366.67) | -1.15 (-1.43 to -0.86) | 0 |
| Mauritius | 136.16 (89.87 to 197.80) | 135.43 (89.18 to 196.76) | -0.01 (-0.07 to 0.06) | 0.809 | 2.83 (2.55 to 3.15) | 1.57 (1.33 to 1.81) | -1.71 (-2.23 to -1.19) | 0 | 236.70 (212.88 to 263.75) | 131.87 (111.51 to 152.76) | -1.68 (-2.19 to -1.16) | 0 |
| Mexico | 137.10 (94.51 to 192.63) | 124.64 (87.42 to 173.70) | -0.32 (-0.40 to -0.25) | 0 | 8.37 (7.67 to 9.27) | 4.48 (3.72 to 5.42) | -2.12 (-2.51 to -1.74) | 0 | 709.44 (649.33 to 787.14) | 375.58 (310.25 to 456.41) | -2.16 (-2.55 to -1.76) | 0 |
| Micronesia (Federated States of) | 146.50 (97.94 to 213.42) | 147.19 (98.42 to 214.82) | 0.01 (0.00 to 0.03) | 0.03 | 3.13 (2.15 to 4.36) | 2.20 (1.44 to 3.27) | -1.16 (-1.49 to -0.83) | 0 | 261.46 (179.54 to 364.02) | 182.98 (119.67 to 271.69) | -1.18 (-1.51 to -0.85) | 0 |
| Monaco | 179.82 (127.57 to 249.95) | 187.62 (134.43 to 257.81) | 0.24 (0.15 to 0.33) | 0 | 5.29 (3.75 to 7.11) | 4.94 (3.94 to 6.07) | 0.03 (-0.79 to 0.86) | 0.939 | 464.28 (325.69 to 628.89) | 437.77 (345.25 to 540.48) | 0.06 (-0.78 to 0.91) | 0.886 |
| Mongolia | 392.05 (263.85 to 554.94) | 385.01 (257.38 to 548.29) | -0.06 (-0.08 to -0.03) | 0 | 10.83 (7.76 to 14.57) | 4.55 (3.48 to 5.80) | -2.65 (-3.52 to -1.77) | 0 | 926.14 (662.26 to 1244.36) | 380.45 (290.45 to 486.05) | -2.73 (-3.57 to -1.88) | 0 |
| Montenegro | 504.04 (342.43 to 702.56) | 495.24 (333.91 to 693.10) | -0.05 (-0.11 to 0.01) | 0.096 | 5.93 (4.76 to 7.25) | 2.51 (1.67 to 3.45) | -2.55 (-4.85 to -0.19) | 0.034 | 501.61 (401.03 to 614.87) | 209.37 (138.20 to 288.84) | -2.58 (-4.82 to -0.29) | 0.028 |
| Morocco | 91.18 (61.48 to 132.06) | 90.24 (60.64 to 130.67) | -0.04 (-0.06 to -0.01) | 0.002 | 3.69 (2.51 to 5.08) | 2.07 (1.42 to 2.99) | -1.90 (-2.18 to -1.62) | 0 | 313.17 (212.08 to 430.44) | 175.39 (120.26 to 254.65) | -1.90 (-2.19 to -1.62) | 0 |
| Mozambique | 52.64 (38.00 to 72.29) | 47.42 (32.75 to 68.11) | -0.32 (-0.47 to -0.17) | 0 | 11.48 (7.91 to 15.68) | 6.70 (3.73 to 11.54) | -1.71 (-2.43 to -0.98) | 0 | 996.65 (687.25 to 1358.43) | 577.00 (320.09 to 999.11) | -1.73 (-2.46 to -1.00) | 0 |
| Myanmar | 142.72 (95.67 to 204.18) | 139.68 (94.20 to 200.23) | -0.07 (-0.09 to -0.05) | 0 | 9.18 (4.01 to 15.22) | 5.49 (3.70 to 7.92) | -1.64 (-1.90 to -1.38) | 0 | 775.54 (334.67 to 1290.80) | 462.33 (310.46 to 669.42) | -1.65 (-1.92 to -1.39) | 0 |
| Namibia | 148.67 (98.64 to 214.21) | 151.61 (102.09 to 217.16) | 0.07 (0.05 to 0.09) | 0 | 3.67 (2.52 to 5.04) | 4.46 (3.03 to 6.58) | 0.65 (0.20 to 1.10) | 0.005 | 310.41 (213.17 to 428.00) | 378.55 (256.87 to 559.29) | 0.66 (0.21 to 1.12) | 0.004 |
| Nauru | 148.61 (99.86 to 215.63) | 149.52 (100.59 to 216.61) | 0.02 (0.01 to 0.03) | 0 | 3.76 (2.74 to 5.03) | 3.76 (2.58 to 5.23) | 0.01 (-0.16 to 0.18) | 0.877 | 315.58 (229.92 to 422.11) | 316.06 (216.44 to 439.98) | 0.01 (-0.16 to 0.19) | 0.867 |
| Nepal | 94.45 (64.09 to 131.47) | 113.34 (79.96 to 157.66) | 0.57 (0.50 to 0.63) | 0 | 6.63 (3.85 to 9.80) | 3.55 (2.56 to 4.90) | -1.97 (-2.38 to -1.57) | 0 | 561.89 (324.65 to 832.50) | 296.85 (214.25 to 411.12) | -2.02 (-2.43 to -1.61) | 0 |
| Netherlands | 160.97 (112.06 to 223.50) | 156.64 (107.30 to 218.51) | -0.09 (-0.11 to -0.07) | 0 | 3.83 (3.51 to 4.12) | 1.92 (1.68 to 2.20) | -2.36 (-3.03 to -1.69) | 0 | 327.87 (300.90 to 353.73) | 166.82 (145.32 to 191.26) | -2.32 (-3.11 to -1.52) | 0 |
| New Zealand | 97.56 (67.02 to 135.72) | 82.24 (59.94 to 109.83) | -0.53 (-0.68 to -0.38) | 0 | 4.08 (3.67 to 4.54) | 2.06 (1.79 to 2.36) | -2.09 (-3.23 to -0.93) | 0 | 345.59 (310.69 to 384.56) | 177.43 (154.74 to 203.30) | -2.17 (-3.09 to -1.24) | 0 |
| Nicaragua | 119.15 (83.40 to 166.34) | 113.71 (77.76 to 160.62) | -0.16 (-0.18 to -0.14) | 0 | 8.64 (6.79 to 10.96) | 3.28 (2.45 to 4.37) | -3.22 (-3.41 to -3.02) | 0 | 734.80 (576.76 to 931.76) | 275.93 (206.22 to 369.89) | -3.24 (-3.44 to -3.05) | 0 |
| Niger | 62.89 (43.29 to 88.73) | 58.30 (38.95 to 83.68) | -0.26 (-0.32 to -0.19) | 0 | 7.19 (4.95 to 9.69) | 3.51 (2.06 to 5.38) | -2.36 (-2.90 to -1.82) | 0 | 620.57 (427.08 to 836.96) | 299.43 (175.06 to 458.77) | -2.40 (-2.94 to -1.86) | 0 |
| Nigeria | 68.57 (47.88 to 95.93) | 67.79 (46.37 to 95.55) | -0.04 (-0.06 to -0.01) | 0.004 | 7.24 (5.42 to 9.34) | 5.91 (3.30 to 8.59) | -0.67 (-0.84 to -0.49) | 0 | 621.07 (466.35 to 799.46) | 505.67 (281.35 to 734.46) | -0.67 (-0.85 to -0.49) | 0 |
| Niue | 147.69 (98.69 to 215.05) | 179.05 (127.66 to 248.45) | 0.69 (0.56 to 0.82) | 0 | 3.00 (2.06 to 4.26) | 13.57 (10.24 to 17.97) | 4.88 (2.97 to 6.84) | 0 | 252.43 (173.06 to 358.88) | 1149.05 (867.15 to 1522.86) | 4.90 (2.97 to 6.85) | 0 |
| North Macedonia | 502.49 (341.71 to 700.66) | 497.09 (335.89 to 695.03) | -0.04 (-0.06 to -0.02) | 0 | 8.24 (6.64 to 10.02) | 3.56 (2.64 to 4.65) | -2.80 (-3.85 to -1.74) | 0 | 699.78 (562.64 to 851.59) | 297.63 (219.83 to 391.24) | -2.83 (-3.87 to -1.77) | 0 |
| Northern Mariana Islands | 148.87 (99.12 to 217.60) | 146.86 (98.13 to 213.82) | -0.05 (-0.13 to 0.04) | 0.295 | 1.56 (1.11 to 2.17) | 1.28 (0.94 to 1.71) | -0.60 (-1.07 to -0.13) | 0.012 | 129.87 (92.51 to 180.75) | 107.63 (78.97 to 143.72) | -0.50 (-1.03 to 0.03) | 0.067 |
| Norway | 208.39 (143.48 to 291.58) | 205.14 (140.37 to 288.43) | -0.05 (-0.24 to 0.14) | 0.603 | 3.99 (3.76 to 4.22) | 1.96 (1.77 to 2.18) | -2.05 (-3.69 to -0.38) | 0.016 | 341.70 (321.53 to 362.13) | 168.98 (152.78 to 188.30) | -2.30 (-3.60 to -0.98) | 0.001 |
| Oman | 90.52 (60.85 to 130.45) | 90.95 (61.27 to 131.45) | 0.00 (-0.05 to 0.06) | 0.917 | 3.12 (2.24 to 4.26) | 1.54 (1.16 to 1.94) | -2.43 (-2.83 to -2.03) | 0 | 262.23 (188.81 to 357.86) | 130.42 (98.14 to 164.65) | -2.40 (-2.79 to -2.01) | 0 |
| Pakistan | 141.43 (95.92 to 200.96) | 144.14 (98.99 to 204.02) | 0.06 (0.04 to 0.09) | 0 | 7.53 (5.21 to 10.13) | 7.94 (6.13 to 10.20) | 0.20 (-0.08 to 0.48) | 0.17 | 637.69 (440.55 to 859.14) | 669.19 (516.52 to 861.64) | 0.19 (-0.09 to 0.46) | 0.189 |
| Palau | 146.41 (97.83 to 213.84) | 146.49 (97.66 to 214.00) | 0.00 (-0.01 to 0.01) | 0.813 | 1.94 (1.35 to 2.71) | 1.34 (0.97 to 1.91) | -1.20 (-1.50 to -0.90) | 0 | 164.66 (114.72 to 229.96) | 114.18 (82.69 to 161.87) | -1.18 (-1.47 to -0.90) | 0 |
| Palestine | 96.38 (66.51 to 136.91) | 96.71 (67.13 to 136.18) | 0.03 (-0.06 to 0.12) | 0.5 | 6.42 (4.48 to 8.74) | 3.98 (3.06 to 5.13) | -1.35 (-2.17 to -0.51) | 0.002 | 541.31 (376.07 to 738.16) | 335.45 (257.55 to 433.32) | -1.34 (-2.18 to -0.49) | 0.002 |
| Panama | 119.30 (83.39 to 166.31) | 119.29 (83.66 to 166.20) | 0.01 (-0.06 to 0.07) | 0.835 | 7.84 (6.75 to 9.05) | 5.12 (4.18 to 6.23) | -1.33 (-1.90 to -0.75) | 0 | 660.28 (567.77 to 762.88) | 433.25 (352.67 to 528.19) | -1.31 (-1.87 to -0.75) | 0 |
| Papua New Guinea | 146.51 (97.56 to 212.87) | 147.15 (98.54 to 213.48) | 0.01 (-0.01 to 0.04) | 0.243 | 3.55 (1.79 to 5.64) | 3.46 (2.08 to 5.46) | -0.08 (-0.63 to 0.48) | 0.783 | 299.64 (151.28 to 477.37) | 292.28 (175.80 to 460.83) | -0.08 (-0.62 to 0.46) | 0.772 |
| Paraguay | 78.07 (53.47 to 110.75) | 78.40 (54.22 to 111.00) | 0.06 (-0.01 to 0.12) | 0.09 | 5.72 (4.54 to 7.17) | 4.43 (3.24 to 6.08) | -0.68 (-2.04 to 0.70) | 0.329 | 482.30 (382.34 to 606.58) | 371.89 (271.14 to 511.90) | -0.69 (-2.04 to 0.67) | 0.319 |
| Peru | 101.82 (71.77 to 141.22) | 99.80 (69.73 to 140.27) | -0.06 (-0.10 to -0.03) | 0 | 9.87 (7.91 to 12.77) | 5.30 (3.68 to 7.23) | -1.98 (-2.55 to -1.40) | 0 | 834.43 (667.76 to 1083.25) | 442.58 (306.03 to 605.41) | -2.02 (-2.57 to -1.47) | 0 |
| Philippines | 127.60 (86.86 to 178.99) | 81.42 (56.53 to 113.97) | -1.42 (-1.52 to -1.32) | 0 | 7.04 (5.08 to 9.49) | 4.68 (3.71 to 5.73) | -1.29 (-1.55 to -1.02) | 0 | 592.13 (424.46 to 801.48) | 391.96 (309.77 to 481.15) | -1.30 (-1.57 to -1.03) | 0 |
| Poland | 863.36 (581.71 to 1232.01) | 957.06 (725.62 to 1216.05) | 0.32 (0.20 to 0.44) | 0 | 5.80 (5.39 to 6.12) | 2.04 (1.80 to 2.29) | -3.12 (-3.91 to -2.33) | 0 | 489.99 (455.07 to 517.67) | 172.73 (151.57 to 194.83) | -3.13 (-3.90 to -2.34) | 0 |
| Portugal | 145.66 (103.04 to 200.13) | 169.97 (122.22 to 232.83) | 0.50 (0.40 to 0.61) | 0 | 6.79 (6.17 to 7.42) | 2.57 (2.22 to 2.98) | -3.35 (-4.07 to -2.63) | 0 | 573.77 (521.18 to 627.61) | 222.25 (191.94 to 258.08) | -3.26 (-3.99 to -2.54) | 0 |
| Puerto Rico | 93.63 (64.49 to 131.37) | 91.66 (62.32 to 129.56) | -0.05 (-0.18 to 0.07) | 0.412 | 3.80 (3.40 to 4.23) | 1.43 (1.20 to 1.72) | -2.97 (-4.52 to -1.41) | 0 | 320.03 (286.23 to 357.10) | 123.65 (103.13 to 148.21) | -2.89 (-4.42 to -1.33) | 0 |
| Qatar | 102.74 (68.99 to 148.29) | 104.23 (70.31 to 150.51) | 0.04 (-0.01 to 0.08) | 0.097 | 2.84 (2.15 to 3.78) | 1.29 (0.96 to 1.74) | -2.50 (-3.09 to -1.91) | 0 | 236.99 (179.57 to 316.78) | 110.02 (81.14 to 148.49) | -2.43 (-3.01 to -1.85) | 0 |
| Republic of Korea | 403.85 (270.82 to 584.29) | 408.58 (273.99 to 589.17) | 0.04 (0.01 to 0.07) | 0.009 | 5.99 (4.67 to 7.29) | 1.95 (1.37 to 2.47) | -3.51 (-3.88 to -3.14) | 0 | 499.99 (389.04 to 609.31) | 170.11 (119.76 to 216.00) | -3.36 (-3.73 to -2.99) | 0 |
| Republic of Moldova | 445.78 (302.10 to 633.36) | 434.29 (291.64 to 621.45) | -0.09 (-0.11 to -0.06) | 0 | 10.45 (9.15 to 11.99) | 3.53 (2.82 to 4.40) | -3.55 (-4.38 to -2.71) | 0 | 889.52 (778.66 to 1022.83) | 301.76 (240.32 to 377.64) | -3.53 (-4.35 to -2.70) | 0 |
| Romania | 561.10 (384.23 to 789.20) | 554.91 (378.39 to 783.16) | -0.04 (-0.07 to -0.00) | 0.039 | 8.13 (7.19 to 9.06) | 2.87 (2.46 to 3.32) | -3.21 (-3.87 to -2.55) | 0 | 688.90 (609.11 to 767.38) | 242.57 (208.21 to 281.76) | -3.21 (-3.86 to -2.55) | 0 |
| Russian Federation | 484.75 (322.01 to 684.69) | 475.24 (312.55 to 674.56) | -0.06 (-0.10 to -0.03) | 0 | 7.54 (7.29 to 7.81) | 2.58 (2.34 to 2.81) | -3.61 (-4.85 to -2.36) | 0 | 638.66 (616.90 to 662.02) | 219.71 (198.89 to 240.19) | -3.60 (-4.88 to -2.30) | 0 |
| Rwanda | 53.34 (39.21 to 72.37) | 47.62 (33.46 to 66.72) | -0.34 (-0.70 to 0.03) | 0.071 | 12.11 (9.08 to 15.52) | 6.28 (4.27 to 9.00) | -2.00 (-2.98 to -1.02) | 0 | 1030.66 (771.46 to 1320.60) | 533.40 (361.58 to 765.66) | -1.99 (-3.15 to -0.81) | 0.001 |
| Saint Kitts and Nevis | 93.74 (64.53 to 131.79) | 94.60 (65.21 to 132.12) | 0.03 (-0.05 to 0.12) | 0.425 | 5.63 (4.86 to 6.46) | 4.26 (3.45 to 5.21) | -0.89 (-1.47 to -0.32) | 0.002 | 471.39 (406.38 to 542.19) | 357.36 (288.61 to 439.48) | -0.89 (-1.45 to -0.33) | 0.002 |
| Saint Lucia | 93.85 (64.17 to 131.72) | 95.02 (65.82 to 132.42) | 0.04 (-0.01 to 0.10) | 0.123 | 4.90 (4.05 to 5.81) | 4.03 (3.19 to 5.10) | -0.62 (-1.21 to -0.04) | 0.038 | 413.93 (341.37 to 491.84) | 337.50 (266.39 to 428.78) | -0.65 (-1.23 to -0.07) | 0.027 |
| Saint Vincent and the Grenadines | 97.51 (68.47 to 135.58) | 98.18 (69.08 to 135.86) | 0.02 (-0.15 to 0.20) | 0.778 | 6.93 (5.69 to 8.53) | 5.64 (4.64 to 6.83) | -0.60 (-2.11 to 0.93) | 0.44 | 583.64 (477.95 to 720.54) | 468.44 (384.64 to 568.72) | -0.65 (-2.16 to 0.89) | 0.405 |
| Samoa | 148.45 (100.20 to 214.94) | 150.07 (100.74 to 217.66) | 0.03 (0.02 to 0.05) | 0 | 4.04 (2.70 to 5.58) | 2.98 (1.86 to 4.66) | -0.97 (-1.30 to -0.63) | 0 | 340.00 (226.43 to 469.45) | 250.71 (155.63 to 393.04) | -0.98 (-1.32 to -0.63) | 0 |
| San Marino | 200.77 (143.29 to 268.83) | 171.56 (121.95 to 237.63) | -0.50 (-0.66 to -0.34) | 0 | 7.00 (5.27 to 9.15) | 3.06 (2.19 to 4.12) | -2.67 (-2.98 to -2.36) | 0 | 619.68 (465.91 to 809.57) | 272.38 (193.10 to 368.69) | -2.61 (-2.92 to -2.31) | 0 |
| Sao Tome and Principe | 61.06 (41.87 to 86.53) | 57.60 (38.17 to 83.09) | -0.19 (-0.30 to -0.08) | 0 | 5.26 (3.73 to 7.16) | 2.01 (1.10 to 3.26) | -3.06 (-4.42 to -1.68) | 0 | 450.26 (319.24 to 613.25) | 170.16 (92.97 to 276.17) | -3.09 (-4.50 to -1.67) | 0 |
| Saudi Arabia | 91.52 (62.02 to 131.32) | 88.89 (59.55 to 128.93) | -0.10 (-0.10 to -0.09) | 0 | 4.01 (2.90 to 5.46) | 1.34 (0.95 to 1.89) | -3.49 (-3.65 to -3.34) | 0 | 339.52 (245.50 to 463.17) | 112.67 (79.21 to 158.65) | -3.53 (-3.69 to -3.36) | 0 |
| Senegal | 61.14 (41.75 to 87.10) | 58.34 (39.06 to 83.85) | -0.17 (-0.32 to -0.02) | 0.03 | 5.48 (4.14 to 7.14) | 3.36 (2.15 to 5.11) | -1.66 (-3.49 to 0.21) | 0.081 | 470.44 (356.11 to 612.69) | 285.03 (182.03 to 435.38) | -1.70 (-3.56 to 0.19) | 0.078 |
| Serbia | 491.28 (333.79 to 685.63) | 470.47 (317.09 to 662.14) | -0.15 (-0.23 to -0.06) | 0.001 | 6.86 (5.10 to 8.82) | 1.93 (1.49 to 2.50) | -3.98 (-5.22 to -2.73) | 0 | 579.72 (429.98 to 747.21) | 162.85 (125.72 to 211.52) | -3.98 (-5.23 to -2.70) | 0 |
| Seychelles | 136.61 (90.55 to 198.53) | 135.36 (89.48 to 197.22) | -0.03 (-0.10 to 0.04) | 0.365 | 3.45 (2.61 to 4.47) | 1.74 (1.29 to 2.31) | -2.48 (-4.53 to -0.38) | 0.021 | 287.74 (218.25 to 373.10) | 149.37 (110.66 to 197.64) | -2.39 (-4.38 to -0.36) | 0.021 |
| Sierra Leone | 62.30 (42.66 to 87.75) | 59.91 (40.41 to 85.41) | -0.11 (-0.21 to -0.01) | 0.025 | 6.61 (4.57 to 8.85) | 4.36 (2.92 to 6.14) | -1.26 (-2.01 to -0.51) | 0.001 | 571.55 (395.75 to 764.32) | 372.03 (249.54 to 524.34) | -1.30 (-2.06 to -0.55) | 0.001 |
| Singapore | 403.82 (270.85 to 584.82) | 416.67 (280.59 to 600.28) | 0.11 (0.05 to 0.16) | 0 | 4.29 (3.85 to 4.78) | 1.98 (1.69 to 2.28) | -2.48 (-3.66 to -1.28) | 0 | 363.56 (325.76 to 406.65) | 172.25 (148.74 to 199.15) | -2.37 (-3.53 to -1.20) | 0 |
| Slovakia | 479.66 (321.76 to 677.62) | 479.16 (320.62 to 677.01) | -0.00 (-0.09 to 0.08) | 0.94 | 4.27 (3.68 to 4.97) | 2.60 (2.00 to 3.32) | -1.37 (-1.56 to -1.17) | 0 | 359.38 (309.67 to 418.25) | 221.54 (170.41 to 283.28) | -1.33 (-1.52 to -1.14) | 0 |
| Slovenia | 407.50 (276.19 to 577.28) | 406.72 (275.22 to 575.60) | 0.03 (-0.41 to 0.47) | 0.897 | 3.20 (2.85 to 3.57) | 1.22 (1.00 to 1.48) | -3.06 (-4.08 to -2.04) | 0 | 270.12 (241.07 to 301.60) | 105.59 (86.79 to 127.78) | -3.10 (-4.08 to -2.12) | 0 |
| Solomon Islands | 145.46 (96.54 to 212.20) | 146.18 (97.37 to 212.98) | 0.01 (-0.01 to 0.04) | 0.154 | 2.63 (1.47 to 4.04) | 2.58 (1.54 to 3.94) | -0.14 (-0.88 to 0.62) | 0.723 | 220.27 (123.70 to 337.23) | 214.27 (128.28 to 328.21) | -0.15 (-0.92 to 0.62) | 0.697 |
| Somalia | 47.02 (32.45 to 65.77) | 44.24 (30.54 to 62.69) | -0.18 (-0.27 to -0.08) | 0 | 7.59 (4.58 to 11.06) | 4.96 (2.86 to 7.55) | -1.30 (-1.92 to -0.67) | 0 | 650.52 (391.75 to 948.75) | 417.83 (239.87 to 637.21) | -1.35 (-1.98 to -0.72) | 0 |
| South Africa | 163.79 (108.97 to 236.63) | 163.55 (109.01 to 236.40) | -0.00 (-0.02 to 0.01) | 0.624 | 3.16 (2.35 to 4.04) | 2.86 (2.31 to 3.47) | -0.25 (-0.90 to 0.39) | 0.441 | 266.98 (197.86 to 342.62) | 239.19 (192.86 to 291.65) | -0.28 (-0.96 to 0.40) | 0.413 |
| South Sudan | 50.15 (35.17 to 69.60) | 50.61 (35.95 to 70.00) | 0.06 (-0.09 to 0.21) | 0.42 | 9.37 (6.19 to 13.00) | 8.88 (5.84 to 12.39) | -0.04 (-0.59 to 0.51) | 0.877 | 808.82 (533.44 to 1123.13) | 755.62 (496.79 to 1056.25) | -0.10 (-0.68 to 0.49) | 0.743 |
| Spain | 139.69 (99.35 to 192.54) | 146.29 (112.40 to 182.28) | 0.14 (0.06 to 0.22) | 0 | 4.69 (4.34 to 5.05) | 2.49 (2.22 to 2.81) | -2.27 (-2.45 to -2.09) | 0 | 398.69 (368.27 to 430.01) | 217.04 (193.51 to 245.02) | -2.18 (-2.28 to -2.09) | 0 |
| Sri Lanka | 139.50 (93.33 to 200.68) | 136.97 (91.06 to 199.09) | -0.04 (-0.09 to -0.00) | 0.047 | 5.15 (3.93 to 6.59) | 1.98 (1.40 to 2.76) | -2.99 (-3.61 to -2.36) | 0 | 428.33 (327.66 to 547.85) | 165.26 (116.02 to 230.80) | -2.97 (-3.59 to -2.35) | 0 |
| Sudan | 98.45 (66.98 to 140.13) | 96.57 (66.44 to 136.95) | -0.06 (-0.09 to -0.04) | 0 | 10.06 (5.00 to 16.13) | 6.30 (4.22 to 8.88) | -1.52 (-1.68 to -1.36) | 0 | 856.91 (422.51 to 1375.99) | 530.63 (355.73 to 750.36) | -1.55 (-1.71 to -1.39) | 0 |
| Suriname | 95.52 (65.52 to 133.09) | 95.60 (66.24 to 133.80) | 0.01 (-0.07 to 0.09) | 0.883 | 6.54 (4.31 to 8.15) | 5.55 (4.17 to 7.13) | -0.53 (-1.30 to 0.24) | 0.177 | 552.11 (365.62 to 688.03) | 467.07 (350.71 to 600.44) | -0.54 (-1.30 to 0.22) | 0.165 |
| Sweden | 190.00 (130.72 to 264.48) | 125.88 (87.81 to 174.47) | -1.33 (-1.53 to -1.12) | 0 | 3.96 (3.59 to 4.35) | 1.84 (1.61 to 2.14) | -2.16 (-2.35 to -1.98) | 0 | 342.25 (310.34 to 377.42) | 160.23 (139.46 to 186.47) | -2.14 (-2.33 to -1.96) | 0 |
| Switzerland | 173.91 (122.25 to 243.22) | 169.80 (119.47 to 239.02) | -0.02 (-0.23 to 0.18) | 0.82 | 3.64 (3.32 to 4.01) | 2.08 (1.78 to 2.43) | -1.72 (-2.05 to -1.39) | 0 | 316.46 (288.23 to 347.99) | 183.50 (157.19 to 216.52) | -1.68 (-2.01 to -1.36) | 0 |
| Syrian Arab Republic | 95.40 (65.24 to 136.49) | 94.40 (64.39 to 134.80) | -0.03 (-0.08 to 0.02) | 0.183 | 6.93 (4.72 to 9.39) | 3.43 (2.45 to 4.69) | -2.21 (-2.68 to -1.73) | 0 | 579.86 (392.44 to 788.01) | 285.19 (202.38 to 391.91) | -2.23 (-2.71 to -1.75) | 0 |
| Taiwan (Province of China) | 186.50 (126.22 to 267.21) | 188.04 (127.87 to 268.64) | 0.03 (-0.00 to 0.07) | 0.086 | 3.68 (3.39 to 3.99) | 2.15 (1.82 to 2.45) | -1.57 (-2.26 to -0.88) | 0 | 309.56 (285.31 to 336.22) | 183.90 (155.91 to 210.96) | -1.52 (-2.20 to -0.83) | 0 |
| Tajikistan | 391.59 (262.16 to 554.65) | 388.18 (260.95 to 551.04) | -0.03 (-0.04 to -0.02) | 0 | 10.11 (7.46 to 13.56) | 7.36 (5.36 to 10.05) | -1.00 (-1.33 to -0.66) | 0 | 858.69 (629.98 to 1155.26) | 620.69 (449.91 to 849.93) | -1.02 (-1.35 to -0.68) | 0 |
| Thailand | 140.77 (94.67 to 201.11) | 139.92 (93.70 to 200.98) | -0.02 (-0.04 to 0.01) | 0.125 | 6.10 (4.55 to 7.81) | 3.24 (2.53 to 4.01) | -1.97 (-2.28 to -1.66) | 0 | 509.87 (378.60 to 653.04) | 271.92 (212.89 to 337.84) | -1.95 (-2.26 to -1.64) | 0 |
| Timor-Leste | 138.02 (92.14 to 197.82) | 136.41 (90.66 to 196.83) | -0.04 (-0.06 to -0.02) | 0 | 7.13 (3.43 to 11.57) | 4.29 (2.88 to 6.29) | -1.67 (-2.06 to -1.28) | 0 | 603.53 (286.08 to 983.70) | 360.01 (240.71 to 528.66) | -1.70 (-2.10 to -1.30) | 0 |
| Togo | 59.23 (40.07 to 84.78) | 58.05 (38.76 to 83.21) | -0.06 (-0.11 to -0.02) | 0.003 | 4.24 (3.17 to 5.61) | 3.13 (1.81 to 4.83) | -0.96 (-1.43 to -0.49) | 0 | 363.18 (271.39 to 479.32) | 265.45 (152.78 to 410.28) | -0.99 (-1.46 to -0.52) | 0 |
| Tokelau | 147.00 (98.01 to 214.27) | 185.46 (127.44 to 262.89) | 0.88 (0.82 to 0.95) | 0 | 3.02 (1.97 to 4.40) | 17.95 (10.25 to 28.31) | 7.27 (5.87 to 8.69) | 0 | 253.80 (165.47 to 371.48) | 1533.80 (869.45 to 2418.66) | 7.33 (5.88 to 8.80) | 0 |
| Tonga | 148.89 (100.35 to 215.45) | 151.51 (102.60 to 218.37) | 0.05 (0.02 to 0.09) | 0.005 | 3.07 (2.15 to 4.23) | 2.99 (2.05 to 4.48) | -0.20 (-0.82 to 0.42) | 0.526 | 261.49 (182.67 to 360.21) | 254.62 (175.12 to 384.45) | -0.20 (-0.83 to 0.42) | 0.524 |
| Trinidad and Tobago | 95.49 (66.17 to 133.31) | 94.20 (65.00 to 131.80) | -0.02 (-0.14 to 0.10) | 0.758 | 6.06 (5.17 to 6.95) | 3.82 (3.00 to 4.89) | -1.27 (-1.81 to -0.72) | 0 | 508.08 (433.02 to 582.73) | 322.50 (252.73 to 413.36) | -1.28 (-2.28 to -0.27) | 0.013 |
| Tunisia | 98.15 (68.03 to 138.36) | 95.22 (65.53 to 136.05) | -0.10 (-0.13 to -0.07) | 0 | 6.43 (4.94 to 8.20) | 2.81 (2.03 to 3.86) | -2.62 (-2.82 to -2.41) | 0 | 544.51 (417.57 to 696.19) | 238.49 (172.17 to 327.77) | -2.62 (-2.83 to -2.41) | 0 |
| Turkey | 143.66 (99.05 to 202.30) | 141.52 (96.99 to 201.09) | -0.05 (-0.07 to -0.02) | 0.001 | 10.20 (7.29 to 13.58) | 3.80 (2.96 to 4.81) | -3.13 (-3.30 to -2.96) | 0 | 864.04 (616.41 to 1150.66) | 323.24 (251.11 to 409.89) | -3.10 (-3.26 to -2.94) | 0 |
| Turkmenistan | 389.94 (262.22 to 553.69) | 386.99 (259.89 to 549.82) | -0.02 (-0.04 to -0.01) | 0.007 | 8.50 (7.28 to 9.81) | 5.92 (4.86 to 7.16) | -1.11 (-1.72 to -0.50) | 0 | 724.21 (618.47 to 837.25) | 494.90 (405.33 to 598.87) | -1.16 (-1.78 to -0.54) | 0 |
| Tuvalu | 146.22 (98.09 to 212.49) | 145.96 (97.82 to 212.69) | -0.00 (-0.02 to 0.01) | 0.591 | 3.64 (2.13 to 5.55) | 2.23 (1.52 to 3.12) | -1.54 (-1.99 to -1.09) | 0 | 306.36 (178.04 to 468.47) | 186.04 (127.28 to 260.93) | -1.56 (-2.02 to -1.10) | 0 |
| Uganda | 51.73 (37.60 to 71.21) | 51.77 (36.59 to 72.50) | -0.00 (-0.28 to 0.27) | 0.983 | 10.01 (7.12 to 14.12) | 8.91 (5.28 to 13.85) | -0.40 (-1.56 to 0.77) | 0.499 | 860.88 (613.01 to 1216.16) | 757.28 (445.94 to 1182.66) | -0.44 (-1.60 to 0.73) | 0.461 |
| Ukraine | 483.78 (322.51 to 683.36) | 475.42 (313.81 to 674.41) | -0.05 (-0.06 to -0.05) | 0 | 8.67 (7.32 to 10.11) | 3.90 (3.11 to 4.76) | -2.53 (-2.81 to -2.25) | 0 | 730.68 (615.62 to 852.86) | 330.85 (263.44 to 404.81) | -2.47 (-2.74 to -2.21) | 0 |
| United Arab Emirates | 92.03 (62.69 to 132.39) | 90.54 (60.82 to 131.18) | -0.05 (-0.11 to 0.00) | 0.067 | 4.10 (2.96 to 5.59) | 1.89 (1.36 to 2.56) | -2.48 (-3.32 to -1.63) | 0 | 340.95 (246.41 to 465.55) | 158.38 (113.39 to 216.15) | -2.46 (-3.29 to -1.62) | 0 |
| United Kingdom | 141.09 (97.88 to 196.10) | 101.37 (73.39 to 135.23) | -1.08 (-1.22 to -0.95) | 0 | 3.95 (3.83 to 4.09) | 1.96 (1.81 to 2.08) | -2.13 (-2.62 to -1.64) | 0 | 338.86 (327.85 to 350.51) | 169.35 (155.99 to 181.03) | -2.10 (-2.60 to -1.60) | 0 |
| United Republic of Tanzania | 52.57 (38.59 to 70.92) | 50.44 (36.23 to 69.98) | -0.14 (-0.20 to -0.08) | 0 | 10.74 (8.30 to 13.34) | 7.96 (5.42 to 11.22) | -1.02 (-1.28 to -0.75) | 0 | 920.68 (710.88 to 1143.91) | 679.86 (460.25 to 960.07) | -1.03 (-1.29 to -0.77) | 0 |
| United States of America | 237.38 (161.19 to 335.76) | 312.26 (218.22 to 433.68) | 0.87 (0.63 to 1.12) | 0 | 3.58 (3.51 to 3.65) | 2.14 (1.98 to 2.32) | -1.58 (-1.88 to -1.29) | 0 | 307.10 (300.10 to 314.47) | 185.05 (170.94 to 200.60) | -1.56 (-1.86 to -1.27) | 0 |
| United States Virgin Islands | 93.71 (64.38 to 131.34) | 90.34 (61.15 to 128.12) | -0.12 (-0.22 to -0.02) | 0.016 | 4.31 (3.24 to 5.54) | 1.66 (1.01 to 2.63) | -3.09 (-4.68 to -1.47) | 0 | 359.45 (269.07 to 462.65) | 143.39 (86.92 to 227.09) | -2.98 (-4.64 to -1.30) | 0.001 |
| Uruguay | 185.86 (122.81 to 268.14) | 184.10 (121.61 to 265.82) | -0.04 (-0.06 to -0.02) | 0 | 5.09 (4.55 to 5.70) | 2.85 (2.37 to 3.46) | -2.16 (-2.63 to -1.69) | 0 | 427.91 (382.20 to 479.59) | 239.06 (198.03 to 290.14) | -2.17 (-2.64 to -1.70) | 0 |
| Uzbekistan | 387.89 (260.70 to 551.14) | 387.38 (260.71 to 549.70) | -0.00 (-0.01 to 0.00) | 0.064 | 6.98 (5.77 to 8.40) | 6.02 (4.92 to 7.35) | -0.52 (-0.87 to -0.18) | 0.003 | 587.70 (484.50 to 708.64) | 505.92 (412.24 to 619.47) | -0.53 (-0.86 to -0.19) | 0.002 |
| Vanuatu | 144.67 (96.00 to 211.58) | 145.80 (97.12 to 212.84) | 0.03 (-0.01 to 0.07) | 0.197 | 2.02 (1.25 to 3.02) | 1.99 (1.23 to 2.95) | -0.03 (-1.92 to 1.90) | 0.974 | 168.58 (104.97 to 252.35) | 165.54 (102.17 to 244.89) | -0.04 (-1.96 to 1.91) | 0.967 |
| Bolivarian Republic of Venezuela | 116.92 (81.00 to 164.16) | 117.33 (81.54 to 164.58) | 0.01 (-0.04 to 0.06) | 0.59 | 6.83 (6.34 to 7.34) | 5.10 (3.85 to 6.50) | -0.40 (-1.47 to 0.70) | 0.476 | 572.01 (530.69 to 615.63) | 426.40 (321.10 to 545.53) | -0.39 (-1.48 to 0.70) | 0.481 |
| Viet Nam | 137.68 (91.65 to 198.52) | 137.68 (91.75 to 198.59) | 0.00 (-0.02 to 0.02) | 0.955 | 4.47 (3.25 to 5.90) | 2.78 (1.90 to 3.87) | -1.51 (-1.86 to -1.15) | 0 | 380.83 (276.81 to 502.68) | 235.73 (160.61 to 328.45) | -1.53 (-1.89 to -1.16) | 0 |
| Yemen | 93.36 (63.29 to 134.00) | 92.70 (63.40 to 132.44) | -0.02 (-0.05 to 0.00) | 0.063 | 6.65 (3.59 to 10.65) | 4.67 (3.10 to 6.63) | -1.18 (-1.41 to -0.94) | 0 | 563.32 (303.01 to 904.70) | 392.14 (260.44 to 556.36) | -1.18 (-1.62 to -0.73) | 0 |
| Zambia | 53.02 (38.80 to 71.57) | 48.54 (34.49 to 67.79) | -0.26 (-0.34 to -0.17) | 0 | 11.39 (8.76 to 14.36) | 6.80 (4.43 to 10.14) | -1.52 (-1.81 to -1.22) | 0 | 981.30 (754.35 to 1237.96) | 578.41 (375.75 to 864.28) | -1.55 (-1.85 to -1.26) | 0 |
| Zimbabwe | 148.37 (98.24 to 214.15) | 151.55 (102.33 to 217.42) | 0.06 (0.01 to 0.11) | 0.012 | 3.12 (2.06 to 4.19) | 5.38 (3.83 to 7.31) | 1.54 (0.12 to 2.98) | 0.034 | 264.21 (175.16 to 354.51) | 452.45 (321.61 to 615.60) | 1.52 (0.13 to 2.93) | 0.032 |

Estimates are for individuals aged 0–14 years. Rates are reported per 100000 population. UI=uncertainty interval. CI=confidence interval. AAPC=average annual percent change. DALY=disability-adjusted life year.
