# Supplementary material for: Childhood cancer burden and health inequality: A systematic analysis from the global burden of diseases study 2021
Source: PLoS One. 2026 Jan 27;21(1):e0341303. doi: 10.1371/journal.pone.0341303 (PMC12843563; doi:10.1371/journal.pone.0341303)
Supplement: S4 Table — (DOCX) [file pone.0341303.s016.docx]

**S4 Table. Burden of Different Childhood Cancers from 1990 to 2021**

| **Characteristics** | **ASIR in 1990**  **(95% UI)** | **ASIR in 2021**  **(95% UI)** | **AAPC, %**  **(95% CI)** | ***P*** | **ASMR in 1990**  **(95% UI)** | **ASMR in 2021**  **(95% UI)** | **AAPC, %**  **(95% CI)** | ***P*** | **ASDR in 1990**  **(95% UI)** | **ASDR in 2021**  **(95% UI)** | **AAPC, %**  **(95% CI)** | ***P*** |
| --- | --- | --- | --- | --- | --- | --- | --- | --- | --- | --- | --- | --- |
| **Global** | | | | | | | | | | | | |
| Acute lymphoid leukemia | 3.09 (2.36 to 4.10) | 2.35 (1.56 to 3.07) | -0.97 (-1.18 to -0.76) | 0 | 2.30 (1.65 to 3.20) | 0.94 (0.64 to 1.16) | -2.86 (-3.05 to -2.67) | 0 | 194.66 (139.16 to 271.79) | 79.20 (53.86 to 98.31) | -2.90 (-3.04 to -2.76) | 0 |
| Brain and central nervous system cancer | 1.90 (1.45 to 2.38) | 1.65 (1.33 to 2.03) | -0.52 (-0.62 to -0.42) | 0 | 1.26 (0.93 to 1.63) | 0.82 (0.64 to 1.02) | -1.44 (-1.57 to -1.31) | 0 | 106.71 (78.54 to 137.96) | 68.54 (53.58 to 85.48) | -1.46 (-1.59 to -1.33) | 0 |
| Neuroblastoma and other peripheral nervous cell tumors | 0.24 (0.18 to 0.33) | 0.29 (0.19 to 0.40) | 0.57 (0.45 to 0.68) | 0 | 0.09 (0.08 to 0.11) | 0.10 (0.07 to 0.13) | 0.26 (0.10 to 0.41) | 0.001 | 8.24 (6.84 to 9.91) | 9.02 (6.44 to 11.68) | 0.26 (0.10 to 0.41) | 0.001 |
| Non-Hodgkin lymphoma | 1.23 (0.99 to 1.44) | 1.03 (0.83 to 1.29) | -0.60 (-0.82 to -0.37) | 0 | 0.74 (0.57 to 0.90) | 0.45 (0.35 to 0.58) | -1.60 (-1.73 to -1.48) | 0 | 63.09 (47.81 to 76.05) | 38.09 (29.59 to 48.87) | -1.62 (-1.75 to -1.48) | 0 |
| Hodgkin lymphoma | 0.25 (0.16 to 0.31) | 0.21 (0.12 to 0.28) | -0.59 (-0.78 to -0.40) | 0 | 0.15 (0.08 to 0.20) | 0.09 (0.05 to 0.13) | -1.56 (-1.74 to -1.38) | 0 | 12.55 (6.72 to 16.86) | 7.63 (3.94 to 10.92) | -1.57 (-1.75 to -1.39) | 0 |
| Soft tissue and other extraosseous sarcomas | 0.62 (0.47 to 0.86) | 0.38 (0.28 to 0.55) | -1.62 (-1.83 to -1.40) | 0 | 0.29 (0.22 to 0.40) | 0.15 (0.11 to 0.22) | -2.10 (-2.24 to -1.96) | 0 | 24.71 (19.08 to 34.50) | 12.70 (9.32 to 18.82) | -2.12 (-2.26 to -1.98) | 0 |
| Kidney cancer | 0.59 (0.48 to 0.71) | 0.49 (0.38 to 0.61) | -0.66 (-0.85 to -0.47) | 0 | 0.23 (0.19 to 0.29) | 0.16 (0.12 to 0.20) | -1.28 (-1.47 to -1.09) | 0 | 20.56 (16.20 to 24.97) | 13.92 (10.08 to 17.87) | -1.28 (-1.47 to -1.08) | 0 |
| Liver cancer | 0.43 (0.35 to 0.50) | 0.23 (0.18 to 0.28) | -1.96 (-2.10 to -1.82) | 0 | 0.30 (0.25 to 0.36) | 0.14 (0.11 to 0.18) | -2.37 (-2.52 to -2.23) | 0 | 26.54 (21.66 to 31.07) | 12.57 (10.01 to 15.78) | -2.38 (-2.52 to -2.23) | 0 |
| Malignant neoplasm of bone and articular cartilage | 0.57 (0.44 to 0.71) | 0.59 (0.48 to 0.73) | 0.13 (-0.05 to 0.31) | 0.151 | 0.22 (0.17 to 0.27) | 0.20 (0.16 to 0.24) | -0.38 (-0.53 to -0.23) | 0 | 18.04 (13.97 to 22.64) | 16.02 (13.09 to 19.71) | -0.38 (-0.52 to -0.24) | 0 |
| Thyroid cancer | 0.09 (0.07 to 0.10) | 0.10 (0.08 to 0.12) | 0.44 (0.26 to 0.62) | 0 | 0.01 (0.01 to 0.01) | 0.01 (0.01 to 0.01) | -1.04 (-1.19 to -0.90) | 0 | 0.84 (0.70 to 0.98) | 0.62 (0.48 to 0.76) | -0.96 (-1.10 to -0.81) | 0 |
| Eye cancer | 0.30 (0.19 to 0.40) | 0.37 (0.22 to 0.51) | 0.58 (0.41 to 0.76) | 0 | 0.18 (0.10 to 0.24) | 0.15 (0.09 to 0.20) | -0.67 (-0.83 to -0.51) | 0 | 15.99 (9.05 to 21.10) | 13.00 (7.87 to 17.83) | -0.66 (-0.82 to -0.51) | 0 |
| Acute myeloid leukemia | 0.84 (0.48 to 1.53) | 0.42 (0.30 to 0.57) | -2.27 (-2.36 to -2.18) | 0 | 0.80 (0.45 to 1.48) | 0.38 (0.27 to 0.52) | -2.43 (-2.52 to -2.34) | 0 | 68.06 (38.06 to 125.99) | 31.95 (22.59 to 43.84) | -2.46 (-2.56 to -2.37) | 0 |
| Chronic myeloid leukemia | 0.14 (0.07 to 0.24) | 0.04 (0.02 to 0.06) | -3.94 (-4.09 to -3.80) | 0 | 0.12 (0.06 to 0.22) | 0.03 (0.02 to 0.04) | -4.57 (-4.77 to -4.37) | 0 | 10.20 (4.95 to 18.92) | 2.36 (1.27 to 3.52) | -4.61 (-4.75 to -4.48) | 0 |
| Nasopharynx cancer | 0.07 (0.06 to 0.08) | 0.05 (0.04 to 0.06) | -1.49 (-1.72 to -1.27) | 0 | 0.05 (0.05 to 0.06) | 0.03 (0.02 to 0.03) | -2.30 (-2.58 to -2.02) | 0 | 4.25 (3.64 to 4.91) | 2.07 (1.65 to 2.56) | -2.31 (-2.59 to -2.03) | 0 |
| Other malignant neoplasms | 1.41 (0.86 to 1.79) | 0.98 (0.75 to 1.22) | -1.19 (-1.32 to -1.07) | 0 | 0.91 (0.54 to 1.18) | 0.48 (0.36 to 0.59) | -2.07 (-2.19 to -1.95) | 0 | 77.81 (46.19 to 101.43) | 40.97 (30.70 to 50.63) | -2.09 (-2.21 to -1.96) | 0 |
| Other leukemia | 0.29 (0.14 to 0.51) | 0.17 (0.08 to 0.29) | -1.68 (-1.84 to -1.52) | 0 | 0.11 (0.05 to 0.21) | 0.04 (0.02 to 0.07) | -3.14 (-3.29 to -2.99) | 0 | 9.74 (4.54 to 17.95) | 3.67 (1.76 to 6.25) | -3.14 (-3.29 to -2.99) | 0 |
| **High SDI** | | | | | | | | | | | | |
| Acute lymphoid leukemia | 4.34 (4.01 to 4.71) | 4.08 (3.55 to 4.64) | -0.24 (-0.95 to 0.48) | 0.519 | 0.94 (0.88 to 1.03) | 0.32 (0.28 to 0.36) | -3.49 (-3.63 to -3.35) | 0 | 80.35 (74.87 to 87.68) | 29.16 (25.64 to 32.92) | -3.25 (-3.37 to -3.12) | 0 |
| Brain and central nervous system cancer | 2.80 (2.66 to 2.96) | 2.74 (2.51 to 2.99) | -0.09 (-0.29 to 0.10) | 0.351 | 1.08 (1.03 to 1.13) | 0.72 (0.67 to 0.77) | -1.35 (-1.56 to -1.13) | 0 | 91.01 (86.92 to 95.02) | 60.47 (56.19 to 65.05) | -1.34 (-1.56 to -1.12) | 0 |
| Neuroblastoma and other peripheral nervous cell tumors | 0.64 (0.53 to 0.76) | 0.50 (0.40 to 0.60) | -0.87 (-1.07 to -0.66) | 0 | 0.19 (0.18 to 0.21) | 0.13 (0.12 to 0.15) | -1.24 (-1.43 to -1.04) | 0 | 17.04 (15.94 to 18.15) | 11.78 (10.27 to 13.27) | -1.25 (-1.45 to -1.05) | 0 |
| Non-Hodgkin lymphoma | 1.41 (1.31 to 1.53) | 1.28 (1.13 to 1.44) | -0.35 (-0.73 to 0.04) | 0.075 | 0.27 (0.25 to 0.29) | 0.10 (0.09 to 0.11) | -3.09 (-3.36 to -2.82) | 0 | 22.96 (21.58 to 24.42) | 9.19 (8.42 to 10.02) | -2.97 (-3.25 to -2.69) | 0 |
| Hodgkin lymphoma | 0.23 (0.22 to 0.25) | 0.13 (0.11 to 0.15) | -1.91 (-2.13 to -1.68) | 0 | 0.03 (0.02 to 0.03) | 0.01 (0.01 to 0.01) | -4.81 (-4.86 to -4.75) | 0 | 2.39 (2.10 to 2.72) | 0.55 (0.47 to 0.67) | -4.57 (-4.63 to -4.52) | 0 |
| Soft tissue and other extraosseous sarcomas | 0.55 (0.50 to 0.60) | 0.45 (0.40 to 0.50) | -0.71 (-1.25 to -0.16) | 0.011 | 0.15 (0.14 to 0.17) | 0.10 (0.09 to 0.11) | -1.36 (-1.93 to -0.80) | 0 | 13.04 (12.23 to 13.99) | 8.71 (7.91 to 9.58) | -1.37 (-1.93 to -0.80) | 0 |
| Kidney cancer | 0.65 (0.62 to 0.68) | 0.61 (0.56 to 0.66) | -0.27 (-0.71 to 0.17) | 0.223 | 0.13 (0.13 to 0.14) | 0.07 (0.07 to 0.08) | -2.00 (-2.39 to -1.61) | 0 | 11.77 (11.20 to 12.36) | 6.50 (5.98 to 7.01) | -1.96 (-2.32 to -1.60) | 0 |
| Liver cancer | 0.20 (0.19 to 0.22) | 0.23 (0.21 to 0.26) | 0.65 (0.44 to 0.85) | 0 | 0.10 (0.09 to 0.11) | 0.06 (0.06 to 0.07) | -1.32 (-1.72 to -0.92) | 0 | 8.55 (7.68 to 9.67) | 5.60 (5.12 to 6.07) | -1.27 (-1.67 to -0.87) | 0 |
| Malignant neoplasm of bone and articular cartilage | 0.62 (0.57 to 0.69) | 0.50 (0.45 to 0.55) | -0.78 (-1.09 to -0.47) | 0 | 0.15 (0.14 to 0.16) | 0.10 (0.09 to 0.11) | -1.47 (-1.82 to -1.11) | 0 | 12.51 (11.79 to 13.33) | 8.06 (7.51 to 8.67) | -1.45 (-1.79 to -1.12) | 0 |
| Thyroid cancer | 0.15 (0.14 to 0.16) | 0.11 (0.10 to 0.12) | -0.98 (-1.45 to -0.50) | 0 | 0.01 (0.01 to 0.01) | 0.00 (0.00 to 0.00) | -2.76 (-3.19 to -2.33) | 0 | 0.61 (0.57 to 0.66) | 0.28 (0.25 to 0.32) | -2.49 (-3.03 to -1.94) | 0 |
| Eye cancer | 0.51 (0.39 to 0.66) | 0.45 (0.32 to 0.60) | -0.34 (-0.78 to 0.12) | 0.144 | 0.02 (0.02 to 0.03) | 0.01 (0.01 to 0.01) | -3.31 (-4.01 to -2.62) | 0 | 2.36 (1.95 to 2.84) | 1.00 (0.79 to 1.26) | -2.81 (-3.57 to -2.05) | 0 |
| Acute myeloid leukemia | 0.60 (0.52 to 0.67) | 0.38 (0.33 to 0.41) | -1.55 (-1.83 to -1.27) | 0 | 0.49 (0.43 to 0.56) | 0.26 (0.23 to 0.29) | -2.15 (-2.44 to -1.85) | 0 | 41.52 (35.78 to 47.24) | 21.78 (19.00 to 24.00) | -2.16 (-2.43 to -1.88) | 0 |
| Chronic myeloid leukemia | 0.14 (0.13 to 0.16) | 0.05 (0.04 to 0.05) | -3.65 (-3.92 to -3.38) | 0 | 0.07 (0.06 to 0.08) | 0.01 (0.01 to 0.01) | -6.11 (-6.37 to -5.85) | 0 | 6.00 (5.16 to 7.05) | 0.87 (0.70 to 1.06) | -6.09 (-6.36 to -5.83) | 0 |
| Nasopharynx cancer | 0.04 (0.04 to 0.04) | 0.02 (0.02 to 0.03) | -1.73 (-1.84 to -1.63) | 0 | 0.01 (0.01 to 0.01) | 0.00 (0.00 to 0.00) | -3.83 (-4.03 to -3.62) | 0 | 1.07 (0.98 to 1.18) | 0.33 (0.29 to 0.38) | -3.78 (-3.98 to -3.57) | 0 |
| Other malignant neoplasms | 1.22 (1.14 to 1.30) | 1.09 (0.94 to 1.24) | -0.38 (-0.76 to -0.00) | 0.049 | 0.38 (0.35 to 0.40) | 0.16 (0.14 to 0.17) | -2.87 (-3.21 to -2.53) | 0 | 32.30 (29.77 to 34.61) | 13.58 (11.93 to 15.32) | -2.82 (-3.18 to -2.46) | 0 |
| Other leukemia | 0.07 (0.05 to 0.08) | 0.05 (0.03 to 0.07) | -0.85 (-1.09 to -0.62) | 0 | 0.01 (0.01 to 0.02) | 0.00 (0.00 to 0.01) | -2.98 (-3.23 to -2.74) | 0 | 1.05 (0.76 to 1.51) | 0.42 (0.26 to 0.61) | -2.92 (-3.15 to -2.68) | 0 |
| **High-middle SDI** | | | | | | | | | | | | |
| Acute lymphoid leukemia | 4.71 (3.61 to 5.94) | 5.70 (3.25 to 8.38) | 0.49 (0.12 to 0.85) | 0.009 | 3.12 (2.31 to 3.99) | 0.80 (0.52 to 1.04) | -4.41 (-4.90 to -3.92) | 0 | 264.58 (195.30 to 339.50) | 70.01 (45.26 to 92.04) | -4.31 (-4.73 to -3.89) | 0 |
| Brain and central nervous system cancer | 3.02 (2.35 to 3.68) | 2.74 (2.15 to 3.56) | -0.43 (-0.70 to -0.16) | 0.002 | 1.96 (1.52 to 2.38) | 0.96 (0.79 to 1.22) | -2.36 (-2.60 to -2.11) | 0 | 165.74 (128.14 to 201.99) | 81.25 (66.16 to 103.25) | -2.37 (-2.62 to -2.13) | 0 |
| Neuroblastoma and other peripheral nervous cell tumors | 0.30 (0.23 to 0.40) | 0.34 (0.24 to 0.44) | 0.31 (0.00 to 0.61) | 0.048 | 0.11 (0.09 to 0.13) | 0.10 (0.08 to 0.12) | -0.31 (-0.55 to -0.07) | 0.012 | 9.50 (7.93 to 11.38) | 8.77 (6.69 to 10.92) | -0.32 (-0.55 to -0.08) | 0.008 |
| Non-Hodgkin lymphoma | 1.38 (1.21 to 1.57) | 1.07 (0.91 to 1.28) | -0.99 (-1.40 to -0.58) | 0 | 0.60 (0.51 to 0.70) | 0.19 (0.16 to 0.22) | -3.84 (-4.20 to -3.48) | 0 | 50.86 (43.27 to 59.60) | 15.96 (13.60 to 19.07) | -3.82 (-4.19 to -3.45) | 0 |
| Hodgkin lymphoma | 0.27 (0.21 to 0.30) | 0.16 (0.13 to 0.19) | -1.75 (-2.04 to -1.45) | 0 | 0.09 (0.06 to 0.12) | 0.02 (0.01 to 0.02) | -5.18 (-5.40 to -4.95) | 0 | 7.85 (5.03 to 9.98) | 1.58 (1.21 to 2.10) | -5.10 (-5.33 to -4.87) | 0 |
| Soft tissue and other extraosseous sarcomas | 0.43 (0.34 to 0.54) | 0.23 (0.19 to 0.27) | -2.13 (-2.34 to -1.92) | 0 | 0.16 (0.13 to 0.20) | 0.06 (0.05 to 0.07) | -3.15 (-3.42 to -2.87) | 0 | 13.53 (10.95 to 17.21) | 5.13 (4.28 to 6.26) | -3.18 (-3.45 to -2.91) | 0 |
| Kidney cancer | 1.06 (0.88 to 1.27) | 0.70 (0.58 to 0.83) | -1.47 (-1.72 to -1.21) | 0 | 0.33 (0.27 to 0.39) | 0.11 (0.09 to 0.13) | -3.59 (-3.88 to -3.30) | 0 | 28.75 (23.93 to 34.64) | 9.74 (8.10 to 11.44) | -3.57 (-3.86 to -3.29) | 0 |
| Liver cancer | 0.41 (0.35 to 0.48) | 0.19 (0.15 to 0.24) | -2.38 (-2.87 to -1.88) | 0 | 0.29 (0.24 to 0.34) | 0.08 (0.06 to 0.10) | -4.09 (-4.82 to -3.36) | 0 | 24.82 (21.04 to 29.53) | 6.72 (5.56 to 8.27) | -4.11 (-4.82 to -3.40) | 0 |
| Malignant neoplasm of bone and articular cartilage | 0.72 (0.62 to 0.87) | 0.55 (0.41 to 0.69) | -0.88 (-1.33 to -0.43) | 0 | 0.22 (0.19 to 0.26) | 0.12 (0.09 to 0.15) | -1.75 (-1.87 to -1.63) | 0 | 18.03 (15.93 to 21.64) | 10.07 (7.67 to 12.24) | -1.75 (-1.87 to -1.62) | 0 |
| Thyroid cancer | 0.13 (0.11 to 0.14) | 0.14 (0.11 to 0.17) | 0.25 (-0.14 to 0.65) | 0.204 | 0.01 (0.01 to 0.01) | 0.00 (0.00 to 0.01) | -2.71 (-3.00 to -2.42) | 0 | 0.92 (0.76 to 1.07) | 0.44 (0.35 to 0.54) | -2.38 (-2.67 to -2.09) | 0 |
| Eye cancer | 0.27 (0.17 to 0.41) | 0.49 (0.24 to 0.75) | 1.56 (0.99 to 2.14) | 0 | 0.08 (0.05 to 0.13) | 0.03 (0.01 to 0.04) | -3.41 (-3.62 to -3.20) | 0 | 7.27 (4.11 to 11.26) | 2.82 (1.41 to 4.22) | -3.16 (-3.38 to -2.95) | 0 |
| Acute myeloid leukemia | 1.28 (0.71 to 2.06) | 0.46 (0.32 to 0.62) | -3.33 (-3.61 to -3.04) | 0 | 1.22 (0.66 to 1.97) | 0.38 (0.27 to 0.51) | -3.77 (-4.04 to -3.49) | 0 | 103.46 (55.91 to 168.48) | 31.80 (22.24 to 42.52) | -3.82 (-4.09 to -3.55) | 0 |
| Chronic myeloid leukemia | 0.14 (0.07 to 0.22) | 0.03 (0.02 to 0.05) | -4.50 (-5.15 to -3.85) | 0 | 0.12 (0.06 to 0.19) | 0.01 (0.01 to 0.02) | -6.82 (-7.59 to -6.05) | 0 | 10.29 (4.64 to 15.81) | 1.12 (0.61 to 1.80) | -6.86 (-7.65 to -6.07) | 0 |
| Nasopharynx cancer | 0.09 (0.07 to 0.11) | 0.06 (0.04 to 0.08) | -1.59 (-2.24 to -0.93) | 0 | 0.05 (0.04 to 0.06) | 0.01 (0.01 to 0.02) | -4.29 (-4.80 to -3.77) | 0 | 4.26 (3.54 to 5.19) | 1.12 (0.90 to 1.39) | -4.26 (-4.76 to -3.75) | 0 |
| Other malignant neoplasms | 2.02 (1.37 to 2.47) | 1.26 (0.95 to 1.68) | -1.66 (-2.14 to -1.17) | 0 | 1.08 (0.72 to 1.32) | 0.28 (0.22 to 0.36) | -4.38 (-4.82 to -3.93) | 0 | 92.25 (61.66 to 113.84) | 23.94 (18.73 to 31.14) | -4.40 (-4.86 to -3.94) | 0 |
| Other leukemia | 0.36 (0.19 to 0.56) | 0.17 (0.08 to 0.29) | -2.41 (-2.71 to -2.11) | 0 | 0.10 (0.05 to 0.17) | 0.02 (0.01 to 0.03) | -5.12 (-5.39 to -4.85) | 0 | 9.15 (4.48 to 14.69) | 1.88 (0.84 to 3.03) | -5.07 (-5.34 to -4.81) | 0 |
| **Middle SDI** | | | | | | | | | | | | |
| Acute lymphoid leukemia | 3.58 (2.54 to 4.89) | 2.79 (1.76 to 3.76) | -0.95 (-1.27 to -0.63) | 0 | 3.08 (2.19 to 4.22) | 1.06 (0.73 to 1.32) | -3.50 (-3.71 to -3.28) | 0 | 261.01 (184.72 to 358.54) | 89.01 (61.17 to 111.62) | -3.52 (-3.74 to -3.31) | 0 |
| Brain and central nervous system cancer | 2.22 (1.56 to 2.76) | 1.99 (1.51 to 2.53) | -0.46 (-0.68 to -0.25) | 0 | 1.58 (1.10 to 1.98) | 0.92 (0.70 to 1.16) | -1.81 (-1.99 to -1.63) | 0 | 133.69 (92.90 to 167.87) | 77.48 (58.79 to 97.13) | -1.85 (-2.03 to -1.66) | 0 |
| Neuroblastoma and other peripheral nervous cell tumors | 0.19 (0.13 to 0.27) | 0.27 (0.18 to 0.37) | 1.08 (0.94 to 1.23) | 0 | 0.08 (0.06 to 0.09) | 0.09 (0.07 to 0.12) | 0.44 (0.29 to 0.60) | 0 | 6.91 (5.54 to 8.31) | 7.99 (5.89 to 10.30) | 0.43 (0.28 to 0.58) | 0 |
| Non-Hodgkin lymphoma | 0.99 (0.81 to 1.16) | 0.84 (0.68 to 1.06) | -0.61 (-0.86 to -0.35) | 0 | 0.62 (0.52 to 0.74) | 0.26 (0.21 to 0.33) | -2.84 (-3.07 to -2.60) | 0 | 52.72 (43.55 to 62.28) | 21.97 (17.85 to 27.65) | -2.86 (-3.10 to -2.62) | 0 |
| Hodgkin lymphoma | 0.18 (0.10 to 0.22) | 0.15 (0.09 to 0.20) | -0.61 (-0.85 to -0.36) | 0 | 0.11 (0.06 to 0.15) | 0.04 (0.02 to 0.05) | -3.41 (-3.63 to -3.20) | 0 | 9.37 (5.03 to 12.20) | 3.22 (1.79 to 4.39) | -3.42 (-3.64 to -3.21) | 0 |
| Soft tissue and other extraosseous sarcomas | 0.43 (0.32 to 0.57) | 0.20 (0.15 to 0.27) | -2.51 (-2.69 to -2.33) | 0 | 0.19 (0.14 to 0.25) | 0.07 (0.05 to 0.09) | -3.25 (-3.44 to -3.06) | 0 | 15.97 (12.06 to 21.13) | 5.72 (4.38 to 7.71) | -3.30 (-3.49 to -3.11) | 0 |
| Kidney cancer | 0.63 (0.52 to 0.77) | 0.46 (0.37 to 0.56) | -1.15 (-1.47 to -0.83) | 0 | 0.26 (0.21 to 0.31) | 0.12 (0.10 to 0.15) | -2.47 (-2.78 to -2.16) | 0 | 22.42 (18.63 to 26.71) | 10.70 (8.51 to 13.11) | -2.49 (-2.80 to -2.17) | 0 |
| Liver cancer | 0.50 (0.43 to 0.58) | 0.18 (0.14 to 0.22) | -3.27 (-3.69 to -2.86) | 0 | 0.36 (0.31 to 0.43) | 0.11 (0.09 to 0.13) | -3.83 (-4.22 to -3.43) | 0 | 31.53 (26.97 to 36.94) | 9.17 (7.41 to 11.44) | -3.86 (-4.26 to -3.45) | 0 |
| Malignant neoplasm of bone and articular cartilage | 0.49 (0.38 to 0.63) | 0.57 (0.44 to 0.69) | 0.54 (0.36 to 0.71) | 0 | 0.19 (0.15 to 0.24) | 0.17 (0.13 to 0.20) | -0.39 (-0.56 to -0.22) | 0 | 15.37 (12.08 to 19.76) | 13.59 (10.50 to 16.14) | -0.39 (-0.56 to -0.23) | 0 |
| Thyroid cancer | 0.08 (0.07 to 0.10) | 0.12 (0.09 to 0.15) | 1.19 (0.84 to 1.54) | 0 | 0.01 (0.01 to 0.01) | 0.01 (0.00 to 0.01) | -1.50 (-1.85 to -1.16) | 0 | 0.89 (0.71 to 1.04) | 0.58 (0.43 to 0.71) | -1.32 (-1.66 to -0.98) | 0 |
| Eye cancer | 0.19 (0.11 to 0.29) | 0.32 (0.17 to 0.47) | 1.58 (1.40 to 1.77) | 0 | 0.12 (0.07 to 0.15) | 0.06 (0.03 to 0.08) | -2.13 (-2.36 to -1.91) | 0 | 10.12 (5.91 to 13.55) | 5.42 (3.07 to 7.41) | -2.07 (-2.29 to -1.85) | 0 |
| Acute myeloid leukemia | 1.02 (0.55 to 1.86) | 0.45 (0.33 to 0.64) | -2.66 (-2.81 to -2.51) | 0 | 0.99 (0.54 to 1.81) | 0.41 (0.30 to 0.58) | -2.86 (-3.01 to -2.72) | 0 | 83.73 (45.27 to 154.24) | 34.37 (24.89 to 48.09) | -2.92 (-3.07 to -2.78) | 0 |
| Chronic myeloid leukemia | 0.14 (0.07 to 0.22) | 0.04 (0.02 to 0.06) | -4.30 (-4.54 to -4.05) | 0 | 0.13 (0.06 to 0.21) | 0.02 (0.01 to 0.04) | -5.23 (-5.49 to -4.97) | 0 | 10.76 (5.21 to 17.39) | 2.04 (1.07 to 3.34) | -5.29 (-5.55 to -5.03) | 0 |
| Nasopharynx cancer | 0.09 (0.07 to 0.10) | 0.05 (0.04 to 0.06) | -1.94 (-2.31 to -1.57) | 0 | 0.06 (0.05 to 0.07) | 0.02 (0.02 to 0.02) | -3.56 (-3.87 to -3.26) | 0 | 4.81 (4.17 to 5.57) | 1.56 (1.22 to 1.97) | -3.57 (-3.87 to -3.27) | 0 |
| Other malignant neoplasms | 1.63 (0.83 to 2.09) | 1.09 (0.81 to 1.49) | -1.40 (-1.65 to -1.15) | 0 | 1.00 (0.53 to 1.27) | 0.38 (0.29 to 0.49) | -3.16 (-3.35 to -2.96) | 0 | 85.94 (45.28 to 109.22) | 32.35 (24.44 to 41.99) | -3.19 (-3.39 to -2.99) | 0 |
| Other leukemia | 0.34 (0.14 to 0.58) | 0.16 (0.07 to 0.27) | -2.54 (-2.74 to -2.35) | 0 | 0.12 (0.05 to 0.21) | 0.03 (0.01 to 0.04) | -4.87 (-5.05 to -4.70) | 0 | 10.67 (4.27 to 18.23) | 2.30 (1.09 to 3.88) | -4.86 (-5.04 to -4.68) | 0 |
| **Low-middle SDI** | | | | | | | | | | | | |
| Acute lymphoid leukemia | 1.76 (1.10 to 2.79) | 1.14 (0.79 to 1.44) | -1.41 (-1.54 to -1.28) | 0 | 1.67 (1.03 to 2.68) | 0.92 (0.64 to 1.16) | -1.93 (-2.26 to -1.59) | 0 | 140.27 (85.88 to 225.88) | 76.50 (53.26 to 96.73) | -1.95 (-2.28 to -1.62) | 0 |
| Brain and central nervous system cancer | 1.06 (0.72 to 1.63) | 1.19 (0.90 to 1.53) | 0.40 (0.31 to 0.49) | 0 | 0.85 (0.57 to 1.33) | 0.80 (0.61 to 1.02) | -0.20 (-0.37 to -0.02) | 0.027 | 71.39 (48.13 to 112.15) | 66.73 (50.99 to 85.52) | -0.22 (-0.39 to -0.04) | 0.014 |
| Neuroblastoma and other peripheral nervous cell tumors | 0.17 (0.10 to 0.26) | 0.28 (0.17 to 0.42) | 1.61 (1.23 to 2.00) | 0 | 0.08 (0.06 to 0.10) | 0.11 (0.08 to 0.15) | 1.18 (1.02 to 1.35) | 0 | 6.71 (5.02 to 9.08) | 9.75 (6.74 to 13.43) | 1.18 (1.01 to 1.35) | 0 |
| Non-Hodgkin lymphoma | 1.04 (0.69 to 1.35) | 0.91 (0.69 to 1.22) | -0.43 (-0.61 to -0.24) | 0 | 0.76 (0.50 to 0.98) | 0.47 (0.37 to 0.63) | -1.49 (-1.66 to -1.33) | 0 | 64.18 (42.18 to 83.02) | 39.85 (30.81 to 53.20) | -1.52 (-1.68 to -1.35) | 0 |
| Hodgkin lymphoma | 0.26 (0.14 to 0.37) | 0.23 (0.14 to 0.34) | -0.41 (-0.61 to -0.21) | 0 | 0.20 (0.11 to 0.29) | 0.12 (0.07 to 0.18) | -1.70 (-2.01 to -1.38) | 0 | 16.54 (8.71 to 23.81) | 9.52 (5.61 to 14.30) | -1.70 (-2.02 to -1.38) | 0 |
| Soft tissue and other extraosseous sarcomas | 0.63 (0.44 to 0.89) | 0.34 (0.23 to 0.53) | -1.94 (-2.07 to -1.80) | 0 | 0.32 (0.23 to 0.44) | 0.14 (0.10 to 0.22) | -2.55 (-2.77 to -2.34) | 0 | 27.13 (19.39 to 38.17) | 12.02 (8.28 to 18.68) | -2.58 (-2.78 to -2.38) | 0 |
| Kidney cancer | 0.31 (0.22 to 0.40) | 0.36 (0.27 to 0.46) | 0.50 (0.35 to 0.66) | 0 | 0.16 (0.11 to 0.21) | 0.14 (0.10 to 0.18) | -0.31 (-0.44 to -0.18) | 0 | 13.78 (9.59 to 18.05) | 12.47 (9.10 to 15.97) | -0.31 (-0.44 to -0.18) | 0 |
| Liver cancer | 0.35 (0.24 to 0.44) | 0.21 (0.17 to 0.26) | -1.60 (-1.77 to -1.43) | 0 | 0.26 (0.17 to 0.32) | 0.15 (0.12 to 0.19) | -1.64 (-1.79 to -1.50) | 0 | 22.47 (15.26 to 28.33) | 13.36 (10.62 to 16.61) | -1.65 (-1.80 to -1.50) | 0 |
| Malignant neoplasm of bone and articular cartilage | 0.53 (0.34 to 0.71) | 0.61 (0.47 to 0.78) | 0.45 (0.29 to 0.61) | 0 | 0.24 (0.16 to 0.32) | 0.23 (0.18 to 0.29) | -0.25 (-0.50 to 0.01) | 0.06 | 19.96 (13.29 to 26.46) | 18.42 (14.52 to 23.43) | -0.25 (-0.50 to 0.00) | 0.051 |
| Thyroid cancer | 0.05 (0.04 to 0.06) | 0.08 (0.06 to 0.10) | 1.34 (0.91 to 1.77) | 0 | 0.01 (0.01 to 0.01) | 0.01 (0.01 to 0.01) | -0.44 (-0.99 to 0.11) | 0.117 | 0.77 (0.61 to 0.94) | 0.68 (0.51 to 0.86) | -0.34 (-0.78 to 0.11) | 0.136 |
| Eye cancer | 0.27 (0.15 to 0.38) | 0.31 (0.17 to 0.47) | 0.50 (0.37 to 0.63) | 0 | 0.23 (0.12 to 0.33) | 0.17 (0.10 to 0.24) | -1.01 (-1.22 to -0.79) | 0 | 20.25 (10.92 to 28.98) | 14.82 (8.88 to 21.02) | -1.00 (-1.21 to -0.78) | 0 |
| Acute myeloid leukemia | 0.64 (0.35 to 1.43) | 0.44 (0.30 to 0.66) | -1.25 (-1.44 to -1.05) | 0 | 0.63 (0.35 to 1.40) | 0.42 (0.29 to 0.64) | -1.31 (-1.50 to -1.11) | 0 | 53.41 (29.19 to 119.66) | 35.11 (24.47 to 53.65) | -1.34 (-1.53 to -1.15) | 0 |
| Chronic myeloid leukemia | 0.14 (0.05 to 0.33) | 0.04 (0.02 to 0.06) | -3.85 (-4.13 to -3.57) | 0 | 0.14 (0.05 to 0.32) | 0.04 (0.02 to 0.06) | -4.22 (-4.51 to -3.92) | 0 | 11.63 (3.89 to 26.89) | 2.99 (1.62 to 4.55) | -4.25 (-4.46 to -4.03) | 0 |
| Nasopharynx cancer | 0.07 (0.05 to 0.08) | 0.05 (0.04 to 0.06) | -1.04 (-1.54 to -0.53) | 0 | 0.06 (0.04 to 0.08) | 0.04 (0.03 to 0.05) | -1.50 (-2.00 to -1.01) | 0 | 4.67 (3.44 to 6.05) | 2.88 (2.21 to 3.73) | -1.52 (-2.01 to -1.03) | 0 |
| Other malignant neoplasms | 1.02 (0.60 to 1.50) | 0.83 (0.64 to 1.03) | -0.67 (-0.87 to -0.47) | 0 | 0.84 (0.49 to 1.25) | 0.57 (0.44 to 0.69) | -1.28 (-1.49 to -1.07) | 0 | 71.89 (41.93 to 106.59) | 48.00 (37.49 to 58.85) | -1.30 (-1.50 to -1.10) | 0 |
| Other leukemia | 0.27 (0.11 to 0.57) | 0.19 (0.10 to 0.35) | -1.20 (-1.42 to -0.98) | 0 | 0.13 (0.05 to 0.27) | 0.05 (0.03 to 0.10) | -2.71 (-2.95 to -2.47) | 0 | 10.93 (4.47 to 23.17) | 4.64 (2.44 to 8.67) | -2.72 (-2.96 to -2.49) | 0 |
| **Low SDI** | | | | | | | | | | | | |
| Acute lymphoid leukemia | 1.85 (0.92 to 3.24) | 1.20 (0.71 to 1.59) | -1.40 (-1.64 to -1.15) | 0 | 1.80 (0.89 to 3.19) | 1.10 (0.65 to 1.46) | -1.59 (-1.85 to -1.34) | 0 | 152.35 (75.14 to 270.66) | 92.14 (54.66 to 123.15) | -1.61 (-1.86 to -1.36) | 0 |
| Brain and central nervous system cancer | 0.78 (0.46 to 1.47) | 0.84 (0.56 to 1.12) | 0.26 (0.05 to 0.47) | 0.014 | 0.66 (0.39 to 1.25) | 0.65 (0.43 to 0.88) | -0.06 (-0.32 to 0.20) | 0.639 | 55.87 (33.01 to 106.25) | 54.88 (36.30 to 74.44) | -0.07 (-0.32 to 0.19) | 0.592 |
| Neuroblastoma and other peripheral nervous cell tumors | 0.15 (0.08 to 0.28) | 0.21 (0.10 to 0.38) | 1.17 (0.91 to 1.43) | 0 | 0.07 (0.05 to 0.11) | 0.09 (0.05 to 0.15) | 0.89 (0.72 to 1.05) | 0 | 6.31 (4.14 to 9.86) | 8.17 (4.36 to 13.20) | 0.88 (0.71 to 1.05) | 0 |
| Non-Hodgkin lymphoma | 1.82 (1.16 to 2.40) | 1.28 (0.86 to 1.77) | -1.15 (-1.34 to -0.96) | 0 | 1.51 (0.98 to 2.02) | 0.90 (0.62 to 1.23) | -1.67 (-1.84 to -1.50) | 0 | 128.68 (82.87 to 171.54) | 75.86 (51.99 to 103.94) | -1.70 (-1.87 to -1.53) | 0 |
| Hodgkin lymphoma | 0.39 (0.19 to 0.60) | 0.31 (0.14 to 0.45) | -0.75 (-0.99 to -0.52) | 0 | 0.33 (0.15 to 0.50) | 0.20 (0.09 to 0.30) | -1.52 (-1.75 to -1.29) | 0 | 26.96 (12.64 to 41.48) | 16.62 (7.13 to 24.79) | -1.53 (-1.76 to -1.31) | 0 |
| Soft tissue and other extraosseous sarcomas | 1.29 (0.86 to 2.06) | 0.66 (0.44 to 1.06) | -2.12 (-2.35 to -1.89) | 0 | 0.70 (0.49 to 1.07) | 0.31 (0.21 to 0.48) | -2.63 (-2.84 to -2.43) | 0 | 60.51 (42.13 to 92.78) | 26.26 (17.56 to 41.43) | -2.66 (-2.86 to -2.45) | 0 |
| Kidney cancer | 0.49 (0.29 to 0.67) | 0.53 (0.32 to 0.75) | 0.27 (0.11 to 0.43) | 0.001 | 0.30 (0.18 to 0.41) | 0.26 (0.16 to 0.37) | -0.41 (-0.60 to -0.21) | 0 | 26.36 (15.72 to 36.10) | 23.07 (14.38 to 32.72) | -0.41 (-0.60 to -0.22) | 0 |
| Liver cancer | 0.58 (0.40 to 0.75) | 0.32 (0.22 to 0.43) | -1.94 (-2.03 to -1.84) | 0 | 0.42 (0.29 to 0.55) | 0.23 (0.16 to 0.32) | -1.97 (-2.06 to -1.87) | 0 | 37.02 (25.53 to 47.94) | 20.10 (14.17 to 27.65) | -1.97 (-2.07 to -1.88) | 0 |
| Malignant neoplasm of bone and articular cartilage | 0.63 (0.41 to 0.92) | 0.65 (0.46 to 0.91) | 0.12 (-0.09 to 0.34) | 0.253 | 0.32 (0.22 to 0.46) | 0.27 (0.20 to 0.38) | -0.52 (-0.74 to -0.31) | 0 | 26.09 (17.72 to 37.75) | 22.19 (16.12 to 30.71) | -0.52 (-0.73 to -0.31) | 0 |
| Thyroid cancer | 0.05 (0.04 to 0.07) | 0.07 (0.05 to 0.09) | 0.94 (0.60 to 1.28) | 0 | 0.01 (0.01 to 0.02) | 0.01 (0.01 to 0.01) | -0.62 (-1.00 to -0.24) | 0.002 | 1.00 (0.72 to 1.35) | 0.84 (0.62 to 1.08) | -0.56 (-0.94 to -0.18) | 0.004 |
| Eye cancer | 0.51 (0.27 to 0.74) | 0.43 (0.23 to 0.67) | -0.54 (-0.66 to -0.42) | 0 | 0.45 (0.25 to 0.63) | 0.31 (0.19 to 0.46) | -1.13 (-1.29 to -0.97) | 0 | 39.38 (21.91 to 54.72) | 27.44 (16.43 to 40.05) | -1.13 (-1.29 to -0.97) | 0 |
| Acute myeloid leukemia | 0.47 (0.17 to 1.20) | 0.34 (0.18 to 0.51) | -1.05 (-1.17 to -0.93) | 0 | 0.46 (0.17 to 1.18) | 0.33 (0.17 to 0.50) | -1.07 (-1.20 to -0.95) | 0 | 39.40 (14.47 to 101.24) | 27.97 (14.82 to 42.71) | -1.09 (-1.21 to -0.97) | 0 |
| Chronic myeloid leukemia | 0.11 (0.02 to 0.28) | 0.04 (0.02 to 0.07) | -3.17 (-3.39 to -2.96) | 0 | 0.10 (0.02 to 0.27) | 0.04 (0.02 to 0.07) | -3.36 (-3.57 to -3.15) | 0 | 8.82 (1.81 to 23.45) | 3.05 (1.36 to 5.79) | -3.40 (-3.61 to -3.19) | 0 |
| Nasopharynx cancer | 0.07 (0.04 to 0.09) | 0.04 (0.03 to 0.06) | -1.38 (-1.88 to -0.87) | 0 | 0.06 (0.04 to 0.08) | 0.04 (0.03 to 0.05) | -1.67 (-2.18 to -1.15) | 0 | 4.82 (3.26 to 6.56) | 2.93 (2.13 to 3.97) | -1.68 (-2.19 to -1.16) | 0 |
| Other malignant neoplasms | 1.14 (0.62 to 1.73) | 0.85 (0.57 to 1.12) | -0.94 (-1.10 to -0.77) | 0 | 1.02 (0.56 to 1.55) | 0.70 (0.47 to 0.93) | -1.20 (-1.40 to -0.99) | 0 | 87.52 (47.88 to 132.76) | 59.54 (40.40 to 79.32) | -1.21 (-1.42 to -1.01) | 0 |
| Other leukemia | 0.28 (0.09 to 0.59) | 0.21 (0.09 to 0.38) | -0.93 (-1.03 to -0.82) | 0 | 0.14 (0.04 to 0.30) | 0.07 (0.03 to 0.13) | -2.34 (-2.53 to -2.15) | 0 | 12.35 (3.87 to 26.30) | 5.95 (2.35 to 10.85) | -2.33 (-2.51 to -2.14) | 0 |
| **Central Asia** | | | | | | | | | | | | |
| Acute lymphoid leukemia | 2.39 (1.99 to 2.85) | 1.48 (1.16 to 1.94) | -1.58 (-1.97 to -1.19) | 0 | 2.03 (1.71 to 2.39) | 1.00 (0.80 to 1.30) | -2.30 (-2.67 to -1.92) | 0 | 170.07 (143.03 to 200.34) | 83.42 (66.23 to 108.80) | -2.31 (-2.68 to -1.93) | 0 |
| Brain and central nervous system cancer | 1.91 (1.49 to 2.47) | 2.56 (2.06 to 3.17) | 0.93 (0.57 to 1.29) | 0 | 1.47 (1.16 to 1.89) | 1.74 (1.42 to 2.15) | 0.52 (0.22 to 0.82) | 0.001 | 123.83 (97.29 to 159.39) | 146.06 (118.33 to 180.27) | 0.51 (0.21 to 0.82) | 0.001 |
| Neuroblastoma and other peripheral nervous cell tumors | 0.05 (0.03 to 0.07) | 0.07 (0.04 to 0.11) | 1.25 (0.54 to 1.96) | 0 | 0.02 (0.01 to 0.03) | 0.03 (0.02 to 0.04) | 0.93 (0.61 to 1.26) | 0 | 1.70 (1.21 to 2.45) | 2.23 (1.52 to 3.17) | 0.91 (0.57 to 1.24) | 0 |
| Non-Hodgkin lymphoma | 1.29 (1.01 to 1.60) | 1.08 (0.82 to 1.43) | -0.62 (-0.98 to -0.26) | 0.001 | 0.63 (0.50 to 0.75) | 0.35 (0.28 to 0.44) | -1.82 (-2.18 to -1.46) | 0 | 53.45 (42.36 to 63.62) | 29.77 (23.60 to 37.55) | -1.83 (-2.17 to -1.49) | 0 |
| Hodgkin lymphoma | 0.29 (0.23 to 0.36) | 0.23 (0.18 to 0.30) | -0.70 (-1.07 to -0.32) | 0 | 0.12 (0.10 to 0.15) | 0.06 (0.05 to 0.08) | -2.25 (-2.79 to -1.70) | 0 | 9.89 (7.85 to 12.36) | 4.82 (3.79 to 6.34) | -2.23 (-2.77 to -1.69) | 0 |
| Soft tissue and other extraosseous sarcomas | 0.22 (0.16 to 0.31) | 0.24 (0.18 to 0.31) | 0.18 (-0.35 to 0.71) | 0.507 | 0.09 (0.06 to 0.13) | 0.08 (0.06 to 0.11) | -0.36 (-0.98 to 0.25) | 0.245 | 7.52 (5.36 to 10.84) | 6.93 (5.16 to 9.10) | -0.36 (-0.95 to 0.23) | 0.229 |
| Kidney cancer | 0.60 (0.47 to 0.75) | 0.50 (0.40 to 0.62) | -0.66 (-1.04 to -0.28) | 0.001 | 0.26 (0.20 to 0.32) | 0.17 (0.14 to 0.22) | -1.23 (-1.53 to -0.93) | 0 | 22.23 (17.57 to 27.83) | 15.11 (12.01 to 18.82) | -1.25 (-1.56 to -0.95) | 0 |
| Liver cancer | 0.46 (0.36 to 0.60) | 0.17 (0.12 to 0.22) | -3.24 (-3.45 to -3.02) | 0 | 0.33 (0.26 to 0.43) | 0.12 (0.09 to 0.16) | -3.26 (-3.50 to -3.01) | 0 | 28.92 (22.92 to 37.60) | 10.35 (7.53 to 13.92) | -3.30 (-3.54 to -3.05) | 0 |
| Malignant neoplasm of bone and articular cartilage | 0.54 (0.40 to 0.71) | 0.57 (0.44 to 0.75) | 0.21 (-0.25 to 0.67) | 0.378 | 0.19 (0.14 to 0.24) | 0.17 (0.14 to 0.22) | -0.28 (-0.74 to 0.19) | 0.243 | 15.48 (11.77 to 20.06) | 14.44 (11.30 to 18.42) | -0.28 (-0.74 to 0.19) | 0.24 |
| Thyroid cancer | 0.06 (0.05 to 0.07) | 0.05 (0.04 to 0.06) | -0.78 (-2.20 to 0.67) | 0.291 | 0.01 (0.01 to 0.01) | 0.00 (0.00 to 0.00) | -2.47 (-4.23 to -0.67) | 0.007 | 0.73 (0.65 to 0.81) | 0.35 (0.30 to 0.43) | -2.38 (-4.15 to -0.58) | 0.01 |
| Eye cancer | 0.18 (0.08 to 0.35) | 0.26 (0.13 to 0.46) | 1.11 (0.57 to 1.65) | 0 | 0.06 (0.03 to 0.12) | 0.05 (0.03 to 0.09) | -0.62 (-1.16 to -0.07) | 0.026 | 5.57 (2.65 to 10.35) | 4.71 (2.59 to 8.29) | -0.58 (-1.12 to -0.04) | 0.035 |
| Acute myeloid leukemia | 1.11 (0.82 to 1.39) | 0.70 (0.54 to 0.90) | -1.49 (-1.85 to -1.13) | 0 | 1.07 (0.79 to 1.34) | 0.66 (0.50 to 0.84) | -1.57 (-1.92 to -1.21) | 0 | 89.49 (66.31 to 112.48) | 54.71 (41.98 to 70.50) | -1.59 (-1.95 to -1.22) | 0 |
| Chronic myeloid leukemia | 0.11 (0.08 to 0.15) | 0.05 (0.03 to 0.07) | -2.45 (-2.92 to -1.98) | 0 | 0.10 (0.07 to 0.14) | 0.04 (0.03 to 0.06) | -3.00 (-3.45 to -2.54) | 0 | 8.45 (5.77 to 11.82) | 3.23 (2.20 to 4.75) | -3.04 (-3.49 to -2.59) | 0 |
| Nasopharynx cancer | 0.06 (0.04 to 0.07) | 0.06 (0.04 to 0.08) | 0.11 (-0.25 to 0.47) | 0.551 | 0.05 (0.04 to 0.06) | 0.04 (0.03 to 0.06) | -0.38 (-0.78 to 0.01) | 0.057 | 3.69 (2.94 to 4.64) | 3.26 (2.42 to 4.52) | -0.38 (-0.77 to 0.02) | 0.061 |
| Other malignant neoplasms | 1.76 (1.42 to 2.10) | 1.01 (0.80 to 1.28) | -1.72 (-1.92 to -1.52) | 0 | 1.39 (1.13 to 1.64) | 0.70 (0.57 to 0.88) | -2.09 (-2.65 to -1.53) | 0 | 117.49 (95.30 to 139.58) | 59.20 (47.63 to 74.34) | -2.11 (-2.67 to -1.54) | 0 |
| Other leukemia | 0.17 (0.12 to 0.23) | 0.10 (0.06 to 0.17) | -1.66 (-2.10 to -1.21) | 0 | 0.05 (0.04 to 0.07) | 0.02 (0.01 to 0.03) | -2.94 (-3.32 to -2.56) | 0 | 4.48 (3.26 to 5.90) | 1.80 (1.15 to 2.83) | -2.91 (-3.29 to -2.53) | 0 |
| **Central Europe** | | | | | | | | | | | | |
| Acute lymphoid leukemia | 1.95 (1.73 to 2.22) | 1.58 (1.26 to 2.00) | -0.75 (-1.35 to -0.14) | 0.016 | 1.35 (1.20 to 1.51) | 0.38 (0.33 to 0.44) | -4.08 (-4.59 to -3.56) | 0 | 112.64 (100.63 to 126.62) | 32.33 (28.02 to 37.31) | -4.04 (-4.56 to -3.52) | 0 |
| Brain and central nervous system cancer | 2.81 (2.53 to 3.13) | 1.95 (1.65 to 2.30) | -1.16 (-1.60 to -0.72) | 0 | 1.91 (1.76 to 2.10) | 0.91 (0.80 to 1.04) | -2.31 (-2.74 to -1.88) | 0 | 160.80 (147.50 to 176.66) | 75.92 (66.51 to 86.83) | -2.33 (-2.75 to -1.90) | 0 |
| Neuroblastoma and other peripheral nervous cell tumors | 0.35 (0.26 to 0.47) | 0.28 (0.20 to 0.37) | -0.77 (-1.31 to -0.23) | 0.006 | 0.12 (0.10 to 0.15) | 0.08 (0.07 to 0.10) | -1.19 (-1.78 to -0.61) | 0 | 10.72 (8.92 to 12.99) | 7.24 (5.72 to 9.18) | -1.20 (-1.79 to -0.61) | 0 |
| Non-Hodgkin lymphoma | 1.08 (0.95 to 1.22) | 1.07 (0.88 to 1.31) | -0.10 (-0.83 to 0.65) | 0.799 | 0.43 (0.39 to 0.46) | 0.17 (0.15 to 0.19) | -2.95 (-3.57 to -2.32) | 0 | 36.08 (33.18 to 39.01) | 14.38 (12.62 to 16.35) | -2.88 (-3.50 to -2.26) | 0 |
| Hodgkin lymphoma | 0.39 (0.34 to 0.45) | 0.19 (0.16 to 0.24) | -2.41 (-3.11 to -1.70) | 0 | 0.10 (0.08 to 0.11) | 0.01 (0.01 to 0.02) | -6.08 (-6.67 to -5.50) | 0 | 7.90 (6.85 to 8.99) | 1.19 (0.98 to 1.51) | -5.97 (-6.57 to -5.38) | 0 |
| Soft tissue and other extraosseous sarcomas | 0.48 (0.40 to 0.60) | 0.36 (0.29 to 0.45) | -1.01 (-1.59 to -0.42) | 0.001 | 0.17 (0.14 to 0.21) | 0.10 (0.08 to 0.12) | -1.90 (-2.48 to -1.31) | 0 | 14.31 (11.97 to 17.79) | 8.08 (6.54 to 9.87) | -1.91 (-2.50 to -1.33) | 0 |
| Kidney cancer | 0.58 (0.52 to 0.65) | 0.45 (0.38 to 0.52) | -0.93 (-1.51 to -0.34) | 0.002 | 0.21 (0.18 to 0.23) | 0.09 (0.08 to 0.11) | -2.47 (-3.13 to -1.80) | 0 | 17.90 (15.85 to 19.79) | 8.16 (7.05 to 9.42) | -2.44 (-3.11 to -1.78) | 0 |
| Liver cancer | 0.17 (0.15 to 0.21) | 0.06 (0.05 to 0.07) | -3.41 (-4.18 to -2.62) | 0 | 0.12 (0.10 to 0.14) | 0.03 (0.02 to 0.03) | -4.74 (-5.42 to -4.06) | 0 | 10.34 (8.76 to 12.28) | 2.33 (1.88 to 2.91) | -4.77 (-5.46 to -4.07) | 0 |
| Malignant neoplasm of bone and articular cartilage | 0.82 (0.69 to 0.97) | 0.28 (0.24 to 0.34) | -3.45 (-3.84 to -3.07) | 0 | 0.24 (0.21 to 0.28) | 0.06 (0.05 to 0.07) | -4.28 (-4.70 to -3.85) | 0 | 20.17 (17.27 to 23.32) | 5.23 (4.53 to 6.16) | -4.27 (-4.69 to -3.86) | 0 |
| Thyroid cancer | 0.17 (0.15 to 0.20) | 0.08 (0.07 to 0.09) | -2.31 (-3.69 to -0.92) | 0.001 | 0.01 (0.01 to 0.02) | 0.00 (0.00 to 0.00) | -4.93 (-6.06 to -3.78) | 0 | 1.23 (1.12 to 1.34) | 0.28 (0.25 to 0.32) | -4.67 (-5.80 to -3.52) | 0 |
| Eye cancer | 0.21 (0.12 to 0.39) | 0.17 (0.10 to 0.28) | -0.73 (-1.98 to 0.53) | 0.255 | 0.04 (0.02 to 0.07) | 0.01 (0.01 to 0.02) | -4.36 (-5.66 to -3.05) | 0 | 3.24 (1.69 to 6.30) | 0.88 (0.56 to 1.43) | -4.14 (-5.45 to -2.82) | 0 |
| Acute myeloid leukemia | 0.55 (0.47 to 0.65) | 0.28 (0.24 to 0.33) | -1.95 (-2.53 to -1.37) | 0 | 0.51 (0.44 to 0.60) | 0.23 (0.19 to 0.27) | -2.36 (-2.94 to -1.78) | 0 | 43.46 (36.74 to 51.10) | 19.15 (16.18 to 22.59) | -2.40 (-2.97 to -1.81) | 0 |
| Chronic myeloid leukemia | 0.07 (0.05 to 0.08) | 0.02 (0.01 to 0.02) | -4.45 (-5.16 to -3.74) | 0 | 0.06 (0.04 to 0.07) | 0.01 (0.01 to 0.01) | -6.43 (-7.08 to -5.78) | 0 | 4.70 (3.58 to 5.70) | 0.59 (0.47 to 0.77) | -6.45 (-7.10 to -5.79) | 0 |
| Nasopharynx cancer | 0.02 (0.02 to 0.03) | 0.02 (0.01 to 0.02) | -0.93 (-1.53 to -0.32) | 0.003 | 0.02 (0.01 to 0.02) | 0.01 (0.01 to 0.01) | -2.86 (-3.50 to -2.22) | 0 | 1.28 (1.10 to 1.50) | 0.53 (0.43 to 0.67) | -2.84 (-3.39 to -2.30) | 0 |
| Other malignant neoplasms | 1.32 (1.14 to 1.50) | 0.68 (0.54 to 0.82) | -2.05 (-2.69 to -1.41) | 0 | 0.84 (0.73 to 0.95) | 0.22 (0.18 to 0.26) | -4.21 (-4.97 to -3.45) | 0 | 71.76 (62.75 to 81.25) | 18.69 (15.30 to 22.55) | -4.21 (-4.96 to -3.45) | 0 |
| Other leukemia | 0.11 (0.08 to 0.15) | 0.06 (0.04 to 0.08) | -2.00 (-2.71 to -1.28) | 0 | 0.02 (0.02 to 0.03) | 0.01 (0.00 to 0.01) | -4.39 (-5.15 to -3.63) | 0 | 2.07 (1.66 to 2.77) | 0.53 (0.38 to 0.71) | -4.29 (-5.06 to -3.52) | 0 |
| **Eastern Europe** | | | | | | | | | | | | |
| Acute lymphoid leukemia | 3.22 (2.84 to 3.68) | 1.80 (1.55 to 2.10) | -2.04 (-3.17 to -0.90) | 0.001 | 1.98 (1.78 to 2.20) | 0.46 (0.41 to 0.51) | -4.68 (-5.34 to -4.01) | 0 | 166.66 (149.77 to 184.77) | 39.03 (34.73 to 44.04) | -4.63 (-5.30 to -3.95) | 0 |
| Brain and central nervous system cancer | 2.18 (1.98 to 2.42) | 1.87 (1.66 to 2.11) | -0.65 (-1.23 to -0.07) | 0.028 | 1.53 (1.42 to 1.65) | 1.00 (0.90 to 1.10) | -1.46 (-2.13 to -0.79) | 0 | 128.08 (119.00 to 138.50) | 83.37 (75.07 to 92.18) | -1.48 (-2.17 to -0.79) | 0 |
| Neuroblastoma and other peripheral nervous cell tumors | 0.28 (0.20 to 0.40) | 0.21 (0.15 to 0.29) | -0.94 (-2.00 to 0.13) | 0.086 | 0.10 (0.08 to 0.13) | 0.07 (0.05 to 0.08) | -1.44 (-2.06 to -0.82) | 0 | 8.91 (7.07 to 11.61) | 5.88 (4.35 to 7.35) | -1.44 (-2.08 to -0.80) | 0 |
| Non-Hodgkin lymphoma | 2.13 (1.96 to 2.34) | 1.23 (1.07 to 1.41) | -1.87 (-2.94 to -0.79) | 0.001 | 0.59 (0.55 to 0.63) | 0.19 (0.17 to 0.21) | -3.88 (-5.10 to -2.65) | 0 | 49.88 (46.46 to 53.28) | 15.94 (14.33 to 17.88) | -3.84 (-5.08 to -2.57) | 0 |
| Hodgkin lymphoma | 0.55 (0.51 to 0.60) | 0.27 (0.24 to 0.30) | -2.28 (-3.06 to -1.50) | 0 | 0.12 (0.11 to 0.13) | 0.02 (0.02 to 0.03) | -5.09 (-5.81 to -4.37) | 0 | 9.87 (9.10 to 10.61) | 2.04 (1.83 to 2.31) | -4.99 (-5.68 to -4.30) | 0 |
| Soft tissue and other extraosseous sarcomas | 0.39 (0.33 to 0.46) | 0.32 (0.26 to 0.37) | -0.81 (-1.53 to -0.09) | 0.028 | 0.13 (0.11 to 0.15) | 0.09 (0.07 to 0.10) | -1.28 (-1.96 to -0.61) | 0 | 11.10 (9.78 to 13.04) | 7.54 (6.24 to 8.85) | -1.30 (-1.97 to -0.62) | 0 |
| Kidney cancer | 1.10 (1.01 to 1.21) | 0.53 (0.47 to 0.60) | -2.39 (-2.71 to -2.08) | 0 | 0.29 (0.27 to 0.32) | 0.10 (0.09 to 0.11) | -3.45 (-3.90 to -2.99) | 0 | 25.40 (23.45 to 27.60) | 8.83 (7.84 to 9.90) | -3.42 (-3.86 to -2.98) | 0 |
| Liver cancer | 0.33 (0.31 to 0.36) | 0.20 (0.18 to 0.22) | -1.52 (-2.25 to -0.79) | 0 | 0.23 (0.21 to 0.24) | 0.10 (0.09 to 0.11) | -2.75 (-3.29 to -2.21) | 0 | 19.51 (18.13 to 20.98) | 8.54 (7.63 to 9.43) | -2.75 (-3.29 to -2.21) | 0 |
| Malignant neoplasm of bone and articular cartilage | 1.29 (1.15 to 1.45) | 0.42 (0.36 to 0.49) | -3.67 (-4.49 to -2.85) | 0 | 0.36 (0.34 to 0.39) | 0.10 (0.09 to 0.11) | -3.89 (-4.22 to -3.56) | 0 | 30.08 (28.06 to 32.41) | 8.37 (7.40 to 9.40) | -3.93 (-4.26 to -3.59) | 0 |
| Thyroid cancer | 0.18 (0.16 to 0.19) | 0.13 (0.11 to 0.14) | -1.16 (-1.89 to -0.42) | 0.002 | 0.01 (0.01 to 0.01) | 0.00 (0.00 to 0.00) | -2.85 (-3.69 to -2.00) | 0 | 0.84 (0.77 to 0.91) | 0.38 (0.34 to 0.42) | -2.66 (-3.47 to -1.85) | 0 |
| Eye cancer | 0.30 (0.19 to 0.46) | 0.23 (0.15 to 0.35) | -1.03 (-1.79 to -0.27) | 0.008 | 0.05 (0.04 to 0.07) | 0.02 (0.01 to 0.02) | -3.51 (-5.04 to -1.96) | 0 | 4.65 (3.41 to 6.27) | 1.58 (1.15 to 2.22) | -3.39 (-4.89 to -1.86) | 0 |
| Acute myeloid leukemia | 0.99 (0.78 to 1.19) | 0.31 (0.28 to 0.36) | -3.72 (-4.32 to -3.11) | 0 | 0.91 (0.72 to 1.10) | 0.27 (0.23 to 0.31) | -3.90 (-4.47 to -3.34) | 0 | 76.24 (60.15 to 91.69) | 22.24 (19.60 to 25.76) | -3.90 (-4.49 to -3.31) | 0 |
| Chronic myeloid leukemia | 0.08 (0.07 to 0.10) | 0.02 (0.02 to 0.03) | -4.29 (-4.96 to -3.62) | 0 | 0.06 (0.05 to 0.08) | 0.01 (0.01 to 0.01) | -5.61 (-6.30 to -4.91) | 0 | 5.41 (4.30 to 6.74) | 0.93 (0.71 to 1.22) | -5.63 (-6.34 to -4.91) | 0 |
| Nasopharynx cancer | 0.03 (0.03 to 0.04) | 0.02 (0.01 to 0.02) | -1.86 (-2.63 to -1.09) | 0 | 0.02 (0.02 to 0.03) | 0.01 (0.01 to 0.01) | -2.75 (-3.40 to -2.09) | 0 | 1.95 (1.70 to 2.25) | 0.84 (0.70 to 1.04) | -2.76 (-3.41 to -2.11) | 0 |
| Other malignant neoplasms | 2.79 (2.55 to 3.04) | 1.51 (1.31 to 1.77) | -2.28 (-4.03 to -0.49) | 0.013 | 1.21 (1.10 to 1.34) | 0.37 (0.32 to 0.43) | -3.96 (-5.10 to -2.81) | 0 | 103.15 (93.48 to 113.76) | 31.68 (27.64 to 36.83) | -3.92 (-5.08 to -2.76) | 0 |
| Other leukemia | 0.26 (0.20 to 0.34) | 0.11 (0.07 to 0.15) | -2.77 (-3.63 to -1.91) | 0 | 0.06 (0.05 to 0.08) | 0.01 (0.01 to 0.02) | -4.62 (-5.48 to -3.74) | 0 | 5.10 (4.15 to 6.70) | 1.29 (0.89 to 1.64) | -4.54 (-5.40 to -3.68) | 0 |
| **Australasia** | | | | | | | | | | | | |
| Acute lymphoid leukemia | 3.23 (2.55 to 4.09) | 3.51 (2.46 to 4.84) | 0.31 (-1.10 to 1.74) | 0.666 | 0.87 (0.77 to 0.98) | 0.28 (0.22 to 0.35) | -3.55 (-4.00 to -3.10) | 0 | 73.84 (65.30 to 83.35) | 25.12 (19.89 to 31.26) | -3.32 (-3.78 to -2.85) | 0 |
| Brain and central nervous system cancer | 2.61 (2.17 to 3.16) | 2.19 (1.60 to 2.93) | -0.60 (-1.31 to 0.12) | 0.105 | 1.16 (1.05 to 1.28) | 0.71 (0.60 to 0.84) | -1.67 (-2.40 to -0.94) | 0 | 97.81 (88.25 to 108.07) | 59.87 (50.41 to 70.39) | -1.67 (-2.40 to -0.95) | 0 |
| Neuroblastoma and other peripheral nervous cell tumors | 0.63 (0.50 to 0.80) | 0.52 (0.35 to 0.77) | -0.53 (-1.62 to 0.57) | 0.34 | 0.17 (0.14 to 0.21) | 0.14 (0.10 to 0.20) | -0.59 (-1.75 to 0.59) | 0.327 | 15.23 (12.56 to 18.33) | 12.19 (8.32 to 17.75) | -0.60 (-1.77 to 0.58) | 0.314 |
| Non-Hodgkin lymphoma | 1.64 (1.25 to 2.18) | 1.15 (0.82 to 1.60) | -1.06 (-1.95 to -0.15) | 0.022 | 0.24 (0.21 to 0.29) | 0.08 (0.06 to 0.10) | -3.58 (-4.48 to -2.68) | 0 | 21.19 (17.88 to 25.29) | 7.02 (5.53 to 8.79) | -3.44 (-4.34 to -2.52) | 0 |
| Hodgkin lymphoma | 0.32 (0.23 to 0.44) | 0.16 (0.11 to 0.22) | -2.22 (-3.22 to -1.20) | 0 | 0.02 (0.02 to 0.03) | 0.01 (0.00 to 0.01) | -5.09 (-6.12 to -4.05) | 0 | 2.15 (1.70 to 2.72) | 0.49 (0.36 to 0.67) | -4.80 (-5.85 to -3.74) | 0 |
| Soft tissue and other extraosseous sarcomas | 0.50 (0.41 to 0.62) | 0.47 (0.34 to 0.65) | -0.15 (-1.95 to 1.68) | 0.871 | 0.13 (0.11 to 0.16) | 0.10 (0.07 to 0.14) | -0.80 (-2.43 to 0.86) | 0.345 | 11.12 (9.11 to 13.54) | 8.58 (6.09 to 11.75) | -0.79 (-2.57 to 1.01) | 0.387 |
| Kidney cancer | 0.55 (0.47 to 0.65) | 0.43 (0.34 to 0.55) | -0.84 (-1.15 to -0.54) | 0 | 0.13 (0.11 to 0.15) | 0.05 (0.04 to 0.07) | -2.74 (-3.02 to -2.45) | 0 | 11.34 (10.01 to 12.92) | 4.86 (3.96 to 5.87) | -2.69 (-2.99 to -2.40) | 0 |
| Liver cancer | 0.15 (0.13 to 0.17) | 0.26 (0.20 to 0.34) | 1.99 (0.77 to 3.24) | 0.001 | 0.05 (0.05 to 0.06) | 0.07 (0.05 to 0.09) | 0.97 (-0.13 to 2.08) | 0.083 | 4.71 (4.01 to 5.46) | 6.11 (4.69 to 7.83) | 0.98 (-0.11 to 2.09) | 0.079 |
| Malignant neoplasm of bone and articular cartilage | 0.63 (0.53 to 0.76) | 0.41 (0.31 to 0.54) | -1.31 (-3.02 to 0.44) | 0.141 | 0.14 (0.12 to 0.17) | 0.07 (0.06 to 0.10) | -2.16 (-3.76 to -0.53) | 0.009 | 11.70 (9.89 to 13.72) | 6.18 (4.75 to 7.91) | -2.14 (-3.73 to -0.53) | 0.01 |
| Thyroid cancer | 0.07 (0.06 to 0.09) | 0.08 (0.05 to 0.11) | 0.23 (-0.48 to 0.95) | 0.523 | 0.00 (0.00 to 0.00) | 0.00 (0.00 to 0.00) | -1.13 (-1.64 to -0.62) | 0 | 0.27 (0.22 to 0.33) | 0.20 (0.15 to 0.27) | -0.92 (-1.39 to -0.44) | 0 |
| Eye cancer | 0.25 (0.16 to 0.36) | 0.11 (0.05 to 0.21) | -2.43 (-3.93 to -0.91) | 0.002 | 0.01 (0.00 to 0.01) | 0.00 (0.00 to 0.00) | -3.80 (-6.16 to -1.38) | 0.002 | 0.71 (0.52 to 0.96) | 0.21 (0.11 to 0.36) | -3.99 (-6.26 to -1.66) | 0.001 |
| Acute myeloid leukemia | 0.62 (0.52 to 0.73) | 0.47 (0.36 to 0.61) | -0.83 (-1.47 to -0.18) | 0.012 | 0.39 (0.33 to 0.44) | 0.23 (0.18 to 0.29) | -1.64 (-2.29 to -0.99) | 0 | 32.52 (27.94 to 37.41) | 19.21 (15.13 to 23.94) | -1.73 (-2.43 to -1.01) | 0 |
| Chronic myeloid leukemia | 0.11 (0.08 to 0.14) | 0.04 (0.03 to 0.07) | -3.04 (-3.73 to -2.34) | 0 | 0.05 (0.04 to 0.07) | 0.01 (0.01 to 0.01) | -5.62 (-6.14 to -5.09) | 0 | 4.40 (3.38 to 5.67) | 0.73 (0.46 to 1.19) | -5.59 (-6.12 to -5.06) | 0 |
| Nasopharynx cancer | 0.08 (0.06 to 0.10) | 0.04 (0.03 to 0.06) | -2.31 (-3.04 to -1.57) | 0 | 0.01 (0.01 to 0.02) | 0.00 (0.00 to 0.01) | -3.63 (-4.32 to -2.94) | 0 | 1.02 (0.83 to 1.27) | 0.34 (0.23 to 0.49) | -3.56 (-4.25 to -2.86) | 0 |
| Other malignant neoplasms | 1.57 (1.17 to 2.11) | 1.90 (1.33 to 2.73) | 0.81 (-0.47 to 2.11) | 0.216 | 0.24 (0.21 to 0.28) | 0.15 (0.11 to 0.20) | -1.40 (-2.12 to -0.68) | 0 | 21.13 (18.14 to 24.44) | 13.58 (10.30 to 18.09) | -1.27 (-2.00 to -0.55) | 0.001 |
| Other leukemia | 0.13 (0.08 to 0.21) | 0.11 (0.06 to 0.16) | -0.69 (-1.54 to 0.16) | 0.111 | 0.02 (0.02 to 0.03) | 0.01 (0.00 to 0.01) | -3.56 (-4.19 to -2.93) | 0 | 2.00 (1.56 to 2.51) | 0.66 (0.44 to 0.97) | -3.35 (-3.99 to -2.70) | 0 |
| **High-income Asia Pacific** | | | | | | | | | | | | |
| Acute lymphoid leukemia | 4.55 (3.46 to 6.01) | 4.54 (3.51 to 5.68) | 0.20 (-0.71 to 1.11) | 0.67 | 1.17 (0.96 to 1.39) | 0.31 (0.26 to 0.35) | -4.27 (-4.78 to -3.76) | 0 | 98.19 (80.89 to 117.15) | 28.15 (24.05 to 32.81) | -3.94 (-4.42 to -3.46) | 0 |
| Brain and central nervous system cancer | 2.32 (1.90 to 2.79) | 3.25 (2.46 to 4.14) | 1.02 (0.15 to 1.89) | 0.021 | 0.79 (0.65 to 0.91) | 0.64 (0.55 to 0.72) | -0.74 (-1.37 to -0.11) | 0.022 | 66.19 (54.48 to 76.17) | 54.62 (46.57 to 61.71) | -0.69 (-1.32 to -0.06) | 0.033 |
| Neuroblastoma and other peripheral nervous cell tumors | 0.76 (0.61 to 0.94) | 0.68 (0.55 to 0.81) | -0.20 (-0.52 to 0.12) | 0.231 | 0.23 (0.20 to 0.28) | 0.18 (0.16 to 0.21) | -0.80 (-1.27 to -0.33) | 0.001 | 20.50 (17.89 to 24.17) | 15.82 (13.67 to 18.16) | -0.80 (-1.28 to -0.32) | 0.001 |
| Non-Hodgkin lymphoma | 0.92 (0.73 to 1.17) | 1.02 (0.73 to 1.39) | 0.26 (-0.29 to 0.81) | 0.356 | 0.33 (0.29 to 0.38) | 0.13 (0.11 to 0.14) | -3.33 (-3.60 to -3.06) | 0 | 27.54 (24.43 to 31.59) | 10.82 (9.77 to 12.04) | -3.23 (-3.50 to -2.96) | 0 |
| Hodgkin lymphoma | 0.02 (0.02 to 0.02) | 0.03 (0.02 to 0.03) | 1.20 (0.18 to 2.24) | 0.021 | 0.01 (0.00 to 0.01) | 0.00 (0.00 to 0.00) | -3.33 (-3.62 to -3.04) | 0 | 0.44 (0.35 to 0.61) | 0.16 (0.14 to 0.20) | -3.11 (-3.40 to -2.82) | 0 |
| Soft tissue and other extraosseous sarcomas | 0.53 (0.44 to 0.64) | 0.36 (0.30 to 0.42) | -1.34 (-1.76 to -0.91) | 0 | 0.15 (0.12 to 0.19) | 0.08 (0.07 to 0.09) | -2.05 (-2.58 to -1.51) | 0 | 12.84 (10.48 to 15.81) | 6.59 (5.73 to 7.63) | -2.17 (-2.71 to -1.63) | 0 |
| Kidney cancer | 0.31 (0.27 to 0.35) | 0.32 (0.27 to 0.38) | 0.19 (-0.43 to 0.81) | 0.55 | 0.09 (0.08 to 0.10) | 0.04 (0.04 to 0.05) | -2.11 (-2.59 to -1.63) | 0 | 7.52 (6.60 to 8.60) | 3.93 (3.49 to 4.44) | -2.05 (-2.49 to -1.62) | 0 |
| Liver cancer | 0.37 (0.30 to 0.47) | 0.21 (0.18 to 0.26) | -1.54 (-2.19 to -0.89) | 0 | 0.19 (0.14 to 0.26) | 0.05 (0.05 to 0.06) | -3.97 (-4.30 to -3.63) | 0 | 16.48 (12.13 to 22.48) | 4.68 (4.03 to 5.63) | -3.91 (-4.25 to -3.56) | 0 |
| Malignant neoplasm of bone and articular cartilage | 0.70 (0.60 to 0.84) | 0.43 (0.37 to 0.50) | -1.39 (-2.00 to -0.77) | 0 | 0.17 (0.15 to 0.21) | 0.08 (0.07 to 0.09) | -2.29 (-2.99 to -1.58) | 0 | 14.07 (11.92 to 16.99) | 6.68 (5.91 to 7.60) | -2.28 (-2.99 to -1.56) | 0 |
| Thyroid cancer | 0.19 (0.16 to 0.22) | 0.14 (0.12 to 0.16) | -1.02 (-1.37 to -0.66) | 0 | 0.01 (0.01 to 0.01) | 0.00 (0.00 to 0.00) | -2.80 (-3.12 to -2.48) | 0 | 0.73 (0.61 to 0.90) | 0.34 (0.30 to 0.41) | -2.52 (-2.83 to -2.20) | 0 |
| Eye cancer | 0.49 (0.35 to 0.68) | 0.59 (0.39 to 0.87) | 0.75 (-0.51 to 2.03) | 0.244 | 0.03 (0.02 to 0.04) | 0.01 (0.01 to 0.01) | -3.60 (-4.52 to -2.67) | 0 | 2.72 (1.96 to 3.94) | 1.12 (0.78 to 1.59) | -2.86 (-3.82 to -1.89) | 0 |
| Acute myeloid leukemia | 0.70 (0.55 to 0.90) | 0.34 (0.28 to 0.41) | -2.28 (-2.89 to -1.67) | 0 | 0.60 (0.46 to 0.79) | 0.23 (0.19 to 0.28) | -3.01 (-3.68 to -2.34) | 0 | 49.98 (38.72 to 65.48) | 19.55 (16.19 to 23.24) | -3.09 (-3.69 to -2.49) | 0 |
| Chronic myeloid leukemia | 0.21 (0.17 to 0.27) | 0.07 (0.05 to 0.09) | -3.60 (-3.96 to -3.25) | 0 | 0.12 (0.09 to 0.16) | 0.02 (0.01 to 0.02) | -6.47 (-7.03 to -5.91) | 0 | 10.05 (7.67 to 13.67) | 1.28 (1.02 to 1.65) | -6.48 (-6.98 to -5.96) | 0 |
| Nasopharynx cancer | 0.01 (0.01 to 0.01) | 0.01 (0.01 to 0.01) | -0.16 (-0.59 to 0.27) | 0.469 | 0.01 (0.00 to 0.01) | 0.00 (0.00 to 0.00) | -2.77 (-3.41 to -2.13) | 0 | 0.48 (0.39 to 0.60) | 0.21 (0.18 to 0.25) | -2.74 (-3.38 to -2.10) | 0 |
| Other malignant neoplasms | 0.91 (0.71 to 1.12) | 1.01 (0.77 to 1.27) | 0.33 (-0.10 to 0.77) | 0.135 | 0.38 (0.28 to 0.46) | 0.17 (0.15 to 0.20) | -2.68 (-3.25 to -2.11) | 0 | 31.95 (23.69 to 38.69) | 15.08 (12.88 to 17.65) | -2.63 (-3.19 to -2.06) | 0 |
| Other leukemia | 0.05 (0.03 to 0.08) | 0.06 (0.03 to 0.08) | 0.26 (-0.27 to 0.80) | 0.342 | 0.01 (0.00 to 0.01) | 0.00 (0.00 to 0.01) | -1.61 (-2.13 to -1.10) | 0 | 0.61 (0.43 to 0.82) | 0.38 (0.26 to 0.49) | -1.49 (-2.04 to -0.95) | 0 |
| Acute lymphoid leukemia | 5.06 (4.67 to 5.49) | 3.47 (3.05 to 3.94) | -0.93 (-1.14 to -0.71) | 0 | 0.79 (0.77 to 0.82) | 0.30 (0.27 to 0.32) | -3.18 (-3.61 to -2.74) | 0 | 68.48 (66.21 to 71.10) | 26.55 (24.35 to 29.23) | -3.00 (-3.41 to -2.59) | 0 |
| Brain and central nervous system cancer | 3.44 (3.20 to 3.70) | 3.07 (2.75 to 3.42) | -0.31 (-0.71 to 0.09) | 0.124 | 1.05 (1.02 to 1.08) | 0.75 (0.70 to 0.81) | -1.03 (-1.29 to -0.76) | 0 | 88.75 (86.29 to 91.33) | 63.45 (58.55 to 68.47) | -1.03 (-1.32 to -0.74) | 0 |
| Neuroblastoma and other peripheral nervous cell tumors | 0.77 (0.64 to 0.91) | 0.53 (0.43 to 0.65) | -1.14 (-1.39 to -0.88) | 0 | 0.23 (0.21 to 0.25) | 0.14 (0.12 to 0.17) | -1.44 (-1.77 to -1.11) | 0 | 19.95 (18.26 to 21.59) | 12.60 (10.75 to 14.64) | -1.46 (-1.78 to -1.13) | 0 |
| Non-Hodgkin lymphoma | 1.58 (1.44 to 1.72) | 1.02 (0.88 to 1.17) | -1.22 (-1.47 to -0.98) | 0 | 0.20 (0.19 to 0.20) | 0.07 (0.06 to 0.08) | -3.21 (-3.90 to -2.52) | 0 | 16.92 (16.20 to 17.70) | 6.30 (5.74 to 6.87) | -3.10 (-3.68 to -2.52) | 0 |
| Hodgkin lymphoma | 0.29 (0.28 to 0.32) | 0.11 (0.10 to 0.12) | -3.06 (-3.36 to -2.75) | 0 | 0.02 (0.02 to 0.02) | 0.00 (0.00 to 0.01) | -4.98 (-5.13 to -4.83) | 0 | 1.86 (1.77 to 1.97) | 0.44 (0.41 to 0.48) | -4.78 (-4.91 to -4.65) | 0 |
| Soft tissue and other extraosseous sarcomas | 0.68 (0.62 to 0.73) | 0.54 (0.48 to 0.61) | -0.71 (-1.34 to -0.08) | 0.027 | 0.18 (0.17 to 0.18) | 0.13 (0.12 to 0.14) | -1.06 (-1.79 to -0.33) | 0.005 | 14.97 (14.40 to 15.58) | 10.78 (9.78 to 11.86) | -1.06 (-1.78 to -0.34) | 0.004 |
| Kidney cancer | 0.84 (0.81 to 0.88) | 0.66 (0.60 to 0.72) | -0.69 (-1.29 to -0.08) | 0.027 | 0.12 (0.11 to 0.12) | 0.07 (0.06 to 0.07) | -1.71 (-2.31 to -1.10) | 0 | 10.37 (10.05 to 10.69) | 6.03 (5.54 to 6.56) | -1.67 (-2.26 to -1.08) | 0 |
| Liver cancer | 0.19 (0.18 to 0.20) | 0.32 (0.28 to 0.36) | 1.86 (1.42 to 2.30) | 0 | 0.07 (0.07 to 0.07) | 0.09 (0.08 to 0.10) | 0.66 (-0.09 to 1.42) | 0.087 | 6.22 (6.02 to 6.41) | 7.58 (6.81 to 8.40) | 0.69 (-0.07 to 1.45) | 0.077 |
| Malignant neoplasm of bone and articular cartilage | 0.61 (0.56 to 0.66) | 0.60 (0.53 to 0.66) | -0.04 (-1.18 to 1.10) | 0.94 | 0.14 (0.13 to 0.14) | 0.12 (0.11 to 0.13) | -0.28 (-1.01 to 0.45) | 0.446 | 11.17 (10.74 to 11.64) | 9.82 (9.09 to 10.67) | -0.20 (-1.40 to 1.01) | 0.74 |
| Thyroid cancer | 0.12 (0.12 to 0.13) | 0.11 (0.10 to 0.12) | -0.26 (-0.71 to 0.20) | 0.272 | 0.00 (0.00 to 0.00) | 0.00 (0.00 to 0.00) | -0.87 (-1.21 to -0.53) | 0 | 0.35 (0.33 to 0.38) | 0.27 (0.25 to 0.30) | -0.78 (-1.23 to -0.32) | 0.001 |
| Eye cancer | 0.77 (0.59 to 0.99) | 0.42 (0.27 to 0.63) | -1.97 (-2.96 to -0.96) | 0 | 0.02 (0.02 to 0.03) | 0.01 (0.01 to 0.01) | -3.63 (-4.65 to -2.59) | 0 | 2.51 (2.18 to 2.91) | 0.92 (0.68 to 1.27) | -3.28 (-4.29 to -2.26) | 0 |
| Acute myeloid leukemia | 0.51 (0.49 to 0.53) | 0.39 (0.36 to 0.43) | -0.86 (-1.09 to -0.64) | 0 | 0.39 (0.38 to 0.41) | 0.26 (0.24 to 0.28) | -1.09 (-1.56 to -0.61) | 0 | 33.00 (31.86 to 34.23) | 21.77 (19.99 to 23.74) | -1.35 (-2.08 to -0.61) | 0 |
| Chronic myeloid leukemia | 0.17 (0.16 to 0.18) | 0.04 (0.04 to 0.05) | -4.33 (-4.68 to -3.97) | 0 | 0.06 (0.06 to 0.06) | 0.01 (0.01 to 0.01) | -5.69 (-5.94 to -5.44) | 0 | 5.01 (4.71 to 5.37) | 0.80 (0.70 to 0.92) | -5.67 (-5.92 to -5.43) | 0 |
| Nasopharynx cancer | 0.03 (0.03 to 0.04) | 0.02 (0.02 to 0.02) | -1.97 (-2.30 to -1.65) | 0 | 0.01 (0.01 to 0.01) | 0.00 (0.00 to 0.00) | -2.93 (-3.25 to -2.61) | 0 | 0.54 (0.52 to 0.57) | 0.21 (0.20 to 0.23) | -2.90 (-3.21 to -2.58) | 0 |
| Other malignant neoplasms | 1.26 (1.18 to 1.34) | 1.10 (0.97 to 1.24) | -0.31 (-0.87 to 0.26) | 0.284 | 0.24 (0.24 to 0.25) | 0.14 (0.13 to 0.15) | -1.65 (-1.82 to -1.48) | 0 | 21.25 (20.57 to 21.98) | 12.33 (11.31 to 13.48) | -1.59 (-1.76 to -1.43) | 0 |
| Other leukemia | 0.01 (0.01 to 0.01) | 0.01 (0.01 to 0.02) | 0.53 (0.25 to 0.81) | 0 | 0.00 (0.00 to 0.00) | 0.00 (0.00 to 0.00) | -0.59 (-0.98 to -0.20) | 0.003 | 0.15 (0.13 to 0.16) | 0.12 (0.10 to 0.14) | -0.54 (-0.92 to -0.16) | 0.005 |
| **Southern Latin America** | | | | | | | | | | | | |
| Acute lymphoid leukemia | 2.16 (1.88 to 2.48) | 2.09 (1.58 to 2.74) | -0.09 (-0.65 to 0.48) | 0.762 | 1.62 (1.44 to 1.83) | 0.82 (0.67 to 1.01) | -2.17 (-2.74 to -1.60) | 0 | 134.76 (119.41 to 151.98) | 68.56 (55.61 to 84.34) | -2.16 (-2.73 to -1.59) | 0 |
| Brain and central nervous system cancer | 1.30 (1.04 to 1.66) | 1.59 (1.19 to 2.14) | 0.79 (0.24 to 1.33) | 0.005 | 0.83 (0.71 to 0.97) | 0.77 (0.65 to 0.92) | -0.15 (-0.67 to 0.37) | 0.565 | 69.68 (59.65 to 81.74) | 64.57 (53.98 to 76.67) | -0.17 (-0.68 to 0.34) | 0.507 |
| Neuroblastoma and other peripheral nervous cell tumors | 0.32 (0.21 to 0.47) | 0.43 (0.28 to 0.64) | 1.23 (0.80 to 1.66) | 0 | 0.11 (0.08 to 0.16) | 0.13 (0.09 to 0.20) | 0.59 (-0.47 to 1.66) | 0.277 | 9.94 (7.08 to 13.70) | 11.61 (7.88 to 17.20) | 0.58 (-0.48 to 1.65) | 0.286 |
| Non-Hodgkin lymphoma | 0.85 (0.67 to 1.09) | 0.77 (0.57 to 1.03) | -0.45 (-1.37 to 0.47) | 0.338 | 0.41 (0.36 to 0.47) | 0.20 (0.16 to 0.24) | -2.26 (-2.62 to -1.91) | 0 | 34.03 (29.82 to 39.16) | 16.67 (13.40 to 20.45) | -2.23 (-2.59 to -1.87) | 0 |
| Hodgkin lymphoma | 0.20 (0.14 to 0.29) | 0.20 (0.14 to 0.29) | -0.27 (-2.03 to 1.52) | 0.762 | 0.06 (0.04 to 0.08) | 0.02 (0.01 to 0.03) | -3.57 (-4.46 to -2.67) | 0 | 4.55 (3.32 to 6.21) | 1.67 (1.21 to 2.28) | -3.45 (-4.36 to -2.54) | 0 |
| Soft tissue and other extraosseous sarcomas | 0.43 (0.32 to 0.59) | 0.38 (0.27 to 0.53) | -0.40 (-1.17 to 0.37) | 0.31 | 0.16 (0.12 to 0.22) | 0.11 (0.08 to 0.16) | -1.05 (-1.85 to -0.23) | 0.012 | 13.58 (10.02 to 18.42) | 9.59 (6.72 to 13.51) | -1.01 (-1.78 to -0.22) | 0.012 |
| Kidney cancer | 0.96 (0.80 to 1.15) | 1.04 (0.81 to 1.33) | 0.23 (-0.50 to 0.97) | 0.537 | 0.17 (0.15 to 0.20) | 0.11 (0.09 to 0.13) | -1.63 (-2.25 to -1.01) | 0 | 14.80 (12.74 to 17.02) | 9.36 (7.52 to 11.39) | -1.57 (-2.20 to -0.94) | 0 |
| Liver cancer | 0.03 (0.02 to 0.03) | 0.03 (0.03 to 0.04) | 0.93 (0.55 to 1.32) | 0 | 0.02 (0.02 to 0.02) | 0.02 (0.02 to 0.03) | 0.36 (-0.03 to 0.75) | 0.074 | 1.65 (1.36 to 1.98) | 1.80 (1.39 to 2.29) | 0.32 (-0.06 to 0.71) | 0.101 |
| Malignant neoplasm of bone and articular cartilage | 0.94 (0.73 to 1.19) | 0.60 (0.47 to 0.77) | -1.44 (-2.69 to -0.16) | 0.027 | 0.31 (0.24 to 0.38) | 0.16 (0.12 to 0.20) | -2.20 (-3.41 to -0.98) | 0 | 25.29 (19.90 to 31.39) | 12.83 (10.12 to 16.20) | -2.19 (-3.39 to -0.97) | 0 |
| Thyroid cancer | 0.07 (0.06 to 0.09) | 0.08 (0.06 to 0.10) | 0.23 (-0.91 to 1.38) | 0.696 | 0.01 (0.01 to 0.01) | 0.00 (0.00 to 0.01) | -1.88 (-2.70 to -1.05) | 0 | 0.66 (0.55 to 0.77) | 0.40 (0.32 to 0.49) | -1.72 (-2.54 to -0.90) | 0 |
| Eye cancer | 0.32 (0.16 to 0.59) | 0.24 (0.13 to 0.39) | -1.11 (-1.96 to -0.25) | 0.012 | 0.05 (0.03 to 0.09) | 0.01 (0.01 to 0.02) | -4.76 (-5.72 to -3.78) | 0 | 4.86 (3.04 to 7.80) | 1.26 (0.77 to 1.94) | -4.53 (-5.50 to -3.56) | 0 |
| Acute myeloid leukemia | 0.86 (0.72 to 1.02) | 0.46 (0.36 to 0.59) | -1.77 (-2.59 to -0.94) | 0 | 0.81 (0.68 to 0.97) | 0.41 (0.32 to 0.52) | -1.98 (-2.82 to -1.13) | 0 | 68.05 (57.50 to 80.94) | 34.30 (26.75 to 43.59) | -1.99 (-2.82 to -1.15) | 0 |
| Chronic myeloid leukemia | 0.08 (0.06 to 0.12) | 0.02 (0.01 to 0.03) | -4.52 (-5.21 to -3.84) | 0 | 0.07 (0.05 to 0.10) | 0.01 (0.01 to 0.02) | -5.74 (-6.26 to -5.22) | 0 | 5.87 (4.15 to 8.50) | 0.92 (0.61 to 1.38) | -5.75 (-6.27 to -5.23) | 0 |
| Nasopharynx cancer | 0.02 (0.01 to 0.02) | 0.01 (0.01 to 0.02) | -1.83 (-2.65 to -1.00) | 0 | 0.01 (0.01 to 0.02) | 0.00 (0.00 to 0.01) | -2.94 (-3.30 to -2.58) | 0 | 1.02 (0.79 to 1.30) | 0.39 (0.26 to 0.58) | -2.93 (-3.29 to -2.57) | 0 |
| Other malignant neoplasms | 0.80 (0.63 to 1.02) | 0.51 (0.38 to 0.69) | -1.43 (-1.97 to -0.90) | 0 | 0.58 (0.46 to 0.73) | 0.27 (0.21 to 0.35) | -2.59 (-3.28 to -1.89) | 0 | 49.22 (38.59 to 61.62) | 22.46 (17.21 to 29.28) | -2.61 (-3.32 to -1.89) | 0 |
| Other leukemia | 0.07 (0.05 to 0.11) | 0.06 (0.03 to 0.10) | -0.57 (-1.41 to 0.28) | 0.19 | 0.02 (0.01 to 0.02) | 0.01 (0.00 to 0.01) | -2.36 (-3.15 to -1.57) | 0 | 1.54 (1.09 to 2.17) | 0.74 (0.43 to 1.21) | -2.42 (-3.23 to -1.60) | 0 |
| **Western Europe** | | | | | | | | | | | | |
| Acute lymphoid leukemia | 6.05 (5.42 to 6.80) | 5.55 (4.82 to 6.40) | -0.38 (-0.96 to 0.19) | 0.19 | 0.98 (0.94 to 1.02) | 0.33 (0.30 to 0.36) | -3.50 (-3.62 to -3.37) | 0 | 84.29 (80.33 to 88.54) | 30.53 (27.64 to 34.02) | -3.22 (-3.36 to -3.09) | 0 |
| Brain and central nervous system cancer | 3.05 (2.82 to 3.30) | 2.82 (2.49 to 3.19) | -0.23 (-0.47 to 0.00) | 0.05 | 1.24 (1.19 to 1.28) | 0.78 (0.72 to 0.85) | -1.41 (-1.55 to -1.27) | 0 | 104.06 (100.34 to 107.81) | 66.03 (60.95 to 71.44) | -1.40 (-1.54 to -1.26) | 0 |
| Neuroblastoma and other peripheral nervous cell tumors | 0.77 (0.64 to 0.92) | 0.61 (0.46 to 0.77) | -0.91 (-1.26 to -0.56) | 0 | 0.23 (0.22 to 0.25) | 0.16 (0.13 to 0.19) | -1.08 (-1.53 to -0.64) | 0 | 20.31 (19.03 to 21.77) | 14.16 (11.65 to 17.14) | -1.29 (-1.74 to -0.84) | 0 |
| Non-Hodgkin lymphoma | 2.10 (1.81 to 2.45) | 2.05 (1.71 to 2.46) | -0.04 (-0.43 to 0.35) | 0.834 | 0.26 (0.24 to 0.27) | 0.11 (0.10 to 0.12) | -2.70 (-2.83 to -2.56) | 0 | 22.20 (20.94 to 23.56) | 10.25 (9.18 to 11.41) | -2.50 (-2.70 to -2.30) | 0 |
| Hodgkin lymphoma | 0.36 (0.32 to 0.40) | 0.22 (0.19 to 0.26) | -1.60 (-1.80 to -1.40) | 0 | 0.03 (0.03 to 0.04) | 0.01 (0.01 to 0.01) | -4.66 (-4.90 to -4.42) | 0 | 2.92 (2.69 to 3.17) | 0.77 (0.67 to 0.89) | -4.36 (-4.60 to -4.12) | 0 |
| Soft tissue and other extraosseous sarcomas | 0.57 (0.51 to 0.62) | 0.55 (0.47 to 0.65) | -0.17 (-0.90 to 0.56) | 0.648 | 0.15 (0.14 to 0.17) | 0.12 (0.10 to 0.14) | -0.87 (-1.55 to -0.20) | 0.011 | 13.10 (12.13 to 14.14) | 10.23 (8.81 to 11.85) | -0.88 (-1.55 to -0.20) | 0.011 |
| Kidney cancer | 0.71 (0.66 to 0.76) | 0.68 (0.60 to 0.76) | -0.24 (-0.93 to 0.46) | 0.503 | 0.15 (0.14 to 0.16) | 0.08 (0.07 to 0.09) | -2.13 (-2.75 to -1.51) | 0 | 13.13 (12.45 to 13.80) | 7.05 (6.30 to 7.81) | -2.14 (-2.81 to -1.47) | 0 |
| Liver cancer | 0.17 (0.16 to 0.18) | 0.21 (0.18 to 0.24) | 0.68 (0.11 to 1.26) | 0.02 | 0.07 (0.07 to 0.08) | 0.05 (0.05 to 0.06) | -1.08 (-1.90 to -0.25) | 0.011 | 6.29 (5.90 to 6.69) | 4.53 (3.97 to 5.12) | -1.06 (-1.87 to -0.24) | 0.011 |
| Malignant neoplasm of bone and articular cartilage | 0.82 (0.74 to 0.91) | 0.57 (0.50 to 0.65) | -1.27 (-1.86 to -0.67) | 0 | 0.19 (0.18 to 0.20) | 0.10 (0.09 to 0.11) | -2.04 (-2.61 to -1.46) | 0 | 15.60 (14.77 to 16.55) | 8.54 (7.69 to 9.44) | -2.02 (-2.58 to -1.45) | 0 |
| Thyroid cancer | 0.14 (0.13 to 0.15) | 0.08 (0.07 to 0.10) | -1.72 (-2.02 to -1.41) | 0 | 0.01 (0.01 to 0.01) | 0.00 (0.00 to 0.00) | -3.59 (-3.90 to -3.28) | 0 | 0.57 (0.53 to 0.62) | 0.21 (0.19 to 0.25) | -3.28 (-3.59 to -2.98) | 0 |
| Eye cancer | 0.50 (0.37 to 0.67) | 0.63 (0.43 to 0.86) | 0.70 (-0.43 to 1.84) | 0.224 | 0.02 (0.02 to 0.02) | 0.01 (0.01 to 0.01) | -2.49 (-3.80 to -1.16) | 0 | 1.92 (1.66 to 2.26) | 1.13 (0.84 to 1.50) | -1.67 (-2.66 to -0.68) | 0.001 |
| Acute myeloid leukemia | 0.55 (0.51 to 0.58) | 0.36 (0.32 to 0.40) | -1.29 (-1.53 to -1.05) | 0 | 0.46 (0.43 to 0.48) | 0.26 (0.23 to 0.29) | -1.77 (-2.04 to -1.50) | 0 | 38.28 (36.10 to 40.74) | 21.63 (19.46 to 24.12) | -1.77 (-2.04 to -1.50) | 0 |
| Chronic myeloid leukemia | 0.12 (0.10 to 0.14) | 0.05 (0.04 to 0.06) | -3.07 (-3.43 to -2.71) | 0 | 0.06 (0.05 to 0.07) | 0.01 (0.01 to 0.01) | -6.27 (-6.70 to -5.85) | 0 | 4.88 (4.24 to 5.77) | 0.70 (0.57 to 0.87) | -6.23 (-6.66 to -5.80) | 0 |
| Nasopharynx cancer | 0.05 (0.04 to 0.05) | 0.03 (0.03 to 0.04) | -1.35 (-1.56 to -1.14) | 0 | 0.01 (0.01 to 0.01) | 0.00 (0.00 to 0.01) | -3.51 (-3.71 to -3.32) | 0 | 1.09 (0.99 to 1.21) | 0.38 (0.33 to 0.45) | -3.44 (-3.63 to -3.25) | 0 |
| Other malignant neoplasms | 1.31 (1.21 to 1.42) | 1.03 (0.86 to 1.25) | -0.91 (-1.58 to -0.24) | 0.008 | 0.42 (0.39 to 0.44) | 0.15 (0.13 to 0.17) | -3.17 (-3.35 to -2.99) | 0 | 35.88 (33.82 to 38.08) | 13.28 (11.63 to 15.20) | -3.10 (-3.28 to -2.91) | 0 |
| Other leukemia | 0.07 (0.05 to 0.08) | 0.04 (0.03 to 0.05) | -1.92 (-2.32 to -1.52) | 0 | 0.01 (0.01 to 0.01) | 0.00 (0.00 to 0.00) | -2.98 (-3.46 to -2.50) | 0 | 0.60 (0.50 to 0.68) | 0.24 (0.18 to 0.30) | -2.87 (-3.36 to -2.38) | 0 |
| **Andean Latin America** | | | | | | | | | | | | |
| Acute lymphoid leukemia | 3.43 (2.54 to 4.95) | 3.51 (2.11 to 5.19) | 0.06 (-0.41 to 0.52) | 0.806 | 3.18 (2.36 to 4.55) | 2.01 (1.30 to 2.76) | -1.45 (-1.96 to -0.93) | 0 | 266.02 (196.63 to 382.95) | 167.17 (107.58 to 229.69) | -1.48 (-1.99 to -0.96) | 0 |
| Brain and central nervous system cancer | 1.84 (1.26 to 2.91) | 2.28 (1.58 to 3.29) | 0.71 (0.32 to 1.11) | 0 | 1.42 (0.99 to 2.23) | 1.32 (0.96 to 1.79) | -0.29 (-0.70 to 0.12) | 0.169 | 119.91 (82.82 to 188.26) | 110.00 (79.73 to 150.18) | -0.32 (-0.73 to 0.09) | 0.13 |
| Neuroblastoma and other peripheral nervous cell tumors | 0.28 (0.16 to 0.43) | 0.22 (0.13 to 0.33) | -0.84 (-1.37 to -0.30) | 0.002 | 0.12 (0.08 to 0.17) | 0.08 (0.05 to 0.11) | -1.38 (-1.78 to -0.97) | 0 | 10.26 (6.88 to 14.52) | 6.64 (4.38 to 9.63) | -1.41 (-1.82 to -1.01) | 0 |
| Non-Hodgkin lymphoma | 1.14 (0.89 to 1.48) | 1.20 (0.80 to 1.77) | 0.15 (-0.35 to 0.66) | 0.549 | 0.80 (0.63 to 1.03) | 0.39 (0.29 to 0.52) | -2.37 (-2.74 to -2.00) | 0 | 67.43 (52.95 to 86.50) | 32.21 (23.83 to 43.29) | -2.39 (-2.75 to -2.02) | 0 |
| Hodgkin lymphoma | 0.14 (0.09 to 0.20) | 0.14 (0.09 to 0.23) | 0.15 (-0.46 to 0.76) | 0.63 | 0.10 (0.06 to 0.14) | 0.04 (0.02 to 0.06) | -2.99 (-3.62 to -2.36) | 0 | 7.78 (4.94 to 11.74) | 3.11 (2.03 to 5.09) | -2.97 (-3.59 to -2.35) | 0 |
| Soft tissue and other extraosseous sarcomas | 0.76 (0.48 to 1.16) | 0.27 (0.17 to 0.40) | -3.27 (-3.71 to -2.84) | 0 | 0.35 (0.23 to 0.52) | 0.09 (0.06 to 0.14) | -4.24 (-4.64 to -3.84) | 0 | 30.31 (19.42 to 44.94) | 7.80 (5.08 to 11.77) | -4.29 (-4.68 to -3.89) | 0 |
| Kidney cancer | 0.64 (0.48 to 0.80) | 0.47 (0.32 to 0.66) | -1.00 (-1.55 to -0.45) | 0 | 0.33 (0.26 to 0.42) | 0.15 (0.10 to 0.20) | -2.62 (-3.09 to -2.15) | 0 | 28.92 (22.11 to 36.26) | 12.62 (8.80 to 17.33) | -2.64 (-3.11 to -2.16) | 0 |
| Liver cancer | 0.29 (0.21 to 0.39) | 0.10 (0.07 to 0.15) | -3.23 (-3.81 to -2.64) | 0 | 0.22 (0.15 to 0.29) | 0.07 (0.05 to 0.11) | -3.23 (-3.89 to -2.56) | 0 | 18.63 (13.19 to 24.97) | 6.26 (4.12 to 9.21) | -3.27 (-3.95 to -2.59) | 0 |
| Malignant neoplasm of bone and articular cartilage | 0.98 (0.67 to 1.46) | 0.72 (0.48 to 1.02) | -0.96 (-1.53 to -0.38) | 0.001 | 0.41 (0.29 to 0.58) | 0.21 (0.15 to 0.30) | -2.09 (-2.61 to -1.57) | 0 | 33.25 (23.54 to 47.95) | 17.59 (12.00 to 24.63) | -2.09 (-2.60 to -1.57) | 0 |
| Thyroid cancer | 0.08 (0.06 to 0.10) | 0.14 (0.10 to 0.20) | 1.81 (0.81 to 2.83) | 0 | 0.01 (0.01 to 0.02) | 0.01 (0.01 to 0.01) | -0.99 (-1.92 to -0.05) | 0.039 | 1.09 (0.85 to 1.39) | 0.82 (0.62 to 1.11) | -0.83 (-1.80 to 0.15) | 0.098 |
| Eye cancer | 0.43 (0.22 to 0.76) | 0.69 (0.35 to 1.21) | 1.55 (1.29 to 1.82) | 0 | 0.32 (0.19 to 0.58) | 0.15 (0.09 to 0.26) | -2.53 (-2.88 to -2.18) | 0 | 28.25 (16.29 to 50.31) | 13.28 (7.56 to 23.05) | -2.48 (-2.82 to -2.14) | 0 |
| Acute myeloid leukemia | 1.03 (0.60 to 1.85) | 0.68 (0.40 to 1.01) | -1.34 (-1.63 to -1.04) | 0 | 1.01 (0.59 to 1.81) | 0.63 (0.37 to 0.94) | -1.51 (-1.81 to -1.21) | 0 | 85.10 (49.54 to 154.31) | 52.73 (30.64 to 78.72) | -1.55 (-1.84 to -1.25) | 0 |
| Chronic myeloid leukemia | 0.13 (0.06 to 0.29) | 0.04 (0.02 to 0.08) | -3.26 (-3.67 to -2.84) | 0 | 0.12 (0.05 to 0.27) | 0.03 (0.02 to 0.06) | -4.16 (-4.57 to -3.74) | 0 | 10.23 (4.55 to 23.26) | 2.69 (1.37 to 4.80) | -4.22 (-4.64 to -3.81) | 0 |
| Nasopharynx cancer | 0.01 (0.01 to 0.02) | 0.01 (0.01 to 0.01) | -0.86 (-1.34 to -0.37) | 0.001 | 0.01 (0.01 to 0.01) | 0.01 (0.00 to 0.01) | -2.14 (-2.58 to -1.69) | 0 | 0.80 (0.59 to 1.08) | 0.41 (0.28 to 0.62) | -2.14 (-2.57 to -1.70) | 0 |
| Other malignant neoplasms | 1.25 (0.83 to 1.72) | 0.67 (0.46 to 0.98) | -1.96 (-2.44 to -1.47) | 0 | 1.03 (0.69 to 1.42) | 0.41 (0.28 to 0.57) | -2.95 (-3.41 to -2.49) | 0 | 87.66 (58.50 to 120.93) | 34.14 (23.73 to 48.25) | -2.99 (-3.47 to -2.51) | 0 |
| Other leukemia | 0.15 (0.07 to 0.29) | 0.09 (0.04 to 0.17) | -1.80 (-2.30 to -1.30) | 0 | 0.06 (0.03 to 0.11) | 0.01 (0.01 to 0.03) | -4.17 (-4.47 to -3.87) | 0 | 5.02 (2.61 to 9.50) | 1.37 (0.62 to 2.46) | -4.12 (-4.42 to -3.82) | 0 |
| **Caribbean** | | | | | | | | | | | | |
| Acute lymphoid leukemia | 2.94 (1.98 to 4.48) | 2.28 (1.47 to 3.54) | -0.73 (-1.20 to -0.25) | 0.003 | 2.49 (1.58 to 3.98) | 1.81 (1.05 to 3.08) | -0.90 (-1.05 to -0.74) | 0 | 210.14 (132.56 to 337.23) | 152.17 (87.54 to 259.55) | -0.95 (-1.17 to -0.73) | 0 |
| Brain and central nervous system cancer | 1.61 (1.18 to 2.87) | 1.73 (1.23 to 2.62) | 0.34 (-0.08 to 0.76) | 0.112 | 1.15 (0.81 to 2.19) | 1.19 (0.83 to 1.91) | 0.18 (-0.01 to 0.38) | 0.059 | 96.77 (68.02 to 186.96) | 99.80 (69.09 to 161.58) | 0.17 (-0.02 to 0.36) | 0.079 |
| Neuroblastoma and other peripheral nervous cell tumors | 0.32 (0.20 to 0.50) | 0.49 (0.30 to 0.77) | 1.58 (1.00 to 2.17) | 0 | 0.12 (0.09 to 0.18) | 0.19 (0.12 to 0.29) | 1.48 (0.92 to 2.05) | 0 | 11.03 (7.79 to 16.14) | 16.44 (10.72 to 25.09) | 1.47 (0.91 to 2.04) | 0 |
| Non-Hodgkin lymphoma | 2.02 (1.36 to 2.66) | 1.61 (1.03 to 2.30) | -0.62 (-0.76 to -0.48) | 0 | 1.24 (0.72 to 1.74) | 0.80 (0.45 to 1.23) | -1.32 (-1.67 to -0.97) | 0 | 104.85 (60.21 to 148.33) | 67.72 (37.28 to 104.13) | -1.33 (-1.68 to -0.99) | 0 |
| Hodgkin lymphoma | 0.14 (0.10 to 0.18) | 0.09 (0.06 to 0.12) | -1.35 (-1.82 to -0.89) | 0 | 0.06 (0.04 to 0.08) | 0.03 (0.02 to 0.06) | -1.67 (-1.83 to -1.50) | 0 | 4.89 (3.41 to 6.90) | 2.84 (1.60 to 4.78) | -1.66 (-1.83 to -1.50) | 0 |
| Soft tissue and other extraosseous sarcomas | 1.14 (0.71 to 1.71) | 0.99 (0.55 to 1.67) | -0.34 (-0.49 to -0.20) | 0 | 0.58 (0.34 to 0.87) | 0.46 (0.25 to 0.78) | -0.68 (-0.83 to -0.54) | 0 | 49.71 (29.69 to 75.41) | 39.12 (21.40 to 67.15) | -0.70 (-0.84 to -0.56) | 0 |
| Kidney cancer | 0.65 (0.44 to 0.87) | 0.57 (0.39 to 0.86) | -0.39 (-0.90 to 0.14) | 0.147 | 0.33 (0.21 to 0.46) | 0.24 (0.15 to 0.39) | -0.89 (-1.11 to -0.66) | 0 | 28.19 (18.02 to 39.68) | 20.76 (13.04 to 33.32) | -0.89 (-1.12 to -0.66) | 0 |
| Liver cancer | 0.11 (0.07 to 0.16) | 0.07 (0.04 to 0.11) | -1.39 (-1.69 to -1.09) | 0 | 0.08 (0.05 to 0.12) | 0.05 (0.03 to 0.08) | -1.38 (-1.48 to -1.28) | 0 | 7.00 (4.45 to 10.13) | 4.44 (2.74 to 7.03) | -1.43 (-1.79 to -1.07) | 0 |
| Malignant neoplasm of bone and articular cartilage | 0.65 (0.46 to 0.93) | 0.61 (0.40 to 0.95) | -0.07 (-0.69 to 0.56) | 0.833 | 0.24 (0.17 to 0.36) | 0.21 (0.14 to 0.34) | -0.47 (-0.95 to 0.01) | 0.055 | 20.12 (14.05 to 29.55) | 17.31 (11.13 to 27.96) | -0.39 (-0.89 to 0.11) | 0.127 |
| Thyroid cancer | 0.08 (0.06 to 0.10) | 0.08 (0.06 to 0.11) | 0.24 (-0.05 to 0.53) | 0.099 | 0.01 (0.01 to 0.02) | 0.01 (0.01 to 0.01) | -0.48 (-0.67 to -0.28) | 0 | 0.98 (0.68 to 1.36) | 0.84 (0.53 to 1.20) | -0.44 (-0.64 to -0.24) | 0 |
| Eye cancer | 0.21 (0.12 to 0.35) | 0.15 (0.08 to 0.27) | -0.73 (-1.58 to 0.13) | 0.097 | 0.05 (0.03 to 0.08) | 0.02 (0.01 to 0.04) | -2.65 (-3.99 to -1.29) | 0 | 4.67 (2.95 to 6.94) | 1.96 (1.00 to 3.54) | -2.65 (-4.00 to -1.27) | 0 |
| Acute myeloid leukemia | 0.96 (0.61 to 1.78) | 0.71 (0.41 to 1.30) | -0.92 (-1.35 to -0.49) | 0 | 0.92 (0.59 to 1.71) | 0.68 (0.39 to 1.26) | -0.94 (-1.37 to -0.51) | 0 | 77.97 (49.14 to 145.11) | 57.02 (32.62 to 106.72) | -0.96 (-1.39 to -0.53) | 0 |
| Chronic myeloid leukemia | 0.22 (0.05 to 0.70) | 0.12 (0.03 to 0.37) | -1.85 (-2.00 to -1.71) | 0 | 0.21 (0.05 to 0.66) | 0.11 (0.02 to 0.33) | -1.98 (-2.13 to -1.83) | 0 | 18.19 (3.98 to 57.57) | 9.60 (1.95 to 28.61) | -1.99 (-2.14 to -1.84) | 0 |
| Nasopharynx cancer | 0.03 (0.02 to 0.04) | 0.03 (0.02 to 0.04) | -0.08 (-0.34 to 0.18) | 0.548 | 0.02 (0.02 to 0.03) | 0.02 (0.01 to 0.03) | -0.37 (-0.55 to -0.20) | 0 | 1.87 (1.38 to 2.54) | 1.66 (1.09 to 2.40) | -0.38 (-0.56 to -0.20) | 0 |
| Other malignant neoplasms | 1.29 (0.73 to 1.91) | 1.00 (0.57 to 1.51) | -0.76 (-0.86 to -0.66) | 0 | 0.91 (0.46 to 1.42) | 0.69 (0.36 to 1.12) | -0.84 (-0.97 to -0.72) | 0 | 78.28 (39.36 to 121.85) | 59.07 (30.31 to 95.54) | -0.86 (-0.99 to -0.73) | 0 |
| Other leukemia | 0.22 (0.10 to 0.48) | 0.16 (0.06 to 0.37) | -0.91 (-1.08 to -0.73) | 0 | 0.08 (0.03 to 0.21) | 0.05 (0.02 to 0.12) | -1.52 (-1.70 to -1.34) | 0 | 7.48 (2.74 to 18.28) | 4.67 (1.49 to 11.07) | -1.51 (-1.69 to -1.33) | 0 |
| **Central Latin America** | | | | | | | | | | | | |
| Acute lymphoid leukemia | 3.46 (3.19 to 3.76) | 2.88 (2.34 to 3.62) | -0.70 (-1.25 to -0.14) | 0.014 | 3.03 (2.80 to 3.28) | 1.70 (1.43 to 2.06) | -1.89 (-2.23 to -1.54) | 0 | 253.01 (233.87 to 274.74) | 140.98 (118.10 to 171.02) | -1.93 (-2.28 to -1.56) | 0 |
| Brain and central nervous system cancer | 1.37 (1.26 to 1.51) | 1.30 (1.07 to 1.59) | -0.22 (-0.58 to 0.13) | 0.214 | 1.00 (0.92 to 1.09) | 0.76 (0.63 to 0.92) | -0.93 (-1.30 to -0.57) | 0 | 84.10 (77.71 to 91.72) | 63.33 (52.61 to 77.04) | -0.97 (-1.34 to -0.60) | 0 |
| Neuroblastoma and other peripheral nervous cell tumors | 0.28 (0.20 to 0.38) | 0.27 (0.18 to 0.38) | -0.24 (-0.65 to 0.17) | 0.257 | 0.12 (0.10 to 0.13) | 0.09 (0.07 to 0.12) | -0.80 (-1.27 to -0.32) | 0.001 | 10.11 (9.03 to 11.34) | 8.06 (6.06 to 10.71) | -0.81 (-1.29 to -0.34) | 0.001 |
| Non-Hodgkin lymphoma | 0.88 (0.79 to 0.98) | 0.75 (0.61 to 0.93) | -0.53 (-1.04 to -0.01) | 0.045 | 0.52 (0.48 to 0.57) | 0.23 (0.19 to 0.28) | -2.73 (-3.28 to -2.18) | 0 | 44.03 (40.65 to 48.03) | 19.26 (16.08 to 23.18) | -2.75 (-3.29 to -2.20) | 0 |
| Hodgkin lymphoma | 0.30 (0.28 to 0.33) | 0.18 (0.15 to 0.21) | -1.70 (-2.46 to -0.93) | 0 | 0.17 (0.16 to 0.19) | 0.04 (0.04 to 0.05) | -4.43 (-5.03 to -3.82) | 0 | 14.01 (12.87 to 15.28) | 3.45 (2.96 to 4.04) | -4.42 (-5.03 to -3.81) | 0 |
| Soft tissue and other extraosseous sarcomas | 0.47 (0.41 to 0.55) | 0.32 (0.26 to 0.40) | -1.18 (-1.96 to -0.39) | 0.003 | 0.20 (0.18 to 0.23) | 0.11 (0.09 to 0.13) | -1.93 (-2.60 to -1.26) | 0 | 17.20 (15.52 to 19.25) | 9.06 (7.43 to 11.15) | -1.97 (-2.64 to -1.30) | 0 |
| Kidney cancer | 0.60 (0.56 to 0.67) | 0.44 (0.35 to 0.55) | -1.14 (-1.96 to -0.31) | 0.007 | 0.29 (0.27 to 0.32) | 0.14 (0.11 to 0.17) | -2.55 (-3.38 to -1.71) | 0 | 25.45 (23.51 to 27.91) | 11.80 (9.43 to 14.83) | -2.55 (-3.39 to -1.71) | 0 |
| Liver cancer | 0.28 (0.26 to 0.31) | 0.16 (0.12 to 0.21) | -1.76 (-1.95 to -1.58) | 0 | 0.21 (0.19 to 0.23) | 0.11 (0.08 to 0.14) | -1.93 (-2.10 to -1.75) | 0 | 17.95 (16.54 to 19.69) | 9.35 (7.28 to 12.06) | -1.95 (-2.13 to -1.78) | 0 |
| Malignant neoplasm of bone and articular cartilage | 0.54 (0.47 to 0.61) | 0.59 (0.51 to 0.70) | 0.36 (-0.38 to 1.11) | 0.342 | 0.20 (0.19 to 0.22) | 0.18 (0.16 to 0.21) | -0.43 (-1.17 to 0.31) | 0.251 | 16.76 (15.40 to 18.25) | 14.63 (12.82 to 16.87) | -0.45 (-1.19 to 0.28) | 0.228 |
| Thyroid cancer | 0.09 (0.08 to 0.09) | 0.10 (0.09 to 0.11) | 0.34 (-0.43 to 1.12) | 0.387 | 0.01 (0.01 to 0.01) | 0.01 (0.01 to 0.01) | -1.95 (-2.52 to -1.38) | 0 | 1.03 (0.96 to 1.11) | 0.59 (0.52 to 0.68) | -1.83 (-2.38 to -1.27) | 0 |
| Eye cancer | 0.32 (0.21 to 0.46) | 0.35 (0.22 to 0.56) | 0.30 (-0.01 to 0.63) | 0.062 | 0.15 (0.12 to 0.18) | 0.07 (0.05 to 0.10) | -2.77 (-3.50 to -2.04) | 0 | 12.94 (10.86 to 15.52) | 5.97 (4.04 to 8.61) | -2.72 (-3.44 to -2.00) | 0 |
| Acute myeloid leukemia | 0.88 (0.79 to 0.98) | 0.52 (0.43 to 0.64) | -1.76 (-2.45 to -1.07) | 0 | 0.85 (0.77 to 0.95) | 0.48 (0.40 to 0.59) | -1.90 (-2.60 to -1.19) | 0 | 71.82 (64.69 to 79.87) | 40.12 (32.86 to 49.44) | -1.94 (-2.65 to -1.22) | 0 |
| Chronic myeloid leukemia | 0.10 (0.09 to 0.11) | 0.03 (0.02 to 0.03) | -4.14 (-4.67 to -3.61) | 0 | 0.09 (0.08 to 0.11) | 0.02 (0.01 to 0.02) | -5.05 (-5.49 to -4.60) | 0 | 7.70 (6.77 to 8.88) | 1.57 (1.21 to 2.04) | -5.09 (-5.53 to -4.65) | 0 |
| Nasopharynx cancer | 0.02 (0.01 to 0.02) | 0.01 (0.01 to 0.01) | -1.48 (-1.80 to -1.17) | 0 | 0.01 (0.01 to 0.01) | 0.01 (0.00 to 0.01) | -2.59 (-2.91 to -2.28) | 0 | 0.97 (0.90 to 1.07) | 0.44 (0.37 to 0.52) | -2.61 (-2.92 to -2.29) | 0 |
| Other malignant neoplasms | 1.22 (1.14 to 1.31) | 0.65 (0.52 to 0.83) | -1.99 (-2.47 to -1.52) | 0 | 0.93 (0.87 to 1.00) | 0.37 (0.31 to 0.46) | -2.90 (-3.46 to -2.34) | 0 | 79.11 (74.12 to 84.98) | 31.57 (25.69 to 39.15) | -2.93 (-3.49 to -2.37) | 0 |
| Other leukemia | 0.02 (0.02 to 0.03) | 0.01 (0.01 to 0.02) | -2.25 (-2.58 to -1.92) | 0 | 0.01 (0.01 to 0.01) | 0.00 (0.00 to 0.00) | -4.12 (-4.59 to -3.66) | 0 | 0.69 (0.58 to 0.81) | 0.19 (0.13 to 0.27) | -4.09 (-4.56 to -3.63) | 0 |
| **Tropical Latin America** | | | | | | | | | | | | |
| Acute lymphoid leukemia | 1.86 (1.61 to 2.10) | 1.41 (1.12 to 1.75) | -0.94 (-1.69 to -0.20) | 0.013 | 1.62 (1.40 to 1.83) | 0.87 (0.69 to 1.04) | -2.03 (-2.77 to -1.28) | 0 | 135.72 (117.21 to 153.94) | 72.49 (57.48 to 87.03) | -2.07 (-2.81 to -1.32) | 0 |
| Brain and central nervous system cancer | 2.01 (1.70 to 2.34) | 1.88 (1.51 to 2.25) | -0.24 (-0.61 to 0.13) | 0.197 | 1.46 (1.23 to 1.69) | 1.13 (0.91 to 1.35) | -0.85 (-1.20 to -0.50) | 0 | 123.38 (104.05 to 143.19) | 94.57 (75.60 to 112.88) | -0.89 (-1.25 to -0.54) | 0 |
| Neuroblastoma and other peripheral nervous cell tumors | 0.47 (0.33 to 0.64) | 0.49 (0.34 to 0.68) | 0.04 (-0.43 to 0.51) | 0.877 | 0.19 (0.16 to 0.22) | 0.17 (0.13 to 0.22) | -0.39 (-0.75 to -0.03) | 0.035 | 16.89 (14.14 to 19.73) | 15.12 (11.33 to 19.14) | -0.42 (-0.78 to -0.06) | 0.024 |
| Non-Hodgkin lymphoma | 0.91 (0.79 to 1.03) | 0.58 (0.46 to 0.71) | -1.39 (-1.80 to -0.98) | 0 | 0.56 (0.49 to 0.63) | 0.20 (0.16 to 0.24) | -3.20 (-3.72 to -2.67) | 0 | 46.96 (41.32 to 52.76) | 16.85 (13.39 to 20.16) | -3.23 (-3.57 to -2.88) | 0 |
| Hodgkin lymphoma | 0.15 (0.13 to 0.17) | 0.09 (0.08 to 0.11) | -1.55 (-2.45 to -0.64) | 0.001 | 0.08 (0.07 to 0.10) | 0.02 (0.02 to 0.03) | -3.94 (-4.36 to -3.52) | 0 | 6.74 (5.84 to 7.73) | 1.96 (1.61 to 2.34) | -3.94 (-4.36 to -3.52) | 0 |
| Soft tissue and other extraosseous sarcomas | 0.39 (0.33 to 0.47) | 0.34 (0.26 to 0.42) | -0.49 (-1.18 to 0.19) | 0.158 | 0.17 (0.14 to 0.19) | 0.12 (0.09 to 0.14) | -1.20 (-1.86 to -0.53) | 0 | 14.20 (12.18 to 16.29) | 9.63 (7.49 to 11.94) | -1.25 (-1.92 to -0.59) | 0 |
| Kidney cancer | 0.62 (0.54 to 0.69) | 0.45 (0.36 to 0.55) | -1.06 (-1.57 to -0.55) | 0 | 0.30 (0.26 to 0.34) | 0.14 (0.11 to 0.17) | -2.40 (-3.03 to -1.76) | 0 | 26.00 (22.70 to 29.24) | 12.39 (9.81 to 15.00) | -2.42 (-3.05 to -1.79) | 0 |
| Liver cancer | 0.17 (0.14 to 0.20) | 0.09 (0.07 to 0.11) | -2.19 (-2.57 to -1.80) | 0 | 0.12 (0.10 to 0.14) | 0.06 (0.05 to 0.08) | -2.31 (-2.66 to -1.95) | 0 | 10.53 (9.09 to 12.40) | 5.31 (4.13 to 6.50) | -2.34 (-2.70 to -1.98) | 0 |
| Malignant neoplasm of bone and articular cartilage | 0.62 (0.54 to 0.72) | 0.58 (0.47 to 0.69) | -0.22 (-0.83 to 0.39) | 0.484 | 0.24 (0.21 to 0.26) | 0.18 (0.15 to 0.21) | -0.99 (-1.63 to -0.34) | 0.003 | 19.44 (17.25 to 21.74) | 14.54 (12.14 to 16.93) | -1.01 (-1.64 to -0.38) | 0.002 |
| Thyroid cancer | 0.08 (0.07 to 0.09) | 0.09 (0.07 to 0.10) | 0.24 (-0.33 to 0.82) | 0.408 | 0.01 (0.01 to 0.01) | 0.01 (0.01 to 0.01) | -1.64 (-2.02 to -1.25) | 0 | 0.97 (0.87 to 1.08) | 0.57 (0.48 to 0.67) | -1.54 (-1.95 to -1.13) | 0 |
| Eye cancer | 0.24 (0.16 to 0.35) | 0.20 (0.12 to 0.31) | -0.46 (-0.78 to -0.14) | 0.005 | 0.13 (0.10 to 0.17) | 0.04 (0.03 to 0.06) | -3.57 (-4.00 to -3.14) | 0 | 11.37 (8.74 to 14.66) | 3.71 (2.45 to 5.20) | -3.54 (-3.97 to -3.10) | 0 |
| Acute myeloid leukemia | 0.84 (0.73 to 0.97) | 0.41 (0.33 to 0.50) | -2.28 (-2.82 to -1.74) | 0 | 0.82 (0.70 to 0.94) | 0.39 (0.30 to 0.46) | -2.54 (-3.04 to -2.03) | 0 | 69.00 (59.05 to 79.05) | 32.07 (25.27 to 38.46) | -2.58 (-3.08 to -2.08) | 0 |
| Chronic myeloid leukemia | 0.08 (0.07 to 0.10) | 0.02 (0.01 to 0.02) | -5.28 (-5.66 to -4.90) | 0 | 0.08 (0.06 to 0.09) | 0.01 (0.01 to 0.02) | -5.98 (-6.39 to -5.57) | 0 | 6.46 (5.46 to 7.59) | 0.95 (0.70 to 1.26) | -6.04 (-6.45 to -5.63) | 0 |
| Nasopharynx cancer | 0.02 (0.01 to 0.02) | 0.02 (0.01 to 0.02) | -0.14 (-0.68 to 0.40) | 0.606 | 0.01 (0.01 to 0.01) | 0.01 (0.01 to 0.01) | -1.07 (-1.57 to -0.58) | 0 | 1.00 (0.87 to 1.16) | 0.72 (0.59 to 0.88) | -1.09 (-1.58 to -0.59) | 0 |
| Other malignant neoplasms | 0.82 (0.70 to 0.93) | 0.43 (0.34 to 0.51) | -2.02 (-2.37 to -1.67) | 0 | 0.62 (0.53 to 0.71) | 0.26 (0.21 to 0.31) | -2.75 (-3.09 to -2.41) | 0 | 52.94 (45.46 to 60.36) | 21.60 (17.15 to 25.98) | -2.82 (-3.16 to -2.47) | 0 |
| Other leukemia | 0.04 (0.03 to 0.05) | 0.02 (0.02 to 0.03) | -2.14 (-2.49 to -1.78) | 0 | 0.01 (0.01 to 0.02) | 0.00 (0.00 to 0.01) | -3.82 (-4.16 to -3.48) | 0 | 1.22 (0.96 to 1.47) | 0.37 (0.27 to 0.48) | -3.82 (-4.16 to -3.48) | 0 |
| **North Africa and Middle East** | | | | | | | | | | | | |
| Acute lymphoid leukemia | 2.50 (1.54 to 3.81) | 1.83 (1.04 to 2.48) | -1.03 (-1.19 to -0.86) | 0 | 2.16 (1.31 to 3.30) | 1.01 (0.55 to 1.37) | -2.43 (-2.55 to -2.31) | 0 | 181.15 (109.95 to 277.69) | 83.92 (46.23 to 114.88) | -2.45 (-2.56 to -2.33) | 0 |
| Brain and central nervous system cancer | 2.20 (1.45 to 3.34) | 2.73 (1.91 to 3.55) | 0.67 (0.48 to 0.87) | 0 | 1.39 (0.94 to 2.13) | 1.16 (0.82 to 1.47) | -0.58 (-0.66 to -0.51) | 0 | 116.73 (78.42 to 180.15) | 97.27 (68.83 to 123.46) | -0.60 (-0.67 to -0.52) | 0 |
| Neuroblastoma and other peripheral nervous cell tumors | 0.17 (0.10 to 0.28) | 0.20 (0.13 to 0.29) | 0.54 (0.03 to 1.06) | 0.039 | 0.07 (0.05 to 0.10) | 0.06 (0.04 to 0.09) | -0.26 (-0.42 to -0.10) | 0.001 | 6.05 (4.00 to 8.98) | 5.55 (3.84 to 7.86) | -0.28 (-0.44 to -0.12) | 0.001 |
| Non-Hodgkin lymphoma | 1.09 (0.79 to 1.44) | 1.00 (0.79 to 1.30) | -0.27 (-0.55 to 0.01) | 0.063 | 0.59 (0.42 to 0.80) | 0.25 (0.20 to 0.34) | -2.63 (-2.75 to -2.51) | 0 | 49.53 (35.49 to 67.57) | 21.45 (17.22 to 28.38) | -2.61 (-2.73 to -2.49) | 0 |
| Hodgkin lymphoma | 0.21 (0.12 to 0.31) | 0.23 (0.13 to 0.32) | 0.21 (-0.03 to 0.45) | 0.089 | 0.12 (0.07 to 0.17) | 0.05 (0.03 to 0.08) | -2.51 (-2.82 to -2.20) | 0 | 9.50 (5.43 to 13.67) | 4.36 (2.57 to 6.31) | -2.48 (-2.79 to -2.16) | 0 |
| Soft tissue and other extraosseous sarcomas | 0.68 (0.47 to 1.02) | 0.23 (0.16 to 0.35) | -3.46 (-3.64 to -3.28) | 0 | 0.29 (0.20 to 0.43) | 0.08 (0.05 to 0.12) | -4.08 (-4.24 to -3.92) | 0 | 25.17 (17.45 to 36.77) | 6.78 (4.65 to 10.50) | -4.12 (-4.27 to -3.96) | 0 |
| Kidney cancer | 0.81 (0.49 to 1.17) | 0.62 (0.47 to 0.79) | -0.92 (-1.15 to -0.69) | 0 | 0.21 (0.13 to 0.28) | 0.11 (0.08 to 0.14) | -2.05 (-2.15 to -1.95) | 0 | 18.18 (11.73 to 25.14) | 9.61 (7.51 to 12.18) | -2.05 (-2.14 to -1.95) | 0 |
| Liver cancer | 0.36 (0.26 to 0.48) | 0.20 (0.16 to 0.26) | -1.92 (-2.22 to -1.63) | 0 | 0.27 (0.19 to 0.35) | 0.14 (0.11 to 0.18) | -2.15 (-2.44 to -1.87) | 0 | 23.35 (16.63 to 30.73) | 11.93 (9.20 to 15.60) | -2.17 (-2.45 to -1.88) | 0 |
| Malignant neoplasm of bone and articular cartilage | 0.62 (0.36 to 0.85) | 0.60 (0.46 to 0.78) | -0.09 (-0.21 to 0.04) | 0.189 | 0.24 (0.14 to 0.32) | 0.18 (0.14 to 0.23) | -0.92 (-1.01 to -0.82) | 0 | 19.69 (11.61 to 26.48) | 14.68 (11.37 to 18.77) | -0.90 (-1.00 to -0.81) | 0 |
| Thyroid cancer | 0.10 (0.07 to 0.15) | 0.14 (0.11 to 0.17) | 1.06 (0.86 to 1.27) | 0 | 0.01 (0.00 to 0.01) | 0.00 (0.00 to 0.01) | -0.93 (-1.23 to -0.63) | 0 | 0.58 (0.42 to 0.95) | 0.46 (0.36 to 0.57) | -0.70 (-0.99 to -0.41) | 0 |
| Eye cancer | 0.09 (0.05 to 0.15) | 0.14 (0.08 to 0.23) | 1.46 (1.11 to 1.82) | 0 | 0.05 (0.03 to 0.09) | 0.03 (0.01 to 0.04) | -2.47 (-2.91 to -2.03) | 0 | 4.75 (2.84 to 7.62) | 2.25 (1.34 to 3.72) | -2.40 (-2.83 to -1.97) | 0 |
| Acute myeloid leukemia | 0.96 (0.53 to 1.84) | 0.54 (0.37 to 0.85) | -1.83 (-1.96 to -1.69) | 0 | 0.93 (0.52 to 1.78) | 0.50 (0.34 to 0.79) | -1.99 (-2.11 to -1.87) | 0 | 77.97 (43.39 to 150.93) | 41.55 (28.44 to 65.95) | -2.02 (-2.14 to -1.91) | 0 |
| Chronic myeloid leukemia | 0.23 (0.08 to 0.46) | 0.09 (0.03 to 0.16) | -2.87 (-3.01 to -2.72) | 0 | 0.21 (0.07 to 0.43) | 0.07 (0.02 to 0.12) | -3.48 (-3.71 to -3.25) | 0 | 17.99 (6.27 to 36.70) | 5.91 (1.66 to 10.23) | -3.52 (-3.75 to -3.28) | 0 |
| Nasopharynx cancer | 0.06 (0.05 to 0.08) | 0.03 (0.03 to 0.05) | -1.79 (-1.96 to -1.62) | 0 | 0.05 (0.04 to 0.06) | 0.02 (0.01 to 0.02) | -3.11 (-3.23 to -2.99) | 0 | 3.73 (2.92 to 4.73) | 1.41 (1.03 to 1.87) | -3.11 (-3.23 to -2.99) | 0 |
| Other malignant neoplasms | 1.32 (0.69 to 1.81) | 1.23 (0.84 to 1.68) | -0.27 (-0.51 to -0.04) | 0.022 | 0.78 (0.41 to 1.08) | 0.42 (0.28 to 0.55) | -1.98 (-2.14 to -1.81) | 0 | 66.40 (34.70 to 92.04) | 35.69 (24.15 to 47.05) | -1.99 (-2.16 to -1.82) | 0 |
| Other leukemia | 0.37 (0.19 to 0.72) | 0.29 (0.14 to 0.54) | -0.84 (-1.11 to -0.57) | 0 | 0.12 (0.06 to 0.25) | 0.06 (0.03 to 0.12) | -2.46 (-2.57 to -2.36) | 0 | 10.91 (5.65 to 21.77) | 5.05 (2.50 to 10.05) | -2.44 (-2.55 to -2.32) | 0 |
| **South Asia** | | | | | | | | | | | | |
| Acute lymphoid leukemia | 1.46 (0.83 to 2.44) | 0.84 (0.59 to 1.13) | -1.79 (-2.37 to -1.21) | 0 | 1.41 (0.80 to 2.39) | 0.71 (0.50 to 0.95) | -2.24 (-2.82 to -1.65) | 0 | 118.22 (66.57 to 200.48) | 58.79 (41.38 to 78.99) | -2.28 (-2.89 to -1.68) | 0 |
| Brain and central nervous system cancer | 0.96 (0.56 to 1.53) | 0.99 (0.74 to 1.35) | 0.10 (-0.22 to 0.42) | 0.534 | 0.80 (0.47 to 1.27) | 0.71 (0.53 to 0.96) | -0.37 (-0.67 to -0.08) | 0.013 | 67.11 (39.22 to 107.53) | 59.34 (44.24 to 80.95) | -0.39 (-0.68 to -0.10) | 0.008 |
| Neuroblastoma and other peripheral nervous cell tumors | 0.17 (0.09 to 0.27) | 0.31 (0.18 to 0.49) | 1.97 (1.68 to 2.26) | 0 | 0.08 (0.05 to 0.11) | 0.12 (0.08 to 0.18) | 1.38 (1.02 to 1.73) | 0 | 6.97 (4.56 to 9.69) | 10.87 (7.23 to 15.79) | 1.38 (1.02 to 1.74) | 0 |
| Non-Hodgkin lymphoma | 1.02 (0.62 to 1.34) | 0.89 (0.65 to 1.23) | -0.45 (-0.85 to -0.06) | 0.023 | 0.73 (0.44 to 0.96) | 0.42 (0.32 to 0.58) | -1.75 (-2.15 to -1.35) | 0 | 61.29 (36.93 to 80.90) | 35.39 (26.39 to 48.88) | -1.77 (-2.17 to -1.36) | 0 |
| Hodgkin lymphoma | 0.31 (0.16 to 0.46) | 0.27 (0.17 to 0.42) | -0.45 (-0.74 to -0.15) | 0.003 | 0.25 (0.12 to 0.36) | 0.14 (0.08 to 0.21) | -1.84 (-2.21 to -1.45) | 0 | 20.09 (10.01 to 29.47) | 11.07 (6.86 to 17.20) | -1.84 (-2.23 to -1.45) | 0 |
| Soft tissue and other extraosseous sarcomas | 0.62 (0.40 to 0.87) | 0.30 (0.19 to 0.48) | -2.32 (-2.67 to -1.96) | 0 | 0.32 (0.21 to 0.43) | 0.12 (0.08 to 0.20) | -2.94 (-3.30 to -2.58) | 0 | 27.00 (17.72 to 36.49) | 10.57 (6.95 to 16.72) | -2.97 (-3.31 to -2.62) | 0 |
| Kidney cancer | 0.18 (0.11 to 0.25) | 0.22 (0.15 to 0.30) | 0.56 (0.21 to 0.92) | 0.002 | 0.11 (0.06 to 0.15) | 0.09 (0.06 to 0.13) | -0.41 (-0.71 to -0.11) | 0.007 | 9.34 (5.59 to 13.05) | 8.22 (5.53 to 11.33) | -0.41 (-0.71 to -0.11) | 0.008 |
| Liver cancer | 0.27 (0.17 to 0.37) | 0.19 (0.14 to 0.24) | -1.20 (-1.44 to -0.97) | 0 | 0.20 (0.12 to 0.27) | 0.14 (0.10 to 0.18) | -1.29 (-1.57 to -1.00) | 0 | 17.53 (10.70 to 23.69) | 11.87 (9.12 to 15.31) | -1.30 (-1.60 to -1.00) | 0 |
| Malignant neoplasm of bone and articular cartilage | 0.51 (0.31 to 0.71) | 0.56 (0.41 to 0.72) | 0.23 (-0.02 to 0.48) | 0.075 | 0.24 (0.15 to 0.33) | 0.21 (0.16 to 0.27) | -0.48 (-0.88 to -0.08) | 0.018 | 19.93 (12.27 to 27.35) | 17.03 (13.06 to 21.71) | -0.49 (-0.89 to -0.10) | 0.014 |
| Thyroid cancer | 0.05 (0.04 to 0.06) | 0.09 (0.06 to 0.11) | 1.82 (1.25 to 2.39) | 0 | 0.01 (0.01 to 0.01) | 0.01 (0.01 to 0.01) | -0.08 (-0.54 to 0.38) | 0.73 | 0.80 (0.60 to 1.01) | 0.77 (0.54 to 1.01) | -0.04 (-0.40 to 0.32) | 0.82 |
| Eye cancer | 0.22 (0.10 to 0.34) | 0.28 (0.14 to 0.46) | 0.88 (0.63 to 1.13) | 0 | 0.21 (0.10 to 0.32) | 0.15 (0.08 to 0.23) | -1.09 (-1.31 to -0.87) | 0 | 18.29 (8.63 to 28.07) | 12.91 (7.00 to 19.95) | -1.08 (-1.29 to -0.86) | 0 |
| Acute myeloid leukemia | 0.54 (0.26 to 1.26) | 0.40 (0.27 to 0.60) | -0.96 (-1.12 to -0.79) | 0 | 0.53 (0.26 to 1.24) | 0.39 (0.26 to 0.58) | -1.02 (-1.18 to -0.85) | 0 | 45.45 (21.77 to 106.24) | 32.72 (21.87 to 48.81) | -1.04 (-1.22 to -0.86) | 0 |
| Chronic myeloid leukemia | 0.14 (0.04 to 0.33) | 0.04 (0.02 to 0.07) | -3.85 (-4.09 to -3.61) | 0 | 0.14 (0.04 to 0.32) | 0.04 (0.02 to 0.06) | -4.16 (-4.40 to -3.92) | 0 | 11.48 (3.48 to 26.90) | 2.98 (1.56 to 4.94) | -4.23 (-4.47 to -3.99) | 0 |
| Nasopharynx cancer | 0.09 (0.06 to 0.11) | 0.06 (0.05 to 0.08) | -1.04 (-1.54 to -0.53) | 0 | 0.08 (0.06 to 0.10) | 0.05 (0.04 to 0.06) | -1.57 (-2.10 to -1.05) | 0 | 6.22 (4.58 to 8.07) | 3.68 (2.80 to 4.84) | -1.59 (-2.11 to -1.06) | 0 |
| Other malignant neoplasms | 0.89 (0.50 to 1.38) | 0.75 (0.57 to 0.94) | -0.56 (-0.87 to -0.24) | 0.001 | 0.79 (0.44 to 1.23) | 0.58 (0.44 to 0.73) | -1.00 (-1.30 to -0.69) | 0 | 66.95 (37.47 to 104.77) | 48.91 (37.35 to 61.93) | -1.01 (-1.31 to -0.71) | 0 |
| Other leukemia | 0.35 (0.14 to 0.74) | 0.25 (0.12 to 0.42) | -1.09 (-1.29 to -0.89) | 0 | 0.16 (0.06 to 0.35) | 0.07 (0.04 to 0.13) | -2.58 (-2.82 to -2.35) | 0 | 14.20 (5.60 to 30.03) | 6.29 (3.15 to 10.95) | -2.59 (-2.82 to -2.37) | 0 |
| **East Asia** | | | | | | | | | | | | |
| Acute lymphoid leukemia | 6.20 (4.17 to 8.67) | 7.52 (3.91 to 11.40) | 0.44 (-0.02 to 0.89) | 0.061 | 5.02 (3.36 to 7.00) | 1.20 (0.70 to 1.68) | -4.63 (-4.92 to -4.35) | 0 | 427.79 (286.23 to 598.58) | 104.72 (60.50 to 147.44) | -4.58 (-4.87 to -4.29) | 0 |
| Brain and central nervous system cancer | 3.78 (2.50 to 4.87) | 3.26 (2.40 to 4.57) | -0.61 (-0.86 to -0.36) | 0 | 2.63 (1.74 to 3.41) | 1.13 (0.82 to 1.56) | -2.86 (-3.13 to -2.59) | 0 | 224.31 (147.60 to 290.73) | 95.00 (69.03 to 132.14) | -2.89 (-3.16 to -2.62) | 0 |
| Neuroblastoma and other peripheral nervous cell tumors | 0.15 (0.10 to 0.23) | 0.22 (0.15 to 0.31) | 1.00 (0.64 to 1.36) | 0 | 0.06 (0.04 to 0.08) | 0.06 (0.04 to 0.09) | 0.10 (-0.24 to 0.45) | 0.565 | 5.27 (3.78 to 7.21) | 5.69 (3.95 to 7.67) | 0.08 (-0.26 to 0.42) | 0.662 |
| Non-Hodgkin lymphoma | 1.12 (0.89 to 1.42) | 0.75 (0.58 to 0.99) | -1.49 (-2.27 to -0.70) | 0 | 0.75 (0.60 to 0.94) | 0.19 (0.14 to 0.24) | -4.53 (-4.95 to -4.11) | 0 | 63.50 (50.69 to 80.04) | 15.66 (12.13 to 20.59) | -4.56 (-4.99 to -4.13) | 0 |
| Hodgkin lymphoma | 0.13 (0.05 to 0.19) | 0.05 (0.03 to 0.07) | -3.38 (-3.91 to -2.85) | 0 | 0.11 (0.04 to 0.16) | 0.01 (0.01 to 0.02) | -6.65 (-7.39 to -5.90) | 0 | 8.77 (3.09 to 13.08) | 1.06 (0.67 to 1.76) | -6.78 (-7.39 to -6.16) | 0 |
| Soft tissue and other extraosseous sarcomas | 0.36 (0.25 to 0.50) | 0.09 (0.06 to 0.14) | -4.59 (-5.10 to -4.08) | 0 | 0.15 (0.11 to 0.21) | 0.02 (0.02 to 0.04) | -5.77 (-6.66 to -4.86) | 0 | 12.94 (9.15 to 17.87) | 2.00 (1.36 to 3.12) | -5.87 (-6.56 to -5.18) | 0 |
| Kidney cancer | 1.02 (0.83 to 1.26) | 0.65 (0.48 to 0.83) | -1.59 (-2.14 to -1.04) | 0 | 0.40 (0.32 to 0.49) | 0.12 (0.09 to 0.15) | -4.01 (-4.73 to -3.27) | 0 | 34.84 (28.32 to 43.16) | 10.35 (7.43 to 13.30) | -4.01 (-4.72 to -3.30) | 0 |
| Liver cancer | 0.77 (0.63 to 0.95) | 0.27 (0.19 to 0.37) | -3.25 (-4.02 to -2.47) | 0 | 0.56 (0.46 to 0.68) | 0.12 (0.09 to 0.16) | -4.80 (-5.52 to -4.08) | 0 | 48.41 (39.79 to 59.39) | 10.09 (7.37 to 13.99) | -4.84 (-5.55 to -4.13) | 0 |
| Malignant neoplasm of bone and articular cartilage | 0.39 (0.25 to 0.69) | 0.58 (0.39 to 0.76) | 1.40 (0.97 to 1.82) | 0 | 0.14 (0.09 to 0.25) | 0.13 (0.09 to 0.16) | -0.09 (-0.73 to 0.55) | 0.773 | 11.33 (7.62 to 20.56) | 10.48 (7.15 to 13.63) | -0.07 (-0.75 to 0.61) | 0.829 |
| Thyroid cancer | 0.11 (0.08 to 0.14) | 0.17 (0.12 to 0.23) | 1.32 (0.83 to 1.81) | 0 | 0.01 (0.01 to 0.02) | 0.01 (0.00 to 0.01) | -2.34 (-2.90 to -1.77) | 0 | 1.17 (0.85 to 1.41) | 0.63 (0.41 to 0.85) | -2.03 (-2.58 to -1.47) | 0 |
| Eye cancer | 0.19 (0.10 to 0.32) | 0.50 (0.19 to 0.81) | 2.89 (2.37 to 3.41) | 0 | 0.10 (0.05 to 0.15) | 0.03 (0.01 to 0.05) | -3.56 (-4.20 to -2.92) | 0 | 8.64 (4.46 to 13.39) | 3.04 (1.26 to 4.65) | -3.29 (-3.93 to -2.65) | 0 |
| Acute myeloid leukemia | 1.59 (0.63 to 3.07) | 0.47 (0.29 to 0.77) | -3.94 (-4.38 to -3.50) | 0 | 1.53 (0.61 to 2.99) | 0.38 (0.24 to 0.63) | -4.47 (-4.94 to -3.99) | 0 | 131.40 (51.76 to 256.53) | 32.06 (19.79 to 52.80) | -4.55 (-5.03 to -4.07) | 0 |
| Chronic myeloid leukemia | 0.19 (0.07 to 0.33) | 0.04 (0.02 to 0.07) | -5.26 (-5.96 to -4.55) | 0 | 0.17 (0.06 to 0.30) | 0.01 (0.01 to 0.03) | -7.74 (-8.41 to -7.08) | 0 | 14.63 (5.43 to 25.70) | 1.20 (0.58 to 2.41) | -7.81 (-8.49 to -7.12) | 0 |
| Nasopharynx cancer | 0.15 (0.13 to 0.18) | 0.07 (0.05 to 0.10) | -2.23 (-2.93 to -1.52) | 0 | 0.09 (0.08 to 0.11) | 0.01 (0.01 to 0.02) | -6.10 (-6.50 to -5.70) | 0 | 7.30 (6.16 to 8.71) | 1.10 (0.81 to 1.47) | -6.06 (-6.51 to -5.61) | 0 |
| Other malignant neoplasms | 2.58 (1.06 to 3.57) | 1.68 (1.05 to 2.52) | -1.55 (-2.05 to -1.05) | 0 | 1.45 (0.61 to 2.00) | 0.33 (0.21 to 0.47) | -4.80 (-5.17 to -4.44) | 0 | 125.51 (51.67 to 172.79) | 28.49 (17.84 to 40.98) | -4.81 (-5.18 to -4.44) | 0 |
| Other leukemia | 0.61 (0.23 to 1.05) | 0.26 (0.09 to 0.46) | -2.86 (-3.13 to -2.59) | 0 | 0.21 (0.08 to 0.36) | 0.03 (0.01 to 0.06) | -5.92 (-6.14 to -5.69) | 0 | 18.19 (6.77 to 31.71) | 2.87 (1.07 to 5.15) | -5.88 (-6.09 to -5.68) | 0 |
| **Oceania** | | | | | | | | | | | | |
| Acute lymphoid leukemia | 0.74 (0.31 to 1.44) | 0.70 (0.32 to 1.37) | -0.20 (-0.71 to 0.31) | 0.439 | 0.69 (0.28 to 1.33) | 0.63 (0.28 to 1.22) | -0.29 (-0.80 to 0.22) | 0.264 | 58.29 (23.87 to 112.75) | 53.01 (24.03 to 103.36) | -0.29 (-0.79 to 0.21) | 0.249 |
| Brain and central nervous system cancer | 0.36 (0.18 to 0.60) | 0.41 (0.22 to 0.67) | 0.46 (-0.03 to 0.94) | 0.065 | 0.27 (0.14 to 0.47) | 0.32 (0.17 to 0.51) | 0.44 (-0.00 to 0.89) | 0.051 | 22.87 (11.50 to 39.06) | 26.44 (14.22 to 42.92) | 0.48 (0.05 to 0.92) | 0.03 |
| Neuroblastoma and other peripheral nervous cell tumors | 0.01 (0.00 to 0.02) | 0.01 (0.00 to 0.02) | -0.18 (-0.97 to 0.61) | 0.652 | 0.00 (0.00 to 0.01) | 0.00 (0.00 to 0.01) | -0.57 (-1.03 to -0.10) | 0.017 | 0.36 (0.19 to 0.67) | 0.30 (0.15 to 0.53) | -0.56 (-1.02 to -0.10) | 0.018 |
| Non-Hodgkin lymphoma | 0.88 (0.50 to 1.45) | 1.25 (0.67 to 2.18) | 1.09 (0.34 to 1.84) | 0.004 | 0.25 (0.14 to 0.41) | 0.30 (0.17 to 0.48) | 0.57 (0.02 to 1.13) | 0.043 | 21.63 (11.92 to 34.78) | 25.51 (14.70 to 41.43) | 0.58 (0.03 to 1.13) | 0.04 |
| Hodgkin lymphoma | 0.03 (0.01 to 0.05) | 0.03 (0.01 to 0.05) | -0.45 (-1.20 to 0.31) | 0.245 | 0.02 (0.01 to 0.03) | 0.01 (0.01 to 0.03) | -0.84 (-1.44 to -0.25) | 0.006 | 1.50 (0.71 to 2.50) | 1.13 (0.43 to 2.05) | -0.84 (-1.44 to -0.24) | 0.006 |
| Soft tissue and other extraosseous sarcomas | 0.10 (0.04 to 0.18) | 0.08 (0.04 to 0.16) | -0.68 (-1.31 to -0.04) | 0.037 | 0.05 (0.02 to 0.09) | 0.04 (0.02 to 0.07) | -0.84 (-1.45 to -0.23) | 0.007 | 3.91 (1.71 to 7.29) | 2.98 (1.37 to 6.02) | -0.85 (-1.45 to -0.24) | 0.006 |
| Kidney cancer | 0.13 (0.08 to 0.20) | 0.15 (0.09 to 0.25) | 0.57 (-0.06 to 1.21) | 0.074 | 0.05 (0.03 to 0.08) | 0.06 (0.04 to 0.10) | 0.55 (0.01 to 1.09) | 0.047 | 4.39 (2.74 to 7.05) | 5.16 (3.21 to 8.51) | 0.55 (0.00 to 1.09) | 0.048 |
| Liver cancer | 0.12 (0.07 to 0.22) | 0.09 (0.05 to 0.16) | -1.10 (-1.39 to -0.80) | 0 | 0.09 (0.05 to 0.18) | 0.07 (0.04 to 0.12) | -1.14 (-1.46 to -0.83) | 0 | 8.10 (4.69 to 15.19) | 5.73 (3.19 to 10.25) | -1.15 (-1.47 to -0.83) | 0 |
| Malignant neoplasm of bone and articular cartilage | 0.18 (0.08 to 0.37) | 0.21 (0.07 to 0.50) | 0.62 (0.35 to 0.89) | 0 | 0.08 (0.03 to 0.18) | 0.09 (0.03 to 0.21) | 0.33 (0.10 to 0.57) | 0.005 | 6.71 (2.79 to 14.22) | 7.45 (2.31 to 17.25) | 0.41 (0.03 to 0.78) | 0.035 |
| Thyroid cancer | 0.11 (0.07 to 0.15) | 0.14 (0.08 to 0.20) | 0.78 (0.29 to 1.28) | 0.002 | 0.02 (0.01 to 0.02) | 0.02 (0.01 to 0.03) | 0.33 (-0.01 to 0.68) | 0.058 | 1.34 (0.87 to 1.93) | 1.45 (0.83 to 2.22) | 0.35 (0.01 to 0.69) | 0.044 |
| Eye cancer | 0.05 (0.02 to 0.14) | 0.06 (0.02 to 0.19) | 0.71 (0.05 to 1.36) | 0.035 | 0.05 (0.01 to 0.13) | 0.05 (0.01 to 0.17) | 0.32 (-0.36 to 1.01) | 0.36 | 4.06 (1.23 to 11.64) | 4.54 (1.25 to 15.11) | 0.32 (-0.36 to 1.00) | 0.358 |
| Acute myeloid leukemia | 1.09 (0.53 to 1.82) | 1.05 (0.54 to 1.71) | -0.11 (-0.52 to 0.31) | 0.614 | 1.07 (0.52 to 1.79) | 1.02 (0.52 to 1.67) | -0.11 (-0.53 to 0.31) | 0.598 | 88.43 (43.29 to 148.51) | 85.06 (43.38 to 139.66) | -0.09 (-0.51 to 0.32) | 0.657 |
| Chronic myeloid leukemia | 0.32 (0.10 to 0.75) | 0.31 (0.11 to 0.65) | -0.08 (-0.83 to 0.67) | 0.828 | 0.31 (0.09 to 0.72) | 0.29 (0.10 to 0.59) | -0.20 (-0.94 to 0.53) | 0.587 | 25.22 (7.30 to 59.35) | 23.23 (8.27 to 48.60) | -0.26 (-0.97 to 0.46) | 0.478 |
| Nasopharynx cancer | 0.03 (0.01 to 0.04) | 0.02 (0.01 to 0.03) | -1.08 (-1.28 to -0.88) | 0 | 0.02 (0.01 to 0.03) | 0.01 (0.01 to 0.02) | -1.28 (-1.59 to -0.97) | 0 | 1.52 (0.82 to 2.61) | 1.01 (0.58 to 1.77) | -1.28 (-1.59 to -0.97) | 0 |
| Other malignant neoplasms | 0.73 (0.39 to 1.21) | 0.81 (0.44 to 1.36) | 0.33 (-0.11 to 0.78) | 0.137 | 0.47 (0.25 to 0.76) | 0.47 (0.26 to 0.76) | 0.03 (-0.34 to 0.41) | 0.861 | 40.28 (21.00 to 65.42) | 40.55 (22.09 to 65.75) | 0.04 (-0.33 to 0.40) | 0.845 |
| Other leukemia | 0.00 (0.00 to 0.01) | 0.00 (0.00 to 0.01) | 0.03 (-0.68 to 0.74) | 0.942 | 0.00 (0.00 to 0.00) | 0.00 (0.00 to 0.00) | -0.63 (-1.54 to 0.30) | 0.183 | 0.05 (0.01 to 0.21) | 0.04 (0.01 to 0.16) | -0.62 (-1.47 to 0.24) | 0.158 |
| **Southeast Asia** | | | | | | | | | | | | |
| Acute lymphoid leukemia | 2.33 (1.30 to 3.93) | 1.79 (1.19 to 2.32) | -0.87 (-1.00 to -0.75) | 0 | 2.14 (1.19 to 3.60) | 1.29 (0.85 to 1.65) | -1.65 (-1.79 to -1.51) | 0 | 179.15 (98.83 to 303.81) | 107.45 (70.98 to 138.24) | -1.66 (-1.81 to -1.52) | 0 |
| Brain and central nervous system cancer | 0.95 (0.56 to 1.42) | 1.03 (0.72 to 1.33) | 0.26 (0.16 to 0.36) | 0 | 0.70 (0.41 to 1.08) | 0.66 (0.45 to 0.84) | -0.23 (-0.31 to -0.16) | 0 | 58.67 (33.94 to 90.93) | 54.46 (37.64 to 70.23) | -0.25 (-0.32 to -0.17) | 0 |
| Neuroblastoma and other peripheral nervous cell tumors | 0.14 (0.08 to 0.22) | 0.20 (0.13 to 0.28) | 1.13 (0.95 to 1.31) | 0 | 0.06 (0.04 to 0.08) | 0.07 (0.05 to 0.09) | 0.59 (0.44 to 0.75) | 0 | 5.15 (3.45 to 7.37) | 6.20 (4.59 to 8.03) | 0.59 (0.44 to 0.74) | 0 |
| Non-Hodgkin lymphoma | 0.69 (0.44 to 0.94) | 0.62 (0.47 to 0.84) | -0.40 (-0.56 to -0.24) | 0 | 0.52 (0.32 to 0.70) | 0.27 (0.21 to 0.38) | -2.07 (-2.18 to -1.96) | 0 | 43.36 (26.40 to 59.23) | 22.75 (17.51 to 31.50) | -2.08 (-2.20 to -1.97) | 0 |
| Hodgkin lymphoma | 0.07 (0.04 to 0.10) | 0.06 (0.04 to 0.10) | -0.39 (-0.52 to -0.26) | 0 | 0.04 (0.02 to 0.06) | 0.02 (0.01 to 0.03) | -2.80 (-2.92 to -2.69) | 0 | 3.00 (1.88 to 4.68) | 1.26 (0.82 to 2.31) | -2.78 (-2.90 to -2.66) | 0 |
| Soft tissue and other extraosseous sarcomas | 0.40 (0.27 to 0.59) | 0.20 (0.14 to 0.31) | -2.27 (-2.45 to -2.08) | 0 | 0.19 (0.13 to 0.28) | 0.08 (0.05 to 0.12) | -2.89 (-3.05 to -2.73) | 0 | 15.93 (10.69 to 23.54) | 6.44 (4.62 to 9.84) | -2.92 (-3.08 to -2.76) | 0 |
| Kidney cancer | 0.40 (0.23 to 0.58) | 0.40 (0.31 to 0.51) | -0.06 (-0.28 to 0.16) | 0.617 | 0.15 (0.09 to 0.22) | 0.13 (0.09 to 0.16) | -0.65 (-0.86 to -0.43) | 0 | 13.27 (7.44 to 19.60) | 10.91 (8.24 to 14.14) | -0.65 (-0.86 to -0.44) | 0 |
| Liver cancer | 0.41 (0.27 to 0.54) | 0.17 (0.12 to 0.23) | -2.86 (-3.02 to -2.70) | 0 | 0.30 (0.20 to 0.39) | 0.12 (0.08 to 0.16) | -3.02 (-3.19 to -2.86) | 0 | 26.10 (17.12 to 33.99) | 10.02 (7.28 to 13.85) | -3.04 (-3.20 to -2.88) | 0 |
| Malignant neoplasm of bone and articular cartilage | 0.40 (0.22 to 0.55) | 0.47 (0.31 to 0.64) | 0.49 (0.32 to 0.66) | 0 | 0.16 (0.09 to 0.22) | 0.16 (0.10 to 0.21) | -0.10 (-0.21 to 0.01) | 0.079 | 13.32 (7.28 to 18.04) | 12.88 (8.50 to 17.29) | -0.10 (-0.21 to 0.02) | 0.091 |
| Thyroid cancer | 0.06 (0.05 to 0.08) | 0.08 (0.06 to 0.10) | 0.84 (0.61 to 1.08) | 0 | 0.01 (0.01 to 0.01) | 0.01 (0.00 to 0.01) | -0.94 (-1.09 to -0.80) | 0 | 0.67 (0.48 to 0.87) | 0.52 (0.40 to 0.63) | -0.84 (-0.99 to -0.68) | 0 |
| Eye cancer | 0.16 (0.06 to 0.28) | 0.22 (0.11 to 0.37) | 1.16 (0.85 to 1.47) | 0 | 0.09 (0.04 to 0.16) | 0.06 (0.03 to 0.09) | -1.48 (-1.69 to -1.28) | 0 | 8.20 (3.58 to 13.78) | 5.28 (2.27 to 8.30) | -1.45 (-1.66 to -1.24) | 0 |
| Acute myeloid leukemia | 0.86 (0.43 to 2.07) | 0.62 (0.40 to 0.97) | -1.07 (-1.21 to -0.92) | 0 | 0.83 (0.42 to 1.98) | 0.58 (0.38 to 0.91) | -1.14 (-1.29 to -0.99) | 0 | 69.64 (34.86 to 167.34) | 48.50 (31.73 to 76.09) | -1.15 (-1.31 to -0.99) | 0 |
| Chronic myeloid leukemia | 0.13 (0.05 to 0.30) | 0.04 (0.03 to 0.07) | -3.38 (-3.64 to -3.13) | 0 | 0.12 (0.05 to 0.29) | 0.04 (0.02 to 0.06) | -3.78 (-4.02 to -3.53) | 0 | 10.21 (4.05 to 24.23) | 3.07 (1.77 to 4.94) | -3.83 (-4.09 to -3.57) | 0 |
| Nasopharynx cancer | 0.06 (0.05 to 0.08) | 0.05 (0.04 to 0.06) | -0.87 (-1.07 to -0.67) | 0 | 0.04 (0.03 to 0.05) | 0.02 (0.02 to 0.03) | -2.22 (-2.40 to -2.04) | 0 | 3.43 (2.73 to 4.31) | 1.73 (1.38 to 2.25) | -2.21 (-2.39 to -2.03) | 0 |
| Other malignant neoplasms | 1.11 (0.52 to 1.80) | 1.07 (0.79 to 1.46) | -0.15 (-0.28 to -0.01) | 0.031 | 0.69 (0.33 to 1.10) | 0.42 (0.32 to 0.56) | -1.63 (-1.71 to -1.55) | 0 | 58.70 (27.36 to 93.71) | 35.48 (26.74 to 47.80) | -1.63 (-1.71 to -1.55) | 0 |
| Other leukemia | 0.10 (0.04 to 0.22) | 0.07 (0.04 to 0.15) | -1.25 (-1.49 to -1.01) | 0 | 0.04 (0.02 to 0.09) | 0.02 (0.01 to 0.04) | -2.93 (-3.13 to -2.74) | 0 | 3.33 (1.40 to 7.80) | 1.35 (0.72 to 3.25) | -2.92 (-3.11 to -2.73) | 0 |
| **Central Sub-Saharan Africa** | | | | | | | | | | | | |
| Acute lymphoid leukemia | 0.96 (0.40 to 1.87) | 0.61 (0.33 to 1.03) | -1.46 (-1.55 to -1.36) | 0 | 0.94 (0.39 to 1.85) | 0.57 (0.31 to 0.96) | -1.60 (-1.70 to -1.50) | 0 | 79.59 (32.37 to 157.48) | 47.66 (25.52 to 79.96) | -1.65 (-1.74 to -1.55) | 0 |
| Brain and central nervous system cancer | 0.34 (0.18 to 0.71) | 0.33 (0.20 to 0.50) | -0.11 (-0.23 to 0.01) | 0.062 | 0.29 (0.15 to 0.61) | 0.27 (0.17 to 0.40) | -0.30 (-0.42 to -0.18) | 0 | 24.81 (12.85 to 51.82) | 22.44 (13.97 to 33.71) | -0.33 (-0.45 to -0.22) | 0 |
| Neuroblastoma and other peripheral nervous cell tumors | 0.05 (0.02 to 0.11) | 0.04 (0.02 to 0.08) | -0.63 (-0.88 to -0.38) | 0 | 0.03 (0.01 to 0.05) | 0.02 (0.01 to 0.03) | -0.86 (-0.99 to -0.74) | 0 | 2.21 (0.84 to 4.41) | 1.69 (0.98 to 2.80) | -0.89 (-1.01 to -0.77) | 0 |
| Non-Hodgkin lymphoma | 0.84 (0.25 to 1.32) | 0.41 (0.23 to 0.62) | -2.27 (-2.34 to -2.20) | 0 | 0.75 (0.23 to 1.14) | 0.34 (0.19 to 0.50) | -2.57 (-2.64 to -2.49) | 0 | 64.35 (19.23 to 97.03) | 28.31 (15.80 to 41.95) | -2.63 (-2.70 to -2.56) | 0 |
| Hodgkin lymphoma | 0.06 (0.03 to 0.10) | 0.04 (0.02 to 0.07) | -0.92 (-1.05 to -0.78) | 0 | 0.05 (0.02 to 0.09) | 0.03 (0.02 to 0.05) | -1.65 (-1.77 to -1.53) | 0 | 3.83 (1.87 to 7.27) | 2.29 (1.28 to 4.04) | -1.67 (-1.79 to -1.55) | 0 |
| Soft tissue and other extraosseous sarcomas | 0.91 (0.46 to 1.57) | 0.39 (0.22 to 0.65) | -2.67 (-2.78 to -2.57) | 0 | 0.49 (0.25 to 0.83) | 0.18 (0.10 to 0.30) | -3.16 (-3.25 to -3.07) | 0 | 42.66 (22.08 to 72.06) | 15.53 (8.84 to 25.17) | -3.21 (-3.30 to -3.13) | 0 |
| Kidney cancer | 0.18 (0.08 to 0.31) | 0.13 (0.08 to 0.21) | -0.90 (-0.97 to -0.84) | 0 | 0.11 (0.05 to 0.19) | 0.07 (0.04 to 0.10) | -1.60 (-1.67 to -1.52) | 0 | 9.72 (4.23 to 16.85) | 5.86 (3.70 to 9.03) | -1.62 (-1.69 to -1.55) | 0 |
| Liver cancer | 0.54 (0.27 to 0.90) | 0.17 (0.08 to 0.34) | -3.59 (-3.74 to -3.44) | 0 | 0.40 (0.20 to 0.68) | 0.13 (0.06 to 0.26) | -3.55 (-3.70 to -3.40) | 0 | 34.95 (17.21 to 58.71) | 11.34 (4.92 to 22.16) | -3.58 (-3.73 to -3.43) | 0 |
| Malignant neoplasm of bone and articular cartilage | 0.31 (0.16 to 0.57) | 0.25 (0.12 to 0.42) | -0.65 (-0.85 to -0.46) | 0 | 0.16 (0.09 to 0.29) | 0.11 (0.06 to 0.18) | -1.19 (-1.40 to -0.97) | 0 | 13.04 (6.96 to 23.50) | 9.05 (4.52 to 14.81) | -1.20 (-1.41 to -0.98) | 0 |
| Thyroid cancer | 0.02 (0.01 to 0.04) | 0.02 (0.02 to 0.04) | 0.31 (0.15 to 0.47) | 0 | 0.01 (0.00 to 0.01) | 0.00 (0.00 to 0.01) | -0.93 (-1.10 to -0.77) | 0 | 0.48 (0.28 to 0.76) | 0.36 (0.25 to 0.51) | -0.90 (-1.07 to -0.74) | 0 |
| Eye cancer | 0.22 (0.10 to 0.42) | 0.17 (0.06 to 0.36) | -0.95 (-1.10 to -0.79) | 0 | 0.18 (0.08 to 0.34) | 0.11 (0.04 to 0.23) | -1.52 (-1.62 to -1.42) | 0 | 15.79 (7.08 to 29.20) | 9.82 (3.75 to 19.75) | -1.57 (-1.66 to -1.47) | 0 |
| Acute myeloid leukemia | 0.31 (0.10 to 0.89) | 0.20 (0.09 to 0.37) | -1.43 (-1.54 to -1.33) | 0 | 0.30 (0.10 to 0.90) | 0.19 (0.09 to 0.36) | -1.44 (-1.55 to -1.34) | 0 | 25.81 (8.75 to 77.06) | 16.26 (7.24 to 30.43) | -1.48 (-1.59 to -1.38) | 0 |
| Chronic myeloid leukemia | 0.03 (0.01 to 0.13) | 0.01 (0.00 to 0.03) | -3.89 (-4.06 to -3.71) | 0 | 0.03 (0.00 to 0.13) | 0.01 (0.00 to 0.03) | -4.02 (-4.15 to -3.90) | 0 | 2.76 (0.42 to 10.89) | 0.76 (0.22 to 2.17) | -4.09 (-4.20 to -3.97) | 0 |
| Nasopharynx cancer | 0.01 (0.01 to 0.02) | 0.01 (0.00 to 0.01) | -1.28 (-1.49 to -1.06) | 0 | 0.01 (0.01 to 0.02) | 0.01 (0.00 to 0.01) | -1.50 (-1.72 to -1.29) | 0 | 0.79 (0.47 to 1.22) | 0.50 (0.32 to 0.78) | -1.52 (-1.73 to -1.30) | 0 |
| Other malignant neoplasms | 0.71 (0.27 to 1.24) | 0.42 (0.26 to 0.64) | -1.66 (-1.74 to -1.58) | 0 | 0.64 (0.25 to 1.12) | 0.36 (0.23 to 0.54) | -1.83 (-1.93 to -1.72) | 0 | 55.05 (20.96 to 96.73) | 30.66 (19.35 to 45.61) | -1.87 (-1.97 to -1.77) | 0 |
| Other leukemia | 0.00 (0.00 to 0.01) | 0.00 (0.00 to 0.01) | 0.12 (0.02 to 0.22) | 0.014 | 0.00 (0.00 to 0.00) | 0.00 (0.00 to 0.00) | -0.99 (-1.14 to -0.83) | 0 | 0.12 (0.03 to 0.37) | 0.09 (0.02 to 0.22) | -0.98 (-1.15 to -0.81) | 0 |
| **Eastern Sub-Saharan Africa** | | | | | | | | | | | | |
| Acute lymphoid leukemia | 2.27 (1.12 to 3.93) | 1.48 (0.89 to 2.23) | -1.38 (-1.55 to -1.21) | 0 | 2.21 (1.07 to 3.83) | 1.35 (0.82 to 2.02) | -1.58 (-1.74 to -1.41) | 0 | 187.67 (91.01 to 325.90) | 113.49 (68.78 to 171.02) | -1.60 (-1.77 to -1.44) | 0 |
| Brain and central nervous system cancer | 0.93 (0.60 to 1.61) | 1.06 (0.71 to 1.55) | 0.43 (0.27 to 0.59) | 0 | 0.79 (0.51 to 1.38) | 0.84 (0.56 to 1.23) | 0.20 (0.01 to 0.38) | 0.038 | 67.88 (43.39 to 118.85) | 71.39 (47.89 to 104.90) | 0.18 (0.02 to 0.34) | 0.025 |
| Neuroblastoma and other peripheral nervous cell tumors | 0.24 (0.13 to 0.43) | 0.31 (0.13 to 0.55) | 0.86 (0.54 to 1.18) | 0 | 0.11 (0.08 to 0.19) | 0.13 (0.06 to 0.22) | 0.57 (0.39 to 0.75) | 0 | 9.89 (6.74 to 16.50) | 11.64 (5.67 to 19.31) | 0.57 (0.38 to 0.75) | 0 |
| Non-Hodgkin lymphoma | 2.45 (1.70 to 3.24) | 1.63 (1.03 to 2.42) | -1.30 (-1.42 to -1.18) | 0 | 2.08 (1.44 to 2.80) | 1.17 (0.79 to 1.71) | -1.82 (-1.94 to -1.70) | 0 | 177.03 (122.53 to 238.05) | 98.52 (65.86 to 144.22) | -1.85 (-1.98 to -1.73) | 0 |
| Hodgkin lymphoma | 0.48 (0.28 to 0.72) | 0.38 (0.19 to 0.63) | -0.74 (-0.88 to -0.61) | 0 | 0.41 (0.24 to 0.61) | 0.24 (0.12 to 0.40) | -1.61 (-1.74 to -1.47) | 0 | 33.41 (19.44 to 50.73) | 20.09 (10.00 to 33.27) | -1.62 (-1.75 to -1.49) | 0 |
| Soft tissue and other extraosseous sarcomas | 1.77 (1.16 to 2.87) | 0.86 (0.53 to 1.45) | -2.30 (-2.44 to -2.16) | 0 | 0.97 (0.64 to 1.52) | 0.39 (0.25 to 0.67) | -2.88 (-3.04 to -2.72) | 0 | 83.63 (55.72 to 131.45) | 33.32 (21.08 to 57.11) | -2.92 (-3.08 to -2.76) | 0 |
| Kidney cancer | 0.68 (0.43 to 0.96) | 0.62 (0.33 to 0.92) | -0.32 (-0.53 to -0.12) | 0.002 | 0.43 (0.26 to 0.60) | 0.30 (0.16 to 0.45) | -1.11 (-1.34 to -0.88) | 0 | 37.39 (23.23 to 52.46) | 26.32 (13.98 to 39.82) | -1.12 (-1.31 to -0.92) | 0 |
| Liver cancer | 0.59 (0.40 to 0.78) | 0.30 (0.17 to 0.52) | -2.12 (-2.28 to -1.95) | 0 | 0.43 (0.29 to 0.57) | 0.22 (0.13 to 0.38) | -2.11 (-2.29 to -1.92) | 0 | 37.55 (25.53 to 49.70) | 19.26 (11.35 to 33.38) | -2.14 (-2.31 to -1.97) | 0 |
| Malignant neoplasm of bone and articular cartilage | 0.81 (0.54 to 1.24) | 0.82 (0.51 to 1.36) | 0.06 (-0.06 to 0.17) | 0.34 | 0.42 (0.29 to 0.63) | 0.35 (0.22 to 0.57) | -0.60 (-0.71 to -0.50) | 0 | 34.21 (23.26 to 51.67) | 28.27 (17.89 to 46.58) | -0.60 (-0.70 to -0.49) | 0 |
| Thyroid cancer | 0.07 (0.05 to 0.09) | 0.09 (0.07 to 0.14) | 1.02 (0.90 to 1.14) | 0 | 0.02 (0.01 to 0.02) | 0.01 (0.01 to 0.02) | -0.69 (-0.82 to -0.57) | 0 | 1.45 (1.04 to 1.99) | 1.19 (0.83 to 1.64) | -0.64 (-0.76 to -0.52) | 0 |
| Eye cancer | 1.11 (0.62 to 1.65) | 0.88 (0.50 to 1.44) | -0.74 (-0.85 to -0.64) | 0 | 0.94 (0.55 to 1.35) | 0.59 (0.38 to 0.91) | -1.43 (-1.53 to -1.33) | 0 | 82.25 (48.37 to 117.97) | 52.02 (33.58 to 79.39) | -1.44 (-1.54 to -1.33) | 0 |
| Acute myeloid leukemia | 0.47 (0.16 to 1.09) | 0.33 (0.15 to 0.51) | -1.13 (-1.30 to -0.96) | 0 | 0.46 (0.16 to 1.08) | 0.32 (0.14 to 0.51) | -1.15 (-1.32 to -0.97) | 0 | 39.81 (13.31 to 92.68) | 27.37 (12.10 to 43.64) | -1.16 (-1.35 to -0.97) | 0 |
| Chronic myeloid leukemia | 0.04 (0.01 to 0.13) | 0.02 (0.01 to 0.04) | -2.38 (-2.52 to -2.24) | 0 | 0.04 (0.01 to 0.12) | 0.02 (0.01 to 0.04) | -2.53 (-2.68 to -2.38) | 0 | 3.48 (0.67 to 9.99) | 1.54 (0.61 to 3.24) | -2.57 (-2.73 to -2.41) | 0 |
| Nasopharynx cancer | 0.09 (0.06 to 0.11) | 0.07 (0.05 to 0.10) | -0.54 (-0.78 to -0.31) | 0 | 0.08 (0.06 to 0.10) | 0.06 (0.04 to 0.09) | -0.81 (-1.04 to -0.57) | 0 | 6.20 (4.43 to 8.06) | 4.83 (3.27 to 6.97) | -0.81 (-1.04 to -0.58) | 0 |
| Other malignant neoplasms | 1.45 (0.85 to 2.22) | 1.02 (0.63 to 1.48) | -1.11 (-1.27 to -0.95) | 0 | 1.30 (0.76 to 1.99) | 0.86 (0.54 to 1.26) | -1.31 (-1.47 to -1.14) | 0 | 111.95 (65.53 to 171.19) | 73.33 (46.06 to 107.70) | -1.33 (-1.49 to -1.17) | 0 |
| Other leukemia | 0.42 (0.12 to 0.93) | 0.37 (0.13 to 0.71) | -0.45 (-0.59 to -0.32) | 0 | 0.22 (0.06 to 0.49) | 0.12 (0.04 to 0.24) | -1.89 (-2.02 to -1.75) | 0 | 19.38 (5.40 to 42.59) | 10.79 (3.47 to 21.46) | -1.86 (-1.99 to -1.72) | 0 |
| **Southern Sub-Saharan Africa** | | | | | | | | | | | | |
| Acute lymphoid leukemia | 0.78 (0.50 to 1.17) | 0.86 (0.58 to 1.16) | 0.34 (-0.40 to 1.08) | 0.374 | 0.70 (0.45 to 1.04) | 0.74 (0.49 to 0.99) | 0.18 (-0.47 to 0.83) | 0.589 | 58.80 (37.59 to 87.18) | 60.83 (40.66 to 82.23) | 0.14 (-0.51 to 0.81) | 0.666 |
| Brain and central nervous system cancer | 0.57 (0.42 to 0.81) | 0.81 (0.58 to 1.07) | 1.02 (0.47 to 1.58) | 0 | 0.43 (0.32 to 0.61) | 0.60 (0.43 to 0.79) | 1.00 (0.43 to 1.56) | 0.001 | 35.96 (26.50 to 50.86) | 50.20 (35.78 to 66.13) | 0.99 (0.44 to 1.55) | 0 |
| Neuroblastoma and other peripheral nervous cell tumors | 0.13 (0.08 to 0.20) | 0.18 (0.12 to 0.28) | 1.16 (0.30 to 2.02) | 0.008 | 0.05 (0.04 to 0.08) | 0.07 (0.05 to 0.10) | 1.00 (0.36 to 1.64) | 0.002 | 4.81 (3.38 to 6.68) | 6.43 (4.63 to 8.68) | 0.99 (0.37 to 1.63) | 0.002 |
| Non-Hodgkin lymphoma | 0.60 (0.41 to 0.81) | 1.03 (0.74 to 1.39) | 1.74 (0.82 to 2.67) | 0 | 0.36 (0.25 to 0.48) | 0.54 (0.37 to 0.73) | 1.27 (0.49 to 2.07) | 0.001 | 30.44 (21.17 to 40.01) | 44.92 (30.35 to 61.29) | 1.24 (0.45 to 2.04) | 0.002 |
| Hodgkin lymphoma | 0.07 (0.04 to 0.11) | 0.11 (0.06 to 0.16) | 1.17 (0.25 to 2.09) | 0.012 | 0.05 (0.03 to 0.07) | 0.06 (0.03 to 0.08) | 0.53 (-0.16 to 1.22) | 0.134 | 3.84 (2.26 to 5.80) | 4.52 (2.55 to 6.78) | 0.52 (-0.17 to 1.22) | 0.142 |
| Soft tissue and other extraosseous sarcomas | 0.39 (0.26 to 0.55) | 0.42 (0.28 to 0.59) | 0.24 (-0.82 to 1.31) | 0.656 | 0.17 (0.12 to 0.23) | 0.17 (0.12 to 0.25) | 0.07 (-0.80 to 0.95) | 0.875 | 14.63 (10.02 to 20.00) | 14.62 (9.91 to 20.89) | 0.01 (-0.87 to 0.91) | 0.976 |
| Kidney cancer | 0.33 (0.21 to 0.45) | 0.42 (0.33 to 0.53) | 0.80 (0.08 to 1.53) | 0.03 | 0.16 (0.11 to 0.22) | 0.17 (0.13 to 0.22) | 0.24 (-0.41 to 0.89) | 0.472 | 14.08 (9.25 to 19.07) | 15.09 (11.60 to 19.06) | 0.23 (-0.40 to 0.87) | 0.473 |
| Liver cancer | 0.17 (0.11 to 0.25) | 0.19 (0.13 to 0.27) | 0.22 (-0.45 to 0.90) | 0.518 | 0.13 (0.08 to 0.19) | 0.15 (0.11 to 0.21) | 0.31 (-0.39 to 1.02) | 0.385 | 11.18 (6.95 to 16.21) | 12.88 (9.01 to 17.90) | 0.28 (-0.42 to 0.98) | 0.437 |
| Malignant neoplasm of bone and articular cartilage | 0.32 (0.21 to 0.46) | 0.34 (0.24 to 0.48) | 0.03 (-0.74 to 0.80) | 0.942 | 0.13 (0.09 to 0.18) | 0.13 (0.09 to 0.18) | -0.11 (-0.72 to 0.51) | 0.728 | 10.62 (6.96 to 14.70) | 10.37 (7.46 to 14.76) | -0.13 (-0.74 to 0.49) | 0.69 |
| Thyroid cancer | 0.08 (0.06 to 0.10) | 0.11 (0.08 to 0.14) | 0.97 (-0.26 to 2.21) | 0.122 | 0.01 (0.01 to 0.01) | 0.01 (0.01 to 0.02) | 0.28 (-0.47 to 1.03) | 0.47 | 0.99 (0.72 to 1.23) | 1.07 (0.83 to 1.33) | 0.29 (-0.47 to 1.06) | 0.458 |
| Eye cancer | 0.20 (0.10 to 0.36) | 0.37 (0.15 to 0.68) | 1.77 (1.18 to 2.38) | 0 | 0.10 (0.05 to 0.16) | 0.14 (0.06 to 0.26) | 1.01 (0.33 to 1.70) | 0.004 | 8.68 (4.57 to 13.64) | 12.29 (5.12 to 22.65) | 1.02 (0.45 to 1.59) | 0 |
| Acute myeloid leukemia | 0.31 (0.16 to 0.57) | 0.32 (0.18 to 0.51) | 0.14 (-0.57 to 0.85) | 0.702 | 0.30 (0.15 to 0.55) | 0.31 (0.17 to 0.49) | 0.11 (-0.58 to 0.80) | 0.756 | 25.32 (12.68 to 46.49) | 25.74 (14.10 to 40.70) | 0.08 (-0.60 to 0.77) | 0.819 |
| Chronic myeloid leukemia | 0.00 (0.00 to 0.01) | 0.00 (0.00 to 0.01) | -1.00 (-1.66 to -0.34) | 0.003 | 0.00 (0.00 to 0.01) | 0.00 (0.00 to 0.01) | -1.21 (-1.82 to -0.61) | 0 | 0.30 (0.10 to 0.71) | 0.20 (0.06 to 0.47) | -1.26 (-1.88 to -0.64) | 0 |
| Nasopharynx cancer | 0.01 (0.01 to 0.02) | 0.02 (0.01 to 0.02) | 0.86 (0.36 to 1.38) | 0.001 | 0.01 (0.01 to 0.01) | 0.01 (0.01 to 0.02) | 0.66 (0.00 to 1.32) | 0.049 | 0.90 (0.69 to 1.15) | 1.11 (0.86 to 1.53) | 0.66 (0.00 to 1.32) | 0.049 |
| Other malignant neoplasms | 0.63 (0.44 to 0.84) | 0.66 (0.50 to 0.85) | 0.08 (-0.42 to 0.58) | 0.754 | 0.50 (0.35 to 0.67) | 0.52 (0.39 to 0.67) | 0.10 (-0.44 to 0.65) | 0.711 | 42.76 (30.09 to 57.04) | 43.62 (32.66 to 56.86) | 0.07 (-0.48 to 0.62) | 0.802 |
| Other leukemia | 0.02 (0.00 to 0.04) | 0.02 (0.00 to 0.04) | 0.06 (-0.25 to 0.37) | 0.719 | 0.01 (0.00 to 0.01) | 0.00 (0.00 to 0.01) | -0.79 (-1.05 to -0.52) | 0 | 0.53 (0.13 to 1.18) | 0.41 (0.07 to 0.90) | -0.79 (-1.05 to -0.54) | 0 |
| **Western Sub-Saharan Africa** | | | | | | | | | | | | |
| Acute lymphoid leukemia | 1.04 (0.50 to 1.65) | 0.89 (0.33 to 1.29) | -0.53 (-0.68 to -0.39) | 0 | 1.00 (0.49 to 1.59) | 0.80 (0.31 to 1.15) | -0.73 (-0.96 to -0.49) | 0 | 85.44 (41.42 to 135.71) | 67.98 (25.56 to 97.69) | -0.75 (-0.98 to -0.51) | 0 |
| Brain and central nervous system cancer | 0.43 (0.28 to 0.64) | 0.53 (0.25 to 0.72) | 0.65 (0.55 to 0.75) | 0 | 0.35 (0.23 to 0.54) | 0.41 (0.20 to 0.56) | 0.46 (0.36 to 0.57) | 0 | 30.39 (19.81 to 46.69) | 35.00 (16.79 to 47.56) | 0.45 (0.35 to 0.55) | 0 |
| Neuroblastoma and other peripheral nervous cell tumors | 0.17 (0.05 to 0.33) | 0.29 (0.08 to 0.54) | 1.63 (1.26 to 2.00) | 0 | 0.08 (0.02 to 0.12) | 0.12 (0.03 to 0.21) | 1.30 (1.09 to 1.52) | 0 | 6.87 (1.96 to 10.86) | 10.40 (2.64 to 18.24) | 1.29 (1.08 to 1.51) | 0 |
| Non-Hodgkin lymphoma | 1.81 (1.22 to 2.45) | 1.47 (0.85 to 2.14) | -0.69 (-0.89 to -0.48) | 0 | 1.51 (1.02 to 2.01) | 1.02 (0.65 to 1.39) | -1.28 (-1.45 to -1.12) | 0 | 128.74 (86.92 to 171.80) | 86.18 (54.16 to 117.91) | -1.31 (-1.48 to -1.15) | 0 |
| Hodgkin lymphoma | 0.43 (0.08 to 0.82) | 0.36 (0.07 to 0.68) | -0.60 (-0.84 to -0.37) | 0 | 0.35 (0.07 to 0.66) | 0.23 (0.05 to 0.43) | -1.38 (-1.56 to -1.20) | 0 | 28.45 (5.29 to 54.09) | 18.49 (3.65 to 34.86) | -1.38 (-1.56 to -1.20) | 0 |
| Soft tissue and other extraosseous sarcomas | 1.03 (0.66 to 1.76) | 0.64 (0.39 to 1.09) | -1.55 (-1.69 to -1.41) | 0 | 0.54 (0.36 to 0.90) | 0.29 (0.18 to 0.49) | -1.93 (-2.15 to -1.71) | 0 | 46.97 (31.04 to 78.25) | 25.39 (15.66 to 42.45) | -1.95 (-2.17 to -1.73) | 0 |
| Kidney cancer | 0.57 (0.42 to 0.73) | 0.74 (0.46 to 1.04) | 0.81 (0.66 to 0.96) | 0 | 0.36 (0.26 to 0.47) | 0.38 (0.24 to 0.53) | 0.16 (0.00 to 0.32) | 0.045 | 31.39 (22.55 to 41.17) | 33.31 (20.88 to 46.26) | 0.16 (0.00 to 0.32) | 0.045 |
| Liver cancer | 0.74 (0.52 to 0.98) | 0.37 (0.27 to 0.50) | -2.19 (-2.34 to -2.05) | 0 | 0.53 (0.38 to 0.71) | 0.26 (0.19 to 0.35) | -2.23 (-2.38 to -2.09) | 0 | 46.79 (33.43 to 62.48) | 23.32 (16.76 to 31.01) | -2.23 (-2.38 to -2.09) | 0 |
| Malignant neoplasm of bone and articular cartilage | 0.72 (0.47 to 1.03) | 0.80 (0.53 to 1.12) | 0.35 (0.15 to 0.56) | 0.001 | 0.32 (0.22 to 0.46) | 0.31 (0.21 to 0.42) | -0.20 (-0.45 to 0.05) | 0.114 | 26.89 (18.06 to 38.06) | 25.37 (17.36 to 34.92) | -0.20 (-0.45 to 0.06) | 0.132 |
| Thyroid cancer | 0.02 (0.01 to 0.02) | 0.02 (0.01 to 0.03) | 1.15 (0.95 to 1.34) | 0 | 0.00 (0.00 to 0.01) | 0.00 (0.00 to 0.01) | 0.15 (-0.02 to 0.32) | 0.079 | 0.35 (0.24 to 0.48) | 0.37 (0.22 to 0.53) | 0.19 (0.02 to 0.37) | 0.025 |
| Eye cancer | 0.39 (0.18 to 0.58) | 0.35 (0.13 to 0.58) | -0.41 (-0.59 to -0.23) | 0 | 0.37 (0.18 to 0.55) | 0.26 (0.11 to 0.42) | -1.13 (-1.37 to -0.90) | 0 | 32.67 (15.54 to 47.90) | 23.10 (9.61 to 36.81) | -1.14 (-1.37 to -0.91) | 0 |
| Acute myeloid leukemia | 0.25 (0.09 to 0.65) | 0.20 (0.08 to 0.35) | -0.66 (-0.74 to -0.57) | 0 | 0.24 (0.08 to 0.63) | 0.20 (0.08 to 0.34) | -0.67 (-0.75 to -0.58) | 0 | 21.02 (7.34 to 54.92) | 16.86 (6.71 to 29.57) | -0.68 (-0.77 to -0.59) | 0 |
| Chronic myeloid leukemia | 0.03 (0.01 to 0.11) | 0.01 (0.00 to 0.03) | -2.63 (-2.80 to -2.47) | 0 | 0.03 (0.01 to 0.11) | 0.01 (0.00 to 0.03) | -2.77 (-2.93 to -2.60) | 0 | 2.68 (0.62 to 9.53) | 1.11 (0.32 to 2.73) | -2.79 (-2.95 to -2.63) | 0 |
| Nasopharynx cancer | 0.02 (0.01 to 0.03) | 0.02 (0.01 to 0.03) | -0.56 (-0.79 to -0.33) | 0 | 0.02 (0.01 to 0.02) | 0.01 (0.01 to 0.02) | -0.77 (-1.06 to -0.49) | 0 | 1.47 (1.05 to 1.94) | 1.16 (0.70 to 1.70) | -0.77 (-1.06 to -0.49) | 0 |
| Other malignant neoplasms | 0.89 (0.51 to 1.21) | 0.68 (0.41 to 1.03) | -0.87 (-1.00 to -0.74) | 0 | 0.79 (0.45 to 1.06) | 0.56 (0.34 to 0.84) | -1.06 (-1.17 to -0.94) | 0 | 67.97 (38.50 to 91.78) | 48.49 (29.44 to 72.13) | -1.07 (-1.18 to -0.96) | 0 |
| Other leukemia | 0.00 (0.00 to 0.00) | 0.00 (0.00 to 0.00) | 0.24 (0.05 to 0.42) | 0.011 | 0.00 (0.00 to 0.00) | 0.00 (0.00 to 0.00) | -0.93 (-1.12 to -0.74) | 0 | 0.01 (0.00 to 0.02) | 0.01 (0.00 to 0.01) | -0.91 (-1.10 to -0.73) | 0 |

Estimates are for individuals aged 0-14 years. Rates are reported per 100000 population. UI=uncertainty interval. CI=confidence interval. ASIR=age-standardised incidence rates. ASMR=age-standardised mortality rates. ASDR=age-standardised disability-adjusted life years rates. AAPC=average annual percent change. SDI=Socio-demographic Index. DALY=disability-adjusted life year.
