# Supplementary material for: Childhood cancer burden and health inequality: A systematic analysis from the global burden of diseases study 2021
Source: PLoS One. 2026 Jan 27;21(1):e0341303. doi: 10.1371/journal.pone.0341303 (PMC12843563; doi:10.1371/journal.pone.0341303)
Supplement: S5 Table — (DOCX) [file pone.0341303.s017.docx]

**S5 Table. Slope index of inequality and concentration index in global incidence, deaths, and DALYs of childhood cancer and its subtypes in 2021**

| **Global** | **Slope index of inequality** | | | | | | **Concentration index** | | | | | |
| --- | --- | --- | --- | --- | --- | --- | --- | --- | --- | --- | --- | --- |
|  | **Incidence** | | **Deaths** | | **DALYs** | | **Incidence** | | **Deaths** | | **DALYs** | |
|  | 1990 | 2021 | 1990 | 2021 | 1990 | 2021 | 1990 | 2021 | 1990 | 2021 | 1990 | 2021 |
| Neoplasms | 117.90 (91.54 to 144.27) | 123.25 (94.30 to 152.19) | -3.10 (-4.03 to -2.17) | -2.85 (-3.38 to -2.32) | -272.74 (-351.77 to -193.70) | -238.46 (-282.48 to -194.44) | 0.18 (0.14 to 0.22) | 0.21 (0.17 to 0.26) | -0.03 (-0.06 to 0.01) | -0.13 (-0.16 to -0.11) | -0.03 (-0.06 to 0.01) | -0.13 (-0.16 to -0.11) |
| Acute lymphoid leukemia | 1.30 (0.75 to 1.86) | 1.70 (1.18 to 2.23) | -0.31 (-0.63 to 0.01) | -0.67 (-0.83 to -0.52) | -26.96 (-53.61 to -0.30) | -54.77 (-67.55 to -42.00) | 0.17 (0.13 to 0.22) | 0.32 (0.26 to 0.38) | 0.03 (-0.02 to 0.08) | -0.07 (-0.11 to -0.03) | 0.03 (-0.02 to 0.08) | -0.07 (-0.11 to -0.03) |
| Brain and central nervous system cancer | 1.82 (1.39 to 2.26) | 1.91 (1.49 to 2.32) | 0.57 (0.35 to 0.79) | 0.22 (0.06 to 0.38) | 46.87 (28.47 to 65.27) | 17.79 (4.12 to 31.46) | 0.21 (0.17 to 0.25) | 0.26 (0.22 to 0.30) | 0.12 (0.08 to 0.17) | 0.08 (0.04 to 0.11) | 0.12 (0.08 to 0.17) | 0.07 (0.04 to 0.11) |
| Neuroblastoma and other peripheral nervous cell tumors | 0.24 (0.16 to 0.32) | 0.25 (0.17 to 0.33) | 0.07 (0.04 to 0.10) | 0.05 (0.03 to 0.08) | 5.76 (3.12 to 8.40) | 4.68 (2.33 to 7.04) | 0.25 (0.19 to 0.30) | 0.06 (0.00 to 0.12) | 0.15 (0.10 to 0.20) | -0.03 (-0.09 to 0.04) | 0.15 (0.10 to 0.20) | -0.03 (-0.09 to 0.03) |
| Non-Hodgkin lymphoma | -0.02 (-0.32 to 0.28) | 0.60 (0.29 to 0.90) | -0.91 (-1.06 to -0.77) | -0.61 (-0.69 to -0.54) | -77.77 (-89.89 to -65.66) | -49.90 (-56.52 to -43.29) | -0.03 (-0.07 to 0.02) | -0.06 (-0.11 to -0.00) | -0.23 (-0.28 to -0.19) | -0.37 (-0.43 to -0.30) | -0.24 (-0.28 to -0.19) | -0.37 (-0.43 to -0.30) |
| Hodgkin lymphoma | 0.10 (0.01 to 0.18) | 0.05 (-0.01 to 0.11) | -0.07 (-0.10 to -0.04) | -0.06 (-0.07 to -0.05) | -5.88 (-8.15 to -3.60) | -4.72 (-5.79 to -3.65) | -0.08 (-0.14 to -0.02) | -0.20 (-0.27 to -0.13) | -0.32 (-0.38 to -0.26) | -0.44 (-0.52 to -0.35) | -0.32 (-0.38 to -0.26) | -0.43 (-0.52 to -0.34) |
| Soft tissue and other extraosseous sarcomas | -0.54 (-0.68 to -0.41) | -0.12 (-0.22 to -0.02) | -0.37 (-0.43 to -0.32) | -0.13 (-0.16 to -0.10) | -32.40 (-36.99 to -27.81) | -11.12 (-13.97 to -8.26) | -0.21 (-0.26 to -0.16) | -0.21 (-0.27 to -0.16) | -0.32 (-0.37 to -0.26) | -0.33 (-0.39 to -0.28) | -0.32 (-0.37 to -0.26) | -0.34 (-0.40 to -0.28) |
| Kidney cancer | 0.08 (-0.04 to 0.20) | 0.03 (-0.07 to 0.14) | -0.13 (-0.18 to -0.08) | -0.16 (-0.20 to -0.13) | -11.47 (-15.44 to -7.49) | -14.17 (-17.05 to -11.28) | 0.14 (0.09 to 0.18) | 0.03 (-0.02 to 0.08) | -0.02 (-0.07 to 0.03) | -0.24 (-0.30 to -0.18) | -0.02 (-0.07 to 0.03) | -0.24 (-0.30 to -0.18) |
| Liver cancer | -0.48 (-0.56 to -0.40) | -0.17 (-0.22 to -0.12) | -0.38 (-0.43 to -0.32) | -0.17 (-0.19 to -0.14) | -33.30 (-38.15 to -28.44) | -14.66 (-16.84 to -12.48) | -0.11 (-0.16 to -0.05) | -0.13 (-0.19 to -0.07) | -0.13 (-0.18 to -0.07) | -0.25 (-0.31 to -0.19) | -0.13 (-0.19 to -0.07) | -0.25 (-0.31 to -0.19) |
| Malignant neoplasm of bone and articular cartilage | 0.04 (-0.07 to 0.15) | -0.27 (-0.37 to -0.16) | -0.12 (-0.15 to -0.08) | -0.20 (-0.23 to -0.17) | -9.46 (-12.74 to -6.17) | -16.39 (-18.78 to -13.99) | 0.06 (0.03 to 0.10) | -0.04 (-0.07 to -0.02) | -0.07 (-0.10 to -0.04) | -0.17 (-0.19 to -0.15) | -0.07 (-0.10 to -0.03) | -0.17 (-0.19 to -0.14) |
| Thyroid cancer | 0.08 (0.07 to 0.10) | 0.05 (0.03 to 0.07) | -0.00 (-0.00 to 0.00) | -0.01 (-0.01 to -0.01) | -0.02 (-0.16 to 0.12) | -0.48 (-0.59 to -0.37) | 0.24 (0.21 to 0.27) | 0.16 (0.12 to 0.19) | -0.01 (-0.05 to 0.02) | -0.16 (-0.20 to -0.11) | 0.00 (-0.04 to 0.04) | -0.13 (-0.18 to -0.09) |
| Eye cancer | -0.17 (-0.26 to -0.07) | -0.11 (-0.21 to -0.01) | -0.36 (-0.40 to -0.32) | -0.24 (-0.26 to -0.22) | -31.47 (-34.96 to -27.97) | -20.86 (-22.93 to -18.80) | -0.04 (-0.12 to 0.04) | -0.04 (-0.10 to 0.02) | -0.40 (-0.49 to -0.30) | -0.48 (-0.56 to -0.39) | -0.40 (-0.49 to -0.30) | -0.47 (-0.56 to -0.38) |
| Acute myeloid leukemia | 0.24 (0.10 to 0.38) | 0.07 (-0.03 to 0.16) | 0.17 (0.03 to 0.30) | -0.02 (-0.11 to 0.07) | 12.87 (1.62 to 24.13) | -2.38 (-9.90 to 5.14) | 0.09 (0.05 to 0.13) | 0.05 (0.01 to 0.08) | 0.08 (0.04 to 0.12) | 0.00 (-0.03 to 0.04) | 0.08 (0.03 to 0.12) | -0.00 (-0.04 to 0.04) |
| Chronic myeloid leukemia | 0.02 (-0.00 to 0.05) | 0.01 (0.00 to 0.02) | -0.01 (-0.03 to 0.02) | -0.01 (-0.02 to -0.00) | -0.84 (-2.95 to 1.27) | -0.89 (-1.43 to -0.35) | -0.00 (-0.05 to 0.05) | 0.00 (-0.07 to 0.08) | -0.07 (-0.12 to -0.02) | -0.16 (-0.25 to -0.08) | -0.07 (-0.12 to -0.02) | -0.17 (-0.26 to -0.08) |
| Nasopharynx cancer | 0.01 (-0.00 to 0.02) | 0.00 (-0.01 to 0.01) | -0.01 (-0.01 to 0.00) | -0.01 (-0.01 to -0.01) | -0.46 (-1.00 to 0.07) | -0.78 (-1.17 to -0.39) | -0.03 (-0.09 to 0.03) | -0.00 (-0.06 to 0.06) | -0.13 (-0.18 to -0.08) | -0.23 (-0.30 to -0.16) | -0.13 (-0.18 to -0.07) | -0.23 (-0.30 to -0.16) |
| Other malignant neoplasms | -0.07 (-0.26 to 0.12) | 0.05 (-0.10 to 0.20) | -0.58 (-0.70 to -0.46) | -0.48 (-0.55 to -0.41) | -50.39 (-60.54 to -40.25) | -40.71 (-46.55 to -34.86) | 0.07 (0.03 to 0.11) | 0.08 (0.05 to 0.12) | -0.06 (-0.09 to -0.02) | -0.21 (-0.24 to -0.18) | -0.06 (-0.09 to -0.02) | -0.21 (-0.24 to -0.18) |
| Other leukemia | -0.02 (-0.08 to 0.03) | -0.02 (-0.05 to 0.02) | -0.03 (-0.05 to -0.01) | -0.01 (-0.02 to -0.00) | -2.67 (-4.31 to -1.04) | -0.80 (-1.39 to -0.22) | -0.12 (-0.18 to -0.05) | -0.11 (-0.18 to -0.04) | -0.22 (-0.28 to -0.15) | -0.29 (-0.37 to -0.20) | -0.22 (-0.28 to -0.15) | -0.29 (-0.37 to -0.20) |

DALYs = disability-adjusted life year.
